# Innovative Approaches to Improve Public Health Practice in the Eastern Mediterranean Region: Findings From the Sixth Eastern Mediterranean Public Health Network Regional Conference

**DOI:** 10.2196/11382

**Published:** 2019-03-07

**Authors:** Bashiruddin Noormal, Elmuez Eltayeb, Mohannad Al Nsour, Ezzeddine Mohsni, Yousef Khader, Mark Salter, Scott McNabb, Dionisio Herrera Guibert, Salman Rawaf, Amrish Baidjoe, Aamer Ikram, Christophe Longuet, Abdulwahed Al Serouri, Faris Lami, Asmae Khattabi, Sami AlMudarra, Ibrahim Iblan, Sahar Samy, Nissaf Bouafif ép Ben Alaya, Qahtan Al-Salihi

**Affiliations:** 1 Ministry of Public Health Afghanistan Afghanistan Afghanistan; 2 Federal Ministry of Health Khartoum Sudan; 3 Global Health Development Amman Jordan; 4 Jordan University of Science and Technology Irbid Jordan; 5 Public Health England London United Kingdom; 6 Emory University, Rollins School of Public Health North Carolina, NC United States; 7 Training Programs in Epidemiology and Public Health Interventions Network Decatur, GA United States; 8 Imperial College London London United Kingdom; 9 National Institute of Health Islamabad Pakistan; 10 Connecting Organizations for Regional Disease Surveillance Lyon France; 11 Yemen Field Epidemiology Training Program Sanaa Yemen; 12 Iraq Field Epidemiology Training Program Baghdad Iraq; 13 Ecole Nationale de Santé Publique Rabat Morocco; 14 Saudi Field Epidemiology Training Program Riyadh Saudi Arabia; 15 Field Epidemiology Training Program Amman Jordan; 16 Ministry of Health and Population Cairo Egypt; 17 National Observatory of New and Emerging Diseases Tunis Tunisia; 18 Iraq Ministry of Health Baghdad Iraq

**Keywords:** workshops, public health, one health, capacity building

## Abstract

Public health professionals in the Eastern Mediterranean region (EMR) have limited access to continuing education, including workshops and conferences in public health. Held under the theme *Innovative Approaches: Adapting to the Current EMR Context*, the Eastern Mediterranean Public Health Network (EMPHNET) organized and conducted the *Sixth EMPHNET Regional Conference* from March 26 to 29, 2018. This paper summarizes the key activities including workshops, roundtable discussions, oral and poster presentations, keynote speeches, and side meetings. Before the opening, 5 preconference workshops were held: “Field Epidemiology Training Program (FETP) Accreditation,” “Innovative Public Health Surveillance,” “Human and Animal Brucellosis,” “Rapid Response Teams,” and “Polio Transition and Routine Immunization.” The conference hosted 6 roundtable discussions: “Consolidation of the FETP Network,” “One Health to Achieve Global Health Security,” “Polio Eradication Efforts and Transition Planning for Measles Elimination,” “Mobile Data Collection and Other Innovative Tools to Enhance Decision Making,” “Confronting *Candida auris*: An Emerging Multidrug-resistant Global Pathogen,” and “Functioning and Sustainable Country Public Health Emergency Response Operation Framework.” One of the conference’s key objectives was to provide a space for FETP residents, graduates, and public health professionals to showcase achievements. A total of 421 abstracts were submitted and after professional review, 34.9% (147/421) were accepted (111 for oral presentations and 36 for poster presentations) and published by Iproceeding. The conference met the primary objectives of showcasing the public health accomplishments and contributions of the EMR, encouraging the exchange of ideas and coordination among stakeholders, and engaging cross-sectoral workforce in producing recommendations for approaching regional and global health concerns. Moreover, the conference presented a unique opportunity for FETPs and other public health professionals from the Mediterranean region to present their significant scientific work and also facilitated networking among professionals. EMPHNET strives to continue to present similar exchange opportunities for public health professionals in the region.

## Background

The Eastern Mediterranean region (EMR) has complex and diverse health challenges especially because of the political instability, conflicts and forced displacement, frequent outbreaks of infectious diseases, increasing burden of noncommunicable disease, and poverty [1-3]. This demands public health professionals with a diverse set of skills including medical, behavioral, social, and environmental sciences. Continuing education for public health professionals is also essential to deliver high-quality public health services. However, public health professionals in the EMR have limited access to continuing education [4].

Workshops and conferences provide a critical infusion of public health capital. The Eastern Mediterranean Public Health Network (EMPHNET) holds workshops and conferences, and also supports the Field Epidemiology Training Programs (FETPs) in the EMR (ie, Afghanistan, Egypt, Iraq, Jordan, Morocco, Pakistan, Sudan, Tunisia, Saudi Arabia, and Yemen) [5].

EMPHNET organized and conducted the *Sixth EMPHNET Regional Conference* from March 26 to 29, 2018. It created the space for public health professionals to present accomplishments, share experiences, and provide networking opportunities. The target audiences were public health professionals and policy makers in EMR and regional and international organizations. This manuscript summarizes the key activities of the *Sixth EMPHNET Regional Conference* including workshops, roundtable discussions, oral and poster presentations, keynote speeches, and side meetings.

## The Conference Theme and Objectives

The central theme of the Sixth EMPHNET Regional Conference was addressing a variety of innovative approaches to public health and how these approaches can be adapted to the current EMR context. The conference’s main objectives were to create an opportunity for public health professionals from the region to present their accomplishments to a wide range of audience, discuss innovative approaches and solutions in counteracting public health challenges and problems in the EMR countries, and engage members of the public health community in a dialogue that focuses on reducing the impact of public health problems in the region.

The conference program offered a platform to showcase the scientifically grounded work of FETP residents and graduates, as well as other public health professionals. The abstracts presented at the conference covered a wide range of topics including outbreak investigations for respiratory diseases, outbreak investigations for vaccine preventable diseases, surveillance systems, zoonotic and vector-borne diseases, noncommunicable diseases (NCDs), mental health, child health, hepatitis, and HIV. Presenters included teams of public health experts and FETP graduates and residents from different countries across the EMR including Afghanistan, Algeria, Bahrain, Egypt, Ethiopia, Iraq, Iran, Jordan, Lebanon, Morocco, Oman, Pakistan, Palestine, Qatar, Saudi Arabia, Sudan, Tunisia, United Arab Emirates, Yemen, and other countries.

The preconference workshops were facilitated by experts from the respective fields to highlight public health concepts relevant to the EMR, whereas the roundtable sessions hosted a wide range of expert panelists. Roundtable sessions tackled issues related to the high burden of NCDs, communicable disease outbreaks, emerging and reemerging infections, weak surveillance systems, public health threats in mass gatherings, risks to biosecurity, and public health emergencies.

## Oral and Poster Presentations

One of the conference’s key objectives was to provide a space for FETP residents, graduates, and public health professionals to showcase achievements. A total of 421 abstracts were submitted and after being reviewed, 34.9% (147/421) were accepted (111 for oral presentations and 36 for poster presentations; Table 1) and published by Iproceeding [6]. Multimedia Appendix 1 shows the abstract book. Presentations were given by FETP residents and graduates, as well as other public health professionals from 13 countries (ie, Pakistan, Iraq, Egypt, Yemen, Morocco, Jordan, Saudi Arabia, Lebanon, Bangladesh, Palestine, Tunisia, Oman, and Sudan). Abstracts covered vaccine-preventable diseases, public health service evaluation, food- and waterborne diseases, vector-borne diseases, maternal, child, and reproductive health, respiratory diseases, zoonotic diseases, NCDs, and sexually transmitted infections.

**Table 1:** The distribution of abstracts and posters that were accepted and presented at the Sixth Eastern Mediterranean Public Health Network Regional Conference according to the abstract track and country of origin.

Distribution of abstracts and posters		Statistics (n)
**Track**		
	Vaccine preventable diseases	26
	Surveillance system evaluation	23
	Food- and waterborne diseases	17
	Vector-borne diseases	11
	Maternal, child, and reproductive health	11
	Respiratory diseases	15
	Zoonotic diseases	9
	Noncommunicable diseases	13
	Sexually transmitted infection	4
	Other	18
**Country**		
	Pakistan	52
	Iraq	16
	Egypt	13
	Yemen	13
	Morocco	16
	Jordan	9
	Afghanistan	4
	Sudan	1
	Oman	1
	Palestine	2
	Tunisia	2
	Bangladesh	3
	Lebanon	4
	Saudi Arabia	7
	Other	4

## Preconference Workshops

A total of 5 preconference workshops were organized: FETP Accreditation, Innovative Public Health Surveillance, Human and Animal Brucellosis, Rapid Response Teams, and Polio Transition and Routine Immunization.

### Field Epidemiology Training Program Accreditation Workshop

FETP accreditation by Training Programs in Epidemiology and Public Health Interventions Network (TEPHINET) [7] is a process aimed at improving and maintaining the quality of FETP training and graduates and their involvement and effectiveness in supporting the country’s public health priorities. The accreditation process references a set of minimum quality standards, provides momentum for continuous program quality improvement, and facilitates the identification of program needs.

The purpose of the workshop was to share with program directors and coordinators and FETP management staff both the process of becoming accredited and the ways that programs can be improved even if not seeking accreditation status. Specifically, the objectives of the preconference workshop were to review the purpose and benefits of FETP accreditation, describe in detail the eligibility requirements, indicators, and standards for FETP accreditation, and guide the program directors and coordinators to get their programs accredited.

The main recommendation of this workshop was to sustain the FETPs by institutionalization and integration of curricular at academia with the active contribution of countries to guarantee continuous funding and central program coordination and networking. Moreover, the FETP directors and coordinators and management staff in the EMR countries should work to get their FETPs accredited.

### Innovative Public Health Surveillance

The emerging field of new technologies and innovative tools has led to an evolution in public health surveillance and epidemic intelligence gathering [8]. Informal or “nontraditional” public health surveillance should enable quicker recognition of outbreaks. In response, TEPHINET—in joint venture with the Skoll Global Threats Fund, HealthMap, and ProMED-mail—developed EpiCore (a system that aims to complement existing surveillance methods and speed up the process of finding, reporting, and verifying public health events) [9]. The regional contributions made through the utilization of the EpiCore platform can create an impact on global health development that is significantly different than the one produced by traditional surveillance.

The objectives of this workshop were to discuss types, functions, challenges, and opportunities of traditional and nontraditional public health surveillance, recognize the importance and use of nontraditional public health surveillance in early detection and response to disease outbreak, and to demonstrate the functions and significance of EpiCore in improving public health surveillance. The workshop highlighted innovative public health surveillance tools to detect and respond to public health events in a more timely manner and raised awareness of EpiCore. All participants agreed on the following specific recommendations: Identify and prioritize various applications, platforms, and data collection devices through carefully listed selection criteria, develop a mobile app for EpiCore to allow easier access to the EpiCore platform and in turn easier and faster reporting, and share the concept of EpiCore with other relevant groups including schools of public health.

### Human and Animal Brucellosis

Brucellosis is known to be widespread in Middle Eastern and North African (MENA) countries, yet many of these countries do not have well-established surveillance systems or laboratory capacity to confirm brucellosis in humans or animals. Therefore, true disease burden and circulating species causing illness in humans and animals are not known.

This workshop aimed at improving brucellosis diagnosis and disease management and related practices. Specifically, the main objectives of the workshop were to explore the importance of strengthening the capacity of the public and veterinary health professionals in surveillance, diagnosis, and control of Brucella disease in animals and humans; emphasize EMPHNET experience in brucellosis surveillance, diagnosis, and control in Jordan and Iraq; present the laboratory diagnostic methods for brucellosis, advanced technology, and biotyping; discuss brucellosis control strategy in MENA region; and propose contextualized solutions for improving zoonotic diseases management and related best practices with focusing on awareness.

This workshop discussed the impacts of zoonotic diseases and explored collaborative opportunities to work on human and animal health related to brucellosis. It focused on detecting and preventing brucellosis in rural areas where borders are porous and neighboring countries should collaborate. The participants agreed on the following recommendations:

- Strengthen the surveillance and diagnostic capacities of the EMR countries including veterinary public health surveillance and response systems.
- Implement public awareness programs for the public, health workers, home industries, and farmers, by producing printing materials (eg, brochures, posters, and leaflets).
- Develop a national strategic action plan for the control of the major zoonotic diseases in the region. Evidence-based policy framework should be designed to strengthen information systems within each country in the EMR, and methods should be developed for estimating the total economic burden of zoonotic diseases in each country.
- Establish a regional mechanism that can serve the region and overcome the challenges and delayed responses witnessed in some countries such as Yemen.

### Rapid Response Teams

The EMR is one of the most vulnerable regions in the world. Furthermore, 10 EMR countries have suffered one or more disasters or emergencies just in the last couple of years, ranging from communicable disease outbreaks to complex emergencies. In 2017, more than 76 million people were directly or indirectly affected by political conflicts, environmental threats, or natural disasters [10]. Public health suffers from prolonged responses and the system itself is under immense pressure. EMPHNET and Global Health Development (GHD) has significant experience in the arena of rapid response including training, coordination, and deployment within and outside the region. EMPHNET has trained an important number of rapid response teams from 7 countries from 2012 onward.

This workshop was conducted to update knowledge and understanding of participants on rapid response, update rapid response procedures and protocols as well as structure and management, and expose participants to real-time information on disasters and complex emergencies at the regional level. Most of the participants agreed on the importance of working under a framework for emergency management. There was an agreement among participants on the importance and need for a regional rapid response team mechanism that can serve the region and overcome the challenges and delayed responses witnessed in some countries such as Yemen. The discussion also highlighted the importance of sustained rapid response team training programs at the national and subnational levels. The participants agreed on and recommended the following:

- Adequate public health emergency response operations plan for all the countries of the region. While developing and revising their public health emergency response plans, countries have to consider functionality, sustainability, and flexibility among the key characteristics of the end product and operate accordingly.
- Secure strong synergies and interactions between the country’s public health emergency operation center, rapid response teams, and the available health care workforce.
- FETPs should be considered (among other opportunities such as academia) as an important asset while building the country’s public health emergency response capacities as well as a mechanism to ensure sustainability of the country’s public health emergency response system.
- Countries have to cost their public health emergency response plans and fund them from their government’s basic budget lines to secure functionality and sustainability.

### Polio Transition and Routine Immunization

The progress toward the objectives of the eastern Mediterranean regional vaccine action plan from 2016 to 2020, except polio eradication, has been very slow and some of them are estimated to be off-track. The main reason is that a number of countries are facing acute or protracted emergencies.

The objectives of the workshop were to brief participants on the polio transition planning and postcertification strategy, share the country’s experiences with progress in developing transitional plans (Somalia and Sudan), discuss the remaining nonendemic countries’ plans to develop a transitional plan (Iraq, Syria, and Yemen), focus more on the country’s efforts, achievements, and constraints using polio assets to improve routine immunization (all countries), and discuss the best mechanisms to synergize and support priority countries in maintaining polio-free status.

This workshop offered an opportunity for senior immunization staff to interact with senior officers from the global polio eradication initiative partners (World Health Organization Eastern Mediterranean Regional Office, United Nations International Children’s Emergency Fund Middle East and North Africa [UNICEF MENA], Centers for Disease Control and Prevention [CDC] Atlanta, and Bill and Melinda Gates Foundation) and focus on the country’s major debated concerns. The workshop recommended the following:

- All countries in the EMR should have a polio transition plan, whether these countries were identified, either globally or regionally, as priority countries or not.
- Increasing expanded program on immunization (EPI) coverage is crucial for sustaining gains for polio eradication.
- Countries should document the experience of polio eradication, its best practices, and how it is being used for improving EPI and other health programs.
- In endemic and high priority countries, transition should be initiated in subnational levels, which have been polio-free for a long time or have relatively stronger health systems than others.
- Orientation on transition and provision of technical support if needed for the staff in nonpriority countries should be taken into consideration.

## Roundtable Discussions

The Conference hosted 6 roundtable discussions: Consolidation of the FETP Network, One Health to Achieve Global Health Security, Polio Eradication Efforts and Transition Planning for Measles Elimination, Mobile Data Collection and Other Innovative Tools to Enhance Decision Making, Confronting *Candida auris*: An Emerging Multidrug-resistant Global Pathogen, and Functioning and Sustainable Country Public Health Emergency Response Operation Framework (Textbox 1).

**Roundtable discussions hosted by the Sixth Eastern Mediterranean Public Health Network Regional Conference.**

1. Polio Eradication Efforts and Transition Planning for Measles Elimination:
  - Moderator: Dr Wael Hayejneh, Dean of Faculty of Medicine at Jordan University of Science and Technology
  - Presenter: Dr Dastagir Nazary, National Expanded Program on Immunization manager, Ministry of Health, Afghanistan
  - Panellists:
    - Mr Christopher Maher, Manager, Polio Eradication and Emergency Response, World Health Organization, Eastern Mediterranean Region (EMR)
    - Dr Jay Wenger, Manager, Polio Eradication Program, Bill and Melinda Gates Foundation
    - Dr Noha Farag, Deputy Team Lead for Science and focal point for research with Centers for Disease Control and Prevention’s (CDC) Eastern Mediterranean Team in the Polio Eradication Branch
    - Dr Sittana Ahmed, Polio C4D, UNICEF (United Nations International Children’s Emergency Fund) Regional office for MENA (Middle East and North Africa)
2. Toward the Consolidation of Field Epidemiology Training Program (FETP) Network in the EMR:
  - Moderator: Dr Mohamed Chahed, Director for the Center of Excellence of Applied Epidemiology, GHD/EMPHNET (Global Health Development/ Eastern Mediterranean Public Health Network)
  - Presenter: Dr Mohannad Al-Nsour, Executive Director, GHD/EMPHNET
  - Panellists:
    - Dr Mohamed Chahed, Director for the Center of Excellence of Applied Epidemiology, GHD/EMPHNET
    - Dr Kip Baggett, Chief of the US CDC’s Workforce and Institute Development Branch
    - Dr Dionisio Herrera, Director of the Training Programs in Epidemiology and Public Health Interventions Network
    - Dr Christophe Longuet, Executive Director of Connecting Organizations for Regional Disease Surveillance
    - Dr El Fatih El Semani, Professor at Ahfad University for Women
    - Dr Amrish Baidjoe, President of the European Programme for Intervention Epidemiology Training Alumni Network, currently an Operational Research Associate and Coordinator for outbreak analyses at Imperial College within the R Epidemics Consortium
    - Dana Shalabi, Communication Specialist, GHD/EMPHNET
3. Mobile Data Collection and Other Innovative Tools to Enhance Decision Making:
  - Moderators:
    - Dr Soloman Chandrasegarar, Immunization Specialist, UNICEF MENA Regional Office
    - Scott JN McNabb, Research Professor, Emory University, Rollins School of Public Health
    - Dr Mirwais Amiri, Operational Research Specialist, GHD/EMPHNET
  - Presenters:
    - Dr Moazzem Hossain, Chief of Health and Nutrition in UNICEF Iraq
    - Dr Farida Al Hosani, Manager of Communicable Diseases Department United Arab Emirates (UAE)
  - Panellists:
    - Dr Amr Youssef Ali Kotb, Ethics Committee and FETP Mentor, Egypt
    - Dr Scott Mc Nabb, Research Professor, Emory University, Rollins School of Public Health
4. Confronting *Candida auris*: An Emerging Multidrug-Resistant Global Pathogen:
  - Moderator:
    - Dr Tarek Al-Sanouri
  - Presenters:
    - Snigdha Vallabhaneni, CDC Mycotic Diseases Branch
    - Adnan Alatoom, Consultant physician, Head of Clinical Microbiology, Cleveland Clinic Abu Dhabi, UAE
    - Dr Rana Jawad Asghar, Resident Advisor of the Field Epidemiology and Laboratory Training Program (FELTP) in Pakistan
    - Dr Mirza Amir Baig, FELTP Pakistan
5. Roundtable on building Functioning and Sustainable Country Public Health Emergency Response Operation Framework:
  - Moderator:
    - Dr Ezzeddine Mohsni, Advisor Global Health Security, GHD/EMPHNET
  - Presenters:
    - Mahmoud Kaed Yousef, Crisis Management Unit Director, MoH Jordan
    - Dr Elmuez Eltayeb Elnaiem, General Director, Primary Health care General Directorate, Federal Ministry of Health, Sudan
    - Dr Abdel Wahed Al Serour, professor of Community Health, Faculty of Medicine and Health Sciences, Sana'a University, Yemen; and technical advisor of Yemen (FETP)
  - Panellists:
    - Sheik Dr Mohammed Bin Hamad Bin J. Al-Thani, Director of Public Health Department. Ministry of Public Health – Qatar; Associate Professor of Clinical Health care Policy and Research at Weill Cornell Medical College - Qatar
    - Dr Mark Salter - Consultant for Global Health
    - Tasha Stehling-Ariza, Epidemiologist with the Global Rapid Response Team at the US CDC
6. One Health to Achieve Global Health Security:
  - Moderator:
    - Scott JN McNabb, Research Professor, Emory University, Rollins School of Public Health
  - Presenters:
    - Professor Mahmudur Rahman, Former Director Institute of Epidemiology, Disease Control and Research and National Influenza Center, Bangladesh
    - Professor Ahmad Al-Majali, Professor of Infectious Diseases and Epidemiology at the Faculty of Veterinary Medicine, Jordan University for Science and Technology
    - Dr Zahida Fatima, Deputy Director/ Senior Scientific Officer, Pakistan Agricultural Research Council
    - Dr Ekhlas Qasem Hailat, Senior Disease Control Specialist/GHD/EMPHNET

### Consolidation of the Field Epidemiology Training Program Network

For the past 10 years, EMPHNET has been providing its support to existing FETPs and supporting the establishment of new FETPs in the region (6 new FETPs). The network is growing over time and the number of FETP graduates is now more than 500, which represents an invaluable core capacity for the countries’ health systems. EMPHNET has already undertaken some activities and initiatives to interconnect FETP graduates (via website, social media, and the ambassador initiative). EMPHNET would like to strengthen the consolidation of the network to value field experiences and support the public health systems.

The main objective of this roundtable was to discuss how to secure the emergence of field epidemiology in the EMR based on the lessons learned through the panelist experiences. The roundtable discussion focused on how to reinforce a productive and useful network through the region as a strategic orientation. The panel emphasized the willingness of EMPHNET to mountain and support an FETP network and the big need for the FETPs in the region to be strongly linked and supported by EMPHNET. The participants recommended to develop an interactive communication with graduates through “EpiShares” to consolidate the EMR FETP network.

### One Health to Achieve Global Health Security

*One Health* is based on the concept that the health of humans, animals, and environment is interconnected [11]. The most effective way to reduce human health threats is to interoperate all health disciplines. Protecting human health by combating outbreaks and preventing international spread remain a universal national priority. Addressing emerging and reemerging diseases (most being zoonotic) as a global health security issue will promote the prevention and rapid detection of novel biological threats, as well as assist in contextualized solutions for the response to these disease threats.

This roundtable aimed to present an overview of priority zoonotic global health security concerns and the importance of the interface of human, animal, and environmental public health sciences; describe regional zoonotic disease prevention, detection, and response using brucellosis as a model; and contextualize solutions to improve zoonotic disease management through best practices.

The panelists presented an overview of priority zoonotic global health security concerns and suggested different ways to strengthen collaboration between human and veterinary health and laboratory systems to prevent, detect, and respond to emerging and reemerging global health security threats and enhance information exchange. Facilitators agreed that zoonotic diseases represent significant challenges to human health and identified some gaps in both the veterinary and medical sectors, such as insufficient information to build a strategy; lack of training programs for veterinarians, technicians, clinicians, farmers, and laboratory workers; lack of diagnostic capacity that can ensure early detection of zoonotic pathogens; surveillance gaps for important diseases; limited capacity in field epidemiology and rapid field investigations; delays in reporting insufficient preparedness to control epidemics; and lack of monitoring and evaluation and weak coordination between entities such as ministries of agriculture, health, and environment.

The roundtable discussed the way forward to enable, enhance, and empower One Health and participants agreed on the below steps to enhance and empower the One Health approach:

- A national strategic action plan must be launched for the control of major zoonotic diseases.
- An evidence-based policy framework should be designed to strengthen information systems within each country.
- Methods should be developed for estimating the total economic burden of zoonotic diseases in each country.

- Strengthening of veterinary public health surveillance and response systems.
- Adoption of internet-based information technologies to improve disease reporting, facilitate emergency communications, and dissemination of information.
- Implementing effective preventive and therapeutic interventions.
- Implementing awareness programs in collaboration with media partners.
- Strengthening international collaboration and communication.
- Strengthening collaboration and coordination between different entities such as ministries of health, agriculture, and environment.
- Securing political commitment and adequate resources to address underlying socioeconomic factors.

### Polio Eradication Efforts and Transition Planning for Measles Elimination

Stopping polio transmission has been possible through improvement in implementing supplementary immunization activities (SIAs), supported by important innovative tools such as the precampaign readiness assessment, strong precampaign monitoring, and drastic postcampaign evaluation. Unlike polio SIAs, most priority countries have not implemented strong measles campaigns. Accordingly, countries continue to experience frequent measles outbreaks.

This roundtable aimed to review the package of assets, innovative tools, mechanisms, and procedures that allowed countries to make the change and achieve high-level performance in polio SIAs and to discuss some “low hanging fruit” solutions that countries and partners can support to allow smooth transition of polio assets in terms of measles immunization campaigns.

Participants agreed that polio SIAs benefit from a high government ownership and commitment translated into a strong accountability framework at all administrative levels of the country, an excellent coordination between implementing and supporting partners, and from a sustained high-quality acute flaccid paralysis surveillance. Moreover, they agreed that implementation of measles elimination programs suffers from low political commitment, lack of accountability, limited number of staff at national levels, and lack of dedicated staff in provinces. There was an agreement among the majority that the challenges of measles campaigns can be addressed through better campaign preparation, coordination, monitoring, and evaluation, as well as ownership and accountability, and that polio infrastructure should be used wherever and whenever possible (without jeopardizing the polio eradication efforts) to support required measles elimination vaccination campaigns that are much less frequent than polio SIAs but cost a lot of money.

The roundtable recommendations included the following:

- The country’s decision makers and stakeholders, as well as national, regional, and global partners, have to multiply efforts to support the implementation of high-performance measles vaccination campaigns.
- All priority countries that succeeded to stop polio transmission should start planning for and using, wherever and whenever relevant, polio assets, tools, and mechanisms to tackle public health priorities, with focus on routine immunization and measles elimination, while securing essential polio functions to sustain polio-free status.
- Routine immunization stakeholders and partners should not hide behind the structural differences between polio and measles to start transitioning polio SIAs assets and mechanisms to improve measles campaigns and surveillance performance.
- Social mobilization mechanisms and tools, such as barriers analysis, community empowerment, communication through front-line mothers, and others, used for polio, should be expanded to incorporate routine immunization as well as measles elimination.
- Where polio and EPI management teams are not integrated, countries and partners should encourage establishing of regular communication, information sharing, and coordination mechanisms to secure optimal utilization of resources, avoid duplication, and synergize efforts and messages for the benefits of both programs.

### Mobile Data Collection and Other Innovative Tools to Enhance Decision Making

Mobile data collection (MDC) and other innovative tools can effectively improve the decision-making process by providing on-demand reports and information in the shortest time and across all levels of the health system. Use of MDC is important in many areas such as minimizing cost of data collection and data entry, minimizing human errors, and eliminating quality issues related to data capturing, management, processing, and utilization [12,13]. MDC tools can improve current health systems by providing accurate results and information for public health practice while supporting the decision-making processes at various levels.

The main objectives of this roundtable were to share and highlight experiences from selected efforts in the region on how MDC or other innovative tools improved data quality and timeliness and discuss the most important challenges or lessons learned. The roundtable raised awareness of the importance and use of MDC in enhancing decision making and focused on programmatic implications and other innovative tools. One of the concerns was related to data security and sensitivity of the use of technology in conflict areas. Therefore, it is recommended to identify and prioritize them through carefully listed selection criteria, researching MDC platform options and considering data needs.

### Confronting *Candida auris*: An Emerging Multidrug-Resistant Global Pathogen

*Candida auris* is an emerging serious threat and causes severe illness in hospitalized patients [14]. *Candida auris* is an emerging global pathogen that poses the problem of multidrug resistance.

This roundtable aimed to raise awareness and increase knowledge about *Candida auris* and discuss concerns and issues related to it as an emerging global pathogen, and discuss strategies to prevent and control infections caused by it*.* All agreed that accurate identification is important to estimate the prevalence of this under-reported pathogen in different geographic areas. Some isolates are resistant to antifungal drugs which is a concern for getting the attention of the health care community. All agreed that implementation of stringent infection prevention and control for cases along with regular audits for compliance should be undertaken. Participants concluded that there was a need to stay alert and vigilant in monitoring the epidemiology of *Candida auris* globally through strengthening public health surveillance and laboratory capacities to detect and identify the organism.

### Functioning and Sustainable Country Public Health Emergency Response Operation Framework

Current turbulences and conflicts in the region affected tremendously at least half of the EMR countries, which conjugated with the increased number of threats from infectious diseases as well as other public health events [15]. Public health emergency response plan is a key component of overall emergency preparedness and response.

While developing the public health emergency response operation framework, countries should take measures that the final “product” is functional, sustainable, and flexible (dynamic and adaptable across time and circumstances). Unfortunately, this has not always been the case in the EMR, where the public health emergency response operation framework is fragmented and not built on available opportunities that can ensure sustainability with almost no links with the FETP, academia, and even national public health institutes.

This roundtable highlighted the importance for countries in the EMR to consider a comprehensive approach while developing their public health emergency response operation capacities. In addition, this roundtable helped to understand the importance for countries to consider strong links between their rapid response teams, public health emergency operation centers, and health care workforce development opportunities, particularly FETP graduates, while building their emergency response operations system.

Moreover, it highlighted the importance for countries to regularly check the functionality and performance of their public health emergency response operation framework, in particular after each outbreak or with periodic simulations and tabletop exercises, and undertake the required corrections and revisions. Panellists highlighted the fact that the prerequisites for a good preparedness include adequate knowledge about all potential hazards, perfect risk analysis, resource mapping and prioritization process, and develop capacities to prevent occurrence of public health events, reduce impact when they occur, and recover from their impact.

## Other Activities

### Launch of EpiShares

The conference launched *EpiShares*, a networking platform powered by GHD and EMPHNET. Developed with the aim to increase opportunities for the exchange of knowledge among public health professionals, the platform was developed by GHD and EMPHNET’s team to ensure a mechanism for sharing information and experience and to be a space that can attract public health experts, FETP residents, FETP graduates, or any community of practice. It comes with a host of features including a social media networking platform, the capacity for people with mutual interests to form groups, the capacity for members to start blogs, and a feature listing members within FETP directories or rosters of experts.

### Launch of Alumni Association

The conference also launched an alumni association aimed at bringing FETP alumni together from the region to share their rich experiences. The process of launching was discussed in a meeting well-participated by the interested FETP graduates. The agenda included a presentation by the President of the European Programme for Intervention Epidemiology Training (EPIET) Alumni Network, Dr Amrish Baidjoe, who gave an overview of the work done by EPIET and the challenges faced when initiating its launch. The presentation was deemed beneficial, as it allowed prospective FETP alumni members from the EMR to benefit from past experiences gained by their European peers. In his presentation, Dr Amrish stressed that the success of an alumni association was based on several key factors, the most important of which is having a passionate group of core committee members and a membership base willing to see this initiative succeed.

## Conclusions

The conference met the primary objectives of showcasing the public health accomplishments and contributions of the EMR, encouraging the exchange of ideas and coordination among stakeholders, and engaging cross-sectoral workforce in producing recommendations for approaching regional and global health concerns. Moreover, the conference presented a unique opportunity for FETPs and other public health professionals from the Mediterranean region to present their prominent work and network with other international professionals.

At the heart of action on global health security is a commitment to protecting the health of each community and bridging initiatives of all geographical and political regions. Hence, EMPHNET will continue to present similar exchange opportunities for public health professionals in the region. Conference participants and expert panellists provided many insights for moving onward.

## Appendix

Multimedia Appendix 1 The abstracts book.

## Abbreviations

CDC: Centers for Disease Control and Prevention
EMR: Eastern Mediterranean region
EMPHNET: Eastern Mediterranean Public Health Network
EPI: Expanded Program on Immunization
EPIET: European Programme for Intervention Epidemiology Training
FELTP: Field Epidemiology and Laboratory Training Program
FETP: Field Epidemiology Training Program
GHD: Global Health Development
MDC: mobile data collection
MENA: Middle Eastern and North African
NCDs: noncommunicable diseases
SIAs: supplementary immunization activities
TEPHINET: Training Programs in Epidemiology and Public Health Interventions Network
UNICEF: United Nations International Children’s Emergency Fund

## References

[1] Kennedy J (2016). Why have the majority of recent polio cases occurred in countries affected by Islamist militancy? A historical comparative analysis of the political determinants of polio in Nigeria, Somalia, Pakistan, Afghanistan and Syria. Med Confl Surviv.

[2] Rahim HF, Sibai A, Khader Y, Hwalla N, Fadhil I, Alsiyabi H, Mataria A, Mendis S, Mokdad AH, Husseini A (2014). Non-communicable diseases in the Arab world. Lancet.

[3] (2015). WHO Regional Office for the Eastern Mediterranean.

[4] White F (2013). The imperative of public health education: a global perspective. Med Princ Pract.

[5] The Eastern Mediterranean Public Health Network.

[6] Iproceedings: iproc.

[7] Training Programs in Epidemiology and Public Health Interventions Network.

[8] Groseclose SL, Buckeridge DL (2017). Public health surveillance systems: recent advances in their use and evaluation. Annu Rev Public Health.

[9] Haddad Z, Madoff L, Cohn E, Olsen J, Crawley A, Brownstein J, Smolinski M, Shao J, Pollack M, Herrera-Guibert D (2016). The EpiCore project: using innovative surveillance methods to verify outbreaks of emerging infectious diseases. Int J Infect Dis.

[10] ReliefWeb.

[11] One Health Initiative.

[12] Mukhi SN, Chester TL, Klaver-Kibria JD, Nowicki DL, Whitlock ML, Mahmud SM, Louie M, Lee BE (2011). Online Journal of Public Health Informatics.

[13] Mukhi S, Dhiravani K, Micholson B, Yan L, Hatchard J, Mubareka S, Bergeron C, Beattie T (2018). An innovative mobile data collection technology for public health in a field setting. Online J Public Health Inform.

[14] Tsay S, Welsh RM, Adams EH, Chow NA, Gade L, Berkow EL, Poirot E, Lutterloh E, Quinn M, Chaturvedi S, Kerins J, Black SR, Kemble SK, Barrett PM, Barton K, Shannon DJ, Bradley K, Lockhart SR, Litvintseva AP, Moulton-Meissner H, Shugart A, Kallen A, Vallabhaneni S, Chiller TM, Jackson BR (2017). Notes from the field: ongoing transmission of Candida auris in health care facilities-United States, June 2016-May 2017. MMWR Morb Mortal Wkly Rep.

[15] WHO Regional Office for the Eastern Mediterranean.

