# Supplementary material for: Innovative Approaches to Improve Public Health Practice in the Eastern Mediterranean Region: Findings From the Sixth Eastern Mediterranean Public Health Network Regional Conference
Source: JMIR Public Health Surveill. 2019 Mar 7;5(1):e11382. doi: 10.2196/11382 (PMC6427103; doi:10.2196/11382)
Supplement: Multimedia Appendix 1 [file publichealth_v5i1e11382_app1.pdf]

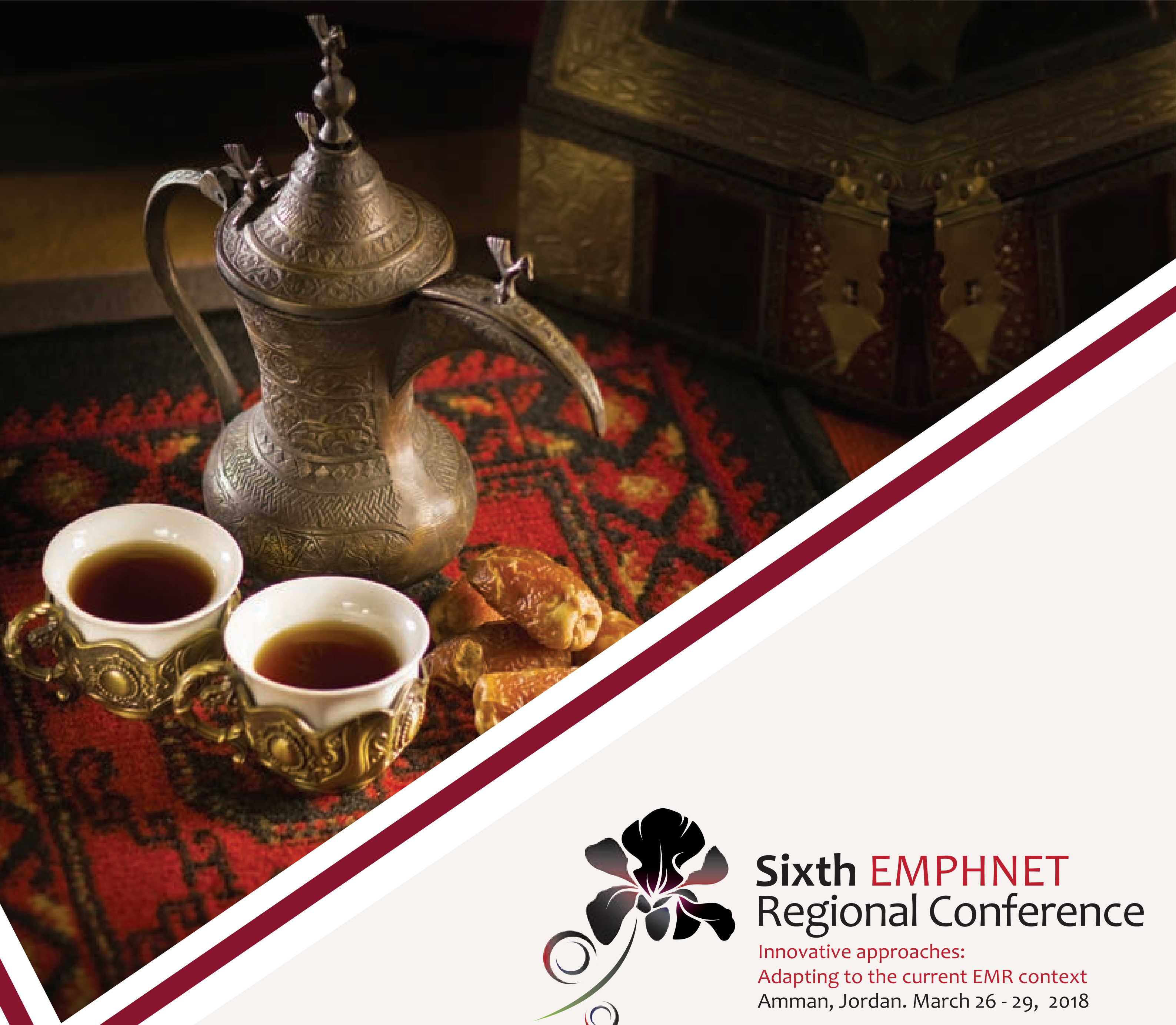

Sixth EMPHNET
Regional Conference

Innovative approaches:
Adapting to the current EMR context

Amman, Jordan. March 26 - 29, 2018

ABSTRACT BOOK

Global Health Development

EMPHNET
The Eastern Mediterranean Public Health Network

GHD and EMPHNET: working together for better health

Sixth EMPHNET Regional Conference

Innovative approaches:
Adapting to the current EMR context

Amman, Jordan. March 26 - 29, 2018

TABLE OF CONTENT

05 Introduction

09 Recognition Letters

20 Conference Committees

25 Pre-conference Workshops

28 Roundtable Discussions

31 Conference Program and Agenda

38 Oral Abstract Presentations

96 Poster Abstract Presentations

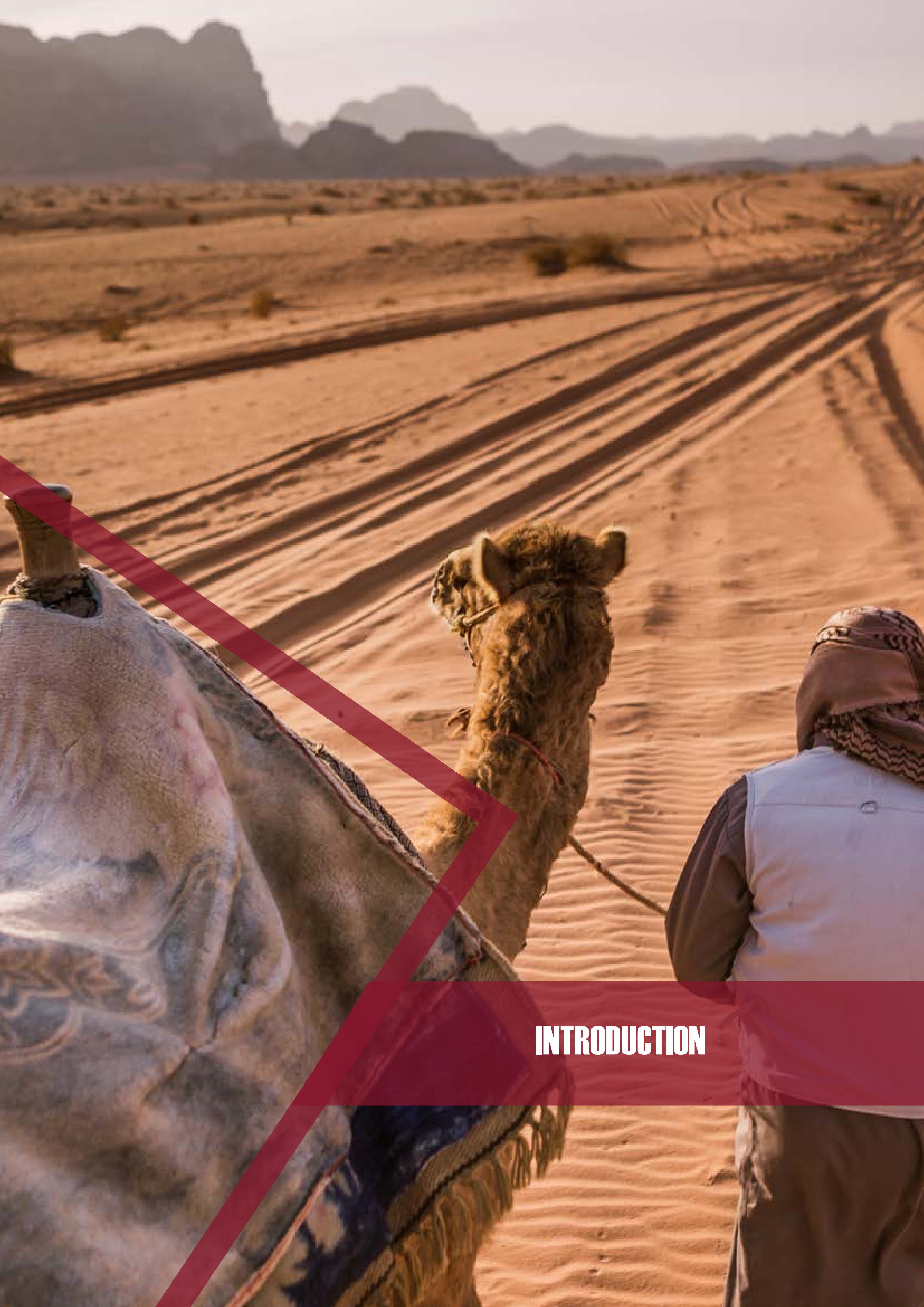

INTRODUCTION

Overview

The Eastern Mediterranean Public Health Network (EMPHNET) is launching its Sixth Regional Conference with the goal of addressing a variety of innovative approaches to public health. Conference sessions will focus on how these approaches can be adapted to the current Eastern Mediterranean Region (EMR) contest and challenges.

Taking place in Amman, Jordan, EMPHNET's Sixth Regional Conference presents field epidemiologists and public health professionals from across the region and around the world with opportunities to share their experiences in dealing with critical issues at the forefront of public health. The conference also presents an important platform for maximizing skills among field epidemiologists and public health professionals working to better serve their countries.

Held between March 26 and March 29, 2018 at the Landmark Hotel, the conference's three-day program will include sessions that will provide valuable opportunities for knowledge exchange regarding issues that jeopardize the health status of the region's populations. It will also offer a platform to showcase the scientifically grounded work of Field Epidemiology Training Program (FETP) residents and graduates, as well as other public health professionals who have chosen to share their achievements, research findings and field investigations with a wide participation base.

More specifically, the conference objectives are to;

- Create an opportunity for public health professionals from the region to present their accomplishments to a wide range of audience.
- Bring together public health officials, experts and scientists to examine and discuss innovative approaches attempted to strengthen public health in the EMR.
- Engage members of the public health community in a dialogue that focuses on reducing the impact of public health problems in the region.
- Encourage the exchange of innovative ideas, approaches and solutions in counteracting public health challenges and problems.

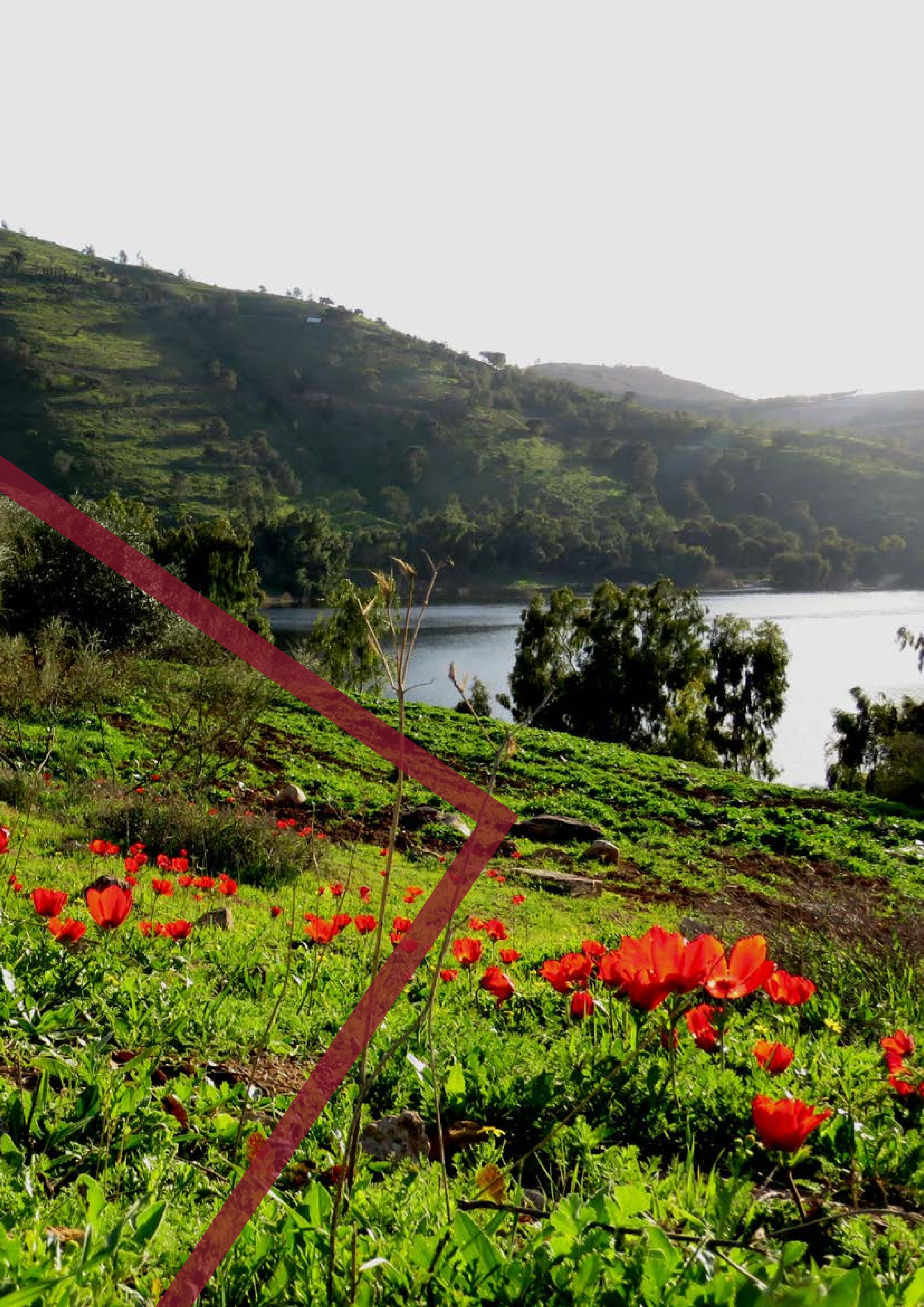

Theme

The central theme of the Sixth EMPHNET Regional Conference is Innovative Approaches: Adapting to the Current EMR Context. Within this theme, the conference sessions will provide a platform to present how innovative approaches can be adapted to improve public health practice in countries throughout the Eastern Mediterranean Region (EMR).

In line with EMPHNET's mandate to secure "better health for people in the EMR", the conference will focus on adopting innovative strategies in response to public health issues and problems challenging countries in the region. Such challenges include the high burden of NCDs, communicable disease outbreaks, emerging and re-emerging infections, weak surveillance systems, public health threats in mass gatherings, risks to biosecurity and public health emergencies. Hence, exploring new ideas and approaches to address public health challenges typical to the EMR is essential when considering the unprecedented political unrest and the enduring instability that left millions of people in the EMR at risk of disease exposure or suffering due to lack of health access.

The Conference Agenda

Prior to the official conference launch there will be a day of pre-conference workshops taking place on March 26, 2018. The conference will be officially launched on March 26, and its sessions will run until March 29, 2018. The conference agenda will include keynote official and scientific addresses, panel discussions, mini-symposiums, as well as oral and poster presentations highlighting the work of FETP residents, roundtable, graduates, and public health professionals, roundtable meetings, and a day of pre-conference workshops.

The pre-conference workshops will be facilitated by experts within their respective fields, while the roundtable sessions will host a wide range of expert panelists who will raise critical and controversial questions that trigger audience discussions and experience sharing. These sessions will tackle issues related to the high burden of NCDs, communicable disease outbreaks, emerging and re-emerging infections, weak surveillance systems, public health threats in mass gatherings, risks to biosecurity and public health emergencies, while pre-conference workshops will highlight public health concepts relevant to the EMR.

On a lighter note, the conference will also offer many social activities to encourage further networking amongst participants within a less formal setting.

The oral and poster presentations of abstracts reflect the work of FETP graduates and residents as well as public health professionals. The abstracts presented at the conference will cover a wide range of topics including cancer, child health, hepatitis and HIV, mental health, non-communicable diseases (NCDs), outbreak investigations for respiratory diseases, outbreak investigations for vaccine preventable diseases, surveillance systems, zoonotic and vector-borne diseases and other pressing topics.

Presenters include teams of public health experts and FETP graduates and residents from different countries across the EMR including Afghanistan, Bangladesh, Egypt, Iraq, Jordan, Lebanon, Morocco, Pakistan, Palestine, Saudi Arabia, Sudan, Tunisia, Yemen and other countries.

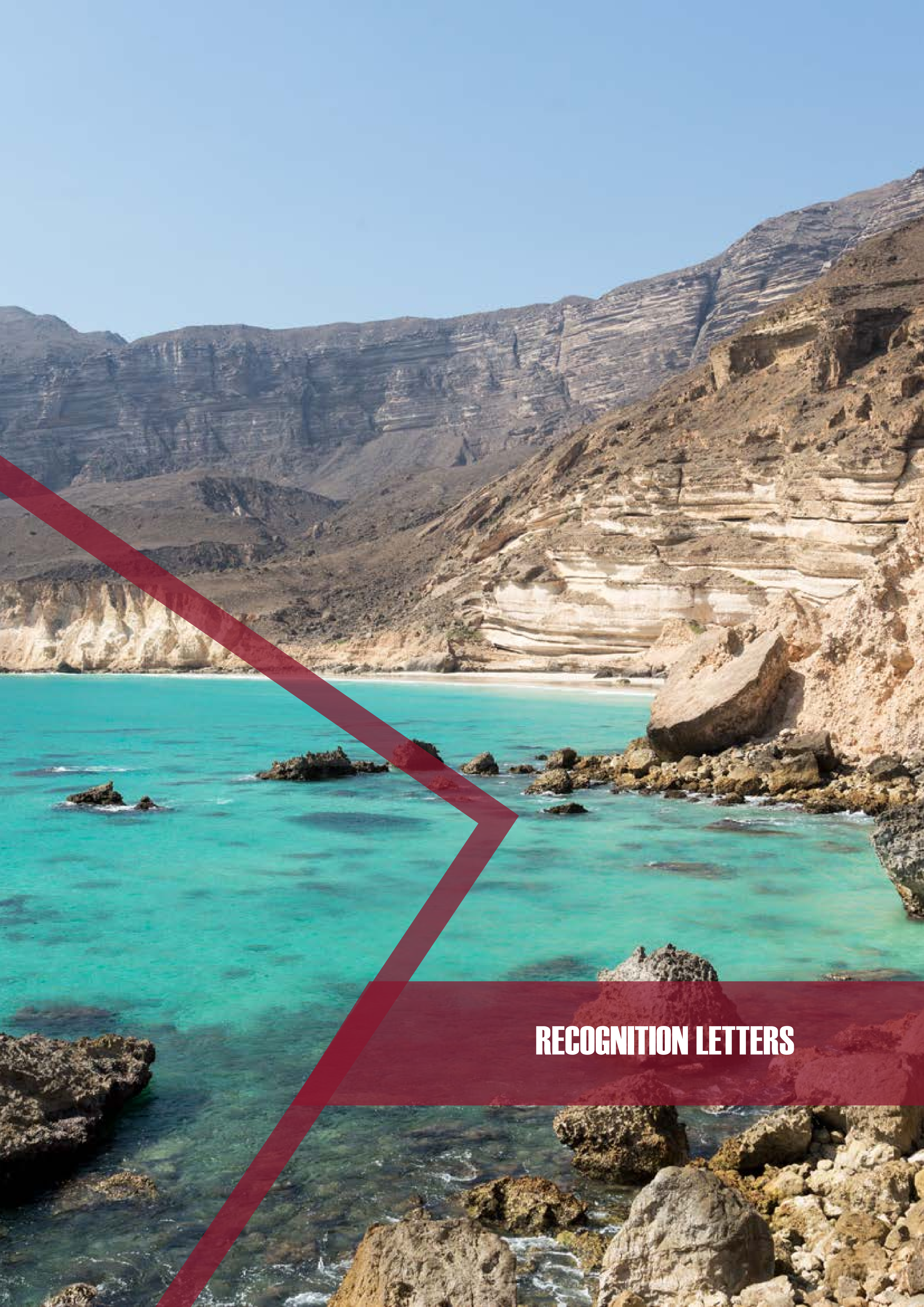

RECOGNITION LETTERS

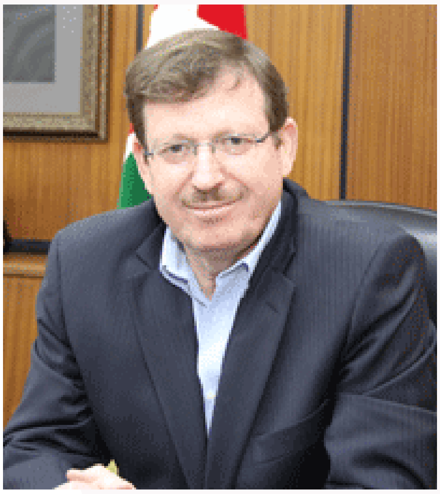

Dear Colleagues,

Once again, the Eastern Mediterranean Public Health Network (EMPHNET) brings together many of the public health community's elite in a forum that manifests success and showcases achievements being made in the field. For that, and within my capacity as both a public health practitioner and Jordan's Minister of Health, I would like to thank EMPHNET for this significant event , an event it has successfully been holding year-after-year. Furthermore, I would like to thank this network for choosing the Hashemite Kingdom of Jordan as the host for its Sixth Regional Conference..

The unrest characterizing our region is no secret. It renders the health sector overburdened with an urgent need for technical and non-technical support. However, we at all levels of practice, have proven to be up to the challenge, bracing ourselves with the knowledge and commitment we have to our mission for achieving better health in the EMR", a mission that unites us all.

This Sixth EMPHNET Regional Conference holds the theme "Innovative Approaches: Adapting to the Current EMR Context". To this effect, our deliberations will focus on the innovation employed in response to the current challenges facing this region. The conference sessions and roundtables offer an opportunity to share knowledge and expertise combined with unparalleled spaces for networking amongst public health professionals from the EMR.

I wish EMPHNET a successful conference and participants a pleasant stay in Jordan.

Prof. Dr. Mahmoud Al-Sheyyab
Minister of Health, Jordan

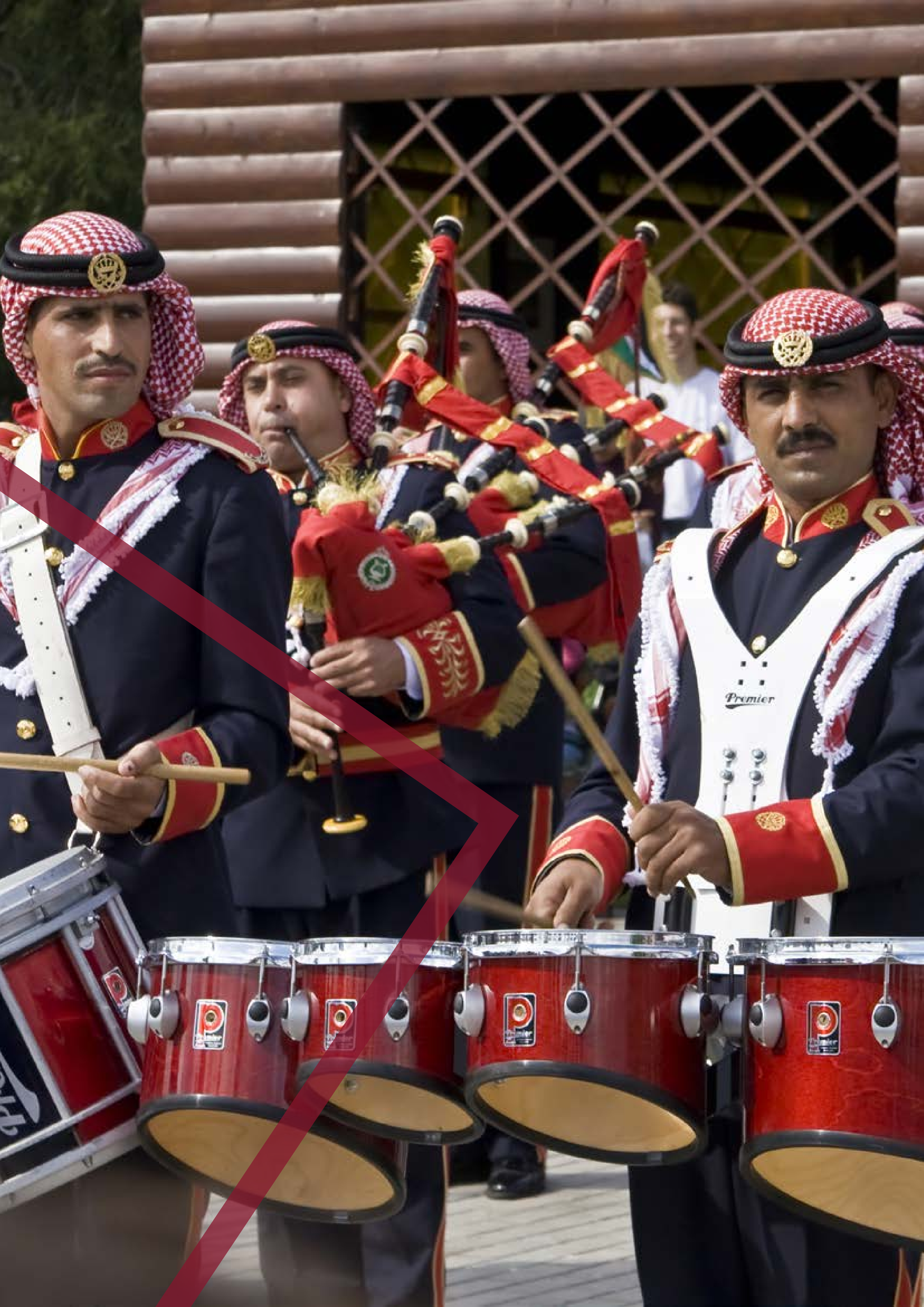
Premier

Excellencies, Distinguished Delegates, Ladies and Gentlemen

It gives me great pleasure to extend to you all a very warm welcome on behalf of the EMPHNET Board of Directors as representing the Field Epidemiology Training programs (FETPs) of the Eastern Mediterranean Region (EMR) to EMPHNET 6th Regional conference. This conference would not have possibly taken place in this beautiful city of Amman, without the tireless efforts of my colleagues in EMPHNET, FETP country directors, the Jordan Ministry of Health and the Jordan Public Health Association. I extend my sincere thanks and appreciation to all.

I believe, this conference will provide us with an opportune time to renew our commitments towards improving the health status of our people with efforts joined with delegates from member countries across our region.

Population health status in the EMR is partly influenced by events and circumstances that challenge the region's national health system. The high burden of non-communicable diseases, the spread of communicable diseases, the presence of emerging and re-emerging infections and the growing threats to biosecurity are issues that challenge public health in the EMR. Furthermore, the long lasting presence of political conflict, humanitarian crises, natural disasters and emergencies interfere with the provision and quality of healthcare services in most countries of our region, thus compromising population health outcomes and increasing the risk of disease outbreaks.

In time of increasing public health needs and diminishing resources, the ability to respond tp public health challenges in the EMR is essential. It requires enhanced public health capacity, which must be supported through collaboration and innovation among countries. As per the theme of this year of the conference which is "Innovative Approaches towards Adapting to the current Eastern Mediterranean Region Context" this conference is an important platform for presenting how innovative approaches can be adapted to improve public health practices in countries throughout the region

No matter how much we can do by ourselves on the national level, whether it be research or development, it is never enough. In a spirit of true cooperation, we must join in an action-oriented effort to attack and solve the problems that beset health development in our region and beyond. If on the national level we can, and must, pursue health development as a multi-dimensional concept, encompassing the economic, social, institutional and physical elements of development, in a wider sense, it would be relatively easy to effect the necessary adjustments for a truly effective cooperation on the regional basis. This is fully consonant with our official position taken and the full support of the concept of international health development adjustment at this conference.

At the end, I'd like to reiterate that, we feel certain that public health service and practice in the EMR will benefit greatly from this conference as we unite to share both accomplishments and challenges so that together we move towards excellence in public health to better serve our communities and people.

Wish you all a fruitful conference and enjoy your stay in the beautiful city of Amman

Associate Prof. Dr. Bashir Noormal
Chair of the Board of Directors of FETP of the
Eastern Mediterranean Public Health Network
and Director General Afghanistan National Public Health Institute

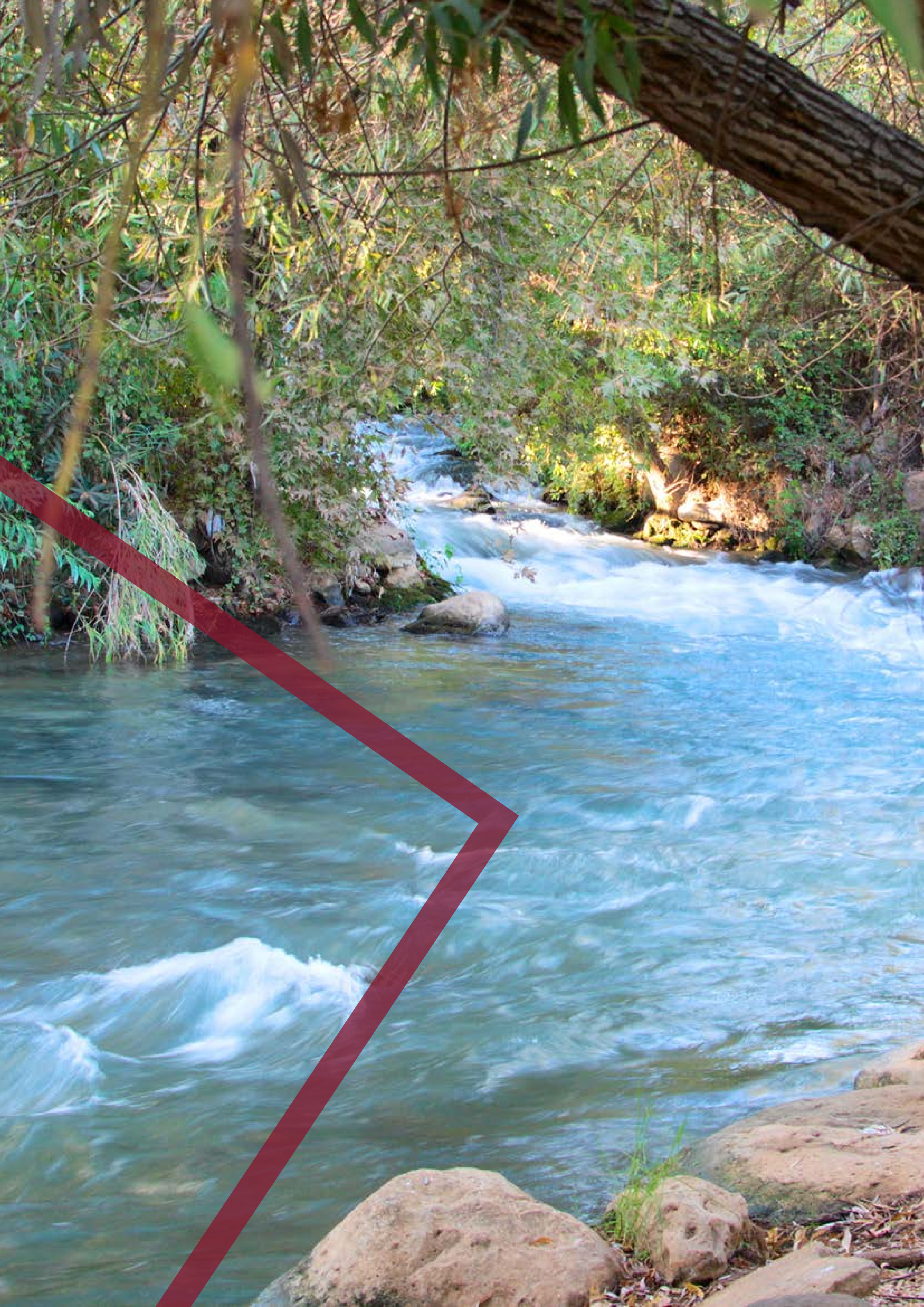

Dear Colleagues,

Congratulations on the sixth EMPHNET/TEPHINET conference.

On behalf of TEPHINET, it is an honor for me to recognize EMPHNET on the successful organization of the sixth regional conference and to acknowledge your ten years of excellent work in supporting field epidemiology training and public health system strengthening activities across the Eastern Mediterranean region.

From EMPHNET's beginnings, our two networks have worked closely together, and we are expecting to continue and increase our collaboration in the future. TEPHINET, as a global network of field epidemiology training programs (FETPs), has been an enthusiastic supporter of the development of EMPHNET and is proud to have its representation on our global Advisory Board as well as on the TEPHINET Accreditation Working Group, the latter of which develops the standards for FETP training quality worldwide. The Eastern Mediterranean region faces unique challenges to its public health systems, and EMPHNET has proven to be a strong resource for FETPs working to boost their disease surveillance and response capacity to tackle these challenges and increase health security in the affected countries. Our colleagues in the region, under the leadership of their board and executive office, have been demonstrating the value of the regional network in different aspects including coordination, support, advocacy for the programs, and a platform to share experiences.

The strong collaboration of the FETPs over the past decade has resulted in training quality improvement and more opportunities for field epidemiologists studying and working in the region, and at the same time, the number and quality of the programs has increased. I am impressed to see that EMPHNET has received more than 400 abstracts for this conference and that more than 140 will be presented. This type of growth is a testament to the increased quality and capacity of the regional programs.

It is my hope that the partnership between our networks will remain strong and take new, exciting directions that we can now only imagine.

On behalf of the TEPHINET Secretariat and our global network of member FETPs, I would like to congratulate all of the presenting authors on their excellent work and wish them much success in their careers. I would also like to congratulate the executive office of EMPHNET for its hard work in this endeavor. I hope to see you at a future TEPHINET conference.

Sincerely,

Dionisio José Herrera Guibert, MD, FMS, MAE, PhD
Director, TEPHINET

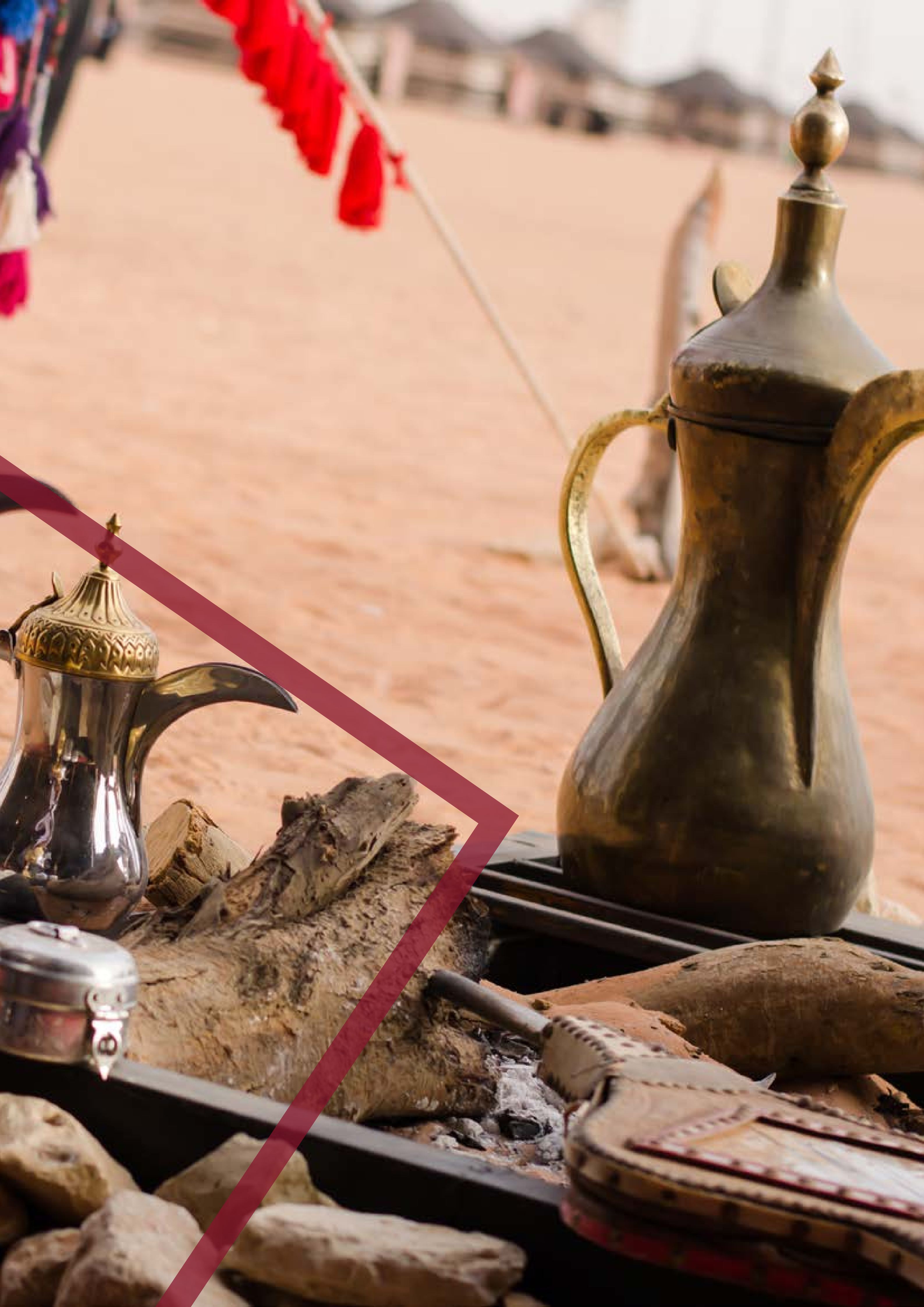

Dear Colleagues,

It gives me great pleasure to extend to you a very warm welcome on behalf of EMPHNET and to express how grateful we are to Her Royal Highness Princess Muna Al-Hussein for her esteemed patronage of our Sixth EMPHNET Regional Conference and to the Jordan Ministry of Health for hosting this event here in our beloved capital Amman.

For this conference we received 421 abstracts, out of which 111 were accepted for oral presentation and 36 for poster presentation. Accepted abstracts were developed by FETP residents and graduates and public health professionals from Pakistan, Iraq, Egypt, Yemen, Morocco, Jordan, Saudi Arabia, Lebanon, Afghanistan, Bangladesh, Palestine, Tunisia, Oman and other countries beyond the EMR.

The agenda for the Conference, which is being held under the theme "Innovative Approaches: Adapting to the Current Context in the EMR", covers a wide range of topics. All these topics are related to the development of innovative strategies . They highlight how such strategies may be adapted to respond to public health issues that pose challenges for countries in the region.

Sessions will focus on the high burden of non-communicable diseases, communicable disease outbreaks, emerging and re-emerging infections, weak surveillance systems, public health threats in mass gatherings, risks to biosecurity and public health emergencies. Hence, exploring new ideas and approaches to address public health challenges specific to our region is essential. We are aware that regardless of what we do on the national level, be it research or response, more can be done through collaboration. In a spirit of true cooperation, we in this region are proud of nurturing all past and present civilizations. Therefore, we must join in an action-oriented effort to respond to those challenges that delay development; especially when considering the unprecedented political unrest and instability that left millions of our people at risk of disease exposure or suffering.

I would like to emphasize that this three-day event presents a great opportunity for you to reach a large number of participants from the public health community, and to exchange information and ideas, draw attention to new methodologies, and receive recognition.

In conclusion, you carry with you best wishes from EMPHNET for both a productive and successful Conference, and a pleasant stay amongst us.

Dr. Mohannad Al Nsour
Executive Director, EMPHNET

A Note of Sincere Thanks and Appreciation

Dear Moderators,

We would sincerely like to thank you for taking on a moderating role during our Sixth EMPHNET Regional Conference, and thus contributing to the conference's success.

All your hard work has paid off, and as a result, we can honestly say that our sessions will be efficiently run. The end result is a fruitful conference experience for all participants, and this is on account of the time and effort you have spent with your contribution.

Yours Sincerely,

The Scientific Committee of the Conference

Moderator Name	Title/Organization	Country
Dr. Aamer Ikram	Executive Director of Pakistan NIH and Pakistan FELTP Director	Pakistan
Dr. Abdalla Sied Ahmad Osman	Director of the Public Health Institute (PHI)	Sudan
Dr. Abdelmounim Belalia	Director of National Public Health School	Morocco
Dr. Abid Saeed	Federal Faculty-Pakistan Field Epidemiology Training and Laboratory Program	Pakistan
Dr. Adel Salman Al Sayyad	Chief of Disease Control Section, Ministry of Health	Bahrain
Dr. Ahmad Abu Slaih	Chief of Community Specialty	Jordan
Dr. Ahmed Darwish	Senior Technical Advisor	Egypt
Dr. Ahmed Rguig	Head of Epidemiology Surveillance, Directorate of Epidemiology and Disease Control	Morocco
Dr. Amrish Baidjoe	President of EPIET Alumni Association	UK
Dr. Ayoub Al-Sayaydeh	Director of Primary health care administration	Jordan
Dr. Bashir Noormal	General Director of Afghanistan National Public Health Institute	Afghanistan
Dr. Chakib Boukhalfa	Professor, The National Institute of Health Administration (INAS)	Morocco
Dr. Christophe Longuet	Connecting Organizations for Regional Disease Surveillance (CORDS)	France
Dr. Derek Ehrhardt	Global Immunization Division - GID - CDC	USA
Dr. Elfatih Elsammani	Senior Instructor, FETP	Sudan
Dr. Elshaikh Mohammed Al Thani	Director of Public Health Department at Ministry of Public Health	Qatar
Dr. Farida Al Hosani	Manager of Communicable Diseases	UAE
Dr. Ghulam Dastagir Nazary	EPI Manager, Ministry of Public Health	Afghanistan
Dr. Hayat Khogali	Supervisor, Expanded Program on Immunization	Qatar
Dr. Hayel Obeidat	General Director Of JFDA	Jordan
Dr. Husien Abu Zaid	Director of Preventive Medicine and Public Health- Royal Medical Services	Jordan
Dr. Isameddine Abdalla	First Under Secretary, Federal Ministry of Health (FMOH)	Sudan
Dr. Jawad Mofleh	Technical Advisor	Germany
Dr. Khalid Temsimani	President, The Moroccan Biosafety Association (MOMSA)	Morocco
Dr. Loulou Kobesssi	Scientist / Adolescents and at-Risk Populations Unit, WHO	Geneva

Moderator Name	Title/Organization	Country
Dr. Madi Tawfiq Al-Jaghbir	Family and Community Medicine Department- Jordan University	Jordan
Dr. Majed Asad	Director, Non-communicable Disease Directorate- Jordan MOH	Jordan
Dr. Mahmoud Abdallat	President of Public Health Association	Jordan
Dr. Mahmud Rahman	Senior Technical Advisor	Bangladesh
Dr. Malek Al-O'ri	Director, Maternal and Child Health Directorate, Jordan MoH	Jordan
Dr. Mazen Malkawi	Regional Centre for Environmental Health Action (CEHA)	WHO-EMRO
Dr. Mir Islaam Saeed	Director of GCMU-Grant and Contract Management Unit	Afghanistan
Dr. Mirza Amir Baig	Federal Faculty FELTP	Pakistan
Dr. Moazzem Hossain	Chief of Health and Nutrition, UNICEF	Iraq
Dr. Mohammad Abdallat	Director Communicable Directorate - MoH	Jordan
Dr. Mohammad Tarawneh	General Secretary High Health Council - MoH	Jordan
Dr. Monic Shaya	Head of Epidemiology and Population Department	Lebanon
Dr. Nabil Hailat	Jordan University of Science and Technology	Jordan
Dr. Nada Ahmed Jaafar	Director of Mother and Child health FMOH, PHEP SPO Sudan Technical Advisor	Sudan
Dr. Nada Ghosn	Head of Epidemiology Surveillance Program (Esumoh) Ministry of Public Health	Lebanon
Dr. Nathir Obeidat	Dean of Faculty of Medicine Jordan University	Jordan
Dr. Naveed Kamran Baloch	Secretary, Ministry of National Health Services, Regulations and Coordination	Pakistan
Dr. Noha Faraj	Global Immunization Division - GID	USA
Dr. Pierre Nabeth	WHO Health Emergencies Programme	WHO-EMRO
Dr. Raeda Al Quttob	Family and Community Medicine Department- Jordan University	Jordan
Dr. Saad Kharabsheh	Former Minister of Health	Jordan
Dr. Sahar Sami	Field Epidemiology Training Program Coordinator	Egypt
Dr. Said Jaradat	Princess Haya Center for Biotechnology-Center Director - JUST	Jordan
Dr. Saif Alaberi	Director General of Communicable Diseases and Control, Ministry of Health Oman	Oman
Dr. Sami Al-Mudarra	Director of Saudi Arabia FETP	KSA
Dr. Samir Reffaey	Technical Advisor, GHD/EMPHNET	Egypt
Dr. Scott McNabb	Research Professor | Emory University, Rollins School of Public Health	USA
Dr. Sultan Abdallah	Head Surveillance Department - MoH	Jordan
Dr. Wail A. Hayajneh	Dean of Faculty of medicine JUST	Jordan
Dr. Yousef Zafar	Chairman of Pakistan Agricultural Research Council Headquarters (PARC)	Pakistan
Dr. Walid Maani	Former Minister of Health	Jordan

A Note of Sincere Thanks and Appreciation

Dear Abstract Review Committee Members,

The Sixth EMPHNET Regional Conference Organizing Committee would like to express its gratitude for your tireless efforts during the review process of the abstracts submitted for our Sixth EMPHNET Regional Conference.

All the hours of your hard work have paid off and, as a result, we are proud to have all submitted 420 abstracts carefully reviewed on a tight schedule. This allowed for the successful and timely accomplishment of this tremendous task. Therefore, you are the reason we should be proud of making this effort happen on time.

We, once again, would like to express our sincerest gratitude towards you for such a commendable job.

Yours Sincerely,

The Organizing Committee of the Conference

Reviewer Name	Organization
Dr. Abdelwahed Al-Serouri	Technical Advisor, FETP Yemen
Dr. Ali Alassabri	Technical Advisor, FETP Yemen
Dr. Faris Lami	Technical Advisor, FETP Iraq
Dr. Haitham Bashir	GHD/EMPHNET
Dr. Mohamed Chahed	GHD/EMPHNET
Dr. Yousef Khader	GHD/EMPHNET

Jordan FETP Program - Cohorts 11 and 12

Feras Bsam Mohammad
Ghaith Sami Owies
Majid Mohammad El-Hawasheh
Meqdad Zuhir Aburomman
Mohammad Abdulrazaq Alfauri
Mohammad Yasin Alqadan
Mohammad Ghazi Hattab
Mouatasem Mousa Hussainat
Naser Hamad Alrawashdeh
Qusai Sabri Adawi
Raad Naif Almalouf
Sami Mohammad Al-Shoubaki
Suhaib Najib Abufailat
Ibrahim Muslih Al-Habarneh
Ashraf Yousef Malkawi
Mohammad Whid Aladhash
Mahmoud Husain Al Habashna
Ashraf Gamil Aqel
Basel Khalid Abu Hdeab
Rakan Ahmad Abu Roman
Sa'ed Adel Assaf
Salam Farhan Khreisat
Adel Salem Al-Rawahneh
Abdullah Mazin Matouq
Ikrimah Fawaz Abu Salim
Mohammad Nimer Alhwarat
Mahmoud Ali Yacoub
Nancy Mohammed Abdullrahim
Fatima Mostafa Zerriouh
Mohammad Mahmoud Alrawahnih

Scientific Committee

Dr. Abdelwahed Al-Serouri

Dr. Ali Al Assabri

Dr. Ali Nimer Odatallah

Dr. Asmae Khattabi

Dr. Biagio Pedalino

Dr. Ekhlas Hailat

Dr. Faris Lami

Dr. Fatih Malik

Dr. Haitham Bashir

Ms. Ilham Abu Khader

Dr. Jawad Rana Asghar

Dr. Mohamed Chahed

Dr. Mirwais Amiri

Dr. Najwa Jarour

Dr. Nissaf Bouafifi

Dr. Robert Fontaine

Dr. Tareq Sanouri

Dr. Yousef Khader

Organizing Committee

Dr. Ibrahim Iblan

Dr. Sahar Sami

Amjad Hiary

Banan Obiedat

Dana Shalabi

Diana Abu Baker

Emad Saif

Haitham Nazzal

Haneen Adwan

Hiba Qtaishat

Katy Carlson

Khaled Al Thaher

Lina Al-Hadid

Mohammad Asad

Mohammad Zraiqi

Murtaja Awad

Rana Manaseer

Rawan Araj

Salam Marzouq

Executive Committee

Dr. Bashir Noormal

Dr. Adel Belbaisi

Dr. Ayoub Al-Sayaydeh

Dr. Dionisio Herrera

Dr. Ezzeddine Mohsni

Dr. Kip Bagget

Dr. Mohannad Al-Nsour

Ms. Samar Abdelnour

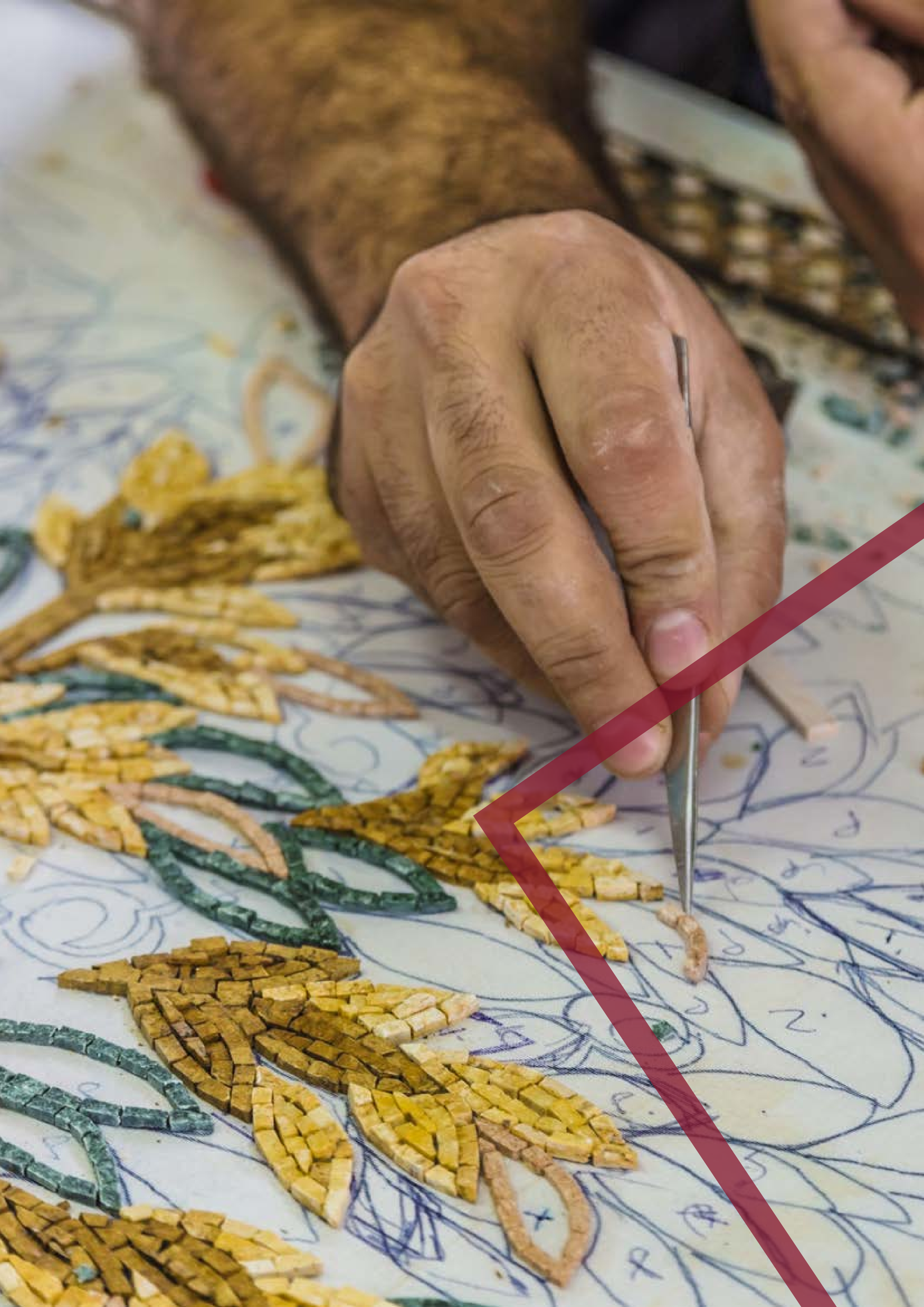

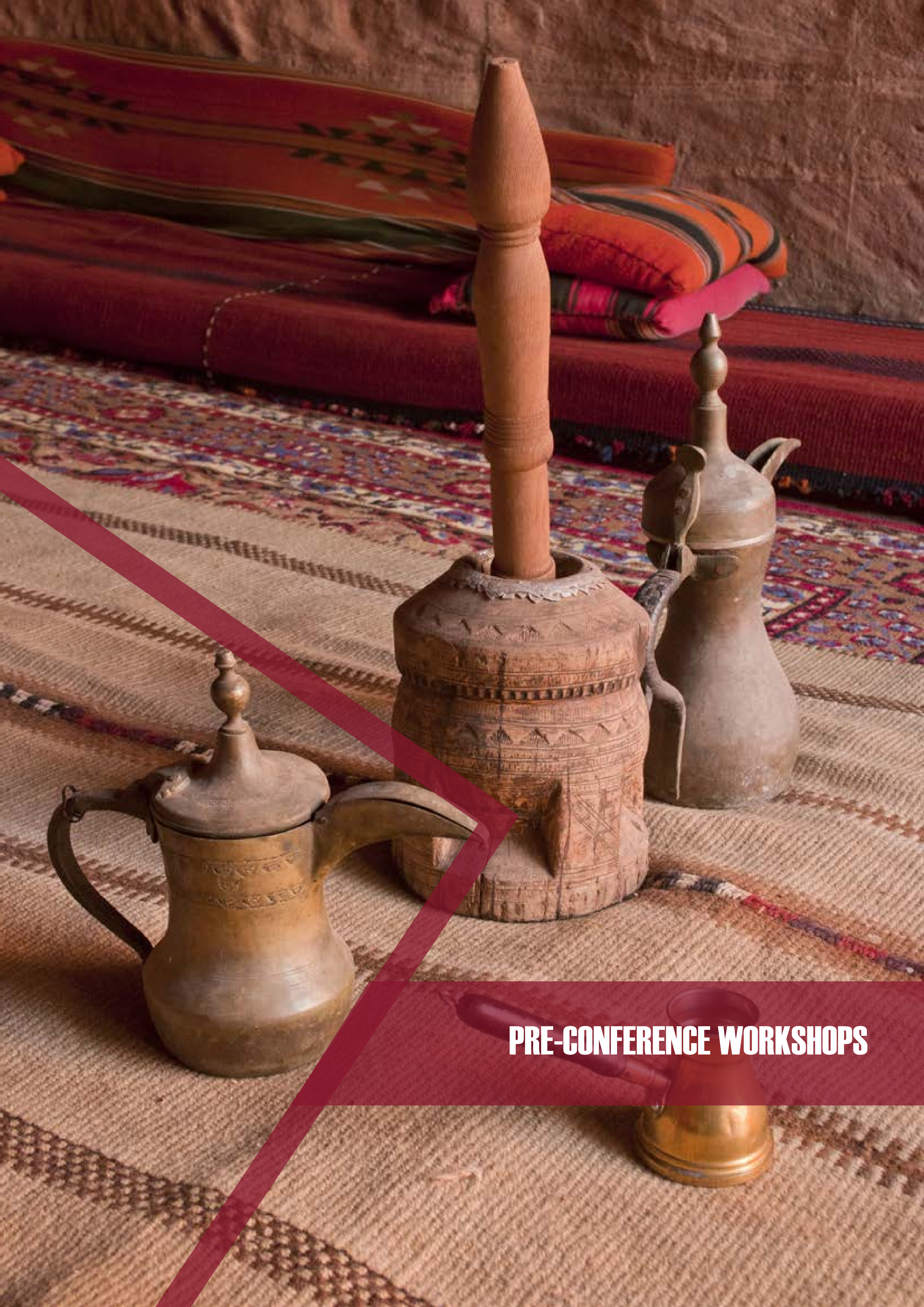

PRE-CONFERENCE WORKSHOPS

The Sixth EMPHNET Regional Conference – Amman, Jordan
Pre-Conference Workshops: Monday, March 26

Before the commencement of the Sixth EMPHNET Regional Conference, several workshops will be facilitated by experts from regional and international entities on FETP Accreditation, Polio Transition and Country Routine Immunization, Innovative Disease Surveillance, and Rapid Response Teams.

1. **FETP Accreditation**
   Field Epidemiology Training Program (FETP) accreditation is a process aimed at improving and maintaining the skills of FETP trainees and graduates, and their involvement and effectiveness in supporting country public health priorities. The accreditation process references a set of minimum quality standards, provides momentum for continuous program quality improvement, and facilitates the identification of program needs. The purpose of this workshop is to share with program directors/coordinators and FETP management staff both the process of becoming accredited, and the ways that programs can be improved even if not seeking accreditation status.

2. **Country Polio Transitional Plans and Routine Immunization**
   The goal of this pre-conference workshop is to actively interact with conference participants on the current challenges facing Routine Immunization (RI) in the region. The workshop will focus on countries facing emergency situations and will explore opportunities to scale up performance of RI. This one-day workshop will tackle regional progress and challenges towards achieving the goals of Regional Vaccine Action Plan (RVAP); immunization in countries of security compromised situations; Polio- circulating vaccine-derived poliovirus (cVDPV) in Syria – lessons learned; Measles outbreaks in the region and progress towards elimination; Reemerging of Diphtheria in Yemen; and Afghanistan innovative strategies in data management.

3. **Innovative Disease Surveillance**
   The emerging field of new technologies and innovative tools has led to an evolution in public health surveillance and epidemic intelligence gathering. This workshop is designed to highlight innovative surveillance tools that contribute to global efforts made to detect and respond to outbreaks and public health events in a timelier manner. Workshop participants will learn about innovative disease surveillance methods and how to use the EpiCore platform to verify outbreaks. As nontraditional surveillance tools become more widely used and relied upon, it is imperative that platforms such as EpiCore have the full participation of public health professionals to demonstrate the validity of informal surveillance data and to harness the full potential of innovative surveillance systems.

4. **Rapid Response Teams**
   Several countries in the Eastern Mediterranean Region (EMR) is suffering from disasters and emergencies that are sometimes worsened by delayed response to disease outbreaks. The purpose of this pre-conference workshop is to refresh and update the knowledge of participants on rapid response to disease outbreaks and emergency management, present available rapid response procedures and protocols, and expose participants to timely information on disasters and complex emergencies in the region. Workshop participants will learn about major emergencies in EMR, principles of rapid response and emergency management, and the role of rapid response teams during disease outbreaks.

5. **Human and Animal Brucellosis**
   This pre-conference workshop is organized to discuss the impact of zoonotic diseases on global health security, and explore collaborative opportunities for professionals working on human and animal health related to brucellosis surveillance, diagnosis and control in the region. The pre- conference workshop will contribute to a larger One Health strategy to zoonotic diseases in the region and will allow countries to share relevant best practices with others. Focus will be on approaches for detecting and preventing brucellosis in rural areas of the region where borders are more porous and neighboring countries need to address the threat together.

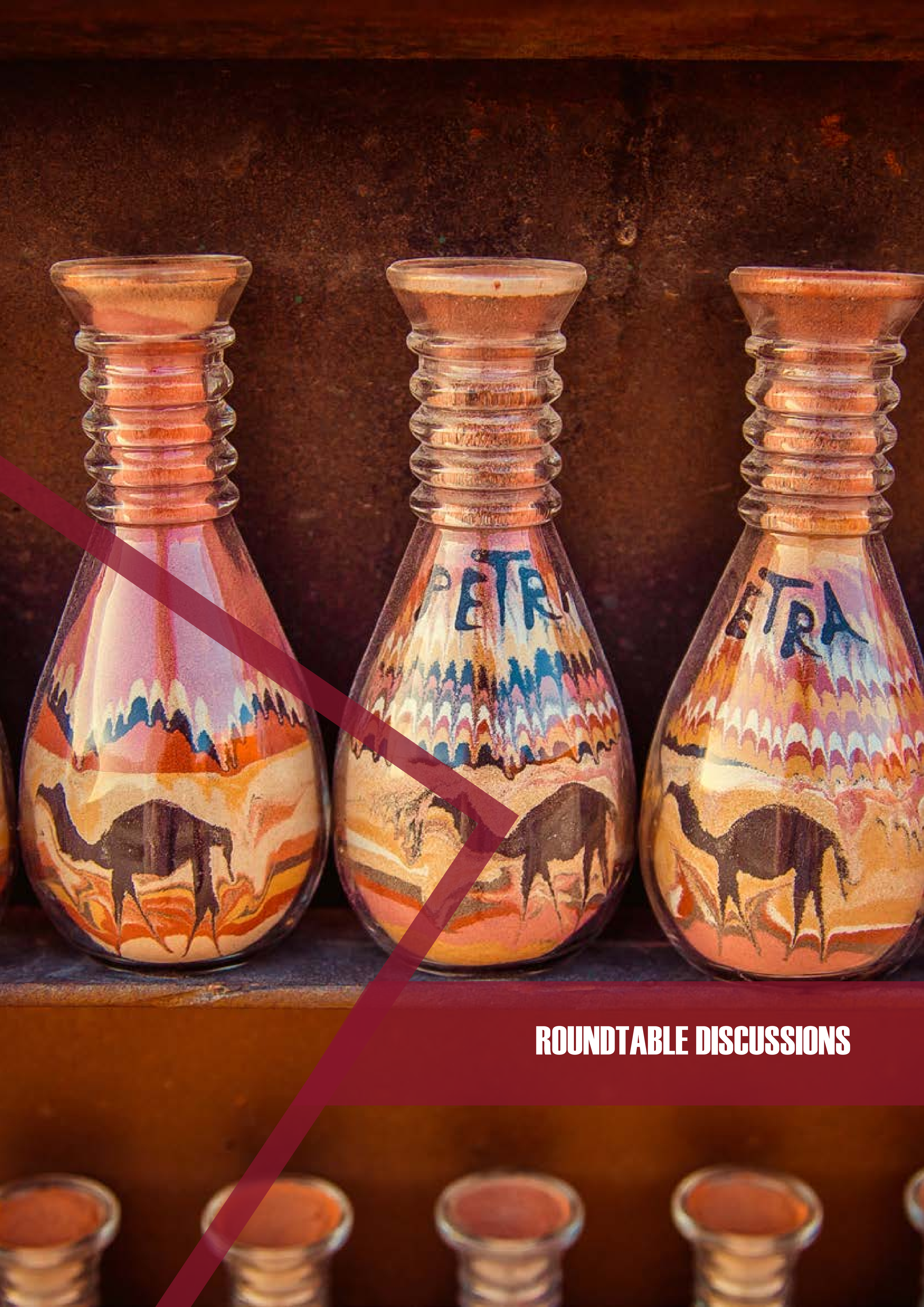

ROUNDTABLE DISCUSSIONS

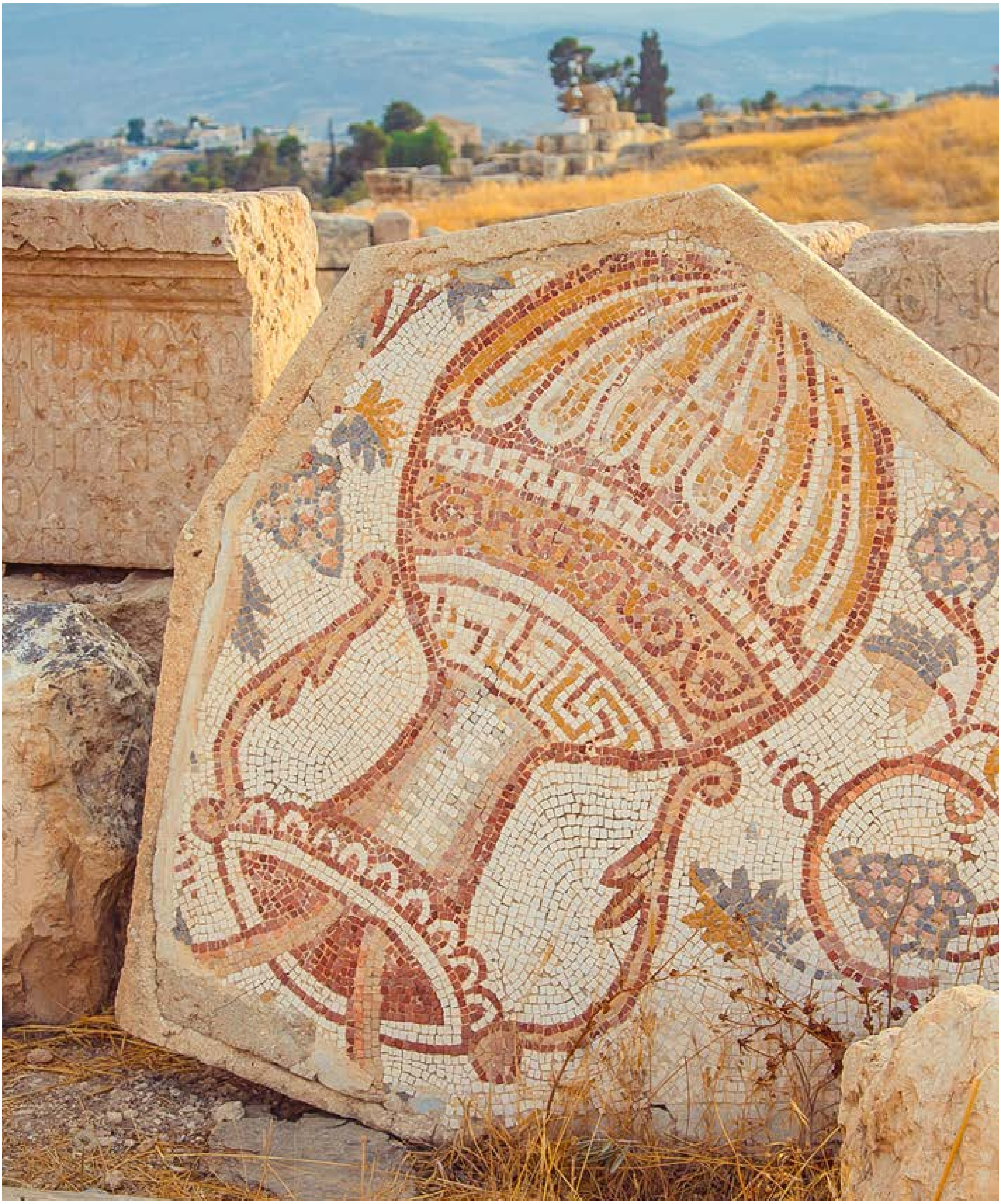

If a child in its first thousand days — from conception to two years old — does not have adequate nutrition, the damage is irreversible."correlations, and more correlations can lead to more discoveries

Josette Sheeran

The Sixth EMPHNET Regional Conference – Amman, Jordan
Roundtable Discussions: Tuesday, March 27 – Thursday, March 29

The Sixth EMPHNET Regional Conference offers a selection of roundtable discussions to highlight pertinent issues to the Eastern Mediterranean Region (EMR) and offer opportunities to "adapt to the regional context." During the conference, expert panelists will exchange experiences with attendees on Towards the consolidation of an FETP network in the EMR, One Health to Achieve Global Health Security, Polio Eradication Efforts and Transition Planning for Measles Elimination, Mobile Data Collection and other Innovative Tools to Enhance Decision Making, Confronting Candida auris: An Emerging Multidrug-Resistant Global Pathogen, and Functioning and Sustainable Country Public Health Emergency Response Operation Framework.

1. **Towards the Consolidation of an FETP Network in the EMR**
   Date and Time: March 27, 9:00-10:30
   Since 2010, EMPHNET has been providing support to existing FETPs and supporting the establishment of new FETPs in the region. The network is growing over time and the number of FETP graduates is now more than 700, which represents an invaluable core capacity for the countries health systems. EMPHNET has already undertaken some activities and initiatives to interconnect FETP graduates in a community of practice. EMPHNET is currently strengthening the emergence of a community of practice in field epidemiology through the use of Information Communication Technology (ICT). This roundtable session will give the opportunity to all FETP family members to share methods and strategies that could help in growing the FETP network and improving the channels for sharing experiences from the field and within public health systems.

2. **One Health to Achieve Global Health Security**
   Date and Time: March 27, 14:00-15:30
   Addressing emerging and re-emerging zoonotic diseases as a health security issue will promote rapid detection of novel biological threats as well as assist in identifying contextualized solutions for the management of these diseases, both from a security and safety perspective as well as from a disease control perspective. The purpose of this roundtable discussion is to identify how the approach of "One Health" can advance the goals of applied public health training programs in the Eastern Mediterranean Region. These discussions will contribute to more effective planning for rapid investigation and response activities for zoonotic disease events. At the end of the roundtable, panelists will provide recommendations for large-scale integration for improving zoonotic diseases management and related best practices among the human, animal health and environmental sectors.

3. **Polio Eradication Efforts and Transition Planning for Measles Elimination**
   Date and Time: March 28, 9:00-10:30
   Countries like Pakistan, Afghanistan and Somalia have not been able to reach as many children with measles campaign as they have been doing with polio supplementary immunization activities (SIAs), although most of the time the implementers of the measles campaigns are those who are conducting the polio campaigns. The goal of this roundtable session is to discuss why priority countries in the Region have not been able to reach with measles campaign as many children and high risk and remote areas as has been the case with polio SIAs, knowing in particular that both campaigns are conducted by the same people. The session will also review the Polio SIAs package of assets, innovative tools and procedures that made polio SIAs highly successful and that could not be used for measles campaigns. The roundtable proposes to tackle these issues using live experiences from two countries of the Region, namely Pakistan and Afghanistan, inputs from experts in this domain from the Region and global level, inputs from other countries' stakeholders and experts present among the audience.

4. **Mobile Data Collection and other Innovative Tools to Enhance Decision Making**
   Date and Time: March 28, 14:00-15:30
   Use of mobile data collection (MDC) is important in many areas such as minimizing data entry and collection, minimizing human errors, and eliminating quality issues related to data capturing, management, processing, and utilization. MDC tools can improve current health systems by providing accurate results and information for public health practice while also supporting the decision-making processes at various levels. This roundtable will bring together experiences from different countries, various technical and programmatic areas of the use and application of MDC, and projects supported by different organizations. Panelists will be sharing their perspective and experience form various countries, discussing how MDC works, challenges faced, lessons learned, and how this affected decision-making processes. The purpose of this roundtable is to highlight the use of MDC and other similar innovative tools to enhance decision making, through effective data collection, processing, reporting, analysis, and action/response, by providing experience from different countries in the region.

5. **Confronting *Candida Auris*: An Emerging Multidrug-Resistant Global Pathogen**
   Date and Time: March 29, 9:00-10:30
   Candida auris is an emerging global pathogen that poses the problem of multi-drug resistance. It can cause various infections such as: bloodstream infections, urinary tract infection, otitis, surgical wound infections skin abscesses related to insertion of the catheter, infection of the heart muscle, meningitis, bone infections, and wound infections. Such infections are challenging to identify and treat. As for treatment challenges, many cases of *Candida auris* infections in many countries of the world were found to be multi-drug resistant to all three antifungal classes. The limited capacity for identification coupled with the multidrug resistance instigate a need to investigate applicable management strategies to prevent, detect and control this virulent pathogen. The purpose of this roundtable is to address *Candida auris* as an emerging global pathogen by providing a concise overview of the pathogen, pinpointing challenges associated with prevention, detection and control, and raising awareness of relevant professionals in the Region to help them develop and implement adequate detection and control strategies.

6. **Building Functioning and Sustainable Country Public Health Emergency Response Operation Framework**
   Date and Time: March 29, 14:00-15:30
   Countries should ensure the major components of their public health emergency response plan are built together, integrated in a solid framework, and supported by strong coordination and regulation mechanisms—including the incident management system, oversight committees and others. For better sustainability of this framework, it should be backed through strong links with relevant human resource capacity building opportunities like FETPs, relevant academia, national institutes of health, etc. The roundtable discussion will serve to highlight the importance for countries to develop their public health emergency response operation capacities while capitalizing on all available opportunities such as their FETP graduates and residents. The discussion will also discuss ways to adopt a comprehensive approach that can result in an integrated and well-functioning operational package which links among rapid response teams, emergency operation center, country FETP and other available relevant human resources capacity building opportunities.

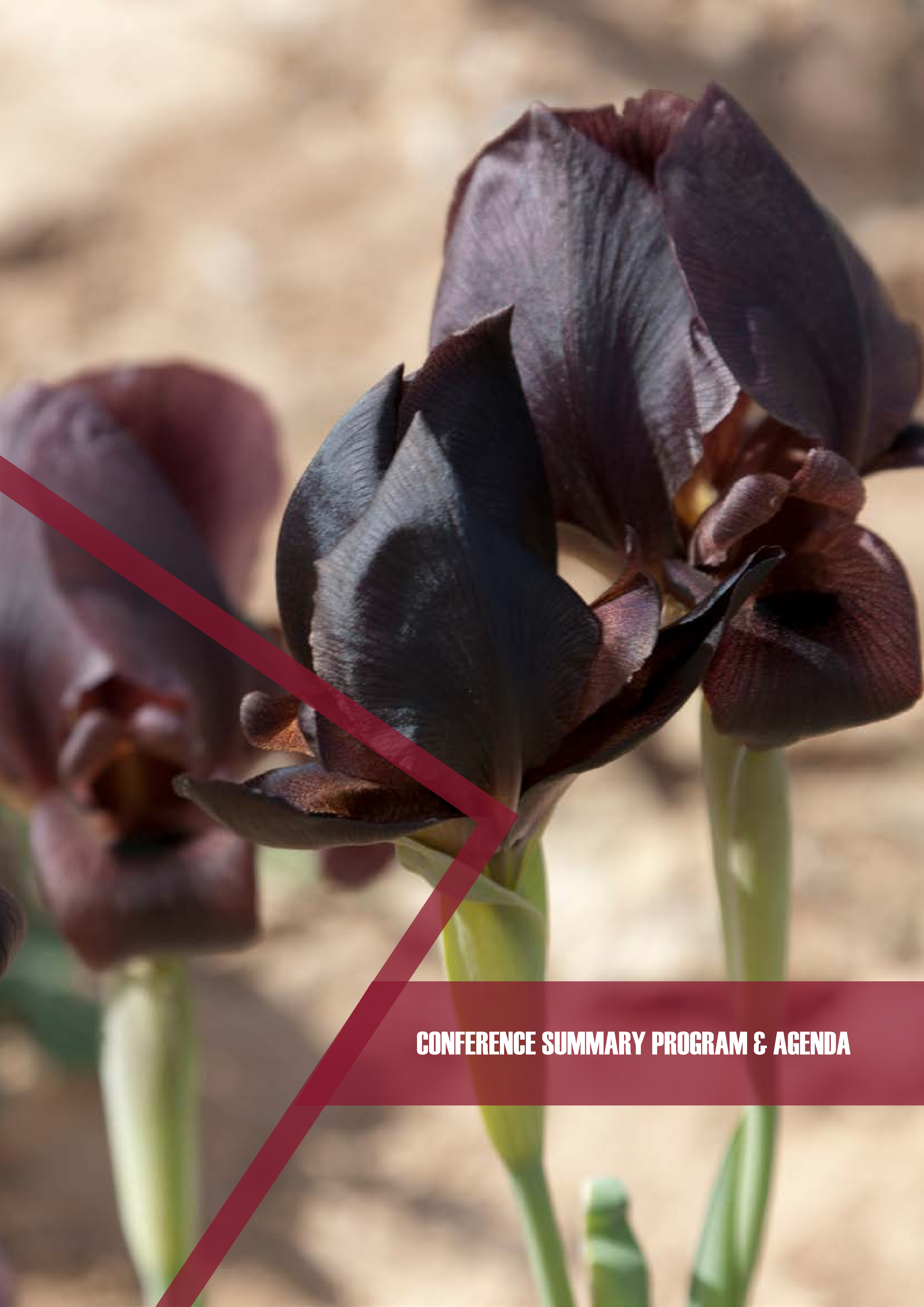

CONFERENCE SUMMARY PROGRAM & AGENDA

Sixth EMPHNET Regional Conference - Pre-conference Workshops
Amman, Jordan - Landmark Hotel - March 26, 2018

Workshop Title	Number of Participants	Duration
**FETP Accreditation**	**Up to 25**	**1 day**
Country Polio Transitional Plans and Routine Immunization	Up to 25	1 day
**Innovative Disease Surveillance**	**Up to 25**	**1 day**
Rapid Response Teams	Up to 25	1 day
**Human and Animal Brucellosis**	**Up to 25**	**1 day**

Sixth EMPHNET Regional Conference Registration
Amman, Jordan - Landmark Hotel - March 25-27, 2018

Time	Registration Date	
**09:00 - 18:00**	**March 25**	**Registration Open**
09:00 - 15:00	March 26	Registration Open
**07:30 - 08:30**	**March 27**	**Registration Open**

Sixth EMPHNET Regional Conference Opening Ceremony
Amman, Jordan - Landmark Hotel - March 26, 2018

Time	
**19:00 - 20:00**	**Opening Ceremony**

Sixth EMPHNET Regional Conference Day One
Amman, Jordan - Landmark Hotel - March 27, 2018

Time	Session 1	Session 2	Session 3
**08:00 - 09:00**	**Key Note Speaker**		
09:00 -10:30	Roundtable 1: Towards the Consolidation of an FETP Network in the EMR		
**10:30 -11:00**	**Networking Break**		
11:00 - 13:00	Respiratory Diseases	Vaccine Preventable Diseases	Surveillance System
**13:00 - 14:00**	**Lunch Break**		
14:00 - 15:30	Roundtable 2: One Health to Achieve Global Health Security		
**15:30 - 15:45**	**Networking Break**		
15:45 - 17:00	Outbreak Investigation/ Foodborne Diseases	Maternal and Child Health	Field Epidemiology Training Programs Activities

Sixth EMPHNET Regional Conference Day Two
Amman, Jordan - Landmark Hotel - March 28, 2018

Time	Session 1	Session 2	Session 3
**08:00-09:00**		**Key Note Speaker**	
09:00 -10:30		Roundtable 3: Polio Eradication Efforts and Transition Planning for Measles Elimination	
**10:30 -11:30**	**Outbreak Investigation/ Vaccine Preventable Diseases**	**Vectorborne Diseases**	**Surveillance System/ Vaccine Preventable Diseases**
11:30 - 11:45		Networking Break	
**11:45 13:00**	**Outbreak Investigation /Waterborne Diseases**	**Vectorborne Diseases**	**Surveillance System/ Vaccine Preventable Diseases**
13:00 - 14:00		Lunch Break	
**14:00 - 15:30**		**Roundtable 4: Mobile Data Collection and Other Innovative Tools to Enhance Decision Making**	
15:30 - 15:45		Networking Break	
**15:45-17:00**	**Outbreak Investigation/ Waterborne Diseases**	**Anti-Microbial Resistance**	**Innovative Tools in Surveillance**
17:00 - 19:00		Free Time	
**19:00 - 22:00**		**Gala Dinner**	

Sixth EMPHNET Regional Conference Day Three
Amman, Jordan - Landmark Hotel - March 29, 2018

Time	Session 1	Session 2	Session 3
**08:00-09:00**		**Key Note Speaker**	
09:00 -10-30		Roundtable 5: Confronting Candida Auris: An Emerging Multidrug-Resistant Global Pathogen	
**10:30 -10:45**		**Networking Break**	
10:45 - 13:00		Poster Session	
**13:00 - 14:00**		**Lunch Break**	
14:00 - 15:30		Roundtable 6: Building Functioning and Sustainable Country Public Health Emergency Response Operation Framework	
**15:30 - 17:00**	**Outbreak Investigation/ Vaccine Preventable Diseases/Measles**	**Non-communicable Diseases**	**Zoonotic Diseases**
17:30 18:30		Closing Ceremony	

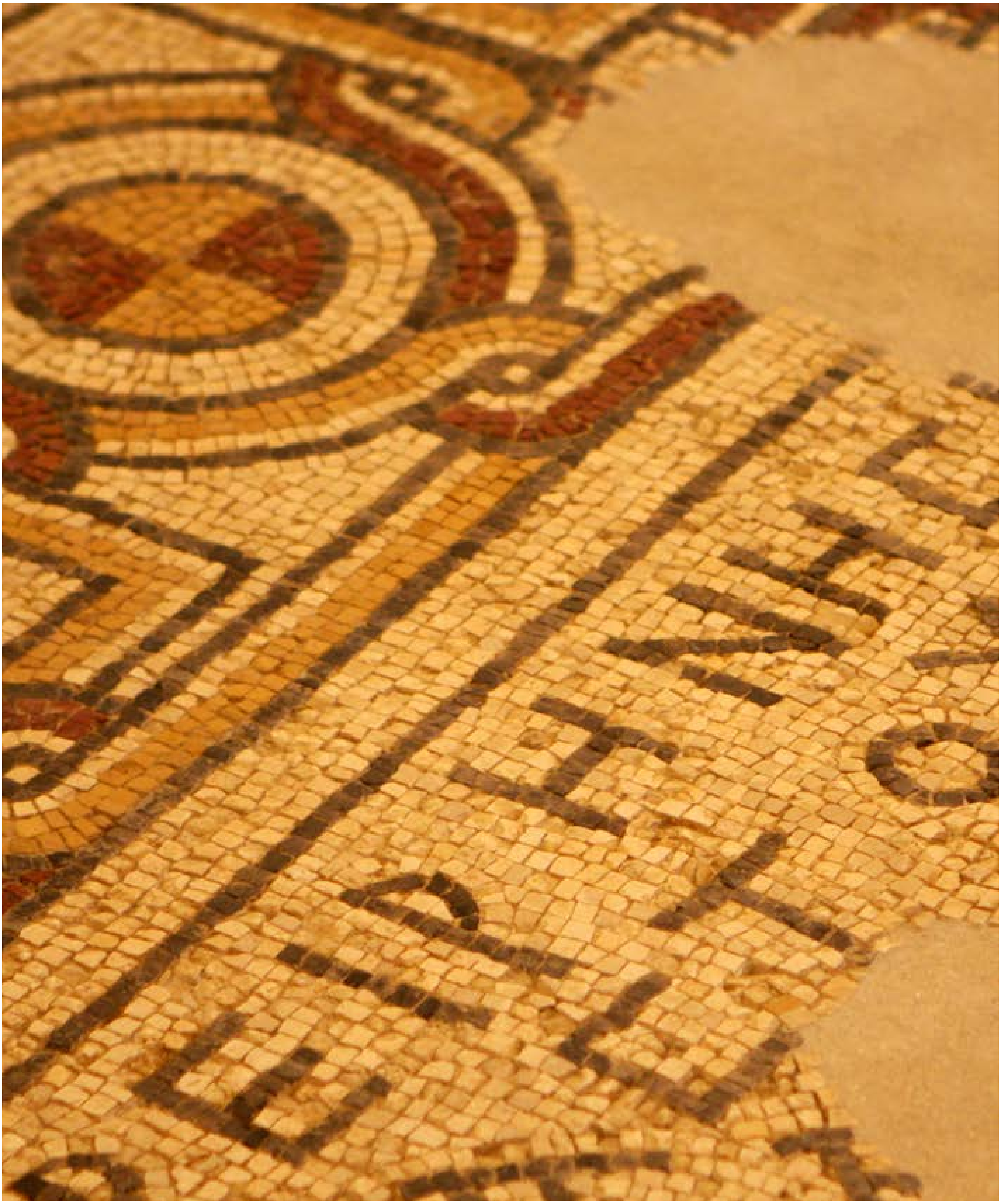

"As our world continues to generate unimaginable amounts of data, more data lead to more correlations, and more correlations can lead to more discoveries"

Hans Rosling

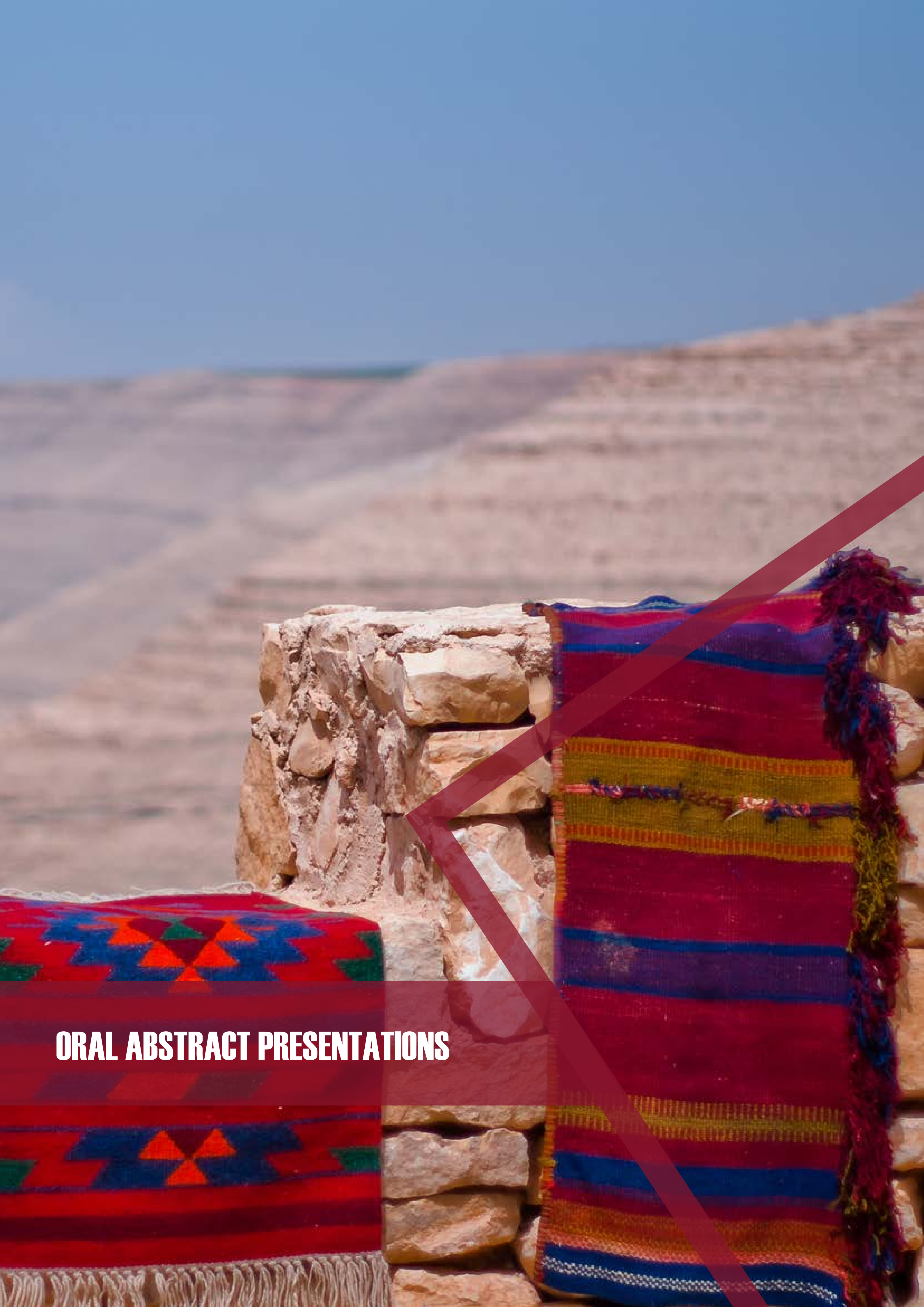

ORAL ABSTRACT PRESENTATIONS

Anti-Microbial Resistance

Abstract Code: 2017-EOH-297

Presenter Name: Dr. Jihan Abdulmughni

Country: Yemen

Presenter Email: gihanthr@gmail.com

Author: E. Mahyoub, A. Al Agabri, Y. Abdulwareth, A. Al-Serouri

Title Drug Resistant Tuberculosis: An Emerging Public Health Threat in Yemen, 2014-2016

Background Drug-resistant tuberculosis (DR-TB) is a serious obstacle for successful TB control. The 2010–2011 Yemen DR-TB Survey showed an overall Multi DR-TB (MDR-TB) prevalence of 2.9% that was 1.4% in newly- and 14.4% in previously- treated patients. DR-TB Surveillance system was introduced in Yemen in December 2013 to improve detection and management of DR-TB.

Purpose To describe the pattern, drug sensitivity and treatment outcome of DR-TB.

Methodology Data on DR-TB cases from 2014 to 2016 was obtained from National Tuberculosis Control Program (NTCP). The NTCP modified WHO case definitions for treatment outcome was used where treatment success defined as completing treatment according to program protocol with at least five consecutive negative smears from samples collected at least 30 days apart in the final 12 months of treatment.

Results Out of 32,528 TB patients diagnosed during 2014 -2016, 115 (4/1000) were DR-TB. The highest number was reported from Aden (40%) and lowest from Taiz 12%; 59% among males; and 67% among most productive years (24-45 years). Furthermore, 97% was among previously treated TB patients and 3% was positive for HIV. MDR-TB confirmed in 68% and Rifampicin Resistance (RR-TB) in 31%. The treatment success rate was 70% and death rates was 15%. Detection and enrollment rates were 27% and 80%.

Conclusions Despite NTCP efforts to respond to the challenge of DR-TB in Yemen, scaling-up of DR-TB services and removing the access barriers are crucial to increase the detection rate. Comprehensive strategies targeting priority population especially those enhancing treatment availability need to be implemented to increase enrollment. More intensive efforts to better manage MDR/RR-TB through adapting WHO shorter recommended regimen and inpatients management for those requires hospitalization will help to improve the treatment success and minimize further emergence of totally drug-resistant TB cases.

Anti-Microbial Resistance

Abstract Code: 2017-HIV/STI-222

Presenter Name: Dr. Kenza Bennani

Country: Morocco

Presenter Email: kenzabennani20@gmail.com

Author: G. Bukassa, A. Khattabi, M. Akrim, A. Maaroufi

Title Evaluation of Drug-Resistant Tuberculosis Surveillance System Before and After Implementing "Genexpert" in Morocco, 2007-2016

Background Drug-resistant tuberculosis (DR-TB) is a serious problem in the fight against tuberculosis worldwide. WHO has been actively encouraging countries to establish continuous DR-TB surveillance systems based on routine drug susceptibility testing (DST). In Morocco, a DR-TB surveillance system was implemented to monitor and to control epidemiology of Multi-drug-resistance TB (MDR-TB). "Genexpert" for Rifampicin resistance detection was introduced in 2012, to scale up of national surveillance system.

Purpose Our study aims to evaluate DR-TB surveillance system before and after implementing "Genexpert."

Methodology This is a retrospective descriptive study with a quantitative approach, to assess the data quality, sensitivity (ability to detect MDR-TB cases compared to cases estimated from national MDR-TB survey) and the PPV of the surveillance system, using data reported from 2007 to 2016 and laboratory results for "Genexpert" and Conventional DST, as gold standard, to validate the data collected.

Results 1063 DR-TB cases confirmed reported from 2007 to 2016, among them 92% were MDR-TB. The missing data for the variables assessed represented 11%. It was important for DST results (32%) and treatment outcomes (34%). The sensitivity of DR-TB surveillance system was low (17% on average) before introducing "Genexpert" (2012) and reached to 70% since 2015. In 2016, the sensitivity was 100% for detection of MDR-TB among patients previously treated for TB and 29% among new TB patients. The PPV of DR-TB surveillance system was 34% before introducing "Genexpert" (2012) and reached to 99% in 2016.

Conclusions The current DR-TB surveillance system based on active case detection was satisfactory for data quality, sensitivity, and PPV. This is related to introduction of "Genexpert" since 2012. It's recommended to strength MDR-TB detection among new TB cases using "Genexpert" and to maintain MDR-TB detection among patients previously treated for TB to improve the sensitivity of the DR-TB surveillance system in order to establish continuous DR-TB surveillance system.

Anti-Microbial Resistance

Abstract Code: 2017-HIV/STI-450

Presenter Name: Dr. Farida Khudaidad

Country: Pakistan

Presenter Email: drfaridakhan9@gmail.com

Author: A. Saeed

Title Evaluation of Drug Resistant Tuberculosis (DR-TB) Surveillance System in Balochistan Province, 2016

Background Eight million people are infected and 3 million die due to TB every year. Pakistan ranks 5th amongst 22 High burden countries with TB and 4th among 27 DR-TB high burden countries and accounting for about 81% of all estimated TB cases worldwide. Pakistan contributes about 65% of TB burden in Eastern Mediterranean Region. In Balochistan patients are filtered from chest and medicine OPDs of tertiary care hospitals and from Basic Management Units (BMU) of districts, sputum of eligible DR-TB patient is sent by transportation mechanism. Diagnosis of DR-TB requires Gene-Xpert testing, available at 04 Programmatic Management Drug Testing (PMDT) sites.

Purpose An evaluation of MDR-TB surveillance system was conducted to identify strengths and weaknesses of surveillance system and make recommendations for improvements.

Methodology Evaluation was performed during March-July 2017 for year 2016. Qualitative and quantitative assessment of system attributes utilizing the Updated CDC Guidelines for Evaluating Public Health Surveillance Systems, 2001 was conducted. A desk review of all available departmental reports and literature was undertaken. Stakeholders were identified, and interviews were held using a semi-structured questionnaire.

Results Case Definition is simple and uniform. System is less flexible but able to integrate with other systems. Data quality is good as 95% forms found complete. Reporting is based on clinical Signs and symptoms and confirmation with gene expert, DST and culture. Acceptability is good, has good coordination with other health systems. Timeliness is good as reporting is within 24-48 hours. System is stable and secure because of timely response. Representativeness is good as 80% public and private sectors are involved. Sensitivity and PVP calculated was 73.4% and 100% respectively for year 2016.

Conclusions System is working effectively at PMDT sites, but Response of private sectors is poor. Recommendations were to sensitize private doctors and paramedics around the province about DR-TB. To increase public private collaboration.

Anti-Microbial Resistance

Abstract Code: 2017-EOH-525

Presenter Name: Dr. Mir Muhammad Hassan Bullo

Country: Pakistan

Presenter Email: docbullomir@gmail.com

Author: A. Baig

Title Evaluation of Risk Factors for Developing Multidrug Resistant Tuberculosis in Rural Islamabad, Pakistan

Background Multi Drug Resistant Tuberculosis (MDR-TB) has emerged as a public health issue globally and especially in developing countries. An adequate and baseline epidemiological information on MDR-TB is critical for effective control and prevention of MDR TB.

Purpose To evaluate the risk factors for developing MDR-TB among the patients registered under TB-DOTS at Federal General Hospital (FGH) Islamabad.

Methodology A case control study **was** carried out at FGH Islamabad from 15 November to 20 December 2016. A case was defined as "any TB patient visiting FGH Islamabad found resistant to anti-tuberculous therapy (ATT) and confirmed by Gen Expert test from January 2014 to December 2016." While a control was defined as "any TB patient who is not resistant to ATT and is negative on Gen expert test." Three Age and sex matched controls were selected for each case. Data was collected using structured questionnaire. Frequencies were calculated, and odd ratios determined at 95% confidence interval and p value of <0.05.

Results Among total of 27 cases 14 (51%) were male. The mean age of the cases was 31 years (range13-61 years). Most of the cases belonged to age group 20-30 years n=11(40.7%). On bivariate analysis, out of a total of 27 cases, 07 were found to have defaulted from TB treatment, (OR: 6.71, CI: 1.7-25), 12 had a contact with MDR TB patient (OR: 5.6, CI: 2-15), 22 had a poor socio-economic status (OR: 3.1, CI:1.1-9.2) and 14 had poor knowledge about MDR-TB (OR: 2.8, CI:1.1-7.4).

Conclusions ATT failure, contact with MDR-TB patient, poor knowledge about MDR-TB were found to be associated with having MDR-TB. Awareness campaigns at an institutional and patient levels was recommended. On recommendations of this study awareness campaign was started in FGH regarding significant risk factors for MDR-TB.

Anti-Microbial Resistance

Abstract Code: 2017-EOH-100

Presenter Name: Dr. Jihan Abdolmughni

Country: Yemen

Presenter Email: gihanthr@gmail.com

Author: Y. Abdulwareth, A. Al Hamady, E. Mahyoub, A. Alagbari, A. Al-Serouri

Title Evaluation of Multi Drug Resistant Tuberculosis Surveillance System, Yemen

Background Developing Multi Drug-resistant TB (MDR-TB) is a threat facing the National Tuberculosis Control Program (NTCP) in Yemen. Four sentinel Surveillance sites were established in 2014 to monitor the situation and guide control.

Purpose Assess the performance of MDR-TB Surveillance and provide recommendations.

Methodology We use the Center of Diseases Control (CDC) updated guideline for evaluating public health surveillance systems. In-depth interviews were conducted with six NTCP and 12 central and regional MDR-TB Centers' managers/staff. We used 5-Likert scale to assess performance by attributes (e.g. usefulness, simplicity, flexibility) according to the following scoring system: Poor (<60%), average (60-80%), and good (>80). NTCP-modified WHO case definitions for treatment outcome was used where treatment success defined as completing treatment according to protocol with at least five consecutive negative smears from samples collected at least 30 days apart in the final 12 months of treatment.

Results MDR-TB Surveillance System achieved good performance in usefulness, acceptability and data quality compared to average score in flexibility and simplicity; and poor score in stability. Detection rate was 25% in 2014 and increased to 32% in 2016 compared to the target of 40%. Enrollment decreased from 92% in 2014 to 69% in 2016 compared to 100% target. Treatment success reached 70% in 2016.

Conclusions MDR-TB surveillance system was found to be useful, but more efforts is needed to improve stability through ensuring constant power supply for the laboratory to be able to perform drug sensitivity with gradual replacement of donor's funds by government. Although detection rate showed some increase in 2016, enrollment decreased due to unavailability of MDR-TB drugs and long waiting list. Scaling-up of MDR-TB services and removing access barriers are crucial to increase detection rate together with enhancing treatment availability to increase enrollment are recommended. Adapting WHO shorter regimen and inpatients management when needed will help to improve treatment success.

Anti-Microbial Resistance

Abstract Code: 2017-EOH-84

Presenter Name: Dr. Yasser Ghaleb

Country: Yemen

Presenter: Email y.ghaleb@ymail.com

Author: A. Al Serouri, M. Alamad, S. Nasher, A. Alsoumainy

Title The Growing Antimicrobial Resistance of Urinary Tract Infections, Sana'a- Yemen, 2015

Background Urinary tract infections (UTIs) remain a growing public health concern as it may lead to treatment failure and increasing morbidity and mortality. To optimize empirical antibiotic prescription, it is important for clinicians to have a working knowledge regarding UTIs etiological pathogens and its susceptibility patterns.

Purpose Determine the prevalence of UTIs, describe their responsible pathogens, and their antimicrobial resistance.

Methodology 2015 data on patients attended the Microbiology Department in National Centre for Public Health laboratories (NCPHL) for urine cultures was obtained. UTIs defined as =100,000 CFU/mL of an uropathogen in midstream urine culture. The causative pathogen was identified, and antibiotic resistance carried out by disc-diffusion method.

Results Out of 2901 patients examined, 58% were females and half among 20-40 years age group. The prevalence of UTIs was 34% and more among females: Odd Ratio (OR): 1.8 (1.5-2.0) and elderly = 60 years (OR: 1.4 (1.1-1.8). Gram-negative bacteria constitute 73% of UTI and found to be highly resistance to Nalidixic acid (70%), Co-trimoxazole (64%), and Piperacillin (62%). Gram-positive bacteria that constitutes 27 % found to be highly resistant to Co-trimoxazole (81%), Norfloxacin (69%) and Amoxicillin (67%). E. coli was the most common pathogen (42%), followed by coagulase negative Staphylococci (10%) and Klebsiella (8%). While E. coli found to be resistant to Co-trimoxazole (66%) and Nalidixic acid (71%), Klebsiella was resistant to Co-trimoxazole (88%) and Nalidixic acid (64%), and Coagulase Negative Staphylococci to Co-trimoxazole (88%) and Amoxicillin (75%).

Conclusions Findings highlight the doubling of UTIs prevalence and growing antibiotics resistance e.g. for Nalidixic acid from 54% to 70% since 2002. Results should guide antibiotic prescribing and developing strategies for controlling resistance. It also underlines the need to establish Antimicrobial Stewardship Program to reduce selection pressure and minimize resistance.

Field Epidemiology Training Programs Activities

Abstract Code: 2017-OTH-320
Presenter Name: Dr. Abdul Rauf Shirzad
Country: Afghanistan
Presenter Email: a.rauf.shirzad@gmail.com

Title An Analysis of Interventions Presented at the 2015 CDC EIS Conference and the 2015 TEPHINET Conference

Background Field Epidemiology Training Programs, TEPHINET, and the CDC EIS Program concentrate almost exclusively on non-intervention epidemiology. Fields of health services research that concentrate on interventions such as randomized controlled trials or operations research are seldom taught. We believe this is a mistake; unless interventions are performed, health and healthcare are not improved. We analyzed the oral presentations of the 2015 EIS Conference (2015 EISC) and the 2015 TEPHINET Conference (2015 TEPHINETC) to determine how frequently interventions were performed.

Purpose To determine the frequency that authors perform interventions.

Methodology Three reviewers read abstracts. Two reviewers read the 2015 EISC and two the 2015 TEPHINETC. Abstracts were judged to have had an intervention or not and, if so, whether the principal intention was to present an intervention ("primary intervention") or, if the intervention followed a noninterventional epidemiology investigation, a "secondary intervention." Abstracts were then graded by whether the presenters called for an intervention to be done or not ("intervention needed"). Discrepancy in scoring was resolved by discussion.

Results Neither set of abstracts contained the words "randomized controlled trial," "operations research" or "operational research." The 2015 TEPHINETC contained 126 presentations, of which 28 (22.2%) contained an intervention (three primary, 2.4%; 25 secondary, 19.8%). Eighty-five abstracts (67.5%) called for an intervention to be done. The 2015 EISC contained 103 presentations but we have been able to read to date only 61. Of these, 11 (18.0%) contained an intervention (three primary, 4.9%; 8 secondary, 13.1%). Thirty abstracts (49.2%) called for an intervention to be done.

Conclusions CDC and TEPHINET do not emphasize intervention epidemiology. While the need for an intervention is highlighted by CDC and FETP authors, it is unclear who will do those or if they will be done. We believe FETP and CDC EIS training programs should be revised to emphasize intervention epidemiology.

Field Epidemiology Training Programs Activities

Abstract Code: 2017-OTH-200
Presenter Name: Dr. Hala Saad
Country: Egypt
Presenter Email: halaaabahaa@gmail.com
Author: S. Elshourbagy, H. Abu Elsood, S. Refaey, A. Kandeel

Title Recruitment of Cohort Number 20 of Field Epidemiology Training Program – Lessons Learned - Egypt, 2017

Background The Field Epidemiology Training Program (FETP) had been established in Egypt since 1993. It was the second in the Eastern Mediterranean region. Up to this year, 18 cohorts were graduated from FETP. Approximately 80% of graduates work in the Egyptian ministry of health and population (MOHP) and many fill leadership positions. Others provide essential epidemiological services abroad and to neighboring countries.

Purpose We aim to describe and evaluate recruitment process for new FETP residents.

Methodology An announcement for recruitment of a new cohort was published on newsletter and on FETP Egypt Facebook page on September 31 for two weeks. Inclusion criteria listed on the announcement based on MoHP requirements. Interested candidates were encouraged to fill an application on FETP Egypt website. The application consists of open ended as well as closed questions. Data were extracted, edit and cleaned using Microsoft Excel. One hundred applicants were short listed and invited by phone call to a Face-to-Face interview. Candidate evaluation was web based, it included measurement of skills, qualifications, and experience using Likert scale.

Results Out 364 responses, 39/346 (11.3%) were replicate applications making 307 total applicants. A total of 269/307 (87.6%) applicants were eligible for FETP requirements. Proportion of females was 176/269 (65.4%). Out of 269 candidates; pharmacists, physicians, dentists and veterinarians were 170 (63.2%), 73 (27.1%), 20 (7.4%), 6 (2.2%) respectively. Out of 100 shortlisted applicants; 87 (87%) responded to the call for the interview. A total of 75 interviewed candidates were placed in various departments of preventive sector; 27 (36.0%) at central level and 48 (64.0%) at directorate level representing 19 governorates. Seven candidates (8.0%) were out of the preventive sector and five (5.7%) were veterinarians serving ministry of agriculture.

Conclusions The online applications made recruitment process ran smoothly; it was much easier and less time consuming. However, open ended questions could be closed.

Field Epidemiology Training Programs Activities

Abstract Code: 2017-OTH-199

Presenter Name: Dr. Hala Saad

Country: Egypt

Presenter Email: halaaabahaa@gmail.com

Author: S. Elshourbagy, H. Abu Elsood, S. Refaey, A. Kandeel

Title Empowerment of Public Health System in Rapid Response and Early Detection by Building Front-line Epidemio logical Capacity - Egypt, 2017

Background The Egyptian Ministry of Health (MoH) launched Public Health Empowerment Program-Basic Field Epidemiology (PHEP-BFE). PHEP-BFE is a three-month in-service training program that focuses on detection of and response to diseases and events of public health concern. In July 2017; first training course was conducted for first cohort and graduation was in October 2017.

Purpose We aimed to raise the epidemiological capacity of sanitarians due to their valuable contribution in detection of and response to public health threats and evaluate program effectiveness.

Methodology A team from MoH was assigned for planning, preparing and rephrasing of training material to fit Arabic language. Material from the Front-line Field Epidemiology Training Program (FETP), referenced by CDC, was used for developing parts of the curriculum. Inclusion criteria for participants were applied. Three workshops were conducted, and participants were asked to conduct field activities after each workshop. Each participant was supervised by a mentor. Evaluation approaches and tools include; documents review, questionnaires and check lists. Instructors, Mentors and Participants were asked to complete an evaluation tool after each workshop and fieldwork. Data was collected and analyzed.

Results Out of 27 Participants joined the program, 25 (92.6%) were graduated. All participants were males aged from 24-50 years. Out of 25 participants, 19 (76.0%) were at directorate level. During their fieldwork, 22/25 (88.0%) noticed low surveillance reporting at district level, and 15/25 (60.0%) participated in outbreak investigations. By the end of training; knowledge of subject of epidemiology was improved, the difference between results of pre- and post-test raised by (30.4%) $P<0.0001$. All participants gained presentation skills and were able to write scientific reports. Yet 16/25 (64.0%) participants need practicing data analysis on Microsoft Excel during training workshops.

Conclusions PHEP-BFE was successful and met its objectives. PHEP-BFE could target sanitarians at district level. More time could be dedicated for statistical skills improvement.

Field Epidemiology Training Programs Activities

Abstract Code: 2017-EOH-317

Presenter Name: Dr. Adel Al-Rawahneh

Country: Jordan

Presenter Email: adel_rawahneh@yahoo.com

Author: M. Rawahnih, Y. Khader

Title Assessment of Water, Sanitation and Hygiene (WASH) within Healthcare Facilities in Selected Eastern Mediterranean Countries

Background Inadequate drinking water, sanitation, and hygiene (WASH) in health care facilities impacts the health, particularly in low and middle-income countries. There is limited knowledge on the status of WASH in such settings.

Purpose The primary objective of this study was to assess WASH conditions in health care facilities in four EM countries; Jordan, Morocco, Lebanon, and Pakistan.

Methodology This study was based on secondary data analysis of the regional study on WASH services in health facilities. Separate samples of health care facilities were selected from Jordan (19 hospitals), Morocco (8 hospitals), Lebanon (14 hospitals), and Pakistan (8 hospitals) and were assessed using the WHO/CEHA tool "WSH in the health facilities assessment tool". The assessment tool consisted of items to assess the WSH services availability, adequacy, and functionality.

Results All health care facilities (100%) in Jordan and Morocco, 71.4% of hospitals in Lebanon and none of the hospitals in Pakistan had a safe water source. Overall, all hospitals in Jordan, Morocco, and Lebanon and 71.4 % of hospitals in Pakistan had improved and gender separated toilets in inpatients settings (One per 20 users). About 84.2% of hospitals in Jordan, none in Morocco, 28.6% in Lebanon, and all hospitals in Pakistan had sufficient improved and gender separated toilets in outpatients setting. Overall, 84.2% of hospitals had sufficient and functioning handwashing basins with soap and water and 79.0% of hospitals had sufficient showers. The majority of hospitals in the selected countries have a policy for the safe management of healthcare waste but inadequate training program on healthcare waste.

Conclusions WASH services are not well implemented in health care facilities of the selected countries. The countries have to develop and implement a monitoring system for WASH services or at least support inclusion of WASH services in routine monitoring of health care services.

Field Epidemiology Training Programs Activities

Abstract Code: 2017-OTH-77

Presenter Name: Dr. Nansi Abdulrahim

Country: Jordan

Presenter Email: luarance2013@yahoo.com

Author: I. Alasasfeh, A. Abusalieh, I. Ablan, Y. Khader

Title Infectious Diseases Surveillance and Notification in Jordan: Physicians' Knowledge, Attitude and Practice

Background Public health surveillance provides crucial information for monitoring the health of the public, identifying public health problems, and triggering action to prevent further illness. Health professionals' knowledge and awareness of the disease surveillance is essential for effective reporting diseases to health departments.

Purpose This study aimed to identify the knowledge and attitudes of Jordanian physicians towards public health surveillance.

Methodology A cross-sectional study was conducted among resident doctors who were working in four main ministry of health (MOH) hospitals and two teaching hospitals in Jordan in September 2017. Residents from all specialties were visited by the researcher and invited to participate in this study. A self-administered paper-based questionnaire was used to collect the data. The questionnaire collected information about socio-demographic and practice-related characteristics of physicians and included items to assess their knowledge of surveillance and reporting practices.

Results This study included 223 physicians (152 males and 71 females). About 60.1% of the residents were graduates from medical schools in Jordan and the remaining (39.9%) were graduates from medical schools in other countries. Approximately two thirds of residents (62.3%) were doing their residency in MOH hospitals and the rest (37.7%) in two teaching hospitals. Only 44.8% of physicians had defined surveillance correctly. About 53.8% identified population surveys and case reporting as a source of public health surveillance data. Only 27.4% of physicians had been educated or trained on surveillance. About 39.5% of physicians had filled at least one report form during their practice. The main reasons for not reporting mandatory diseases were high workload (49.8%) and being not trained on reporting diseases (46.6%).

Conclusions A relatively high percentage of physicians have insufficient knowledge of surveillance and reporting of notifiable infectious diseases. Training of physicians on surveillance and diseases notification is highly needed. The practice of disease notification should be enforced in Jordanian hospitals.

Innovative Tools in Surveillance

Abstract Code: 2017-OTH-413

Presenter Name: Dr. Ashwaq Joodi

Country: Iraq

Presenter Email: dr.ashwaq6@yahoo.com

Title Effect of Reminding Parents on Vaccination Dates Using Mobile Short Messages on the Routine Vaccination Coverage of Infants in Al Resafa-Baghdad, 2015-2016

Background Vaccination is one of the most successful and cost-effective public health interventions. Public confidence in immunization is critical to sustaining and increasing vaccination coverage rates and preventing the outbreaks of Vaccine Preventable Diseases (VPDs). New innovative methods involving technologies need to be employed to increase the vaccine coverage. Technology use is widespread by patients and providers including text message, email, internet, social media and electronic health records.

Purpose To assess the effect of reminding parents on their children's vaccination dates using Short Message System (SMS) on the coverage rates of vaccines in the Primary Healthcare Centers (PHCs) in Baghdad, Iraq, 2015-2016.

Methodology An interventional study was conducted in six PHCs that were selected by simple random sampling technique from all PHCs in Resafa side of Baghdad of 4.8 million inhabitants. All Infants aged less than one year who missed any of the vaccines enlisted in the national immunization schedule in these PHCs were considered defaulters and included in the study. In three PHCs, the parents of those children received SMS reminders while the children in the other three PHCs were left for the routine defaulter tracing practices.

Results There were 1299 defaulter children in the six PHCs during the study period; 625 infants were in the intervention PHCs and 674 infants in the non-intervention PHCs. Prior to the intervention, there was no significant difference in the vaccines' coverage between the two groups. After the intervention, the coverage rates among the intervention versus non-intervention groups were: OPV1 (67.1% vs. 42.9%; $p<0.001$), OPV2 (58%vs.47.1%; $p<0.001$), OPV3 (67.4%vs.31.9%; $p<0.001$), Penta1 (67.1%vs.42.9%; $p<0.01$), Penta2 (67.4%vs.31.9%; $p<0.001$), and Measles (69.6%vs.21.6%; $p<0.001$), respectively. The average cost per respondent defaulted infant was US$ 0.3.

Conclusions The use of SMS in the PHCs to remind parents with defaulters' infants proved effective in improving the vaccination coverage rates.

Innovative Tools in Surveillance

Abstract Code: 2017-OTH-250
Presenter Name: Ms. Dalal Youssef
Country: Lebanon
Presenter Email: dalalyoussef.esu@gmail.com
Author: S. Hemedeh, G. Allouch, A. Yaghi, I. Kaysar, A. Jouny, F. Ghoussaini, H. Zreik

Title Laboratory Based Surveillance Using District Health Information System (DHIS2): Lebanon 2017

Background The Ministry of Health in Lebanon is moving to electronic surveillance using DHIS2. Laboratory-based surveillance is one of the pillars of infectious diseases surveillance.

Purpose The main objective is to have real-time information flows in order to timely detect alerts and outbreaks. The existence of multiple surveillance systems for notifiable diseases allows each system to complement the other.

Methodology In 2017, DHIS2 tool was used for laboratory surveillance. As part of the implementation process, the online application was developed. Aggregated-based dataset is customized according to the paper reporting form. Accounts users and generic dashboards are generated for each organization unit. Two rounds of trainings were conducted: one in May for initiation and one in July for consolidation. Each round consists of 6 training sessions targeting 150 focal persons working in all public and private hospitals in Lebanon. Then, all hospitals are requested to fill on weekly basis an aggregated based dataset using DHIS2. The dataset includes laboratory general information, bacteriological cultures, stool direct exam, rotavirus testing, serology and influenza testing. Several indicators are generated.

Results After the second-round training, the completeness rate reached 89.5% in October. Highest reporting rates were recorded in South, Nabatyeh and Baalbeck-Hermel (100%). Timeliness reached 73.4%. Positive bacteriological cultures are as follow: Streptococcus (639), Pneumococcus (269), Listeria (4), Meningococcus (11), Haemophilus (292), Vibrio (3, 2 data entry error and 1 Vibrio Cholera non O1 non O139), Brucella (70), Salmonella (538), Shigella (101), E. coli (2664), Campylobacter (46). The cumulative number of protozoa in stool are: Amoebia (8661) and Giardia (1550). 5702 cases of rotavirus were recorded. Serology testing finds 724 hepatitis A, 17 measles and 82 Rubella.

Conclusions After 6 months of implementation of DHIS-2, timeliness and completeness of laboratory reporting are improved. Errors and bureaucratic delays are minimized. Extension of DHIS2 for case-based surveillance is recommended.

Innovative Tools in Surveillance

Abstract Code: 2017-HIV/STI-219
Presenter Name: Ms. Dalal Youssef
Country: Lebanon
Presenter Email: dalalyoussef.esu@gmail.com
Author: C. Khoury, G. Allouch, K. Haydar, A. Jouny, H. Zreik, F. Ghoussaini, A. Yaghi

Title Use of District Health Information System (DHIS-2) for Real Time Surveillance: Lebanon 2017

Background The Ministry of Health in Lebanon is in the process of migrating surveillance reporting from a cumbersome paper-based system to a web-based electronic platform (DHIS-2).

Purpose The aim of the project is to have real-time information flows in order to timely detect alerts and outbreaks in order to take quickest action.

Methodology DHIS-2 tool was initially piloted in Lebanon in 2014 for school-based surveillance. In May 2017, the tool was extended progressively for other surveillance programs collecting aggregate data from hospitals, medical centers, dispensaries and laboratories. As part of the roll-out process, the online application was developed: customized aggregated-based datasets, organization units, accounts users and generic dashboards. 80 training sessions targeting 1290 users were conducted throughout the country. Those trained included 35 district and province health officers, 150 focal persons working in all public and private hospitals, 140 focal persons in laboratories and 800 in medical centers and dispensaries. To assess improvements in surveillance reporting, we compare completeness and timeliness for reporting for the period before and after the implementation of DHIS2. Challenges and lessons learned during the roll-out process are documented.

Results For laboratory-based surveillance, completeness of reporting increased from 70.8% in May to 89.6% in October. Timeliness has improved from 25% to 74%. For medical centers an improvement of 8.1% in the reporting and 9.4% in the timeliness is recorded before and after training sessions. For zero reporting, completeness remains the same (88%) and timeliness has improved from 74% to 87%. There was also increase in the reporting of communicable diseases. Implementation challenges included limited access to internet (29%) and limited workforce (21%).

Conclusions Implementation of DHIS2 resulted improvement in timeliness and completeness for aggregated data reporting. Continued onsite support, monitoring and system enhancement included internet connectivity are needed to enhance the performance of DHIS2.

Innovative Tools in Surveillance

Abstract Code: 2017-EOH-247

Presenter Name: Mrs. Nadine Haddad
Country: Lebanon
Presenter Email: esu.haddadn@gmail.com
Author: J. Gomes Diaz, A. Paez Jimenez, N. Ghosn

Title Time Series Analysis and Forecasting of Viral Hepatitis A and Typhoid Fever in Lebanon

Background In Lebanon, population is described as either regular (Lebanese and Palestinian) or Syrian displaced (since 2013). Along with such population dynamics, two endemic diseases exhibited changes over past years: Viral Hepatitis A (VHA) and Typhoid Fever (TF). During 2008-2015, national annual rates (per 100,000) varied between 7.7-44 for VHA and 6-11.6 for TF.

Purpose The following time series analysis (TSA) aim to describe trends and seasonality of each disease and generate predictions.

Methodology Cases included in the National database of Epidemiological Surveillance Program of Ministry of Public Health were considered. Descriptive analysis using Excel was performed for each disease for 2008-2015. Weekly and monthly counts were considered for VHA and TF, respectively. Regression models were generated separately for regular and total populations. TSA models for Syrian displaced alone could not be performed as data is available for 3 years only. TSA was performed using Stata v13. Forecasting was generated for 2016.

Results TSA models for VHA and TF show slightly decreasing trends among both regular and total populations. As of 2016W21, average observed weekly VHA rate (per 100,000) is lower than predicted among regular population (0.08 versus 0.13, respectively) and total population (0.04 versus 0.22, respectively). As for TF, observed monthly rates were higher than predicted during 3 first months of 2016 among both regular and total populations.

Conclusions TSA model shows decreasing VHA trend among regular population, despite national outbreak of 2014. As TF also exhibit national decreasing trend, regional analysis can help understand increased monthly TF rates early 2016. Differences between observed and predicted rates should be carefully interpreted with respect to reporting completeness and timeliness, various reporting mechanisms in addition to intervention measures.

Innovative Tools in Surveillance

Abstract Code: 2017-EOH-536

Presenter Name: Dr. Ranya Ahmed
Country: Bahrain
Presenter Email: rahmed@sams-usa.net

Title How Did Data Visualization Identify and Improve Response to an Enteric Fever Outbreak in Syria?

Background Population displacement and damage to infrastructure in Syria has contributed to an increase in water borne infectious diseases including enteric fever. The collection, presentation and analysis of data collected in real time is therefore vital for early identification of outbreaks. EWARN and EWARS are used in Syria for the reporting of communicable diseases however this is not always available to facilities in real time. As such, the prompt availability of diagnostics and treatment may be lacking. This is vital for enteric fever where morbidity and mortality without appropriate treatment is high.

Purpose In this study, we examine the utility of a data visualization tool to track cases of enteric fever in three facilities supported by the Syrian American Medical Society (SAMS.)

Methodology All cases of enteric fever between March 2017 and July 2017 were tracked in near real time and presented using data visualization software, namely Tableau. Tableau is a visualization software generally used within the business sector, but its ability to visually express data has far reaching potential in the realm of public health. Sites in Daraa, Quneitra and Rural Damascus were included. Close communication with data personnel in SAMS' country offices and health workers working in the facilities was maintained.

Results In March 2017, there were 21 cases of enteric fever across the three governorates and in July 2017, this had increased to 784 cases. Close liaison with the teams on the ground identified that facilities were seeing a significant increase in cases and that there was a shortage of appropriate antibiotics to treat patients. It was also apparent that there was a shortage of microbiology facilities which could isolate the causative organisms (Salmonella typhi or paratyphi) and therefore limited ability to identify the susceptibility of the organisms resulting in empiric therapy where available. Using data visualization in real time can allow for preparedness and responsiveness to outbreaks of communicable diseases during the conflict.

Conclusions Visualization should be integrated into online and offline data collection tools in order to create an early warning system on each device used in the field.

Innovative Tools in Surveillance
Abstract Code: 2017-HIV/STI-435
Presenter Name: Dr. Ibrahim ELdeyahy
Country: Egypt
Presenter Email: isi.health.eg@gmail.com
Author: A. Kandeel, A. Eid, H. Abu Elsood, A. El Sabbah

Title Evaluation of Automated Online Quality Checker Implementation for the Brucellosis Surveillance system, Egypt 2016

Background National Egyptian Disease Surveillance System collects data on 40 diseases and conditions from 584 nominated reporting sites out of 27 governorates. Brucellosis is a weekly notifiable disease. Reporting levels of completeness and timeliness may differ that may influence data quality; these attributes needed to be assessed prior further manipulation of data. In 2016, Surveillance Online Checker (SOC) was launched to facilitate quality check to give feedback about timeliness and completeness of optional (to online entry) variables to the reporting sites (Completeness of mandatory variables is 100%).

Purpose We aimed to measure the effect of Surveillance Online Checker on brucellosis surveillance data completeness and timeliness after one year of implementation.

Methodology Brucellosis data completeness and timeliness of selected optional variables from the case reporting form were compared before and after one year (2015-2016) of launching SOC using Chi square and student t-test.

Results It is found that completeness of optional variables were increased from 67.3 % to 78.4% as follows; Detailed Address (67.3% to 78.9%, p<0.0001), Patient Telephone (22.1% to 59.9%, p<0.0001), National ID (2.8% to 23.8%, p<0.0001), Lab Test (81.3% to 86.2%, p=0.043) Final Diagnosis (68.3 to 91.8, p<0.0001) and Investigation Forms (52.3% to 75.2%, p<0.0001). While other variables had no significant change such as Occupation (92.7% to 93.0%, p= 0.946). The reporting during predefined Timeliness increased from 69.1% to 89.8%, p<0.0001, with Average Data Entry Time per Case since its classification improved to be 3.8 days instead of 8.8 days. The average time to retrieve previous calculations shortened from 2 working days /month to just seconds after running SOC.

Conclusions Completeness and timeliness of brucellosis surveillance improved after SOC. It is recommended to utilize SOC results by intermediate and peripheral levels after adapting SOC for these levels and adding additional surveillance attributes to SOC.

Innovative Tools in Surveillance
Abstract Code: 2017-HIV/STI-50
Presenter Name: Dr. Zayid Al Mayahi
Country: Oman
Presenter Email: almayahi96@hotmail.com
Author: F. Alswaidi, A. Alzahrani

Title Perception of the Health Surveillance Users on the Health Electronic Surveillance Network (HESN) of Saudi Ministry of Health

Background Ministry of health in Saudi Arabia implemented a pioneer electronic health surveillance network in 2012. This advanced system is hoped to play an important role in the prevention and control for possible serious health events and facilitate other public health programs.

Purpose To evaluate the perception of active HESN users on its performance as a surveillance system, identify its weaknesses and suggest practical recommendations for improvement.

Methodology A complete list of 11324 HESN users was identified. Active user was defined as a user with minimum use of one single time for either immunization or investigation purposes and has a valid email contact. Out of 1535 active users, 700 participants were selected randomly, and a cross sectional study conducted. A pre-designed electronic questionnaire was sent via emails to all the participants. The response rate was (87%), of which the completed forms were 493 (81%).

Results Mean age was 36.89±9.11 (24-66 years), 57.8% were males, and 44.6% participants were Saudis. Riyadh and eastern province represented the highest two regions of participation; 93 (18.9%) and 70 (14.2%) respectively. about half of participants were nurses 251 (50.9%), whereas doctors composed nearly a quarter 123 (24.9%). There were (66.5%) who had experienced surveillance system and (23.9%) worked with electronic medical records. Majority (85.2%) underwent trainings and (92.2%) worked with outbreak investigation function of HESN. About (70.8%) were generally satisfied with HESN, and (41%) believed that HESN requires improvements. Those who use HESN several times a week are more satisfied (81.3%) than one-time users weekly (75.6%) (P=0.001). Internet speed has also a significant association with the general satisfaction level about HESN, (P<0.001). Users of Google chrome browser are happier (82.3%) than Firefox (78.7%) and Internet explorer users (72%). (P <0.001). There are (60.9%) of Arabs and (57.7%) Saudis who agree that HESN needs improvements, compared to only (42.9%) of non-Arabs (P<0.001).

Conclusions There is a general reasonable satisfaction level amongst HESN users. However, to ensure achieving the real awaited public health goals of HESN and increase the satisfaction level, there have to be certain and important improvements.

Maternal and Child Health

Abstract Code: 2017-FWBD-340

Presenter Name: Dr. Mahnaz Hamed

Country: Afghanistan

Presenter Email: mahnaz406@gmail.com

Title Epidemiological Characteristics of Maternal Deaths in Kabul, Afghanistan, 2017

Background Each day about 1,000 women die worldwide because of complications related to pregnancy and childbirth. Developing countries account for 99% of the deaths and have the highest maternal mortality ratio (MMR). The vast majority of these deaths would be avoidable if their causes were known and successful interventions implemented. The MMR in Afghanistan has been reduced from 1,300 per 100,000 live births in 1990 to 400 per 100,000 live births in 2016, still among the highest in the world, but the country still needs to reduce the ratio to meet the Millennium Development Goal (MDG) 5 target of 325 deaths per 100,000 live births. We determined the causes of MD in four large maternity hospitals.

Purpose To determine epidemiological characteristics of maternal deaths (MD).

Methodology A prospective review of all death data among child bearing women was performed in four hospitals from January 1-November 15, 2017. We used the WHO definition of maternal death.

Results Fifty women died and the mean maternal age at death was 31 years. The leading causes of death were hemorrhage (18 women, 58/100,000), hypertensive events (17 women, 56/100,000), thromboembolic events (5 women, 16/100,000), septicemia (5 women, 16/100,000) and cardiac diseases (3 women, 10/100,000). The gestational age was more than 30 weeks in 40 women (80%); 30 women (60%) were prime gravida; deaths occurred among rural mothers in 39 women (78%); 44 women (88%) had no antenatal care; and 26 women (52%) had existing risk factors. The stages of death were during the postpartum period (31 women, 62%), the antepartum period (10 women, 20%), and labor (9 women, 18%). The MMR was 162 /100,000 live births.

Conclusions The MMR was surprisingly low, suggesting Afghanistan can achieve the MDG goal in areas served by reasonable health care. The identification of risk factors will allow us to target specific risk factors with appropriate interventions.

Maternal and Child Health

Abstract Code: 2017-NCD-370

Presenter Name: Dr. Alaa HamdAllah

Country: Jordan

Presenter Email: dr.alaa.hodib@gmail.com

Title Rate and Causes of Cesarean Section in North of Jordan

Background WHO recommends that the rate of cesarean section (CS) should not exceed 10-15% in any country. In recent years, the rate of cesarean deliveries increased dramatically worldwide with many countries had exceeded the WHO recommended rate. One study in Jordanian University Teaching hospitals showed that the rate of CS increased from 18.2% in 2002 to 30.3% in 2012.

Purpose Determine the rate and causes of cesarean deliveries in north of Jordan.

Methodology A prospective hospital-based longitudinal study was conducted. Women were enrolled in the study after delivery. All women who gave birth (dead or alive) at 20 weeks of gestation or more in four selected hospitals were eligible for inclusion. Necessary data for mothers were gathered through face-to-face interview using a semi-structured questionnaire and by abstraction of data from medical records. Information on causes of CS were confirmed by physicians.

Results The overall rate of CS was 37.5% (16.3% for emergency CS and 21.2% for planned CS) among Jordanian women. The rate of CS varied significantly according to health sector. The most frequent reason for planned CS was scarred uterus (50.0%). The second most common reason was multiple fetuses (20.8%). The most frequent reasons for emergency CS were prolonged fetal distress (33.5%) followed by obstructed labor (22.2%), abnormal presentation (13.1%), and eclampsia or sudden severe high blood pressure or seizure (6.3%).

Conclusions Jordan has a markedly high rate of CS. The rate of planned CS is higher than that of emergency CS. Scarred uterus and multiple fetuses are the most common reasons for planned CS. A multidisciplinary quality assurance program should be established in all Jordanian facilities in which delivery occurs.

Maternal and Child Health

Abstract Code: 2017-OTH-23

Presenter Name: Dr. Majed Hababeh

Country: Jordan

Presenter Email: m.hababeh@unrwa.org

Title Current Practices of Contraceptive Use Among Palestine Refugee Mothers of Young Children Attending UNRWA Clinics, A Follow Up Study 2015

Background UNRWA introduced family planning services in 1994 as an integral part of its expanded maternal and child health care program. The main objective of UNRWA's family planning program is to promote the health of mothers, children and subsequently their families.

Purpose The ultimate objective of this follow-up study was to assess the current situation regarding contraceptive practices among the target population after five years from the 2010 follow-up study and to identify future program needs leading to the development of action-oriented activities.

Methodology A cross-sectional survey was conducted by trained nurses from June - December 2015. Participants were Palestine refugee mothers who attended well-baby clinics with their youngest child (aged 2 months through 5 years) at all UNRWA health centers. A sample size of 10478 participants was calculated based on contraceptive use prevalence in 2010, using Epi Info sample size calculation. Mothers were interviewed and retrospective data from health records was used as supplement. All participants provided verbal informed consent. The study protocol approved and cleared by UNRWA health department ethical committee. A multiple logistic regression was performed to test if maternal age and parity predicted contraceptive use. Chi-square was used to analyze the relationship between previous contraceptive use and birth interval, birth weight, and gestational age.

Results Data was obtained from 9860 mothers, with a mean age of 29.8 years. Of them, 59.3% were using modern contraceptives at the time of the survey, 17.7% were using traditional methods, and 23.0% were not using any contraceptive method. The most common modern contraceptive was intrauterine device (37.4%), and UNRWA was the main provider for 82.6% of women currently using modern contraceptives. The most common reasons for not using contraceptives were child wish (21.7%), pregnancy (18.6%) and husband opposition (19.7%). Using women with <3 pregnancies as the reference category, women with 3-6 pregnancies are significantly more likely to use contraceptives ($p<0.001$; a OR=1.58, CI (1.43 – 1.73)), as are women with >6 pregnancies ($p<0.001$; a OR=1.6, CI (1.28 – 1.99)). Women with at least 1 male child are significantly more likely to use contraceptives ($p<0.001$; a OR=1.39, CI (1.24 – 1.56)). Maternal age over 35 was not a significant predictor for modern contraceptive use. Chi-square used to test the association of modern contraceptive use prior to pregnancy with birth weight, the result showed statistically significant 23.88 ($p<0.001$)] while gestational age was not a significant in the child born of that pregnancy.

Conclusions It is encouraging that mothers seeking modern contraceptives rely on UNRWA to provide family planning services. We found that mothers with higher parity are more likely to use modern contraceptives, which comply with UNRWA recommendations. As expected, modern contraceptives lead to better birth spacing. However, mothers above 35 years of age are not more likely to use modern contraceptives. These mothers may be at higher risk for negative maternal and infant health outcomes. UNRWA family planning services could focus more on counseling this group of mothers.

Maternal and Child Health

Abstract Code: 2017-EOH-242

Presenter Name: Dr. Ibidolapo Ijarotimi

Country: Nigeria

Presenter Email: dolapoijarotimi@gmail.com

Author: O. Fawole, A. Attahiru, M. Shakir, P. Nguku

Title HIV Status and Use of Contraceptives Among Women of Reproductive Age Group Attending the State Specialist Hospital Akure, 2017: A Comparative Study

Background Globally, in 2015 there were about 17.8 million women living with HIV (WLHA) and about 80% were in their reproductive age and lived in sub-Saharan Africa. WLHA are advised to limit family size and have access to the use of safe and effective contraceptive methods.

Purpose We investigated the association between HIV status and contraceptive use.

Methodology We conducted a cross-sectional comparative study in which we systematically sampled 500 HIV-positive and 500 HIV-negative women of reproductive age group at the Anti-retroviral and General outpatient clinics of the State Specialist Hospital, Akure. We collected data using interviewer-administered questionnaire. We calculated proportions, odds ratio (OR) and 95% confidence intervals (CI). We conducted multiple logistic regression with level of significance of 5%.

Results A total 469 (93.8%) WLHA and 465 (93%) HIV-negative women responded. Most of the WLHA (192, 40.8%) were in the age-group 35-44 years while most of the HIV-negative women (226, 48.7%) were in the age-group 25-34 years ($p<0.001$). Of the 363 (77.4%) WLHA at risk of getting pregnant, 224 (61.7%) were on contraception. Of the 241 (51.8%) HIV-negative women at risk of getting pregnant, 148 (67.2%) were on contraception. While 365 (77.8%) of WLHA used condoms, only 110 (30.1%) used it consistently and while 232 (49.9%) of HIV-negative women used condoms only 39 (16.6%) used them consistently ($p=0.008$). Though WLHA were 1.61 (CI: 1.24-2.08) times more likely to use any form of contraception than HIV-negative women and 3.53 (CI: 2.65-4.68) times more likely to use condoms than HIV-negative women. HIV status was not a predictor of current contraceptive use (AOR-1.33, CI: 0.68-2.60). The unmet need for contraception among WLHA was 17.1% and 14.8% among negative women ($p=0.700$).

Conclusions The contraceptive prevalence and condom use was low regardless of HIV status. There is need to intensify on interventions that increase contraceptive use among women with particular attention to WLHA.

Maternal and Child Health

Abstract Code: 2017-MCH-499
Presenter Name: Ms. Alice Namugamba
Country: Uganda
Presenter Email: alicegasuza@gmail.com
Author: R. Mangwi

Title Prevalence and Factors Associated with Domestic Violence During Pregnancy in Arua District, Uganda, 2015

Background Domestic violence during pregnancy is a serious public health challenge threatening maternal and fetal health outcomes. In Uganda, 16% of women experienced domestic abuse during pregnancy (UDHS 2011).

Purpose To investigate the prevalence and factors associated with domestic violence during pregnancy in Arua district so as to identify the magnitude of the problem, inform policy so as to protect pregnant women.

Methodology A cross-sectional study was conducted. Multi stage sampling technique was applied. Interviewer administered questionnaires were used. Binary and multi variable logistic regression analyses were carried out to identify strongest factors associated with domestic violence.

Results A total of 459 pregnant women were sampled. Prevalence of domestic violence during pregnancy was 48%. Emotional violence was the most prevalent form of violence (40%) followed by physical abuse contributing 29% and sexual violence 28%. Pregnant women reported husbands as their most perpetrators. Partner's alcohol consumption was the strongest risk factor associated with domestic violence during pregnancy (AOR 12.20 CI 2.25-65.92) followed by number of wives (AOR 2.16 C.I 1.08-4.32), wanting to be pregnant (AOR 0.26 C.I 0.14-0.48) and occupation too (AOR 2.22 C.I 1.12-4.42).

Conclusions Domestic violence against pregnant women was quite high. Almost five in ten women experienced domestic violence. Partner's alcohol consumption and number of wives were the strongest factors. Partner involvement during antenatal period is important. Increased attention to this vulnerable group is needed to improve maternal and child health. Antenatal care is known to be an important window of opportunity in providing support.

Maternal and Child Health

Abstract Code: 2017-OTH-15
Presenter Name: Ms. Yara Hadweh
Country: Palestine
Presenter Email: yara.hadweh@gmail.com

Title Palestinian Women Reproductive Health Rights: Knowledge, Opportunities, Challenges and the Way Forward. A Qualitative Study in Bethlehem Area in the West Bank.

Background Reproductive health rights (RHR) were declared as fundamental human rights since Cairo's (1994) and Beijing's (1995) conferences. The acknowledgement of the importance of these rights was a momentous point for reproductive health. In the Palestinian context, RHR are severely affected by the combination of constraints of the political system and the cultural context. RHR remain one of the least prioritized matters making them extremely unmentionable.

Purpose To underpin the multiple political-demographic and cultural factors/conditions that influence Palestinian women RHR.

Methodology This research relied on an empirical qualitative study. Semi-structured interviews were done through Skype. A purposive sample of ten Palestinian women living in Bethlehem Area in the West Bank was selected. These interviews formed the basis of the analysis.

Results Findings were considered under five emergent themes: knowledge, information and education; services and resources; Palestinian context and norms; conflict and occupation; violence against women. These intersecting themes shaped women's knowledge and perceptions of RHR. They highlighted the underpinning factors that influence the existing opportunities and challenges for Palestinian women to access to RHR, and health seeking behaviors and overall wellbeing.

Conclusions This research emphasizes the great importance of this topic to the field of public health in general and specifically women's health. This research is rare in its kind in the Palestinian context. In insuring universal access to RHR in Palestine, it is recommended that governments should demonstrate commitment to prioritizing RHR initiatives. Creating economic opportunities and socio-cultural empowerment for women in Palestine would build women's resilience. Further researches regarding RHR should be conducted in other areas of Palestine.

Maternal and Child Health

Abstract Code: 2017-OTH-142

Presenter Name: Dr. Abdul Wahhab Jewad
Country: Iraq
Presenter Email: alwehab1964@yahoo.com

Title Risk Factors of Preterm Newborns in Al-Zahraa Hospital /Al Najaf Al Ashraf Province, Iraq 2017

Background Preterm is a major determinant of infant mortality and morbidity. It is generally recognized that the etiology of preterm is multifactorial. In Iraq the preterm birth rate in 2010 is generally 6, 5% and deaths due to preterm birth 9%.

Purpose To determine the risk factors of preterm new-borns in Al Zahra Hospital.

Methodology A case control study was conducted Between December 2016 - February 2017 in Al Zahra hospital. All preterm newborns and double number of healthy controls were included in this study. Basic demographics and epidemiological data gathered from all cases and controls by direct interviewing the mothers of newborns, to identify potential risk factors. To assess the strength of association the odds ratio and 95% confidence interval of odds ratio was calculated.

Results The total number of cases was 97, about 62(64%) of cases were females. Majority of mothers 40(41%) were at age group 25-34 years and about 30(41%) of mothers had incomplete antenatal visits. Significant risk factors identified during analysis included female gender [OR=2.31(1.34-3.99)], Rh incompatibility [OR=3.04(1.33-6.91)], inadequate antenatal care [OR=2 (1.13-3.55)], antepartum hemorrhage [OR=3.37(1.60-6.95)], illiterate mothers [OR=1.87(1.01-3.43)], History of LBW [OR=11.1(5.46-22.57)], rural residency [OR=2.31(1.34-3.99)], history of abortion [OR=2.50 (1.47-4.25)], birth interval< 2 years[OR=1.77(1.01-2.99)], and extended family [OR=2.08(1.18-3.76)]

Conclusions Many risks for preterm newborn can be identified before pregnancy occurs. Health education, socio-economic development, and increasing the use of health services during pregnancy were recommended.

Maternal and Child Health

Abstract Code: 2017-FWBD-475

Presenter Name: Dr. Fadoua Oudrhiri
Country: Morocco
Presenter Email: f.oudrhiri@gmail.com
Author: A. Barkat, A. Khattabi, B. Assarag

Title Risk Factors for Preterm Birth in Morocco, 2017

Background Preterm birth (PTB) is a delivery that occurs before 37 weeks of gestation. It is the leading cause of newborn deaths in Morocco and worldwide.

Purpose The aims of our study were to identify the main risk factors of PTB and to propose measures to prevent and improve its management in our context.

Methodology We conducted case-control study in intensive care unit of neonatal medical service in Rabat university hospital considered as the inter-regional hospital. We included 87 preterm births before 37 gestations weeks and 174 term-controls. Data about the women's obstetrical and gynecological history, pregnancy complications and behavior during pregnancy was obtained using a structured questionnaire and medical records. The data was analyzed using SPSS version 20. The logistic regression was employed to identify risk factors of preterm birth.

Results The PTB incidence was 10.92%,The major factors leading to preterm labor were: herbal medicine use during pregnancy (OR adjusted = 20.23, IC: 5.39-75.8); Short interpregnancy intervals (OR adjusted = 14.62, IC: 2.75-77.5), history of preterm delivery (OR adjusted = 9.51, IC: 1.54-58.6); taking medicine during pregnancy (OR adjusted = 2.40 ,IC: 0.98-5.91), history of uterine curettage (OR adjusted = 7.97,IC: 1.63-38.8), having a twin pregnancy (OR adjusted = 8.57,IC: 1.95-37.7), maternal age less than 20 years old (OR adjusted = 8.32, IC: 1.59-43.5); primiparity (OR adjusted= 7.31,IC: 1.26-42.3); urogenital tract infection(OR adjusted = 6.63, IC: 2.37-18.4) and insufficient monitoring of pregnancy (OR adjusted = 2.78, IC:1.04-7.40).

Conclusions Mortality rates of newborn could be reduced if the incidence of prematurity decreases. Therefore, we should improve the prenatal care, the screening and early detection of pregnancies at risk for preterm birth, the screening of urogenital infections. Young women should be aware of risk behaviors during pregnancy.

Maternal and Child Health

Abstract Code: 2017-OTH-56
Presenter Name: Dr. Mouad Merabet
Country: Morocco
Presenter Email: meynet234@yahoo.fr
Author: A. Idrissi, M. Maassoumi, G. Bukassa, H. Tahri, S. Sedrati

Title Risk Factors and Causes of Neonatal Mortality at Al Hoceima Provincial Hospital (Morocco), 2016

Background In Morocco, the neonatal mortality rate is 21.7 / 1000 live births, which represents three quarters of the deaths of children under one year old. In 2015, 3.6% of intra-hospital neonatal deaths in Morocco were recorded in the province of Al Hoceima

Purpose The objective is to describe neonatal deaths and identify their risk factors at the Al Hoceima Provincial Hospital.

Methodology This is a retrospective case-control study at the neonatal unit of the Al Hoceima Provincial Hospital. The cases were the total of neonatal deaths in 2016. One control for each case was chosen randomly from the newborns admitted to the neonatology unit and taken out alive. The analysis of the data was carried out by Epi-Info 7.

Results The total number of newborns who died in 2016 was 81, with an intra-hospital neonatal mortality rate of 22.85 / 1000 live intra-hospital births. 88.89% of deaths occurred in the early period and 37.04% occurred in less than 24 hours. The main causes were prematurity (39.51%), respiratory distress (19.75%), and congenital malformations (14.81%). 6 factors significantly associated with neonatal death in multivariate analysis. 3 factors were poor prognosis: pregnancy at risk (ORadjusted = 9.70 [3.42, 27.50] 95%CI), birth weight <2500g (ORadjusted = 4.74 [1.88, 11.93] 95%CI), Apgar score at birth <7 (ORadjusted = 4.28 [1.62, 11.34]95%CI). And 3 factors were of good prognosis (ORadjusted = 0.07 [0.02, 0.27]95%CI), hospital delivery (ORadjusted = 0.09 [0.03, 0.29]95%CI), pregnancy followed in prenatal consultation (ORadjusted= 0.06, 0.45]95%CI).

Conclusions Neonatal mortality in the Al Hoceima hospital remains high and is mainly related to the course of pregnancy and childbirth as well as the characteristics of the newborn at birth. To this end, pregnancy monitoring, confinement in a supervised environment and integrated management of the mother-to-newborn couple are the key measures to reduce this real health problem.

Maternal and Child Health

Abstract Code: 2017-OTH-315
Presenter Name: Dr. Nora Alafif
Country: Saudi Arabia
Presenter Email: nalafeef@ksu.edu.sa

Title Worse or Just Different? Self-reported Sleep Characteristics of Pregnant and Non-Pregnant Women in the UK Household Longitudinal Study

Background The American Academy of Sleep Medicine include 'pregnancy-associated sleep disorder' (PASD) in its International Classification of Sleep Disorders. PASD is indicated by: either frequent arousals and reduced sleep efficiency or a prolonged habitual sleep duration; and habitual sleep latencies of <10 min.

Purpose The present study aimed to assess whether pregnant women sleep differently to non-pregnant women, and if so whether any differences might reflect underlying PASD.

Methodology Methods Data on self-reported sleep characteristics (duration [hours]; latency, disturbance, coughing/snoring, medication and daytime sleepiness [frequency]; and quality [very good, fairly good, fairly bad, very bad]) from Waves 1 and 4 of the UK Household Longitudinal Study were available for n = 792 pregnant and n = 9,965 age-matched non-pregnant women with complete data on seven potential confounders (age, ethnicity, educational, marital status, employment, parity and household structure). Multinomial logistic regression analyses were conducted before and after adjustment for confounding using STATA IC14 using the least unfavorable response as the referent category.

Results Pregnant women were more likely to sleep both shorter and longer than 7–9 hrs (RRR = 1.22–1.45), and they were also more likely to struggle to get to sleep within 30 min on a regular basis (RRR = 1.07–1.28). Although pregnant women reported more frequent sleep disturbance than non-pregnant women (RRR = 1.35–3.72), they did not report coughing/snoring more frequently (RRR = 0.89–1.20). Likewise, despite reporting poorer sleep quality than non-pregnant women (RRR = 1.60–2.21), pregnant women were less likely to report using sleep medication on a regular basis (RRR = 0.44–0.55). Finally, pregnant women were more likely to report difficulty staying awake during the day than non-pregnant (RRR = 1.28–1.77).

Conclusions The findings of the present study confirm that pregnant women sleep differently to non-pregnant women, although these differences may partly reflect the lower use of medication by pregnant women.

Maternal and Child Health

Abstract Code: 2017-OTH-274
Presenter Name: Dr. Amr Torbosh
Country: Yemen
Presenter Email: amrtorbosh@gmail.com
Author: M. Alemad

Title Impact of the 2015 Yemeni War on the Under One Year Children Immunization Coverage

Background After two-years of the war that have crippled the capacity of the Yemeni National Health System and left only 45% of health facilities are functioning, Yemen faced increasing vaccine preventable diseases (VPD) outbreaks and may become at high risk of polio importation.

Purpose To document the impact of the 2015 war on under one-year Yemeni children immunization coverage.

Methodology Data on vaccination coverage for the 2012-2015 was obtained from the National Expand Program for Immunization (EPI). The vaccination coverage was calculated at national and governorate levels through dividing the number of the actually vaccinated children by the estimated under one-year children population.

Results Although there is an increase from 2012 to 2014 in the national coverage with Penta 3 (82 vs. 88%) and measles (70% vs. 75% respectively), the coverage was still below the national target (= 90%). Furthermore, the 2015 witnessed marked drop in national coverage compared to 2014 for measles (66% vs. 75%) and BCG (49% vs. 73%) but slight drop in coverage by Penta 3 (84% vs. 88%). Such drop was more marked at the governorates that witness armed confrontations e.g. Taiz governorate showed drop in Penta 3 coverage from 93% in 2014 to 73% in 2015 and Sa`dah from 50% to 38%. On the other sides, governorates that did not witness armed confirmations showed increase in coverage e.g. Rima showed increase in Penta 3 coverage from 87% to 106% for the same period.

Conclusions The analysis shows the marked negative impact of the 2015 war on immunization coverage especially in the governorates that witness armed confrontations that may put Yemen at more risk for polio importation and VPD outbreaks. Besides the ongoing struggles to stop the Yemeni war, more innovative vaccine delivery/provision and increasing demands strategies are needed especially in governorates with confrontations.

Maternal and Child Health

Abstract Code: 2017-HIV/STI-429
Presenter Name: Dr. Hasanein Malik
Country: Iraq
Presenter Email: drhasanein@yahoo.com
Author: F. Lami

Title Malnutrition Among Under Five Children in Iraq, 2002- 2016

Background In Iraq, the long-standing war and civil unrest had negatively affected food security, water quality, sanitation and environmental conditions that contributed to decline in the nutritional and health status of children.

Purpose The objective of this study is to assess the trend of different types of malnutrition among <5years children in Iraq, 2002-2016.

Methodology We used data from four Multiple Indicator Cluster Surveys (MICS 2, 3, 4 and 5). Three WHO malnutrition indicators were assessed: stunted growth (z-score of height for age = -2.01), underweight (z-score of weight for age =-2.01) and wasting (z-score of weight for height = -2.01).

Results The prevalence of stunting decreased from 24.2% in 2002 to 16.6% in 2016. Severe stunting was 8.7% in 2002 and 6.7% in 2016. Male: female ratio was 1:1 in 2002 and 1:1.3 in 2016. The highest prevalence was among children aged 12-23 months in 2002 (28.9%) and among those aged 48-59 months (30.1%) in 2016. The prevalence of underweight decreased from 16.9% in 2002 to 5.9% in 2016. Severe underweight was 2.7% in 2002 and 1.7% in 2016. Male: female ratio was 1.1:1 in 2002 and 1.4:1 in 2016. The highest prevalence was among children aged 12-23 months in 2002 (24.4%), while in 2016, it was among children aged 0-11 months (24.8%). The prevalence of wasting had slightly increased from 7.3% in 2002 to 7.8% in 2016. Male: female ratio was 1.2:1 in 2002 and 1.3:1 in 2016. The highest prevalence was among children aged 12-23 months in 2002 (10.0%), while in 2016, it was among children aged 0-11 months (13.0%).

Conclusions In spite of the tremendous challenges that faced Iraq during 2002-2016, the decline in the prevalence of stunting and underweight reflects better-provided health services. All types of malnutrition are still there and intensified multidisciplinary efforts are recommended.

Non-communicable Diseases

Abstract Code: 2017-FWBD-473
Presenter Name: Dr. Reham Kamel
Country: Egypt
Presenter Email: rehamkamel1986@ymail.com
Author: S. Refaey, H. Abu El-Sood, A. Mohsen

Title Prevalence and Risk Factors for Early Childhood Caries in Children Less Than 6 Years Old: A Systematic Review

Background Early childhood caries (ECC) is a very common multifactorial bacterial infection. It negatively impacts children's psychological status and quality of life. Therefore, the World Health Organization (WHO) global oral health goals for 2000 were established stating that 50% of 5–6-year olds would be free of dental caries.

Purpose To describe ECC prevalence and severity among children less than 6 years old in Egypt and compare it with other countries in other regions, to investigate risk factors associated with ECC, and to determine areas for future research.

Methodology The search included published studies of any design in PubMed database, and Google scholar with the key words "dental caries, prevalence, children, childhood, risk factors". The inclusion criteria required that selected children should have deciduous dentition and the method used for caries assessment should be either decayed, missed/extracted, or filled tooth index, or decayed, missing, and filled surface index for children less than 6 years old.

Results The search identified initially 300 articles of which 22 studies published between 2001 and 2017 were included in the review (19 cross sectional, two case control and one interventional). The prevalence of ECC in the reviewed studies ranged widely (16% - 89%). The median prevalence was highest 76% (IQR: 72.5%, 82.5%) in the Middle East Region followed by Asia which was 48.3% (IQR: 32%, 52%) while it was observed to be the least (20.7% - 24.9%) in developed countries. However, in Egypt, it was 66.8% (IQR: 63%, 71%). Some factors were statistically significant with ECC development as age (6 out of 11 studies), parental education and sugar intake (5 out of 6 studies) while gender was not statistically significant (11 out of 14 studies).

Conclusions WHO global target is still far away in Egypt. Enhancing community oral health programs is necessitated to address the gaps and leading initiatives of preventive actions.

Non-communicable Diseases

Abstract Code: 2017-FWBD-318
Presenter Name: Dr. Fatima Zerriouh
Country: Jordan
Presenter Email: toom832016@gmail.com
Author: Y. Khader

Title Diabetes in Jordan: Prevalence, Trend, Awareness and Control

Background Studies have reported an increasing prevalence of type 2 diabetes Mellitus (DM), with the most dramatic increase occurring in developing countries.

Purpose This study aimed to determine the prevalence of type 2 DM and impaired fasting glycemia (IFG), assess the trend, awareness and state of control of type 2 DM in Jordan.

Methodology Data were analyzed from a cross-sectional study in 2017 that included a random sample of 4056 Jordanians aged 25 years and above. DM and IFG were diagnosed according to American Diabetes Association definition. HbA1c >7.5% was defined as unsatisfactory metabolic control.

Results This study included a total of 4056 persons (70.5% females). The age-standardized prevalence rate of IFG was 21.6% among males and 19.2% among females and that for DM was 28.3% (95% CI: 25.5, 31.1) among males and 19.9% (95% CI: 18.1, 21.6) among females. The prevalence rate of DM increased significantly with increasing age peaking at age of 60-64 for both males (58.7%) and females (53.1%). In consecutive surveys (1994, 2004, 2007, and 2017) that adopted the same methodology, the aged-standardized rate of DM increased from 13% in 1994 to 17.1 in 2004 to 13.4 in 2007 to 22.3 in 2017. Of the 888 diabetic subjects, 768 (86.5%) had been previously diagnosed and 13.5% were diagnosed by the study team. Of the 768 patients who were previously diagnosed, 699 (91.0%) were on treatment; of whom 212 (30.0%) had good glycemic control.

Conclusions The prevalence of type 2 diabetes and IFG is high in Jordan and is increasing. More than two thirds of patients had diabetes with unsatisfactory control. Therefore, they are likely to benefit from programs aimed at encouraging behaviors toward achieving optimum weight as well as physical activity behaviors. Physicians caring for patients with diabetes may need to adopt a more vigorous approach for diabetes control.

Non-communicable Diseases

Abstract Code: 2017-FWBD-52

Presenter Name: Dr. Mohammad AlMazroa

Country: Saudi Arabia

Presenter Email: maalmazroa@hotmail.com

Title Cost of Diabetes in Saudi Arabia

Background The Kingdom of Saudi Arabia (KSA) has made tremendous improvements in its health system in a short period of time due to extensive investments from oil revenues. In 2015, the Saudi Ministry of Health (MOH) housed 250,000 personnel, including 31,516 physicians and 75,978 nurses, and operated 249 hospitals with 34,000 beds .It is responsible for 60% of healthcare services, with the remaining 40% managed by a number of semi-public organizations and the private sector .The MOH is in charge of health promotion, disease prevention, and treatment. Health care services are provided for free throughout the country. In recent years and with improvements in infrastructure and health services, an increasing burden of non-communicable diseases is emerging. We recently reported high rates of diabetes mellitus.

Purpose Diabetes mellitus is a major burden in the Kingdom of Saudi Arabia (KSA). We estimated the direct cost of diabetes in KSA and the future cost accounting for currently undiagnosed and borderline diabetics. Methods: We used a bottom-up approach to determine the direct cost of diabetes mellitus in KSA at the population level using Saudi Ministry of Health (MOH) cost data for medications, health visits, laboratories, and hospitals.

Methodology KSA MOH, developed a database of available published and unpublished data sources to generate the burden of disease for KSA. Key inputs into this database included systematic reviews of the literature, analysis of household survey data, antenatal clinic surveillance, reportable disease notifications, disease registries, hospital admissions data, outpatient visit data, population-based cancer registries, active screening data, and other administrative data. Moreover, the MOH provided detailed data on the prices of medications, laboratory testing, medical procedures, and clinic and hospital visits and stays. We assumed that the MOH cost estimates apply to the population served by the private sector and other governmental health agencies such as the armed forces or the National Guard. Blood samples were collected at local health clinics and analyzed in a central lab at the King Fahd Medical City in Riyadh. COBAS INTEGRA400 plus was used to measure blood levels of HbA1c, or glycated hemoglobin. A Roche Hitachi COBAS 8000 system was used to measure cholesterol. Respondents were considered to be diabetic if they met any of the following criteria: 1) measured HbA1c equals or exceeds 6.5% (48.5 mmol/mol), or 2) measured HbA1c does not equal or exceed 6.5% (48.5 mmol/mol), but the respondent reported taking medications for diabetes. Respondents were considered to be have borderline diabetes (glucose intolerant) if: 1) they did not report taking drugs for diabetes, and 2) their measured HbA1c blood level was greater than 5.7% (35.3 mmol/mol) and less than 6.5% (48.5 mmol/mol). Women were asked if they were diagnosed with diabetes during pregnancy. Respondents were considered hypercholesterolemic if they met any of the following criteria: (1) measured cholesterol equal to or exceeding 6.2 mmol/L or measured cholesterol not exceeding 6.2 mmol/L, but the respondent reported taking medications for hypercholesterolemia.

Results Overall, 1,095,776 (8.5%) Saudis reported being diagnosed with diabetes. However, a total of 1,745,532 (13.4%) Saudis aged 15 years or older had diabetes. This total group is the sum of measured diabetes (1,193,075, 68.4%) and those who were currently on diabetes medication with controlled levels of HbA1c (552,457, 31.6%). Among those that our survey identified as diabetic from blood tests, 43.6% were undiagnosed. Moreover, 15.2% of Saudis, or 979,953, had borderline diabetes. the total cost of diabetes in KSA. The cost currently is at 17 billion Riyals. If those who are undiagnosed joined the treatment pool, the cost would increase to 27 billion Riyals. If those with glucose intolerance (pre-diabetes) progressed at the current observed rate, the total cost would be 43 billion Riyals.

Conclusions Our study calls for immediate action to reduce the burden of diabetes and non-communicable diseases (NCD). A national plan to prevent and control the rising burden of chronic diseases should be developed and implemented. We previously reported that Saudis do not seek preventive care. Hence, it is crucial that the programs involve community engagement and early screening. Campaigns to educate the public about the disease should be a priority. The campaigns should include information on the risk factors for diabetes and gestational diabetes. the total cost of diabetes in KSA. The cost currently is at 17 billion Riyals.

Non-communicable Diseases

Abstract Code: 2017-FWBD-309
Presenter Name: Dr. Hejer Letaief
Country: Tunisia
Presenter Email: hejerletaief@gmail.com
Author: A. Hechaichi, S. Chelly, H. Bouguerra, S. Rejaibi, F. Saffar, A. Cherif, M. Chahed

Title Determinants of Cervical Cancer Screening in Tunisia: A Cross Sectional Study Among 1494 Women Aged Between 18 and 65 Years of Age

Background In low- and middle-income countries, cytology-based programs are very difficult to implement, and where they are implemented, the screening coverage is low. Routine screening of cervical cytology has been implemented in Tunisia in order to decrease cervical cancer incidence and mortality. Understanding the factors associated with cervical cancer screening among target populations is important to improve the screening participation rate. In Tunisia, few studies have examined cancer screening among women in relation to socio demographic and socioeconomic status.

Purpose To estimate the coverage of women by cervical cancer testing and to assess the relation with sociodemographic and socioeconomic determinants.

Methodology A cross-sectional survey was conducted in 2014 and face-to-face interview questionnaires were completed by a sample of 1494 women aged 18 years and older.

Results Only 36.6% (95% CI [34.3%-39.2%]) of 1494 women aged =18 years had received a previous cervical screening test. This rate increased significantly (p<10-3) with age from 13.9% for those aged less than 30 years to 29.1% for those aged between 30 and 40 years, to 47.9% for women aged 40-50 years old and 49.3% for those aged more than 50 years. In multivariate, we found previous cervical cancer screening was significantly associated with household income, occupation, level of education, tobacco use, medical previous history and familial history of cancer, at risk sexual behavior and history of sexual transmitted diseases.

Conclusions The coverage of women population with cervical cancer screening was found to be very poor. Analyses of cancer screening rates by measures of income, educational level, and other factors may help to implement a comprehensive, integrated approach across different health programs. Given the importance of knowledge in encouraging women to participate in screening is key to reducing cervical cancer burden in Tunisia. A health promotion intervention should be developed and implemented in project counties focusing on improving their knowledge.

Non-communicable Diseases

Abstract Code: 2017-FWBD-113
Presenter Name: Dr. Liana Haddad
Country: Palestine
Presenter Email: beitjala2000@yahoo.com

Title Knowledge, Attitude and Practice of Breast Self-Examination Among Female's Governmental School Teachers in Bethlehem District – Palestine, 2015

Background Breast Self-Examination (BSE) is an important public health screening principle. It is a no cost and easy process whereby women examine their breasts regularly to detect any abnormal swelling or lumps in order to seek if needed a medical aid. According to Palestinian Ministry of Health Annual Report 2014, breast cancer is the first cause of female's death among the other cancer causes and most of the cases had been discovered at late and sever stages and 58.9% of them are (15 – 64 years). Bethlehem district stays in the third place among the other districts with cancer incidence rate 111.2 per 100,000 of population (103,550 Females, 106,934 Males).

Purpose The aim of the study is to evaluate the level of knowledge and practice of BSE among females' governmental school teachers in Bethlehem District.

Methodology A cross sectional study using a structured, self-completed and pre-tested questionnaire designed by the researcher has been used in 2015 in seven schools that randomly selected. A written informed consent from the participant and permission from the minister of education was obtained.

Results From what is shown, the majority of the teachers were aware of BSE with percentage 97,3% and 76,9% of them only knew the procedures of BSE and actually 47,6% of them do not make BSE periodically. The study also showed that 57.1% of the respondents knew about the breast cancer from the media, followed by 25, 2% from health workers; in addition, the study revealed 77.6% are aware about the symptoms of breast cancer, while 81.6% showed that is BSE is not enough to diagnose breast cancer.

Conclusions Awareness of BSE was high, but the practice was low. It is recommended that public awareness of the importance of BSE be intensified using mass media. And health worker should promote BSE during their contact with clients.

Non-communicable Diseases

Abstract Code: 2017-MCH-217
Presenter Name: Dr. Khitam Ali
Country: Iraq
Presenter Email: dr_khetam@yahoo.com
Author: A. Raddi, F. Lami

Title Fatal Domestic Injuries in Iraq, 2010-2015

Background Home injuries are leading causes of death and disability worldwide. About 5.8 million people die from injuries each year around the world. This accounts for 9% of the world's deaths. About one third of all injuries happen at home.

Purpose This study was conducted to describe the epidemiological characteristics, estimate incidence and identify mechanism of fatal domestic injuries (FDI), Iraq, 2010-2015.

Methodology We conducted this cross-sectional study on all fatalities due to injuries reported from all the coroner offices in Iraq for the period of 2010-2015 as part of Iraqi Injury Surveillance System. The surveillance form included detailed data on the demographics, injury circumstances and injury mechanisms. National and governorates population data were obtained from Ministry of Planning.

Results Total number of FDI during 2010-2015 was 17,211 with an average annual incidence of 11/100,000Population (P). FDI represented 24.5% of total fatal injuries. The incidence of FDI decreased from 11.6/100,000P in 2010 to 10.2/100,000P in 2015. Female to male ratio was 1.1:1 and 38.8% occurred at the age group 20-39 years. The highest incidence 27.5/100,000P was recorded at the age group =70 years. About 71.5% were unintentional with average annual incidence 7.7 /100,000P and the remaining were intentional with average annual incidence of 1.6/100,000P. About 61% of FDI occurred during 7am-4pm. Main causes of FDI were: Burn (44.5%), electric injury (21.8%), gun fire (7.8%), and fall (4.9%) with average annual incidence rate of 4.9, 2.3, 0.8, and 0.4 per 100,000P, respectively. The highest average incidence was reported in Misan (19.4/100,000P), and Erbil (16.6/100,000P) governorates, while the lowest was reported in Najaf (3.4/100,000P) governorate.

Conclusions In Iraq, FDI constituted a considerable public health problem. Public Health officials need to develop a comprehensive program to control home injuries particularly burn and electrical injuries and ensure that people have safe and healthy homes.

Non-communicable Diseases

Abstract Code: 2017-MCH-203
Presenter Name: Dr. Fahad Al-Jasser
Country: Saudi Arabia
Presenter Email: dr.f.j@hotmail.com
Author: A. Gad Mohamed, A. Choudhry, R. Youssef

Title Use of Mobile Phone During Driving and the Risk of Collision Among Preparatory Year Students in King Saud University, Riyadh, 2014

Background Mobile use while driving is a major cause of road traffic injuries.

Purpose To determine the rate of mobile use while driving among King Saud University students, their perception of the risks and its contribution to collisions.

Methodology A cross-sectional study was conducted in May 2014 targeting 986 male students of King Saud University. A questionnaire was used to obtain data on possessing a driving license, years of driving, driving hours, and collision or near collision in the six months preceding the study. Eight statements were used to assess the behavior and perceptions related to the use of mobile phones while driving. Data were analyzed using the chi-square statistic, odds ratio (OR) and the 95% confidence interval (95%CI).

Results Half the participants (45.3%) have a driving experience of 4 – 6 years and 18.3% of them did not have a driving license. Collision in the preceding six months was reported by 44.6% of participants and 37.9% of them attributed their occurrence to mobile phones. Variable proportions reported always texting (53.3%) or talking in hand-held (66.2%) or hand-free (26.1%) phones while driving. A significantly higher risk of collision was observed among participants who reported always talking on mobile phones while driving as hand-held (OR=1.435) and hand-free (OR=1.469) as well as sending or receiving text messages (OR=1.885). The risk increased significantly from 2.052 among participants who reported driving daily for 1 – 2 hours to 3.165 among those who reported driving for more than 6 hours.

Conclusions The risk of collision exists with the use of hand-held and hands-free mobile phones. As hands-free mobile phones are not safer, national legislation should consider their restriction during driving and implementation of the legislations that ensure safety on the road should be reinforced. The objective assessment of the contribution of mobile phones to road traffic injuries is recommended.

Non-communicable Diseases

Abstract Code: 2017-OTH-471
Presenter Name: Dr. Abdulaal Chitheer
Country: Iraq
Presenter Email: abdulaaljetheer@yahoo.com

Title What Causes the Most Death and Disability in Iraq, Findings from GBD Study 2016?

Background Global Burden of Disease study (GBD) is a systematic, scientific effort to quantify the comparative magnitude of health loss from all major diseases, injuries, and risk factors for all ages, sexes, and geographies, and over time. It provides a comprehensive assessment of all-cause and cause-specific mortality for 249 causes.

Purpose To report the most common causes of death and disability in Iraq, 2016

Methodology We used the 2016 GBD study for estimates of mortality and disability-adjusted life years (DALYs) of different cause of deaths and disabilities for Iraq available GBD result tool. Each value has a mean and a 95% uncertainty interval which means a range of values that include the correct estimate. All estimates were rates per 100,000 and age standardized. Percentages of change from 1990 to 2016 were calculated.

Results Age-standardized mortality rate for all causes was 1,209 per 100,000 (1,026-1,385); NCDs accounted for 989 (835-1,141) with 6% decline from that of 1990, injury deaths increased by 61% to be second cause while neonatal/maternal/nutritional diseases declined by 46%. Cardiovascular diseases (CVD) was top cause of death; 613 (517-703) making 34% of all deaths, followed by cancers; 99 (80-117) which is increased by 4%. Age-standardized DALY rate for all causes was 44,138 per 100,000 (37,736-50,736). From which, NCDs accounted for 30,152 (25,905-34,708) with 6% decline from that of 1990, injury related DALYs was 8,546 (5,499-12,110) with 42% positive change, while neonatal/maternal/nutritional diseases declined by 44%. CVD was top cause of DALY; making 14% of total DALYs, followed by war/disasters; 4,255 (1,232-7,544) with 12% increase.

Conclusions Major disease burden in Iraq made by NCDs, mainly CVDs with accountable rise in cancer and injury burden. About half of Iraqi people are disabled or prematurely died by preventable conditions. Sound preventive measures should be established, and decisions based on global estimates may enhance redirecting health policies.

Outbreak Investigation/FWBD (Foodborne)

Abstract Code: 2017-OTH-10
Presenter Name: Dr. Kashif Ur Rehman Khalil
Country: Pakistan
Presenter Email: dr.kashif.khalil@gmail.com
Author: N. Mehsood, M. Faisal

Title Estimation of Heavy Metals in Branded and Local Snacks in the Markets of Peshawar, Khyber Pakhtunkhwa, Pakistan

Background Heavy metals in food is emerging public health problem. They are defined as elements that have high atomic weight as compared to water and a minimum density of 5 times greater than that of water. They are of concern because of their adverse effects on individual health.

Purpose To estimate the concentration of heavy metals (Lead, cadmium, Chromium and copper) in branded and non-branded (local) snacks in the markets of Peshawar, Khyber Pakhtunkhwa, Pakistan.

Methodology A total of 96 different samples of potato and corn snacks were selected from four towns of district Peshawar by convenient sampling technique. All samples were subjected to wet digestion using acid digestion technique and Lead, Cadmium, Chromium and copper were quantitatively detected, using Atomic Absorption spectrometer in the Public Health laboratory Khyber Medical College Peshawar.

Results Mean concentration of Lead, cadmium, chromium and copper in potato based branded snacks were in the range of 0.085-0.423mg/kg, 0.003-0.046 mg/kg, 2.186-2.328 mg/kg and 0.008-0.108 mg/kg and concentration of heavy metals in corn based branded snacks were in range of 0.240-0.351 mg/kg, 0.007-0.012mg/kg, 2.254-2.179 mg/kg, 0.030-0.082 mg/kg, respectively. While mean concentration of Lead, cadmium, chromium and copper in non-branded (local) snacks were 0.057-0.324 mg/kg, 0.005-0.012 mg/kg, 2.137-2.247 mg/kg and 0.018-0.06 mg/kg, respectively.

Conclusions Chromium were exceeding in majority samples of branded and local snacks.

Outbreak Investigation/FWBD (Foodborne)

Abstract Code: 2017-EOH-218
Presenter: Dr. Kamal Kadhim
Country: Iraq
Presenter Email: kamalabdrazaq@yahoo.com
Author: K. Fathallah, Al Jaafar, A. Hamad, S. Saadoon, Z. Awad, F. Lami

Title A Food Poisoning Outbreak in Al-Khazir U2 Camp of Internally Displaced Persons - Iraq, Summer 2017

Background Globally, about 600 million fall ill and 420,000 die every year after eating contaminated food. On June 12, 2017, Iraq CDC informed about a food poisoning outbreak among internally displaced persons (IDPs) in Al-Khazir camp, Northern Iraq after consuming "Iftar" (Ramadan breakfast) provided by an NGO. "Iftar" was composed of rice, white bean broth, chicken, yogurt, date and bread.

Purpose The objective was to describe the outbreak, identify the causative agent and recommend preventive measures.

Methodology A team of FETP residents dispatched to investigate the outbreak. The case patient was defined as any person in the camp who ate "Iftar" and developed nausea/vomiting or diarrhea or abdominal pain. A retrospective cohort study was initiated.

Results The total number of IDPs in the camp was 6,425; 1,470 (22.9%) were in sections C, D and E who got "Iftar" and all were directly interviewed. The mean age of patients was 19 (±15years), and the female: male ratio was 1.1:1. The attack rate was 91%. The most frequent symptoms were nausea and vomiting (86.6%), abdominal pain (68.4%) and diarrhea (59%). The range of incubation period was 15 minutes-16 hours; 62.2% had symptoms after three hours. About 72% of patients treated in the nearby PHCs and 28% transferred to the ER. None of the patients was hospitalized or died. The implicated food items were: rice (RR=1.4; 95%CI: 1.2-1.6) and chicken (RR=1.2; 95%CI: 1.1-1.3). Laboratory results of examined leftover food samples revealed Staphylococcus aureus growth. All meals were prepared by one restaurant, not kept in refrigerators and served after 10 hrs. For legal causes, the team was not allowed to assess the restaurant or examine the workers.

Conclusions The large number of ordered meals from a single restaurant led to suboptimal food preparation and storage. We recommended rigorous implementation of food handing instructions and health education of the food handlers.

Outbreak Investigation/FWBD (Foodborne)

Abstract Code: 2017-EOH-249
Presenter Name: Dr. Mohamed Gouda
Country: Egypt
Presenter Email: gouda.pharmacist88@gmail.com
Author: M. El Ghazaly, S. Samy, S. Refaey, A. Kandiel

Title Outbreak Investigation of Unexplained Abdominal Pain, Port Said Governorate, Egypt October 2017

Background On 12 October 2017, three cases of unexplained abdominal pain including one death after eating fresh unknown plant were reported to epidemiology and surveillance unit at directorate health affairs.

Purpose Investigation was done to confirm the outbreak, identify the risk factors, and provide control measures.

Methodology Outbreak investigation team defined a case as abdominal pain, diarrhea and vomiting. Active case finding was done and 3 cases were detected. One case died, and a questionnaire was filled for the two alive cases by surveillance team. One stool sample was collected for microbiological testing. The team collected water sample from occupation place for microbiological testing. The team collected samples from the unknown mushroom.

Results The dead case consumed the mushroom 1 pm and the case deteriorated rapidly and died at 4.30 pm with acute hepatic and renal failure. Symptoms emerged in one case at 7 pm and active case finding was done for the third case which was hospitalized at 10 pm. Two alive cases had moderate symptoms. No more cases were detected. Age range was 43-55. The 3 cases were male workers in a resort. All sick cases consumed mushroom. The symptoms revealed were 100% vomiting, 100% diarrhea, 100% dyspnea, 33.3% malaise, 100% abdominal pain and 66.67% bitter tongue. Results of water and stool samples were negative microbiologically. The mushroom type was called the death cap (Amanita phalloides).

Conclusions The outbreak was associated with consuming fresh collected death cap mushroom which is hepatotoxic. Control measures were taken to remove all implanted mushroom in the grass of resort and making announcement that there is a poisonous mushroom implanted in the grass. Follow up done for more cases.

Outbreak Investigation/FWBD (Foodborne)

Abstract Code: 2017-EOH-472
Presenter Name: Dr. Zaina Khreisat
Country: Jordan
Presenter Email: dr.zeina@hotmail.com
Author: S. Alqasrawi, M. Abdallat

Title Food Poisoning in the Town of Hatem, Irbid (Jordan)

Background On 14 September 2017, one hundred and one residents in the town of Hatem in Irbid (Jordan) were admitted to local healthcare center and a city hospital in Irbid with symptoms of diarrhea, vomiting, fever, and abdominal pain after eating a meal from a nearby restaurant.

Purpose Identify the cause and mode of transmission and to implement control measures.

Methodology The food poisoning outbreak was described by time, person, and place. A case was defined as any person with two or more of the following symptoms: diarrhea, vomiting, fever, and abdominal pain, who had been in the town of Hatem (Irbid) in the period 14-19 September 2017. We conducted a cohort study and collected data on demographics, water sources, and food sources. We conducted environmental survey for water sources and for food in local restaurants for fecal contamination, and tested water, food, and stool samples for microorganisms.

Results Among 8,000 residents, we identified 101 food poisoning cases. Outbreak was during 14-19 September 2017 with peak (73 cases) on 17 September 2017. Attack rate was the highest among residents aged 10-19 years (47 cases) and residents aged 5-9 years (33 cases). Females were more affected than males (58% vs. 42%). The food poisoning outbreak was associated with taking 'Hommos" meal and 'Falafel' from a local restaurant. The stool samples of patients and the restaurant owner were positive for Shigella sonnei and Escherechia col. The ground 'Hommos' samples taken from the local restaurant were positive for Shigella sonnei.

Conclusions The food poisoning outbreak in the town of Hatem in Irbid (Jordan) was associated with eating 'Hommos" meal and 'Falafel' from a local restaurant contaminated with Shigella sonnei. We recommend periodic inspection and active monitoring of water and food, as well as of the caterers, across the county to reduce the likelihood of such outbreaks.

Outbreak Investigation/FWBD (Foodborne)

Abstract Code: 2017-EOH-121
Presenter Name: Dr. Moumni Abdou Houda
Country: Morocco
Presenter Email: houdamoumnni@gmail.com
Author: D. Ilhame

Title Investigation of a Collective Foodborne Illness Shows a Multi-Pathogen Contamination in Training Institute in Rabat, June 2017, Morocco – A Case Control Study

Background Foodborne diseases outbreaks are common worldwide and an important public health issue. According to WHO, approximatively 600 million of foodborne illness and 400 thousand related deaths per year. In Morocco, an average of 1500 cases is notified annually. On June 18, 2017, the public health service was alerted about 43 suspected students in a training Institute in Rabat admitted in emergency for collective foodborne disease following an uptake of meal a day before.

Purpose We investigated this outbreak to confirm the outbreak, to identify the source of contamination and to recommend control measures.

Methodology We conducted a case control study. We selected cases and controls in the ratio of 1:1. We defined a case as anyone who attended the meal served on 17 June 2017 at the Institute's restaurant and presented in the two days after diarrhea and one of the following signs: nausea, vomiting, abdominal pain, fever, and dizziness. A control was defined as anyone who had consumed the same meal and had not presented any digestive signs. We conducted bivariate and multivariable analysis. Stools of ill students were collected, and food specimen was collected for bacterial testing.

Results Among 100 students interviewed, we identified 50 cases. Among cases, males were predominant (86%), the median age was 21 years. 47 sought medical care. The episode was short with an estimated average incubation period of 9 hours. The epidemic curve oriented towards a common source of contamination. Among food items, Briwates were strongly associated with the illness with an odd ratio of 14.23 (5.04-40.04). Laboratory testing of Briwates found EColi O157 and Staphylococcus Aureus.

Conclusions This was an outbreak associated with the consumption of Briwates contaminated by two germs, which was the source of this episode. We recommended to strengthen hygiene measures and safe food handling, to raise awareness and educate the staff.

Outbreak Investigation/FWBD (Foodborne)

Abstract Code: 2017-FWBD-343

Presenter Name: Dr. Turki Alaslani
Country: Saudi Arabia
Presenter Email: dr_turki2015@hotmail.com

Title Outbreak of Foodborne Disease in Hajj Camp During Hajj Season 2017

Background On 30th August 2017, the Food Safety Department of Saudi Ministry of Health received notification of potential outbreak of foodborne illness among Hajj pilgrims. Residents from the Saudi Field Epidemiology Training Program team and public health officials investigated the outbreak to identify the source and prevent additional illnesses.

Purpose To investigate the outbreak and identify the main source of foodborne illness.

Methodology A case control study was conducted, with a case definition as any pilgrims who ate lunch meal at the Fajr Al Eman Hotel on Wednesday 30th August 2017 and experienced abdominal pain, diarrhea, vomiting and fever. The ratio of cases to control was 2:1. A standardized questionnaire was used to collect information on symptoms, onset of illness, and food history.

Results In this study we identified 30 (15 males and 15 females) cases and 60 controls. The most commonly reported symptoms were abdominal pain (100%), diarrhea (24%), vomiting (3%) and fever (17%). The epidemiological curve shows that the onset of the first cases was at 3:00 AM on Thursday 31/08/2017 then the cases started to increase gradually until 9:00 PM on the same day; the peak was at 3:00 PM. The incubation period was 6-32 hours with a mean of 22 hours. The analysis showed a statistically significant association with eating lamb meat and tomato stew (P = 0.03 for and P = 0.04, respectively). The association with the other two kinds of food; rice and Kubah were not statistically significant. All laboratory results were negative.

Conclusions According to the clinical picture and the incubation period, the probable causative organism might be Salmonella, or E. coli. The outbreak was epidemiologically linked to the lamb meat and tomato stew. At the same time the delay of notification could be the reason behind the negative laboratory investigation. To prevent any similar future outbreaks, an improvement in food transport pathway and food storage is recommended.

Outbreak Investigation/FWBD (Waterborne)

Abstract Code: 2017-EOH-371

Presenter Name: Dr. Safaa Saadoon
Country: Iraq
Presenter Email: safaasaadoon@gmail.com
Author: R. Hashim, Q. Abdulaziz, H. Ismael, M. Ali, Z. Wajih, S. Saadoon, F. Lami

Title Cholera Outbreak Investigation in Baghdad Al-Rusafa August-November 2017

Background Iraq is a cholera-endemic country with outbreaks occurring every 3-4 years. Since 29th, August 2017, Iraq CDC received reports on confirmed cholera patients in Baghdad/ Resafa (Eastern Baghdad with a population of 4.8 million). Up to Nov 29th the total number of confirmed cholera patients was 257.

Purpose To determine the epidemiological characteristics of the patients and identify potential risk factors.

Methodology A team of FETP residents dispatched to investigate the outbreak and developed a form to compile demographic and epidemiological data from all the patients' investigation forms that were filled at the treating hospitals. Then a case-control study conducted using the accessible cholera patients and neighbors' controls. The cholera case was defined as any patient with acute watery diarrhea, and isolation of Vibrio cholera O1 or O139 from a stool sample.

Results Among the 257 confirmed cases, 51% were male, mean age was 38.2 years and 44% were illiterate. The attack rate was 5.4/100,000; the highest was in Baladiyat (13/100,000), Baghdad - Jadida (12/100,000), and Madain (8/100,000) districts. The case fatality rate was 1.17%. The significant risk factors were: drinking tap water (OR=5.2, 95% CI:2.2-12.5), using Reverse Osmosis water (OR=8, 95% CI:2.4-26.1), not using boiled water (OR=2, 95% CI:1.2-3.8), not practicing washing hand after using a toilet (OR=5, 95% CI:1.9-13.4), lack of awareness toward boiling water (OR=1.8, 95% CI:1.04-3.3) and history of having meals outside home within seven days preceding onset (OR=4.8, 95% CI: 1.9-12.2). No significant association found with the following factors: education, occupation, crowding index, house sewage disposal type, using aqua tabs, chlorine readings, and travel history.

Conclusions Shortage of safe water and poor hygiene are still the main causes of cholera outbreaks in Iraq. Political commitment towards the provision of safe water and improving sanitary conditions are the cornerstone to stop these outbreaks.

Outbreak Investigation/FWBD (Waterborne)

Abstract Code: 2017-EOH-345
Presenter Name: Dr. Khurram Akram
Country: Pakistan
Presenter Email: drkhurram114@gmail.com

Title Investigation of Cholera Outbreak at Rawalpindi, Pakistan - August 2017

Background Cholera is endemic in Pakistan with many outbreaks during the summer season. On July 29, 2017, two suspected cholera cases were reported from a tertiary care hospital in Rawalpindi. On the request of District Health Authorities, a team was constituted.

Purpose To assess the magnitude of the outbreak, evaluate possible risk factors and recommend control measures.

Methodology Investigation was carried out from Aug 01-15, 2017. Hospital records were reviewed, and active case-finding was conducted. A case was defined as "sudden onset of loose watery stools (≥3 in past 24 hours) with any of the symptoms like vomiting, nausea, abdominal cramps or fever in a resident of Dhok-Paracha, Amarpura & Dhok-Chaudhriyan, Rawalpindi, from July 19-August 07, 2017. Age and sex-matched neighborhood controls were enrolled. Data was collected using a structured questionnaire. Four stool samples and three water samples were sent to National Institute of Health for microbiological analysis

Results A total of 30 cases with 02 deaths (CFR 2.2%) were identified out of which 28 cases were detected through active case-search. There was a male predominance (n= 20; 66%) with mean age of 13.7 years (range: 02 months-55 years). Overall AR was 0.68% with 16-20 years being the most severely affected age group (AR 1.8%). Out of 30 cases, 14 were consuming well-water (OR:10.37, 95%CI:3.61-29.74) and12 were consuming tap water (OR:3.94, 95%CI:1.54-10.08). Water samples showed presence of coliforms (240 CFU/100 ml). Vibrio Cholera Serotype Inaba isolated from stool samples. Heavy rainfall was recorded (455.5 ml) from June 26 to August 6, 2017.

Conclusions Consumption of contaminated water was the most probable cause of the outbreak. Contamination of water sources during recent flash floods was the source of contamination. Chlorination of water sources was conducted. Health awareness sessions on safe drinking water were conducted in the community.

Outbreak Investigation/FWBD (Waterborne)

Abstract Code: 2017-EOH-193
Presenter Name: Mrs. Ahlam Alsaidi
Country: Yemen
Presenter Email: ahlamalsaidi@hotmail.com
Author: M. Al-Amad, N. Al-Abhar, L. Al-Zagar, Y. Abdaulwareth

Title Cholera Outbreak Investigation in Sawan Area, Shuaub District, Sana'a Capital City, Yemen, October 2016

Background Diarrheal diseases are the second leading cause of death among children <5, 1 in 9 children die from diarrhea infection worldwide. The majority (88%) of these deaths are associated with unsafe water, inappropriate sanitation and lack of hygiene. Vibrio Cholerae bacterium can cause extremely virulent diarrhea. On October 2016 an outbreak of watery diarrhea occurred among a family in Al-Nasser St. Sawan area, Shuaub district, Sana'a capital city, Yemen.

Purpose An outbreak investigation was done to confirm the outbreak, to identify the source, risk factors and recommend control measures.

Methodology A descriptive and analytical studies were conducted. Active search of cases and control was performed. WHO case definition of cholera during epidemic was adopted. Stool samples were taken and sent to the public health central lab. for confirmation. The data was entered and analyzed by Epi Info 7.2 and Excel sheet were performed. Chi square and odds ratio were calculated.

Results Out of 28 cases, 15 (54%) were females. The index case, a 65 years-old female, was identified on October 3rd. The peak of cases was on October 8th, 2016 (46%). The attack rate was 3/1000 population with no case fatality. Children < 5 (24%) and those 5-10 years (24%) were mostly affected. Out of 18 stool samples, 11 Vibrio cholera serotype 01 Eltor-ogawa was isolated (61%). The identified risk factors were: untreated water OR=10.7, (CI: 3.95-29.1) grape OR=4 (CI: 1.5-10.8), and porridge OR=3.1 (CI: 1.4-7.5).

Conclusions Cholera outbreak was confirmed. Most cases were children up to ten years old. There was association between drinking water, eating raw vegetables and cholera infection. Boiling or chlorination of water and hand washing were recommended to control the outbreak

Outbreak Investigation/FWBD (Waterborne)

Abstract Code: 2017-EOH-474
Presenter Name: Dr. Aftab Khaskheli
Country: Pakistan
Presenter Email: draftabali@gmail.com
Author: N. Masood

Title Outbreak Investigation on Acute Watery Diarrhea in Village Mir Khan Otho, District Shaheed Benazirabad, Sindh Pakistan, 2017

Background On 9th July 2017, media reported eight cases of acute watery diarrhea and abdominal pain in village Mir Khan Otho, District Shaheed Benazirabad to the DG Health Office Sindh in Hyderabad. On 10th July 2017 a team of FELTP fellows was deputed to affected village.

Purpose To confirm the outbreak, estimate the magnitude, evaluate risk factors and make recommendations.

Methodology Age and sex-matched case-control study was carried out. Active case finding was done. A case was defined as sudden onset of acute watery diarrhea (≥3 loose motions within 24 hours) with or without vomiting, fever or abdominal pain in a resident of Village Mir Khan Otho, between 2nd and 20th July 2017. A structured questionnaire was used to collect data. Frequencies and attack rates were calculated and bivariate analysis was conducted. Three stool samples were sent to People University of Medical & Health Sciences, Nawabshah for microbiological analysis.

Results A total of 30 cases were identified (22 through active case finding) and n=16 (53.7%) were females. Mean age was 25.3 years (range: 1-50 years). Overall attack rate was 23%. People aged 21-30 years were the most affected (n=10; AR 43.5%). Apart from diarrhea, abdominal cramps (n=28; 93%) was the most common symptom. On bivariate analysis, consumption of water from the hand-pump near the swamp was significantly associated with the disease (OR=8.4, 95% CI: 3.1-22.7) while frequent hand washing had a protective effect (OR=0.3, 95% CI: 0.16-0.59). Vibrio cholerae was detected in 2 stool samples. A swamp was created near the hand pump due to recent rains.

Conclusions The most probable cause of outbreak was contamination of hand-pump by swamp water. Hand-Pump was removed thereby culminating the outbreak. ORS and chlorine tablets were distributed and health education sessions on personal hygiene and treatment of drinking water was imparted to the community.

Outbreak Investigation/FWBD (Waterborne)

Abstract Code: 2017-EOH-87
Presenter Name: Dr. Labiba Anam
Country: Yemen
Presenter Email: labibaanam25@gmail.com

Title Cholera Outbreak in Heran Area, Dhamar District, Dhamar Governorate, January 2017

Background On January 14, 2017, a suspected watery diarrhea outbreak was reported in Heran area, Dhamar district, Dhamar Governorate by electronic disease early warning system (eDEWS) coordinator. A team from FETP had investigated the outbreak.

Purpose To confirm the outbreak, find out the source, and recommend control measures.

Methodology Unmatched case control study was conducted with ratio of 1: 2 respectively. A case was defined as a person with acute watery diarrhea, with or without vomiting in Heran area. A control was defined as a person from the same house or neighborhood of a case who does not has diarrhea or vomiting. Data were collected from cases and control using semi-structured questionnaire including basic demographic information, potential food and water exposures, and hygienic practices. Seven stool samples were taken only from patients for laboratory confirmation of cholera by culture. Water and sewage samples were also taken. We performed bivariate analysis using Epi-Info version 7.2.

Results A total of 12 cases and 24 control that met the case definition were recruited. About 83% of cases were clustered in one house, 40% of cases aged 7 -14 years, and 58% were females. The overall attack rate in Haran area was 0.1% and the case fatality among cases was 8.3%. The only significant risk factor identified was poor sanitation [OR= 5.9, 95% CI:1.1-32.9]. Vibrio cholera 01 serotype ogawa was isolated from all stool samples but all water samples were found to be negative.

Conclusions An outbreak of cholera was confirmed in Heran area, Dhamar governorate. Poor sanitation was identified as possible mode of transmission. Therefore, improving environmental sanitation through setting up a sewage system is needed. Boiling water and chlorination are also recommended with increasing community awareness regarding the importance of hand washing and safe water and food hygiene.

Outbreak Investigation/FWBD (Waterborne)

Abstract Code: 2017-EOH-440

Presenter Name: Dr. Jamshed Khan

Country: Pakistan

Presenter Email: dr.jamshedkhanzada@hotmail.com

Title Acute Watery Diarrhea (AWD) in a Chashma village of Ibrahim Hyderi, District Malir Karachi, Sindh, Pakistan - 2015

Background On 4th August 2015, electronic media reported unusual occurrence of acute watery diarrhea (AWD) cases and one death in Chashma village at Ibrahim Hyderi, District Malir, Karachi. On same day, District health authority deputed 3 members team to identify the source, risk factors and recommend preventive measures.

Purpose To estimate the extent and magnitude of the outbreak, to assess risk factors associated with the outbreak, to institute control measures, and to formulate recommendations for future prevention.

Methodology A matched case control study was conducted. Case was defined as "any person living at Chashma village, who has 3 or more watery stools in last 24 hours from 2nd to-10 August 2015". Review of hospital records and active case finding was done. Age and sex matched controls (1:1) were taken to analyze risk factors. Environmental assessment was done. Six water samples and rectal swabs were collected to isolate the causative agent.

Results A total of 189 diarrhea cases were identified out of which (56%) were female. Mean age was 32 years (rang: 4- 64 years). Along-with diarrhea (100%), the abdominal pain (56%), Vomiting (54%), nausea (11%), blood in stool (6%), and fever (2%)were the predominant symptoms. Case fatality was 3.2 with Attack rate 1.4%. Drinking water from storage tank (OR: 12.8, p-value <0.05, CI=1.5-14) found strongly associated with the illness. Sanitary assessment revealed that usage of contaminated drinking water from storage tank resulted outbreak. The water samples were unfit for drinking due to presence of coliform bacteria. Vibrio cholera O1 El Tor Bio type isolated from 4 of 6 stool samples.

Conclusions Contaminated storage tank water was possible source of outbreak. Immediately evacuate and contain the water tank. Poor personal hygiene and sanitation may be aggravating factors. Health education regarding hygienic practices and use of safe drinking water were imparted. Chlorinated tablets distributed. Washing and bathing was prohibited at drinking water source. Sustained health education and provision of purified water were recommended.

Outbreak Investigation/FWBD (Waterborne)

Abstract Code: 2017-EOH-404

Presenter Name: Dr. Abdul Razziq

Country: Pakistan

Presenter Email: Dr.abdulrazziq@outlook.com

Author: B. Saeed

Title Acute Gastroenteritis Outbreak in Union Council Khirzan, District Khuzdar Pakistan - 2017

Background On 30th June 2017, district Health Officer (DHO) Khuzdar reported 60 suspected cases of acute gastroenteritis from union council Khirzan 2 days after heavy rains and flood in the area. DHO requested Provincial Disease surveillance and response Unit for investigation.

Purpose On 1st July 2017 a team was deputed to confirm outbreak, evaluate risk factors and recommend control measures.

Methodology A case was defined as "sudden onset of 3 or more episodes of loose stools per day with or without vomiting in a resident of UC Khirzan District Khuzdar from 28th June to 4th July 2017". Active case finding was done in affected village and records of Rural Health Center were reviewed. A case control study was conducted. Age and sex matched controls were enrolled in a ratio of 1:1. Water sources and toilet facilities were assessed. Three water samples from water stream and household wells were taken and sent to NIH Islamabad.

Results A total of 105 cases were identified with an overall attack rate of 1.08%. No deaths were recorded. Mean age was 27 years (range = 1 to 70 years). Males were more affected n=54 (51%). Most affected age group was 0-4 years (n=29, 27.6%) with attack rate 0.3%. Dehydration (n=95, 94%), nausea (n=80, 76.1%) and vomiting (n=70, 66.6%) were most frequent symptoms. About 95% of people practiced open defecation. About 78% of cases used stream water (OR= 11.40, 95% CI: 5.98-21.73) while 22% used well water (OR= 0.087, 95% CI: 0.046-0.167). Lab results showed coliform and fecal coliform organisms in stream water sample.

Conclusions The most probable cause of outbreak was fecal contamination of stream water after floods. On recommendations of this study, community started boiling water before use and local authorities also distributed chlorine tablets for disinfecting water along with awareness regarding usage of latrines.

Outbreak Investigation/FWBD (Waterborne)

Abstract Code: 2017-EOH-508
Presenter Name: Dr. Zee Shan
Country: Pakistan
Presenter Email: drzeeshan1986@gmail.com
Author: M. Saleem

Title Outbreak investigation of Suspected Acute Watery Diarrhea Cases in a Village of District Shangla, Khyber Pakhtunkhwa, Pakistan in September 2016

Background On 18th September 2016, leading newspapers reported about 150 suspected acute watery diarrhea cases in a village in district Shangla. In response, FELTP Fellow visited the area on the next day.

Purpose To investigate the outbreak and take necessary control measures.

Methodology A case was defined as 'any person of any age and gender belonged to the reported village of district Shangla, with 3 or more episodes of loose stools per day with or without vomiting from 12–20 September 2016'. Descriptive study was carried out by visiting the affected village to identify cases, areas and risk factors. District Health Information System reports were reviewed for any reported cases from that area during previous months. Admitted cases and other cases found during active search were interviewed. Samples from different water sources were taken and sent to laboratory for analysis.

Results Three hundred and thirty-six cases were identified from hospital record and during active case finding in the affected village among population of 2800 (attack rate; 12%). Age range of cases was 5–65 years with mean age of 26 years. 32% (n=109) cases were females while 68% (n=227) were males. Male to female ratio of cases was 2.1:1. 27% (n=92) cases belonged to 20-29 years age group while 24% (n=81) from 30-39 years age group. It was found that 71% (n=237) cases were using spring water (attack rate; 8.5/100,000 population) and remaining 29% (n=99) were using other water sources (attack rate; 3.3/100,000 population). Laboratory reports confirmed presence of Escherichia coli in the spring water samples.

Conclusions Use of contaminated water was the probable cause of outbreak. Outbreak was controlled, no death occurred, and cases have recovered. Health education sessions were conducted wherein community was advised to boil water for drinking and cooking. Supply of safe drinking water to the community was recommended.

Outbreak Investigation/FWBD (Waterborne)

Abstract Code: 2017-OTH-158
Presenter Name: Dr. Mahmood Abdulrazzak
Country: Yemen
Presenter Email: dmahmood1982@gmail.com
Author: M. Alemad, A. Al Shahithy, L. Alzagar

Title Diarrhea Outbreak Investigation in Weld-Rabiae District, Albaida Governorate, Yemen, 2016

Background Globally, diarrheal disease is the second leading cause of death among children under five. Account for one per nine deaths of children. About 88% of diarrhea death cases associated with unsafe water, inappropriate sanitation and lack of hygiene. In Yemen, it is the second cause of mortality and morbidity in children. On 19 August 2016, an increase number of diarrheal patients was reported in Wild-Rabiae district, Albaida governorate notified by local surveillance coordinator. A team from FETP was sent for investigation.

Purpose To confirm the outbreak, find out the source, and recommend control measures.

Methodology A descriptive cross-sectional study was performed. Data were collected using modified CDC cases definition. Samples (drinking waters, and powder milk) were collected, and sent to the National Central Public Health laboratories for testing. Data were entered into Excel and analyzed using EPI-info program.

Results A total of 53 residents met the case definition; 85% of them were in the neighborhood, and 15% were in the refugee building. All cases were children less than 15 years old. Age group of 1 - < 3 years was most affected (38%). About 60% of cases were male. The overall attack rate was 9.5% (13% in the refugee building and 9% in the neighborhood). The overall cases fatality rate was 7.5%. Drinking water in neighborhood and powder milk in refugee building were contaminated by coliform and E. coli.

Conclusions Diarrhea outbreak among children less than 15 years old was confirmed in Weld-Rabiae district, Albaida Governorate, Yemen. Two sources of infection were identified; drinking water in neighborhood and powder milk in refugee building. Consumption of contaminated powder milk among the refugee building led to severe fatal diarrhea.

Outbreak Investigation/FWBD (Waterborne)

Abstract Code: 2017-EOH-347

Presenter Name: Dr. Rabia Subuktageen
Country: Pakistan
Presenter Email: drrabia_babna@hotmail.com
Author: N. Azam, A. Baig

Title Risk Factor Assessment of Hepatitis-E Outbreak at a Military Training Center – Karachi, Pakistan 2017

Background In Pakistan hepatitis E occurs in both sporadic and epidemic forms. On 30th March 2017, 30 suspected cases of hepatitis were reported from a military training center in Karachi.

Purpose A team of FELTP fellows was deputed to assess the magnitude, evaluate risk factors and recommend control measures.

Methodology The investigation was carried out in the military training center in Karachi. A case-control study was conducted. A case was defined as "sudden onset of jaundice with or without fever, nausea, vomiting, loss of appetite, malaise, diarrhea and abdominal pain plus presence of Hepatitis E IgM on ELISA in a resident of military training center from 9th March to 12th May 2017". Cases were identified by reviewing hospital records and active case finding. Age-matched controls were selected from the same center. Water samples were tested for presence of coliforms. Frequencies were ascertained, attack rates calculated, and odds ratios were determined at 95% confidence interval and p value of less than 0.05.

Results A total of 79 cases were identified (49 through active case finding). All cases were male with mean age of 22 years (range18-45 years). Overall attack rate was 9% with most affected age group of 18-22 years (attack rate of 12%). Out of 79 cases 62 consumed tap water (OR: 2.28, 95%CI:1.14-4.58). Consumption of filtered water from coolers and hand washing was shown to have a protective effect. Water samples were positive for coliforms. On environmental assessment water pipelines were seen to be running parallel to the sewage line

Conclusions Consumption of contaminated tap water was the most probable cause of the outbreak. Cross contamination between the water and sewage lines was the possible source. Replacement of old pipelines and decontamination of drinking water before consumption was also recommended.

Outbreak Investigation/VPD

Abstract Code: 2017-EOH-510

Presenter Name: Dr. Shumaila Rasool
Country: Pakistan
Presenter Email: drshumailazubair@gmail.com
Author: N. Masood

Title Diphtheria Outbreak in Village Khabri Bhitt Tehsil Salehpat District Sukkur, Pakistan, 2016 - A Descriptive Study

Background On 9th September 2016, 2 deaths reported in local newspaper due to unknown disease of respiratory system in village Khabri Bhitt Tehsil Salehpat, District Sukkur. On 10th September FELTP fellows visited the affected village.

Purpose To assess the cause of death, evaluate the magnitude of outbreak, and to recommend control measures.

Methodology A descriptive study conducted. Active case finding done and data collected using pretested questionnaire. Health facility records checked and verbal autopsies carried-out. "A case was defined as any person with fever, throat pain and Greyish white membrane on Tonsils or nasal mucosa or Pharynx or larynx with or without any of the following symptoms including: neck swelling, difficulty in swallowing residing in Union council Tarai from 20th August to10th September 2016". Data was analyzed while using MS Excel and Epi Info.

Results Six cases including 2 deaths identified (CFR=33%). Median age of 7.1, males 83% and females were 17%. Affected age group was between 5-10 years with 2.8/1000 followed by 0-5 Years with attack-rate of 2.5/1000. Overall attack rate was 4/1000.Clinical Features were Fever, sore throat, difficulty in swallowing 100% and cough 33%. On examination Rhinitis 83%, tender enlarged cervical Lymph nodes 67%, Tonsillitis 100% and whitish membrane on tonsils 67%. Vaccination status of cases for Pentavalent I, II, III was 17%. Seven clusters of routine immunization checked on recall BCG (51%), Penta-I 4%, Penta II 2%, Penta III 2% and Measles-I 38%.

Conclusions Outbreak was most probably due to very low routine immunization coverage and poor health seeking behaviors contributed the mortality. Area was remote and health facilities were far-flung from the reach of affected population. Mortality was due to non-availability of Diphtheria anti-toxin and improper medication. Outbreak was timely contained with initiation of focused vaccination. VPD Surveillance and EPI activities must be enhanced.

Outbreak Investigation/VPD

Abstract Code: 2017-EOH-178
Presenter Name: Mr. Ali Alhasani
Country: Yemen
Presenter Email: ailalhassni@hotmail.com

Title Diphtheria Outbreak in Bait Hulboob, Wasman Villages, Ibb governorate - Yemen, September 2017

Background Remerging of diphtheria outbreaks have been associated with wars and threaten the health of children and adult. On 25th of September 2017 surveillance officer of Ibb governorate received a notification from surveillance coordinator of Alsaddah district about suspected deaths due to diphtheria. A team from Ibb surveillance office was sent to investigate on 26/09/2017.

Purpose To verify the existence of an outbreak, describe its epidemiology and recommend preventive and control measures.

Methodology A descriptive study was done. WHO case definition was used. A line list was created, active case finding, and clinical diagnosis were performed. Records of surveillance and vaccinations programs were reviewed. Data was entered and analyzed using Excel program. Descriptive statistics were performed.

Results A total of 63 cases were identified. The first case was in Epi week 34, the highest peak with 32 cases were in week 38. The index case was 17 years old male from Bait Hulboob village. The majority of cases 47 (74%), were from that village. Almost three quarters of cases 45 (71%) were males, 22 cases (35%) among children aged from 5-10years. 43 cases (68%) was unvaccinated and the rest was partially vaccinated. All presented with pseudo membrane, 42 cases (67%) with sore throat and 10 (16%) with bull neck. The attack rate was 15/1000. 4 cases died (the case fatality rate was 6%).

Conclusions Diphtheria outbreak was clinically confirmed. Low vaccination coverage. Vaccination campaign for population and provision of Diphtheria antitoxin for patients are urgently recommended. Immunization and health education should be emphasized by governorate authority.

Outbreak Investigation/VPD

Abstract Code: 2017-EOH-308
Presenter Name: Dr. Hejer Letaief
Country: Tunisia
Presenter Email: hejerletaief@gmail.com
Author: A. Hechaichi, H. Bouguerra, F. Saffar, S. Chelly, A. Cherif, S. Rejaibi, M. Mechala, K. Chahed, S. Bougatef, M. Kouni, M. Ardhaoui, E. Ennaifer, S. Boubaker

Title Determinants for Genital Human Papillomavirus (HPV) Infection in Tunisian Women with Normal Pap smear: Are There Different Risk Profiles for Oncogenic and Nononcogenic HPV Types?

Background Most studies of risk factors for HPV infection have focused on overall HPV positivity and have not examined determinants for high-risk and low-risk HPV types separately.

Purpose To evaluate the prevalence and type distribution of high-risk and low-risk HPV infection and to describe the risk factors associated with different profiles in Tunisia.

Methodology We studied risk determinants for genital HPV infection in a randomly sample of women aged 18 years and older with normal cervical cytology in Tunisia during 2014. All women had a personal interview, a Pap smear, and cervical swabs for HPV DNA detection using a PCR technique. Information on sociodemographic, reproductive, lifestyle characteristics, and health-seeking behaviors was collected.

Results The overall HPV prevalence was 7.5% (IC95% [5.9%-9.0%]). Prevalence of HRHPV was 4.8%(IC95% [3.7%-6.2%]) and LRHPV 3.8%(IC95% [2.8%-4.8%]). Of HPV-positive women, 64.0% (IC95% [53.8%-74.4%]) had high oncogenic HPV types, and 45.3% (IC95% [33.0%-54.8%]) had low oncogenic HPV types. Simple infection was predominant within 76.6% (IC95% [66.3%-83.9%]) of positive women. Younger age and lifetime measures of sexual activity (notably, number of partners) were the main risk factors for the oncogenic HPV types. A statistically increase risk was found in women whose partners had multiple sex partners. Furthermore, a previous sexual infection was associated with the high-risk HPV types. HR-HPV infection risk was observed in women aged less than 30 years, who were smokers, divorced/separated and having a high educational level. In contrast, the most important determinants for nononcogenic HPV infection were contraceptive variables related to the physical protection of the cervix and low standard of living.

Conclusions The results indicate that different risk profiles exist for infections with different HPV groups with high-risk types being the most common types detected. HR-HPV positivity was associated with social determinants, behavioral and sexual characteristics. These results should assist in designing strategies for control of cervical cancer targeting at risk population.

Outbreak Investigation/VPD

Abstract Code: 2017-OTH-95
Presenter Name: Dr. Mona Mayad
Country: Yemen
Presenter Email: alferdos25@yahoo.com
Author: W. Albakhshi

Title Measles Outbreak-Khywan, Huth District, Amran Governorate, Yemen, March, 2017

Background On March 3, 2017, cases with fever and skin rash were reported in Khywan, Huth district, Amran by electronic disease early warning system (eDEWS) coordinator. A team from Y-FETP was sent to investigate the problem.

Purpose To confirm the existence of outbreak and recommend control measures.

Methodology We conducted active house to house- case search. Suspected case was any person with fever and skin rash at any age in Khywan area, Huth district, Amran governorate, from 5 February to 5 March 2017. Data analysis was performed by Epi Info version 7.2.

Results 32 cases met the standard case definition, the index case was reported in 5 Feb. 2017, the outbreak continued till 5 March with the peak of cases in 3 March 2017 (6 cases). The percentage of females was 53%. The highest percent was among the age group 1-< 5 years with the attack rate 21/1000 of population. The percent of non-vaccinated cases was 97%. Reasons for non – vaccination were: lack of outreach vaccination services (39%), absence of vaccine in the nearby health center (39%), and vaccination refusal (22%). All cases had fever and skin rash, 97% of cases had cough and conjunctivitis. About 13% of cases were admitted in Huth hospital due to pneumoniae as a complication of measles.

Conclusions Existence of Measles outbreak in Khywan area, Huth district, Amran governorate was confirmed. Urgent measles immunization campaign with ongoing routine and outreach immunization services are recommended to increase vaccination coverage.

Outbreak Investigation/VPD

Abstract Code: 2017-EOH-376
Presenter Name: Dr. Aslam Pervaiz
Country: Pakistan
Presenter Email: aslam_pervaiz92@yahoo.com
Author: M. Ali

Title Investigation of a Measles Outbreak Identified by Front Line Polio Workers, District Shikarpur, Sindh - 2017

Background An innovative strategy; to identify the vaccine preventable disease cases from the community by the front-line polio workers during door to door OPV campaign, was adopted in district Shikarpur, Sindh. During polio field work training front line workers were also briefed about sign and symptoms of vaccine preventable diseases to identify and report their cases. On January 17, 2017 (1st day of OPV campaign) front line polio teams reported three measles cases including one death from a remote rural village. A team was deployed to confirm and determine the extent of the outbreak and implement preventive and control measures.

Purpose To confirm and determine the extent of the outbreak.

Methodology A case was defined as a child (= 15 years of age) residing in district Shiparpur, with: a generalized rash for three or more days, fever at or above 101°F, and one or more of the associated symptoms, including cough, or coryza, or conjunctivitis from 1st January to March 6, 2017. Active case finding was done from the community and health facilities. Blood samples from fourteen willing cases were collected and sent to NIH Islamabad for Laboratory diagnosis.

Results Twenty cases were identified with one death (CFR 5%), 55% (n = 11) were females. Mean age was 43 months (range: 11 to 108 months). Eight (40%) cases were identified by front line polio workers from remote areas. Eight cases were from two families. Fourteen (70%) cases were un-immunized and 6 (30%) partially immunized against measles. NIH Lab declared thirteen (93%) cases positive out of fourteen.

Conclusions Cases appeared due to poor vaccine coverage. Mopping–up activities were conducted in the five villages with clustering of cases and in five KM radius surrounding areas. Innovative strategy of identify the vaccine preventable disease cases from the community by the front-line polio workers have proved successful and needs to be implemented across the country.

Outbreak Investigation/VPD

Abstract Code: 2017-EOH-119

Presenter Name: Dr. Muhammad Ismail
Country: Pakistan
Presenter Email: drismail_82@yahoo.com
Author: A. Khan

Title Measles Outbreak Investigation in Village Bara, Khyber Agency, Pakistan-February 2017: A case Control Study.

Background Measles is highly contagious and remains a leading cause of childhood mortality. Outbreaks of Measles were reported from various parts of Pakistan, particularly tribal areas. In February 2017 Measles outbreak was reported from Bara, Khyber Agency.

Purpose To confirm the outbreak, assess its magnitude, identify risk factors and to implement prevention and control activities to stop spread of the outbreak.

Methodology A case was defined as "Rash with fever and cough, coryza or conjunctivitis in a child younger than 14 years of age, resident of Bara, during 3rd February to 15th March, 2017." Active search of cases done. Data was collected using a pretested structured questionnaire and analyzed using Epi Info version 7.1. Descriptive analysis was done followed by age & sex matched case control study. Case fatality rate, attack rate, vaccine efficacy, and secondary infectivity rates were calculated.

Results Total 42 cases were identified. The Epi curve ranges from 3rd February to 15th March with a bi-modal peak on 23rd Feb and 5th March. Mean age of the cases was 37 months (range 1-120 months). Males were 52.38% (n=22). Socioeconomic status of parents was poor (28.57%laborers, 38.10% earn below 10000 PKR/month, 59%fathers were literate while all mothers were illiterate). Case Fatality rate was 7.14% (n=3). Vaccination showed protective effect [OR 0.12 (95% CI: 0.03-0.40; P<0.05)]. Vaccination coverage survey showed 46.3% coverage for Measles-1. Secondary infectivity was high in houses where children were more than 7 in number [OR 8.67 (95% CI: 1.76-42.6, P<0.05). Distance from health facility (>3kms) showed higher odds of getting the illness [OR 2.89 (95%CI: 1.22-6.84); P<0.05].

Conclusions This study in a post-conflict repatriated population showed relationship of low socioeconomic status, low vaccination coverage, distance from health facility and overcrowding with the disease. Awareness sessions and mop-up vaccination in the area contributed in controlling the outbreak.

Outbreak Investigation/VPD

Outbreak Investigation/VPD

Abstract Code: 2017-EOH-524

Presenter Name: Dr. Rukhsana Channar
Country: Pakistan
Presenter Email: rukhsanapreet@gmail.com
Author: N. Masood

Title Measles Outbreak Investigation in Village, Mehmood Jamali, UC Ghandtar District Shaheed Benazirabad

Background On 10th November 2017, District Surveillance Coordinator Shaheed Benazir-Abad notified eight suspected measles cases to Disease Surveillance Unit Hyderabad. Teams of Fellows constituted and deputed to the village to investigate the reported cases.

Purpose To verify, assess the magnitude, to evaluate risk factors and formulate recommendations for future control.

Methodology A descriptive study was carried out. Active case finding carried out; hospital records checked. Case was defined, a person resident of Jogi para Village Mehmood Jamali having fever with maculopapular rash and one of the following clinical features; cough, coryza and conjunctivitis from 1st November 2017 to 13th December 2017. Verbal autopsy was carried out with parents of deceased children. Data were analyzed using Epi Info 7.1.

Results Total 37 suspected cases identified with 3 deaths (CFR 8%). Median age was 36 months (range: 6-216 months). Male were more affected n=27 (73%) than Females n=10(27%) most affected age group was 12 to 35 months n=23 (AR 1.5%) while overall AR was 1.2%. Fever and maculopapular rash n=37 (100%), coryza n=25 (67.5%) and cough n=23 (62%) were clinical presentation. Post measles complications were Pneumonia n=12 (32%) and diarrhea n=5 (13.5%). All cases were un vaccinated for measles vaccinations. The immunization status evaluation showed n=12 (32%) cases had not received measles-1 vaccination.

Conclusions Measles outbreak was most probably due to very low routine immunization coverage. Outbreak was timely contained with active surveillance and initiation of Mop up vaccination. Cases were mobilized and admitted in hospitals therefore, no more deaths reported. There is need of strong supervision and monitoring of routine immunization activities in the district. Health education sessions should be arranged in the community on importance of immunization with active involvement of local stakeholders.

Outbreak Investigation/VPD

Abstract Code: 2017-EOH-425

Presenter Name: Dr. Waqar Dahar

Country: Pakistan

Presenter Email: daharwaqar@yahoo.com

Author: N. Memon

Title Measles Outbreak in Village Manzoor Sawand District Ghotki, Province Sindh, Pakistan- 2016

Background On 3rd December 2016, District Health office Ghotki reported three suspected measles cases from Village Manzoor Sawand to DG Health Office Hyderabad. On 4th December 2016, two FELTP Fellows were deputed to initiate an outbreak investigation.

Purpose The objectives of the investigation were to assess the magnitude, evaluate risk factors, and suggest control measures.

Methodology A review of the reported cases was conducted. Active case finding was undertaken to identify more cases in the village. A case was defined as sudden onset of fever, maculopapular rash, and any of cough, coryza, conjunctivitis in a resident of village Manzoor Sawand, from 10th November to 10th December 2016. Data was collected using a structured questionnaire. Vaccination coverage survey was done by using 30 x 7 cluster sampling technique. Six blood samples were sent to NIH for lab confirmation.

Results A total of 15 cases including 2 deaths (CFR13.3%) were identified; 12 through active case finding. Mean age was 24 Months (range 9-54 months). Overall attack rate was 1/1000 and the most affected age group was 12-48 months n=13 (AR 3.8/1000). Apart from fever and rash cough (100%), coryza (80%) and conjuctivitis (60%) were the most prominent symptoms. A total of 210 children were assessed for vaccination status. Attack rate in unvaccinated children was 2.1% and in vaccinated children was 4.3%; vaccine efficacy was 51%. Reasons for non-vaccination was unawareness (n=12; 80%) followed by vaccinator did not visit (n=7; 46.6%). Ice-lined-refrigerator (ILR) was found to be non-functional at the EPI Centre. All blood samples were positive for measles.

Conclusions The most probable cause of the outbreak was low immunization status. Awareness sessions were conducted. Vaccinator was assigned to conduct outreach activities. A total of 713 eligible children were vaccinated against measles and Vitamin A was administered. On our recommendations ILR was made functional.

Outbreak Investigation/VPD

Abstract Code: 2017-EOH-220

Presenter Name: Dr. Safaa Saadoon

Country: Iraq

Presenter Email: safaasaadoon@gmail.com

Author: F. Lami

Title Trends of Performance Indicators of Acute Flaccid Paralysis (AFP) in Iraq, 2007-2016

Background AFP Surveillance is a key strategy of Global Polio Eradication. In Iraq, it started in 1995. WHO established some performance indicators that should be used to assess the quality of AFP surveillance.

Purpose The objective of this study is to identify the trends of different performance indicators of AFP in Iraq, 2007 – 2016.

Methodology We reviewed AFP surveillance data in the IFA (Information for Action) software for 2007-2016. The data were compiled from almost all public health outlets in Iraq and compiled at the National AFP surveillance office. Six WHO indicators were reviewed: Non-polio AFP rate <15 years of age (Target >2/100,000), Reported AFP cases investigated <48 hours of report (Target >80%), Reported AFP cases with 2 specimens collected <14 days since onset (Stool adequacy) (Target >80%), Specimens arriving at national laboratory <3 days of being sent (Target >90%), Specimens arriving at laboratory in good condition (Target >80%) and Stool specimens from which non-polio enterovirus was isolated (NPEV%) (Target >10%).

Results A total of 4,915 cases were reported; 35% aged <2 years, 60% were male, 60% had fever at onset of paralysis and 38% had asymmetrical Paralysis. Only 1.3% received no OPV vaccine, 77% received >3 doses. At sixty days' examination 24% had residual paralysis. Seven cases were vaccine-derived, 156 cases were Sabin-like polioviruses and no vaccine-associated polio case. Two wild polio cases reported in 2014. Guillain-Barre syndrome accounted for 51% of cases. Specimens arriving at the national laboratory <3 days was not achieved during the whole period. NPEV% achieved the target except in 2013 (7.3%). All other indicators achieved the target throughout the whole period.

Conclusions While most indicators achieved the standard, additional efforts are still needed to address the timeliness of adequate stool specimens' arrival to the laboratory. We are currently working on similar analysis at governorates and districts levels.

Respiratory Diseases

Abstract Code: 2017-EOH-392

Presenter Name: Dr. Nisharga Meraj Chowdhury
Country: Bangladesh
Presenter Email: nisharga786@gmail.com
Author: M. Abdul Aleem, M. Sabrina,

Title Antibiotics Prescribed Among Patients with Severe Acute Respiratory Illness in Bangladesh

Background Proper use of antibiotics helps to minimize the recovery time of patients with severe acute respiratory illness (SARI). Although Bangladesh faces increasing concerns with antimicrobial resistance, there is no national guideline covering preferred antibiotics for SARI cases. Hospital Based Influenza Surveillance (HBIS) identifies SARI patients caused by different microorganism including influenza and other bacterial organisms.

Purpose We analyzed HBIS data to observe the antibiotic prescription pattern for SARI patients.

Methodology We analyzed HBIS data from May 2013 to December 2016 from 12 sentinel hospitals regarding length of hospital stay, chronic illness, different classes and generations of antibiotics prescribed to SARI patients. We used proportion test with 95% confidence interval to compare use of different classes of antibiotics.

Results Out of 9146 reported SARI cases, 66.3 % were male and 33.7% were female. Among them, 86.6% patients were prescribed antibiotics. Overall, proportion of cephalosporin use was the commonest (54.1%) followed by penicillin (27.3%), macrolides (13.2%) and fluoroquinolones (2.7%). Although use of penicillin was higher (43.5%) than cephalosporin (38.1%) in elderly patients ($p<0.05$). The third-generation cephalosporin was most commonly prescribed (91%). Antibiotic prescription was higher in patients who had no chronic illness (87.2%) than those with asthma (84.1%), hypertension (81.5%) and diabetes (81.1%) ($p <0.05$ for all comparison). SARI patients who were prescribed macrolides stayed in hospital for shorter period (median 3 days, 2-4 IQR) than those prescribed cephalosporin (median 4 days, 3-6 IQR) and penicillin (median 4 days, 2-6 IQR). In government hospitals cephalosporin was commonly prescribed (69.7%) than non-government hospitals (30.3%) ($p<0.001$).

Conclusions Newer generation cephalosporin was most frequently prescribed, which raises the concern of developing antimicrobial resistance. As patients given macrolides had a shorter hospital stay may be recommended for SARI patients. However, development of a national guideline for management of SARI patients including appropriate use of antibiotic is recommended.

Respiratory Diseases

Abstract Code: 2017-EOH-503

Presenter Name: Basma AbdElGawad
Country: Egypt
Presenter Email: basma.mostafa10@yahoo.com
Author: M. Fahim, A. Naguib, S. El Shourbagy, H. Abu El Sood, S. Refaey

Title Epidemiological Characteristics of Severe Acute Respiratory Illness Cases in Sentinel sites - Egypt, 2009-2017

Background Severe acute respiratory illness (SARI) is recognized as a leading cause of morbidity and mortality. SARI tends to be rapidly progressive illness caused by pathogens like influenza, which have pandemic potential. Egypt established SARI sentinel surveillance sites since 2007 in eight representative sites.

Purpose Describe epidemiology of SARI, identify influenza positivity and circulating influenza subtypes among SARI patients.

Methodology Patients who met standard WHO- SARI case definition (fever =38c + cough without any other cause in the last 10 days in a hospitalized patient) from 2009 to mid-2017, were enrolled. Epidemiological data were collected using standardized investigation form. Nasopharyngeal/ oropharyngeal swabs, for all patients, were tested for influenza viruses by reverse transcription polymerase chain reaction (RT-PCR). Data were extracted and analyzed by MS excel 2013.

Results From January 2009 to July2017, 22419 SARI patients were enrolled. The most affected age group was below five years with "5490(24.5%) of 22419". Median hospital stay was 5 days (IQR=3-7). During hospitalization, "159(0.7%) of 22419" were ICU admitted, out of them "65 (41%) of159" were ventilated. Of all SARI admissions, 18.3% (CI: 17.8-18.8) were positive for influenza viruses, 37.9% (CI: 36.4-39.4) were FluA/H1N1 Pdm09, 30.3% (CI: 28.9-31.7) were Flu B, 23.8% (CI: 22.5-25.1) were FluA/H3N2 and 7%(CI:6.3-7.8) were mixed influenza infections. Out of influenza-positive patients, fatalities were significantly higher ($P<0.001$) in pregnant women and those with preexisting chronic diseases, "41(5%) of 890" and "7(4.4%) of 158" respectively. Vaccination coverage was "13 (0.3%) of 4056" among influenza patients with zero fatality.

Conclusions Influenza viruses are frequent cause of SARI admissions, so developing strategies to control Influenza is a key stone to reduce SARI. Improving influenza vaccination coverage for risk groups will prevent some SARI cases who are associated with fatal outcome.

Respiratory Diseases

Abstract Code: 2017-EOH-66

Presenter Name: Dr. Mohammed Al Amad

Country: Yemen

Presenter Email: mohdalemad@yahoo.com

Title Severe Acute Respiratory Infections with Influenza and Non-Influenza Respiratory Viruses: Yemen, 2011-2016

Background Sentinel surveillance for severe acute respiratory infections (SARI) is an important tool to monitor influenza circulation and burden of other respiratory pathogens. In Yemen, two sites established at Sana'a and Aden city. Pharyngeal samples are tested for influenza and non-influenza by the Real-Time-PCR assay in NAMRU 3.

Purpose Describe severity of SARI as indicated by admission to intensive care unit (ICU) and fatality as well as associated influenza and non-influenza viruses among patients in the two sites to provide recommendations for improving SARI surveillance.

Methodology Data from 2012-2016 of SARI patients who admitted in the two sites based on WHO case definition was obtained from Ministry of Health, It analyzed by Epi info 7 and P value < 0.05 was the cut point for significance.

Results 2,211 patients were admitted in the two sites, 32% in 2013, 62% from Aden, 63% < two years, 20% had chronic diseases and 35% admitted to ICU. Overall SARI fatality was 8% which was significantly higher in Aden than Sana'a (10% vs. 5%, P <0.001), among patients with chronic disease (14% vs. 6.5% P <0.001) and admitted to ICU (10% vs. 7%, P = 0.04). Samples of 82% (1,811) patients were tested where influenza viruses (75% Type A) were detected in 5% (89) more in Sana'a than Aden (6% vs. 4%, P = 0.04) compared to 36% (655) of non-influenza viruses that included 43% (279) Respiratory Syncytial Virus and 17% (109) Adenovirus. The fatality of confirmed influenza was 9 % compared to 8% for non-influenza viruses.

Conclusions Our findings showed that children < 2 years are more affected by SARI. Both influenza and non-influenza viruses lead to mortality and necessitate prompt diagnosis and treatment. Expanding SARI surveillance to involve more hospitals at different governorate is recommended to give more comprehensive picture regarding SARI.

Respiratory Diseases

Abstract Code: 2017-EOH-434

Presenter Name: Dr. Basma AbdElGawad

Country: Egypt

Presenter Email: basma.mostafa10@yahoo.com

Author: S. Refaey, H. Abu El Sood, S. El Shourbagy, A. Mohsen, M. Fahim

Title Defining Influenza Baseline and Threshold Values Using Surveillance Data - Egypt, Season 2016-17

Background Influenza infection represents a substantial public health problem resulting in global burden of mortality and morbidity. Influenza thresholds indicate level of disease activity that would signal the start or end of a season and provide an alert to an unusually severe or atypical season so, adjust preventive and control measures.

Purpose To establish baseline and threshold values for 2016/17 season.

Methodology Using Acute Respiratory Illness (ARI) surveillance data from 2013 to 2017, two parameters were assessed to monitor influenza activity: percentage of ARI samples positive for influenza and composite parameter (percentage of samples tested positive *ARI rate). Three threshold levels (baseline, alert and epidemic) were established by calculation of average of each week in all preceding seasons, 40% Upper Confidence Limit (UCL) and 90% UCL of each week respectively, then a four-week running average used to smooth the curve. Each parameter was compared against corresponding threshold and transmission intensity was categorized as low, moderate and high.

Results For season 2016/2017, both parameters showed two waves of activity crossing baseline threshold. First started at week 35 to 45 with dominance of Flu A/H3 activity (293(89) % of 329 positive samples, remain was Flu B) that exceeds epidemic threshold. The other, started week 12 to 14 with dominance of Flu B activity (136(99) % of 138 positive samples, remain was Flu A/H3). Percentage positive parameter signaled other weeks away from the defined season.

Conclusions Public health actions were taken in response to the observed increase flu A/H3 activity, to trim the impact and serious consequences of the disease. Continuous calculation of baseline and threshold levels can assess not only seasonal influenza but also potential pandemic influenza, contributing to the country's pandemic preparedness and have important implications especially for resource-limited countries.

Respiratory Diseases

Abstract Code: 2017-EOH-111

Presenter Name: Dr. Yousaf Ali Khan

Country: Pakistan

Presenter Email: yousafalikhan37@gmail.com

Title Constrains Faced by Tb Patients Leading to Non-Compliance: A Cross Sectional Study in Mardan, Kpk, Pakistan

Background Pakistan is among the high endemic countries for TB and ranked 5th in the high TB burden countries with estimated 4th highest prevalence of multidrug resistance (MDR) TB. TB patients in Pakistan are facing different socio-economic and cultural constraints. TB patients are stigmatized and have been affected negatively due to poor knowledge about the disease dynamics, wrong socio-cultural myths and misapprehensions in general public.

Purpose The study conducted by active TB patients to explore and evaluate different constraints that TB patients are facing in Mardan, Pakistan.

Methodology A cross sectional study was conducted in district Mardan during March to June 2015. From 350 selected patients 210 were enrolled in study after informed consents. Data were collected though structured questionnaire and statistically analyzed by Epi-info/SPSS.

Results Overcrowding (p = 0.001, CI, 95%), unawareness of disease (86%), low educational status (p < 0.002, CI 95%), poverty and access to healthcare facilities were directly related to poor compliance. Attitude of family members, colleagues, society and even healthcare staff (p = 0.002, CI 95%) were also found significant. Age groups, marital status and treatment duration were found to be highly significant (p < 0.002). 85% patients were unaware with the risk factors and precautions during the treatment. 42% patients were unemployed, 58% employed less than 200 USD/month (p = 0.001, CI 95%). 59% patients complained worst behavior of their colleagues (p = 0.001) and 41.7% complained worst behavior of healthcare staff (p = 0.003. CI 95%).

Conclusions TB patients were found stigmatized due to poor economic conditions and bad attitude of family, colleagues and healthcare staff. Unemployment, malnutrition and overcrowding were among the worst constraints. Sensitization of medical staff and doctors to diagnose the disease in time and behave properly with patients is recommended. Dedicated patients and family education sessions must be conducted.

Respiratory Diseases

Abstract Code: 2017-EOH-202

Presenter Name: Dr. Fahad Al-Jasser

Country: Saudi Arabia

Presenter Email: dr.f.j@hotmail.com

Author: R. Nooh, R. Youssef

Title Epidemiology and Predictors of Survival of MERS-CoV Infections in Riyadh Region, 2016

Background MERS-CoV emerged as a zoonotic disease in Saudi Arabia with 1437 cases as of July 2016.

Purpose This study aimed at describing the epidemiology of MERS-CoV infection, clinical aspects of the disease and the determinants of survival.

Methodology Medical records were reviewed between April 2014 and December 2015 to identify admission and discharge with MERS-CoV. Patient's characteristics, epidemiologic and clinical data and laboratory results were extracted and described. Logistic regression analyses were used to model the determinants of the survival of these patients. Significance of the results were judged at the 5% level.

Results 249 laboratory confirmed cases were admitted mostly in August (20.48%) and September (14.86%) of the year 2015. These cases were non-healthcare workers (85.14%), Saudi (58.63%), males (57.03%) with a mean age of 46.71±17.92 years. A third (39.36%) reported contact with suspected or confirmed cases, developed the disease after 6.2 days and continued to shed the virus for 13.17 days on average. The case fatality rate was 20.08%. The likelihood of being discharged alive was significantly higher among non-Saudi (OR=2.35), healthcare workers (OR=10.822), with no co-morbidities (OR=6.57).

Conclusions MERS-CoV mortality is higher among older patients with severe disease. Further studies are recommended for a better estimation of the incubation period and the period of communicability and the role of animal reservoir.

Respiratory Diseases

Abstract Code: 2017-EOH-108
Presenter Name: Dr. Abdullah Al-Guaimy
Country: Saudi Arabia
Presenter Email: doctorabduallah@gmail.com
Author: S. Kamel

Title MERS-CoV Outbreak at Domat Al-Jandal Hospital

Background Saudi Arabia was first to report MERS-CoV in the middle east region in 2012. Several outbreaks had occurred since that time and still occur. In August,2017 an outbreak of MERS-CoV at Al-Jouf province in Saudi Arabia possibly linked to an index case who admitted to hospital while infectious.

Purpose A team of Saudi field epidemiology training program was responsible to investigate the outbreak, to determine causes and to prevent recurrence.

Methodology List of cases were obtained from hospital administration. Information was collected by interviewing infection control team, outbreak team at hospital, local MERS-CoV coordinator and by observing most relevant sections at hospital.

Results A total of 13 cases of MERS CoV infection were reported at Domat Al-Jandal hospital. Of these 13 cases, 8 cases were health care workers (3 physicians and 5 nurses), 3 cases were contacts cases of the index case. Most of cases acquired infection by person to person transmission at male medical ward and intensive care unit, only 3 contacts cases may get infected when they brought the primary case to hospital. Attack rate among physicians was 12% and among nurses was 9.8%. We found that late diagnosis, improper isolation of patients and non-compliance on infection control protocols are the leading causes of spread of the infection.

Conclusions Sorting and examining patients carefully at triage and emergency before admission to hospital, adhering to infection control protocols, applying effective isolation measures are a must in the way to stop or prevent any MERS-CoV infection at hospitals.

Respiratory Diseases

Abstract Code: 2017-EOH-169
Presenter Name: Dr. Jassim Almogrin
Country: Saudi Arabia
Presenter Email: aboadnan313@hotmail.com

Title Assessment of Awareness Towards Respiratory Infections Among Qatif District Pilgrims During Hajj 2017

Background Hajj is one of the largest human mass gatherings. Due to crowding, there is the potential for respiratory infections to spread among attendees. Health education and vaccination for respiratory infections are the main preventive measures taken for Saudi Arabian pilgrims prior to hajj.

Purpose To describe knowledge gained from preventive programs in Qatif district in Saudi Arabia's eastern province. This information can improve the health educational programs for Saudi pilgrims.

Methodology We randomly sampled pilgrims from 39 troops distributed across Qatif district. Questionnaires assessed pilgrim's awareness towards respiratory infections during hajj and to identify the factors influencing pilgrim's awareness about MERS-CoV in Saudi Arabia. Data analyses were completed using Epi Info 7.

Results We surveyed 400 pilgrims from five different troops. Most (94%) were Saudi, 71% were between 20-40 years of age. For vaccinations, 75% of pilgrims were vaccinated against influenza, 87% for meningococcal, and 4% for pneumococcal. Half (50%) of pilgrims correctly identified drug effectiveness against respiratory infections. For knowledge, 85% of pilgrims identified sneezes and cough products as a cause of infections and contact with ill persons by 72%. Most pilgrims considered cough (65%), dyspnea (58%), sneeze (60%) and fever (56%) as common symptoms. For preventive measures, 91% of pilgrims identified face masks, 86% identified frequent hand washing and 85% identified social distancing as effective. MERS-CoV was identified a serious disease by 76%, and 30% said MERS-CoV was treatable. For transmission of MERS-CoV, 75 % identified airborne transmission and 60% identified consuming infected camel. Only 55% of pilgrims correctly responded that MERS-CoV is still circulating in the kingdom.

Conclusions Pilgrim knowledge about causes, symptoms, and preventive measures for respiratory infections were acceptable, but knowledge towards respiratory illness treatment and MERS-CoV were poor. We recommend national health education programs target these areas in the future.

Surveillance System

Abstract Code: 2017-EOH-456
Presenter Name: Dr. Mohammad Afzalur Rahman
Country: Bangladesh
Presenter Email: drafzalur1981@gmail.com
Author: M. Flora, M. Rahman, M. Billah

Title Food-borne Illness Surveillance and Etiology of Diarrhea in Bangladesh

Background In Bangladesh, yearly 30 million people are affected by foodborne illnesses caused by Rotavirus, Vibrio cholerae, Escherichsia coli, Shigella and Salmonella. Since 2013, the country has been conducting active foodborne illness surveillance (FBIS) in ten sentinel sites to collect epidemiological data and stool samples of acute watery diarrhea (AWD) cases.

Purpose To describe the microbiological findings of stool samples and their relation with epidemiological data of FBIS.

Methodology We described AWD cases reported in FBIS from 2014 to 2015 by age, causative organisms and analyzed cholera cases by behavioral risk factors and geographic area. FBIS defined AWD cases as >3 episodes of loose stools or <3 loose stools causing dehydration or at least a single episode of bloody loose stool in 24 hours among >2 months of age, and watery stools that changed from usual pattern and frequency among <2 months of age. To find statistical association, we performed z-test for two proportions.

Results Among 4,064 enrolled AWD cases, stool culture identified 8% of cases as cholera, 3% ETEC, 2% shigella, and 1% salmonella. Microbiological test could not identify any specific organism in 86% of samples. Median age of cholera cases was 27 years (IQR: 18-40), ETEC 23 years (IQR: 1.7-42.5), salmonella 36 years (IQR: 25-50), shigella 19 years (IQR: 1.9-40), and no-organism 2.5 years (IQ: R0.9-33). Both proportions of cholera (23%) and taking food from roadside vendors (33%) were higher in 5-14 years age group. In Chittagong district, both proportions of cholera (26%) and drinking well water (21%) were higher than other districts. Cholera was associated with taking food from roadside vendors (p=0.04) and drinking well-water (p<0.01).

Conclusions As the majority of stool cultures could not identify causative organisms, we recommend increasing the microbiological identification capacity of FBIS. We also recommend conducting studies to identify true association of cholera with taking food from roadside vendors and drinking well-water.

Surveillance System

Abstract Code: 2017-EOH-346
Presenter Name: Dr. Eisha Mansoor
Country: Pakistan
Presenter Email: dreishamansur@gmail.com
Author: N. Azam

Title Rising HIV Seroconversion Rates and Associated Risks Among Civilian Employees of Pakistan Army: A Case Control Study - Pakistan, 2017

Background In 2004, Pakistan escalated from 'low-prevalence' to 'concentrated' phase of HIV epidemic. Despite global decline in HIV incidence since 1997, rate of HIV infections in Pakistan is persistently rising since 1990. Available literature focuses on key populations or localized outbreaks and is limited by short study duration and regional applicability of results.

Purpose We studied HIV seroconversion trends over a period of 8 years in a geographically diverse population and evaluated associated risk factors.

Methodology A desk review of HIV surveillance data from 2010 to 2017 was carried out at Armed Forces Institute of Pathology. A case was defined as any adult enrolled in Pakistan Army as civilian employee, initially screened for HIV but later seroconverted on ELISA and western blot. Case-control study was conducted on cases diagnosed in 2017. Age and sex matched controls were identified from same population sub-group. Structured telephonic interviews were conducted, and statistical analysis done at 5% margin of error.

Results From 2010-2017, 109 cases of HIV were notified from 75,000 HIV negative civilian workers. Annual case count remained <12 till 2016 when it rose to 24. Upward trend continued in 2017 with 34 cases reported to date (183% increase from baseline). Acquisition of HIV was significantly associated with commercial sex activities (OR=5.71; 95%CI: 1.25-395). No statistically significant association was found for blood transfusion, surgical/dental procedure, barber shops visits, piercing or substance abuse.

Conclusions HIV seroconversion rates among Pakistan Army civilian employees have increased significantly in past 2 years. Unlike HIV outbreaks previously reported from Pakistan, sexual route has been recognized as the predominant mode of transmission. Consequently, sex education sessions were conducted in all formations and a section on prevention of STIs was included in Pak Army health pamphlet. Similar focus is mandated on prevention of sexual transmission of HIV at national level as well for all vulnerable populations.

Surveillance System

Abstract Code: 2017-HIV/STI-436

Presenter Name: Dr. Fawad Khalid Khan

Country: Pakistan

Presenter Email: fawadkhan786@hotmail.com

Author: M. Baig, M. Najeeb

Title Evaluation of Dengue Surveillance System – Islamabad, 2017

Background Dengue is a significant public health problem affecting 50% population worldwide. Every year 50-100 million cases of DF while 250000-500000 cases of DHF are reported worldwide. Mortality rate of DHF/DSS is 5-10%.

Purpose The study was conducted to evaluate the system in terms of its core functions, system attributes and challenges faced in order to make recommendations for improvement.

Methodology This evaluation was conducted during November 2017 at Islamabad District. A desk review of literature, departmental reports and documents was conducted. Quantitative and qualitative system attributes were assessed using Updated CDC Guidelines for Evaluating Public Health Surveillance Systems, 2001. Stakeholders were identified and interviewed. A semi structured questionnaire was used to collect data.

Results Staff was trained in data collection and data entry. NS1 (Non-structural protein1) and dengue-specific IgM antibody test were available at all tertiary care hospitals to confirm diagnosis. Case definition was simple and strictly followed. Data flow was easy. System is less flexible but able to integrate with other systems. Quality of data was poor as 80% of filled forms were incomplete in demographic and clinical profile. Acceptability was good due to sense of ownership and good coordination among all stakeholders. Sensitivity was 27.6% and predictive value positive was 81.5%. Representativeness was poor, covering only tertiary care hospitals. Timeliness was excellent with daily reporting and case response within 24 hours. The system is useful as it provides dengue fever data base for planning and management purpose. System is stable, secure and available when required.

Conclusions The evaluation shows the performance of dengue surveillance system was good overall. System is not representative but has ability to detect and respond to outbreaks within time. Expansion of the coverage to include all public and private healthcare facilities is needed. Regular data collection trainings are recommended. Feedback mechanism is necessary to ensure data quality.

Surveillance System

Abstract Code: 2017-HIV/STI-400

Presenter Name: Dr. Abdul Razziq

Country: Pakistan

Presenter Email: Dr.abdulrazziq@outlook.com

Author: A. Saeed

Title Evaluation of Malaria Surveillance System in Balochistan- 2017

Background Globally, an estimated 212 million cases of malaria were reported in 2015. In Pakistan, health facility-based confirmed cases were reported to be 0.2 million. Total reported deaths were 34 as compared to estimated 740 deaths. Malaria information system (MIS) detected 0.084 million confirmed cases of malaria in 2016, in province Balochistan.

Purpose An evaluation of Malaria Surveillance System in Balochistan was conducted to identify strengths and weaknesses and make recommendations for improvement.

Methodology A desk review of literature, office records and reports were conducted in Provincial Malaria directorate Quetta from March to May 2017. Evaluation was conducted for the year 2016. Assessment of qualitative and quantitative system attributes was done using the framework of updated CDC guidelines for evaluating public health surveillance systems, 2001. A semi-structured questionnaire was used for interviews. Stakeholders were identified and engaged.

Results Case definition was simple, and system was easy to operate but inflexible in accommodating additional information like outbreaks. Data quality was assessed as poor, because on random evaluation 30% forms were found completed. Timeliness was also poor as report took 2 months to reach Malaria directorate from district level. system had good acceptability, stability and representativeness. sensitivity of the system was excellent 100% whereas PPV was 12.8%.

Conclusions New approach of Malaria Control Program should be vector control rather than malaria control because coastal areas of Balochistan are at high risk for Dengue, Chikungunya, Zika and Yellow fever as these have common vector for spread. A software is strongly recommended for reporting of malaria cases which will improve the timeliness of reporting. During transmission season active case finding and taking blood samples from affected people would be highly effective in picking cases at right time for better management. All the staff working for malaria control and prevention should be trained for other vector borne diseases as well.

Surveillance System

Abstract Code: 2017-HIV/STI-328
Presenter Name: Dr. Fatma Osman
Country: Egypt
Presenter Email: msmfatma1976@gmail.com
Author: A. Kandi, S. Elrefaey, S. Elshourbagy, H. Abuelsoud, M. Taha, A. Gehad

Title Enforcement of Functionality and Effectiveness of Event-Based Surveillance System (EBS), Egypt ,April-September 2017

Background To meet the requirements of International Health Regulations 2005 (IHR), initiation of EBS is essential to complement the Indicator Based Surveillance (IBS) and to boost early detection of potential Public health threats. In November 2015, the Ministry of Health (MoH) launched the EBS at the central level. The hot line in emergency room and active browsing for electronic media are the main sources of information.

Purpose To enhance early detection and rapid response to potential public health threats and to improve the performance of the EBS team.

Methodology At the end of June 2017, the reporting form, database and the standard Operating Procedures (SOP's) of EBS were updated. EBS team received training for the system updates. Data from April to September was investigated for the source of information, signal filtration, verification, and timeliness of response. For the events that are routinely reported by IBS, early detection was checked for both systems. The results were compared three months before and after the training.

Results Out of 762 raw signals, 199 events were detected, from which 65% (130/199) events after June. Proportion of events captured by electronic media, health care workers and community were 75% (150/199), 20% (40/199) and 5% (9/199) respectively. Filtration of signals varied significantly from 14 % to 51% (P value < 0.0001). Verification improved from 78% to 86 % (P value= 0.154). Positive Predictive Value (PPV) significantly differed from 80% to 93% (P value = 0.012). Rapid response within 24 hours significantly improved from 53% to 88% (P value < 0.0001). Capability of EBS to capture events Preceding IBS improved from 27% (9/33) to 38% (32/85) (P value= 0.288).

Conclusions Increase EBS capacity for early detection of potential public health threats. Marked improvement of EBS team performance. Raise community awareness, expand the sources of information and shift to electronic database are highly recommended.

Surveillance System

Abstract Code: 2017-HIV/STI-313
Presenter Name: Dr. Mohamed El-sayed Abdel-Aziz Abdel-Aty
Country: Egypt
Presenter Email: dr.mohammed.abdelaty@gmail.com

Title Evaluation of Knowledge, Attitude and Practices of Adverse Drug Reaction Reporting Among Hospital Pharmacists-Mansoura, Egypt, 2016-Cross Sectional Survey

Background Adverse drug reactions (ADRs) are a major cause of drug related morbidity and mortality. Globally, about 5% of all hospital admissions are due to an ADR and 10%–20% of inpatients have at least one ADR during their hospitalization. Pharmacovigilance is the science and activities relating to the detection, assessment, understanding and prevention of adverse effects or any other drug-related problem(s).

Purpose To evaluate the knowledge, practice and attitudes (KAP) toward ADRs reporting and pharmacovigilance among hospital pharmacists in Mansoura, Egypt, 2016.

Methodology A cross-sectional study was conducted among random sample of hospital pharmacists in Mansoura, who were invited to attend a workshop on Pharmacovigilance. A structured pilot tested questionnaire was developed based on the previous literature. The questionnaire was distributed to the attending pharmacists before and after the workshop. Collected data was analyzed using SPSS v.16.0.

Results Of the total 200 invited pharmacists, 95% attended and agreed to participate in the study. The majority were females (87%), with mean age 30.6±4.2 years. Before the workshop, the average percentage of correct answers on the knowledge items was 55% indicating a fair to poor score which had been enhanced after the workshop (90%). The majority of pharmacists had positive attitude toward Pharmacovigilance and agreed that ADRs reporting should be mandatory, prompt and online but without legal penalties on the reporter. According to their practice, the most reported ADRs were severe serious reaction (22.5%), unexpected reaction (21.5%) and unlabeled reaction (17.5%). Besides, the most barriers to ADRs reporting were uncertainty whether the reaction is actually due to ADR (25%), already well-Known ADR (15.5%) and forgetfulness (14.5%).

Conclusions Awareness campaigns, incorporation of pharmacovigilance in health education, mandatory online ADRs reporting system will greatly enhance implementation of pharmacovigilance in Egypt.

Surveillance System/VPD

Abstract Code: 2017-HIV/STI-468
Presenter Name: Dr. Shereen Elghazaly
Country: Egypt
Presenter Email: drshereen84@yahoo.com
Author: H. Abu El Sood, S. El Shorbagy, S. Refaey

Title Evaluation of National Electronic Disease Surveillance System (NEDSS) Regarding Typhoid Fever in Sharkia governorate, Egypt, 2016

Background Typhoid fever is a systemic infection caused by Salmonella Typhi. It is still common in the developing world, where it affects about 21.5 million people each year, 222 000 typhoid-related deaths occur annually worldwide. It can be prevented and can usually be treated with antibiotics. Cases are reported through National Electronic Disease Surveillance System (NEDSS) from all healthcare facilities to department of Epidemiology and Surveillance.

Purpose To identify strengths and weaknesses of surveillance system of reported typhoid cases for improvement and disease control.

Methodology The evaluation was conducted using CDC guidelines for evaluating public health surveillance. NEDSS data regarding Typhoid from 1st January 2016 to 31 December 2016 were extracted on an excel sheet for calculating completeness and timeliness. A structured questionnaire was used to assess the following attributes: Usefulness, Simplicity, Acceptability, and Stability. The assessment was implemented on four levels: central, Health directorate, Health district, Fever hospital levels. Sharkia governorate had been selected which was the highest governorate in reporting typhoid cases. Data were analyzed using Microsoft Excel.

Results According to surveillance officers in all levels which interviewed (n=10), the mean of system usefulness, simplicity, acceptability and stability was 92.5%, 98.8%, 94%, and 85.5%, respectively. The mean completeness of some variables (Outcome, occupation, national ID, address, final diagnosis) was 75.9%. It takes 2 minutes for case / day for data entry and 27minutes/ month for data analysis. Median time between electronic Insertion to Reporting and admission to Notification date was 1 day for both, IQR (3-1) and (2-1) respectively. Positive predictive value was 50%. Satisfaction of surveillance team was 100%.

Conclusions The system is simple, flexible, stable, acceptable and useful for the surveillance team which is satisfied with their job, the timeline of reporting is considered acceptable in between different levels and Lack of laboratory confirmation of cases (use tube agglutination test only).

Surveillance System/VPD

Abstract Code: 2017-EOH-205
Presenter Name: Dr. Kamal Kadhim
Country: Iraq
Presenter Email: kamalabdrazaq@yahoo.com
Author: F. Lami

Title Prevalence and Seroconversion of Viral Hepatitis B and C and HIV Among Hemophilia Patients in Baghdad, Iraq, 2016

Background Hemophilia is an inherited bleeding disorder caused by a deficiency of either factor VIII (hemophilia A) or IX (hemophilia B). Treatment with intravenous replacement of these factors and blood carries the risk of transfusion transmitted viral infections.

Purpose We performed this study to estimate the prevalence and seroconversion rates and identify risk groups of hepatitis C (HCV), hepatitis B (HBV) and human immunodeficiency virus (HIV) infections among hemophilia patients in Baghdad City, Iraq, 2016.

Methodology We conducted this cross-sectional study by reviewing records of all hemophilia patients resided in Baghdad in 2016 and registered and received treatment in the four hemophilia centers in Baghdad. All hemophilia patients are annually screened for anti-HCV antibody, HBsAg and HIV antibodies. Positive samples are sent for confirmation at the Central Public Health Laboratory and the results are reported in the patients' records.

Results The total number of registered hemophilia patients in Baghdad in 2016 was 639. There were 150 (22.9%) patients with HCV infection, six (0.9%) with HBV infection, and only one patient (0.2%) had HIV infection. The seroconversion rate for HCV was 8/1000 and for HBV was 1.7/1000. The median period between birth and acquiring HCV infection was 17 (IQR=24) years, and for HBV was 11.8 (IQR=9.4) years. Binary analysis, revealed the following statistically significant risk factors ($P<0.05$) for acquiring viral hepatitis infection: age, severity of hemophilia, presence of inhibitors, type of treatment, no. of treatment products used and presence of target joints. After applying logistic regression analysis, the significant independent risk factors were: age (14-18years: OR=4.03; 95%CI: 1.25-12.94), (19-44years: OR=18.8; 95%CI: 6.69-52.85), (=45years: OR=5.18; 95%CI: 1.01-26.58) and severe hemophilia (OR=6.25; 95%CI: 1.27-31.25).

Conclusions Despite screening of blood and blood factors, HBV and HCV infections still occurring in hemophilia patients. Closer monitoring of transfused blood and ensue vaccination of all hemophilia patients for HBV are recommended.

Surveillance System/VPD

Abstract Code: 2017-OTH-75
Presenter Name: Dr. Ilham Dahbi
Country: Morocco
Presenter Email: dahbiilham@gmail.com
Author: I. Khoudri

Title The Epidemiological Profile of Viral Hepatitis B and C in Morocco Between 2013 and 2016

Background Hepatitis B and C virus infections (HBV/HCV) are a global health problem. Morocco is considered as a middle endemic country. HBV/HCV surveillance system was implemented in Directorate of Epidemiology by the Ministry of Health as an ongoing process of case investigation, data collection, analysis and also dissemination of data to public health professionals.

Purpose The aim of this study was to analyze the epidemiological profile of HBV/HCV infections during a four-year period.

Methodology This was a descriptive and retrospective study of HBV/HCV cases registered from 2013 to 2016 by 22 referral centers for viral hepatitis in Morocco. Registration of new cases was reported monthly via a reporting form using WHO case definitions. Socio-demographic characteristics, diagnostic circumstances, modes of transmission, clinical and biological data of suspected or confirmed cases were collected.

Results A total of 1160 HBV/HCV cases were registered (95% HCV vs 5% HBV). Male/female ratio was 0.52 and 48% were married. Free Medical regimen was available in public health services for 90% of patients. HCV cases came mostly from two northern regions: Rabat (24%) and Casablanca (21%). Regarding diagnostic circumstances, cases were identified during screening (25%), systematic blood check (21%), cytolyse (18%) or during blood exams before surgery (13%). Infections were frequently transmitted during dental care (58%), followed by transfusion-transmitted infections in 8% of cases and sexually transmitted in 4% of cases. In 80% of cases, HCV were active. The presence of detectable HCV RNA sequences, 54% were Genotypes 1 and 43% were genotype 2.

Conclusions This analysis is from the data of surveillance system recently implemented. It allowed us to have an idea about the epidemiological profile of HCV/HBV in Morocco. However, these results point out the necessity to reinforce the surveillance system by involvement of more different regions in order to report more cases, and also the computerization of notification process.

Surveillance System/VPD

Abstract Code: 2017-HIV/STI-9
Presenter Name: Dr. Touria Essayagh
Country: Morocco
Presenter Email: essayagh.toula@gmail.com
Author: A. Khattabi, S. Essayagh, M. Khouchoua

Title Evaluation of the Meningitis Surveillance System in Meknes, Morocco

Background Meknès is a big city of Morocco with 860.972 population in 2016. It includes 47 health centers, 5 hospitals and 2 laboratories which one is implicate in meningitis surveillance. The Meningitis Surveillance System (MSS) was implemented in Meknes in 1995.

Purpose The objective is to evaluate the MSS and to identify its strengths and its potential gaps for its improvement.

Methodology The evaluation was based on the CDC guidelines, 2001 for evaluating surveillance systems. A questionnaire was structured and tested to evaluate simplicity and acceptability. Twenty health professionals at the prefectural epidemiology unit, the hospitals and the laboratory were interviewed. MSS Data across 2012-2016 were analyzed to evaluate the representativeness, reactivity and quality of the data.

Results 207 cases were reported with an incidence ranged from 4 per 100.000 population in 2012 to 5 per 100.000 in 2016. 172 (183%) of the cases reside in urban areas and 183 (88%) were from the public sector. The Completeness of selected variables was 89% (184/207). The system was simple regarding structure and all interviewed staff considered the system acceptable. Of 32 confirmed meningococcal meningitis cases, 15 had a serotype confirmation result. The epidemiological investigation of the case after declaration was carried out on average on the day of the declaration.

Conclusions The evaluation of the MSS has demonstrated its strengths, namely good data quality, acceptability and responsiveness. However, these weaknesses reside in the low proportion of the serotype of meningitis agents. These results suggest the need to consolidate achievements and reinforce the importance of routine meningococcal serotype according to clinical and laboratory best practices.

Surveillance System/VPD

Abstract Code: 2017-HIV/STI-275
Presenter Name: Dr. Rafal Altalib
Country: Iraq
Presenter Email: altalibrafal@gmail.com
Author: F. Lami, A. Al-Safi

Title Assessment of Competencies of District Surveillance Officers, Iraq, 2017

Background Communicable diseases (CDs) surveillance is vital for early detection of outbreak and reduce CDs burden. Surveillance officers at the grassroots up to the highest level should be adequately equipped with field epidemiology competencies. In Iraq, district surveillance officers (DSOs) are responsible for compiling, analysis of CDs data from Primary Healthcare Centers (PHCs) in their district and forwarded to the provincial level.

Purpose to assess background characteristics, qualifications and competencies of DSOs in Iraq.

Methodology In this cross-sectional study, we involved all DSOs (136) in Iraq. We developed a structured questionnaire through a meeting involved central and provincial officers and academia. The questionnaire gathered data on demographics, service characteristics, status of competencies in basic epidemiology, biostatistics, surveillance, outbreak investigation, rapid response to health incidence, laboratory models, developing scientific report and basic computers skills. Questionnaires were sent and received from DSOs via e-mails.

Results The response rate was 85.3%. About half of DSOs was responsible for <10 PHCs, while only 13.8% were responsible for 20-29 centers. Also, about 55% were responsible for 1-2 hospitals and only 10% were responsible for 3-4 hospitals. Males constituted 78.4%. The highest academic degree of 63.4% was a two years Diploma after high school. The proportion of previous training of the DSOs was: basic surveillance 69.8%, outbreak investigation 34.5%, basic epidemiology 26.7%, rapid response to health incidents 21.6%, laboratory models 19.8%, biostatistics 13.8% and scientific reporting 8.6%. DSOs who didn't attend any training activity were 15.6%. Cholera and food poisoning were the most frequently reported incident and outbreak investigated (53.8%, 43.8%, respectively). Microsoft word was the most frequently reported computer skill (48.3%).

Conclusions Although DSOs are the backbone in CDs surveillance, their technical capacities were sub-optimal. We started training of 25 DSOs (as first cohort) using a 3-months frontline FETP model.

Surveillance System/VPD

Abstract Code: 2017-HIV/STI-36
Presenter Name: Dr. Inayatur Rehman
Country: Pakistan
Presenter Email: drinayat.na43@gmail.com
Author: M. Saleem

Title Evaluation of Acute Flaccid Paralysis (AFP) Surveillance System in Bajaur Agency, Federally Administrated Tribal Areas (FATA), Pakistan During 2015

Background Federally Administered Tribal Area (FATA) remains a major reservoir and source of transmission of polio to other parts of the country and abroad. Terrorism, war against terror and anti-polio activities from 2008-2014 in many parts of FATA have badly affected Acute Flaccid Paralysis (AFP) surveillance system. FATA contributed 70% (n=65) and 58% (n=179) in 2013 and 2014 respectively. Bajaur Agency shares borders with Afghanistan and Mohamed Agency due to which it remains a major reservoir of polio virus and has contributed 18 cases in 2010.

Purpose The Purpose of this evaluation was to identify strengths and weaknesses of the system to formulate recommendation.

Methodology CDC's updated guidelines for evaluating public health surveillance system 2011 were followed. Descriptive study was conducted in May 2015. Major stakeholders were identified and interviewed through Semi-structured questionnaire. Qualitative & quantitative assessment of AFP surveillance system attributes were done. Sensitivity and PPV was calculated using previous years estimates for FATA.

Results System was found simple, stable and flexible. Representativeness was found average as not covering private sector. Sensitivity was 100% while predictive value positive was 48%. Cases with adequate stools were 81% (n=128). Completeness and timeliness of reports were 87% (n=26). Case investigations within 48 hours of report was 99% (n=156). Stool specimens collected within 14 days of paralysis onset were 81% (n=128). AFP cases with 60 days follow-up were 36% (n=58). Lack of ownership and accountability, poor data management and analysis, inadequate logistics and feedback were identified as major weaknesses.

Conclusions Health care provider's knowledge regarding AFP was found inadequate and needs regular capacity building. There was lack of government ownership and this system needs official ownership at all levels in order to bring improvements. Human resources and logistics provision needs to be ensured. Regular surveillance data analysis and feedback to health workers is recommended.

Surveillance System/VPD

Abstract Code: 2017-HIV/STI-321

Presenter Name: Dr. Fatima Zerriouh

Country: Jordan

Presenter Email: toom832016@gmail.com

Author: M. Abdallat, L. Ghaffari, I. Iblan, K. Abusal, N. Sabri, Y. Khader

Title Evaluation of Acute Flaccid Paralysis Surveillance System in Polio-Free Jordan, 2012–2016

Background As part of polio eradication strategy, World Health Organization (WHO) has established a global Acute Flaccid Paralysis (AFP) surveillance. In Jordan, AFP surveillance has succeeded to achieve the polio-free certification. However, there is a substantial risk of polio importation from neighboring countries including Syria and Iraq.

Purpose This study aimed to evaluate the AFP surveillance in Jordan to ensure that the system operates effectively in order to maintain the polio free status.

Methodology This retrospective study was based on secondary analysis of data routinely collected between 2012 and 2016 by the Jordan's Expanded Program on Immunization (EPI). All AFP cases reported to the EPI during this period were included. The WHO minimum performance indicators were used to evaluate the quality of AFP surveillance. Data were analyzed using the data management system for AFP surveillance data and excel 2010.

Results Cumulatively, 328 AFP cases had been reported during the study period. Almost half (51.3%) of cases aged between one and five years and 55.8% were males. All cases had been discarded. The most common cause of AFP was Guillain-Barre Syndrome (35.1%). Annualized non-polio AFP rate increased from 1.4/100,000 population under 15 years in 2012 to 4.3 in 2016. National and subnational sensitivity was not met in 2012 and 2013. Adequacy of stool specimens and timeliness of specimens arriving and processing in the laboratory were constantly above the WHO minimum target. Timeliness of investigation met the expected target but with a decreasing trend. The proportions of stool specimens where non-polio enterovirus (NPEV) was isolated were below the WHO minimum target except in 2016.

Conclusions AFP surveillance system in Jordan is performing well, however additional efforts are needed to strengthen the subnational sensitivity. Moreover, laboratories are required to increase the NPEV isolation rate. Providing feedback to the reporters is recommended to maintain the reporting and investigation above the target.

Surveillance System/VPD

Abstract Code: 2017-HIV/STI-428

Presenter Name: Dr. Waqar Dahar

Country: Pakistan

Presenter Email: daharwaqar@yahoo.com

Author: N. Memon

Title Evaluation of Acute Flaccid Paralysis (AFP) Surveillance System in Sukkur Division - Sindh Province, Pakistan, 2016

Background Pakistan is among three countries in the world with ongoing wild poliovirus transmission. Pakistan established an AFP Surveillance system in 1995 and in 2000 further technical strengthening was provided to system at all levels by WHO. Sindh reported 8 confirmed cases of poliomyelitis in 2016 and 3 cases were from Larkana Division. An adjacent division, Sukkur, is a big reservoir for poliovirus and an intersection between three provinces.

Purpose The evaluation was conducted to identify the gaps in the surveillance system and suggest recommendations for improvement.

Methodology A descriptive study was conducted during November-December 2016. The study setting was Sukkur Division. Quantitative and qualitative attributes of the system were assessed using Updated CDC Guidelines for Evaluating Public Health Surveillance Systems, 2001. A desk review of literature, departmental reports and records was undertaken. Major stakeholders were identified and interviewed using a semi-structured questionnaire. Sensitivity and PPV were calculated by using WHO estimates of Sindh Province for previous year.

Results Case definition used was simple and gathered all essential information. System demonstrated its flexibility by incorporating information on other diseases like measles and neonatal-tetanus (NNT). The System captured all cases from 3 districts of the Sukkur Division. Data quality is good with 90% of the reports being completely and correctly filled and timeliness of data sharing was excellent with 85% reports reaching the provincial level weekly. Representativeness is average as system has limited coverage of private sector. System sensitivity is 100%, whereas PVP is 0.087%. System functions showing good stability without any disruption.

Conclusions Polio is marked for eradication hence the system is fulfilling its objectives. We recommend coverage of the system to be extended to private sector and report sharing at directorate level. Regular capacity building of the staff is recommended for maintaining the quality and timeliness.

Surveillance System/VPD

Abstract Code: 2017-HIV/STI-396

Presenter Name: Dr. Nadia Noreen

Country: Pakistan

Presenter Email: nadia.jamil3@gmail.com

Title Evaluation of Lab-based Influenza Surveillance System in Pakistan, 2017

Background Globally 5-10% of adults and 20-30% of the children are affected by influenza annually. Annual epidemics results in 3-5 million cases and 500,000 deaths. Influenza is a common illness in Pakistan however absence of a robust surveillance system makes assessment of burden of disease an issue.

Purpose The study was conducted to identify key strengths and weaknesses of the system and to make recommendations based on findings.

Methodology An evaluative descriptive study was conducted from April to July 2017. The Lab-based Influenza Surveillance System was conducted at the national level. Assessment of qualitative and quantitative system attributes was done utilizing the CDC's Updated Guidelines for Evaluating Public Health Surveillance Systems, 2001. Desk review of literature, departmental documents and reports were also conducted. The stakeholders were identified and interviewed using a semi-structured questionnaire.

Results The system was found to be simple and easy to operate but less flexible to integrate with other diseases. Data quality was good as 80% of observed forms were completely filled. Timeliness was good as the data takes 24-48 hours from sample collection to report submission to the central level. Acceptability is good as private and public-sector hospitals and labs are involved. Sensitivity calculated was 62% and Predictive Value Positive (PVP) was 37.2%. The representativeness of Lab based influenza surveillance system is poor as it is a sentinel surveillance with specific reporting sites strategically placed. Data from all sentinel sites is analyzed at national reference lab where it is summarized to use for planning and management purposes.

Conclusions The system is meeting its objectives. Sustainability and stability of the system needs to be improved by allocation of public funds. Extension of the coverage of the system will result in improved representativeness. Regular capacity building of the staff at reporting site will ensure continued quality of reporting.

Vaccine Preventable Diseases

Abstract Code: 2017-EOH-141

Presenter Name: Dr. Abbas Abbas

Country: Iraq

Presenter Email: drabbasmahdi@yahoo.com

Author: A. Sameer

Title Evaluation of Hepatitis B Surface Antigen Rapid Test in Detection of Hepatitis B Virus Infection in Salahaldin Governorate, Iraq, 2012

Background Hepatitis B virus (HBV) can be detected by different serological and molecular biological methods. Nationally, high laboratory cost and lack of availability of Enzyme linked immunosorbent assay (ELISA) technique in peripheral health facilities raise the need for evidence-based decision on the use of HBs Ag rapid test in detection of HBV infection.

Purpose to evaluate the validity of ACON-HBs Ag rapid diagnostic tests in detection of hepatitis B virus infection in sentinel sites at Salahaldin Province.

Methodology Cross sectional study conducted from 1st march to 1st September 2012 in three sentinel sites at Salahaldin governorate. Depending on Hepatitis B median endemic index of 10%, a total of 900 subjects of both genders attending these sentinel sites and screened by ELISA technique for HBs Ag presence, were re-screened by the ACON® HBs Ag rapid test device. The ACON HBsAg One Step Test is a qualitative, solid phase, two-site sandwich immunoassay for the detection of Hepatitis B surface Antigen (HBsAg) in serum or plasma. Results of both techniques were compared and validity of HBs Ag rapid test was calculated statistically by SPSS version 17.

Results ACON HBs Ag rapid test showed an overall sensitivity, specificity, positive predictive value, and negative predictive value of 92.8%, 99.6%, 97.9%, and 99.2% respectively. But nothing is perfect. These rapid tests fail to detect HBs Ag concentrations between the cutoff value to < 1 OD value.

Conclusions ACON® HBs Ag rapid tests proved to be valid in detection of HBV infection in peripheral health care facilities lacking ELISA machine. However, at these facilities we should depend on ELISA technique in detection of HBV infection in those subjects requiring invasive procedures.

Vaccine Preventable Diseases

Abstract Code: 2017-OTH-438
Presenter Name: Dr. Ali Amily
Country: Iraq
Presenter Email: amily_ali@yahoo.com
Author: M. Abbass, F. Lami

Title Impact of Training of Primary Healthcare Centers' Vaccinators on Immunization Session Practices-Wasit Province, Iraq, 2016-An Interventional Study

Background Immunization is one of the successful and cost-effective health interventions that averts >2.5 million child deaths annually. WHO and UNICEF estimates of immunization coverage in Iraq in 2015 revealed 58% for DTP3 and 57% for measles. High-quality immunization session practices (ISPs) can ensure safer, more effective vaccination and higher coverage rates.

Purpose The objective of this study was to assess the impact of training of primary healthcare centers (PHCs) vaccinators on quality of ISPs in PHCs in Wasit province.

Methodology An interventional study was conducted on ten (18%) PHCs in Wasit province where two PHCs were randomly selected from each health district. ISPs were first assessed by direct on-job observation of immunization sessions through a single visit for each PHC using modified WHO immunization session checklists and findings were grouped into seven domains: vaccine and diluent management, cold chain management, session's equipment, registration, communication, vaccine preparation and administration and waste management. Then, the vaccinators in these PHCs were enrolled in a one-day training using WHO module "Managing an Immunization Session" as a training material. A second assessment was made one month later using the same method. Mean differences in the domains' scores were calculated.

Results A significant improvement was clear in three domains: vaccine and diluent management (P=0.005), cold chain management (P=0.01) and vaccine preparation and administration (P=0.003). Eight PHCs (80%) showed improved, whereas the remaining two (trained vaccinators were moved away soon after training in one, while the other was conducting a badly managed campaign with influenza vaccine) showed some decline.

Conclusions Training of PHCs' vaccinators was effective in improving ISPs. We recommend using this training module for other PHCs to improve utilization of immunization services. The impact on vaccination coverage may be assessed one year later.

Vaccine Preventable Diseases

Abstract Code: 2017-OTH-437
Presenter Name: Dr. Ali Amily
Country: Iraq
Presenter Email: amily_ali@yahoo.com
Author: F. Lami

Title Assessment of Immunization Session Practices in Primary Healthcare Centers - Wasit Province, Iraq, 2016-17

Background Annually, vaccines prevent more than 2.5 million child deaths globally. WHO and UNICEF estimates of immunization coverage in Iraq in 2016 revealed 63% for DTP3 and 66% for MCV1. Wasit is among governorates with a large number of under-immunized children, opening the door for many future outbreaks. Immunization session practices (ISPs), when maintained of high-quality, can ensure safer and more effective vaccination as well as higher coverage rates.

Purpose The objective of this study was to assess ISPs in Primary Healthcare Centers (PHCs) in Wasit province.

Methodology We conducted this cross-sectional study on 24(44%) PHCs in Wasit province, selected by simple random sampling. Based on WHO and the national guidelines, checklists were developed to assess 58 ISPs that were grouped into seven domains: vaccine and diluent management, cold chain management, session's equipment, communication with clients and caregivers, vaccine preparation and administration, card review and registration and waste management. The score (out of 100%) was calculated for each domain in all selected PHCs, then the average for all domains was calculated in each PHC. The assessment was made by direct on-job observation of immunization sessions, through a single visit conducted to each PHC.

Results PHCs were ranging in their ISPs: 52-78%; with a mean of 67% (± 8%). The highest scores were for the following domains: session's equipment (88%), waste management (82%) and card review and registration (81%). The least achieving domains were: communication with clients and caregivers (36%) and cold chain management (38%). Vaccine preparation and administration was scoring 69%, whereas the score for vaccine and diluent management was only 50%.

Conclusions ISPs practiced in PHCs in Wasit province were far from the standard. National Expanded Program on Immunization should work on raising the capacity of vaccinators, particularly their communication skills with the clients and caregivers and the management of vaccines and cold chain.

Vaccine Preventable Diseases

Abstract Code: 2017-EOH-243

Presenter Name: Mrs. Nadine Haddad

Country: Lebanon

Presenter Email: esu.haddadn@gmail.com

Author: H. Abou Naja, S. Kassouf, A. Paez Jimenez, G. Abou Mrad, W. Ammar, N. Ghosn

Title Effectiveness of Previous Mumps Vaccination During the 2014-2015 Outbreak in Lebanon

Background In Lebanon, MMR was introduced at 12 months and 4-5 years in 1996. In 2014, the 2nd MMR dose was shifted to 18 months with 79% coverage; a six-fold increase in mumps national incidence was observed in December.

Purpose This study aims to determine mumps vaccine effectiveness (VE) among Lebanese population to guide immunization policies.

Methodology Clinical and confirmed mumps cases reported to Epidemiological Surveillance Program between 2014W46 and 2015W11 were eligible if Lebanese and aged 1.5 to 19 years-old. They were matched 1:1 on age and locality to randomly selected controls using phonebook of the same area. Information was collected by structured phone interviews. Mumps vaccination status was based on documented valid dates for MMR doses. Data were entered using Epidata 3 and analyzed using Stata13. VE [(1-OR) x100] of one and two doses and ORs (95%CI) for acquiring mumps were estimated using conditional logistic regression.

Results 91 cases and 91 controls were included. Only 36% of cases had vaccination cards, compared to 71% of controls (p<0.001), and 94% of cases were not vaccinated compared to 51% of controls (p<0.001). Vaccine effectiveness was estimated 60% (CI= -27%: 88%) for one dose and 88% (CI=60: 96%) for 2 doses.

Conclusions Two-doses of MMR vaccine were estimated as 88% effective against mumps, similar to results found in the literature. Suboptimal MMR2 coverage can explain this outbreak. Efforts should focus on achieving high MMR coverage and raising population's awareness about preserving documentation of vaccination.

Vaccine Preventable Diseases

Abstract Code: 2017-EOH-446

Presenter Name: Dr. Azddine Bouzid

Country: Morocco

Presenter Email: drbouzidaz@gmail.com

Author: M. Adnane, N. Derfoufi

Title Epidemiological Profile of Meningitis in Prefecture of Temara, Morocco, 2011-2016

Background Meningitis remains a serious public health problem worldwide. Morocco has implemented a national program against this disease since 1989, but it still causes high morbidity and mortality, with an average rate of lethality of 10%.

Purpose Our study aims to describe the epidemiology profile of meningitis in the prefecture of Temara from 2011 to 2016.

Methodology We conducted a descriptive study of all cases of meningitis reported to the Provincial Epidemiology Unit (CPE) of the province of Temara in Morocco between 2011 and 2016. We collected data from the database of the CPE at the prefectural delegation of health. Data analysis was done using the Epi-info software.

Results During our study period, we recorded 140 meningitis cases. Trend shows a maximum of cases in 2012 (34.0%), because we tested an application of instant declaration via internet which was abandoned. Cases were males in 59.0% and most were in urban areas (80.0%). The mean age was 40.5 years and the most affected age group was 5 to 15 years (28.5%). Most cases of meningitis (51.4%) were presumptively attributed to bacterial pathogens without final determination of causative organisms, and a third of cases were viral meningitis (30.0%). Only 9.0% of cases were culture-confirmed. The mains pathogens were Nesseria meningitidis (5 cases = 38.5%) followed by pneumococcus (4 cases, 30.7%) and Heamophilus influenza (1 case, 7.6%). Among patients with known outcome (n=36), case-fatality rate was 33.3%. Investigations around cases were carried out.

Conclusions Meningitis remains an important cause of mortality in Morocco. Surveillance needs to be improved, particularly by computerizing case notifications. Laboratory conditions and improved methods of bacterial detection including isolate serotyping should be available to increase the confirmation rate. We also need to strengthen the involvement of the private sector which is very weak in notification.

Vaccine Preventable Diseases

Abstract Code: 2017-EOH-112
Presenter Name: Dr. Fatima Zahra Benfouila
Country: Morocco
Presenter Email: fatima.zahra.benfouila@gmail.com
Author: M. Merabet, A. Rguig, K. Khatri, A. Khattabi, F. Meski

Title Measles Vaccine Effectiveness Among Children – Morocco - 2017

Background Measles are one of the leading causes of vaccine-preventable death among young children in the worldwide. In Morocco, vaccination against measles has been introduced into the National Immunization Program (NIP) since 1987, as a single dose at nine months old. A second dose has been introduced since 2003 as part of the elimination strategy.

Purpose The purpose of our work was to evaluate the vaccine effectiveness of measles vaccination after the first and the second dose among children aged between 12 and 60 months, from 2010 to 2016 in Morocco.

Methodology We conducted a test negative design using data from the measles surveillance system. Only children aged 12-60 months with laboratory result recorded was included. The vaccine status (unvaccinated, vaccinated one dose, vaccinated two doses) was defined among cases: children who had confirmed infection (presence of IgM specific antibodies for measles) and controls: children who had negative lab result (absence of IgM specific antibodies for measles). Vaccine effectiveness (VE) was estimated using the formula VE = [1-odds ratio (vaccinated/unvaccinated)] × 100.

Results In total 897 children were included from January 2010 to December 2016. The mean age was 36 months. The male female sex ratio was 0.8:1. According to the vaccination status, 785 were vaccinated, 79% of them have received one dose and 21% have received two doses. Lab result was positive for 186 (21%) of 897 patients. VE was 87% (CI 95%: 82%-93%) after one dose and 97% (CI 95%: 93%-99%) after two doses.

Conclusions The field assessment of vaccination effectiveness confirms that measles vaccine is an effective way to prevent measles especially with two doses. The NIP should be reinforced by more vaccination campaign to cover all children who have not received the second dose.

Vaccine Preventable Diseases

Abstract Code: 2017-EOH-397
Presenter Name: Dr. Nadia Noreen
Country: Pakistan
Presenter Email: nadia.jamil3@gmail.com

Title Outbreak Investigation of Measles in Farash Town, Islamabad, April 2017

Background On April 14, 2017, two measles cases were reported by local health practitioner from Farash Town. A team of FELTP fellows were deputed to investigate the outbreak.

Purpose On the request of district health authorities an outbreak investigation was conducted to assess magnitude, identify risk factors and recommend control measures.

Methodology Outbreak investigation was carried out from April 18 to May 05, 2017. Active case finding was conducted through a house-to-house survey. A case was defined as "onset of maculopapular rash with fever and presence of any of the sign/symptoms like coryza, conjunctivitis and cough in a resident of Farash Town from March 25, 2017 to April 30, 2017". Community-based age and sex-matched controls were selected. Vaccine coverage survey was conducted in a cluster of 245 houses. Frequencies were calculated, attack rates computed, and vaccine efficacy was determined. Blood samples of 03 suspected cases were sent to Public Health Laboratories at NIH Islamabad for confirmation.

Results A total of 15 cases were identified; 13 through active case finding. Mean age was 44.5 months (range 05-120 months). The cases were predominantly male n=9(60%). Overall attack rate was 1.15% and most severely affected age group was 24-36 months (n=4, AR=10.81%) followed by 12-24 months (n=3, AR=8.10%). Diarrhea developed in n=12 (80%) and pneumonia developed in n=2 (13.3%). Immunization coverage survey showed that AR in unvaccinated was 25% and AR in vaccinated was 10%, hence the vaccine efficacy was calculated to be 60%. The most significant reason for non-vaccination was misconception about vaccination (OR: 24.0, CI: 4.9-116.1). All blood samples were positive for measles-specific IgM on ELISA.

Conclusions Low immunization status was the most probable cause of outbreak. The results were communicated to district health authorities for mass vaccination. Health awareness session was conducted for all households. No new case was reported during the two weeks follow-up.

Vaccine Preventable Diseases

Abstract Code: 2017-EOH-14

Presenter Name: Dr. Palwasha Anwari

Country: Afghanistan

Presenter Email: anwari222@gmail.com

Author: F. Debellut, C. Pecenka, S. Mohammed, A. Clark, D. Groman, N. Safi

Title Potential Impact and Cost-Effectiveness of Rotavirus Vaccination in Afghanistan

Background Despite progress made in child survival in the past 20 years, 5.9 million children under five years died in 2015, with 9% of these deaths due to diarrhea. Rotavirus is responsible for more than a third of diarrhea deaths. In 2013, rotavirus was estimated to cause 215,000 deaths among children under five years, including 89,000 in Asia. As of April 2017, 92 countries worldwide have introduced rotavirus vaccination in their national immunization program. Afghanistan has applied for Gavi support to introduce rotavirus vaccination nationally.

Purpose This study estimates the potential impact and cost-effectiveness of a national rotavirus immunization program in Afghanistan.

Methodology This study examined the use of Rotarix® (RV1) administered using a two-dose schedule at 6 and 10 weeks of age. We used the ProVac Initiative's UNIVAC model (version 1.2.09) to evaluate the impact and cost-effectiveness of a rotavirus vaccine program compared with no vaccine over ten birth cohorts from 2017 to 2026 with a 3% annual discount rate. All monetary units are adjusted to 2017 US$.

Results Rotavirus vaccination in Afghanistan has the potential to avert more than one million cases; 660,000 outpatient visits; approximately 50,000 hospital admissions; 650,000 DALYs; and 12,000 deaths, over 10 years. Not accounting for any Gavi subsidy, rotavirus vaccination can avert DALYs at US$82/DALY from the government perspective and US$80/DALY from the societal perspective. With Gavi support, DALYs can be averted at US$29/DALY and US$31/DALY from the societal and government perspective, respectively. The average yearly cost of a rotavirus vaccination program would represent 2.8% of the total immunization budget expected in 2017 and 0.1% of total health expenditure.

Conclusions The introduction of rotavirus vaccination would be highly cost-effective in Afghanistan, and even more so with a Gavi subsidy.

Vector Borne Diseases

Abstract Code: 2017-OTH-398

Presenter Name: Dr. Abdul Sami Khan

Country: Pakistan

Presenter Email: drask99@hotmail.com

Author: A. Saeed

Title Suspected Chikungunya Outbreak Investigation, District Gwadar Baluchistan Pakistan March 2017

Background District Health Officer Gwadar reported chikungunya cases to Provincial Disease Surveillance and Response Unit Quetta and requested for an epidemiological field investigation.

Purpose A team sent to confirm, investigate and suggest control measures on 17th March 2017.

Methodology A case was defined as a resident of District Gwadar with history of fever with joints pain within last 03 months (January – March 2017). Active case finding was done from 18th to 24th March 2017. Public and private health facilities were visited to review their records. water storage and sanitation practices for vector identification were assessed.

Results 684 patients were identified (over all attack rate = 0.25%). 29 blood samples collected and sent to NIH Islamabad, 72% (n=21) were Chikungunya positive. Among patients 50% (n=344) were male and 50% (n=340) females. Mean age was 27 years (range=1-70 years). Most affected age group was 15 to 19 years (14.6%, n=100), 20 to 24 years (13%,n=92), 25 to 29 years (11%, n=76) and 35 to 39 years (9% n= 59) with attack rates 0.35%, 0.38%, 0.38%,and 0.45% respectively.56%(n=382) patients were from Gwadar, 23%(n=161) from Jewani and 21% (n=141) from Pasni with attack rates 0.37%, 0.41% and 0.14% respectively. Investigation revealed that first case was reported on 4th Jan-2017. Being an emerging disease there is lack of knowledge about this disease, its prevention and control among health care providers and community. Sanitation/water storage practices were very poor, larvae of vector also isolated during house hold survey.

Conclusions Lack of proper sanitation and storage techniques in combination of warmer temperature of this coastal district are most probable causes of outbreak. Awareness sessions among community, district administration and healthcare providers regarding chikungunya, its prevention and control were conducted along with vector control on immediate basis through fogging of reservoirs and internal residual spray (IRS). Surveillance system established for regular reporting.

Vector Borne Diseases

Abstract Code: 2017-EOH-492

Presenter Name: Dr. Bisma Memon
Country: Pakistan
Presenter Email: drbisma@gmail.com
Author: S. Hussain, A. Khaskheli, N. Masood

Title An Outbreak Investigation of Chikungunya Fever -District Tharparkar, Pakistan, August 2017

Background On 8th August 2017 print media reported 49 cases of fever with severe joint pains from different villages of District Tharparkar. On the same day a team of FELTP fellows was deputed to investigate the situation.

Purpose The objectives of the investigation were to determine the extent of outbreak, evaluate the risk factors and suggest recommendation for control.

Methodology A descriptive followed by age and sex-matched case-control study was conducted in District Tharparkar in August 2017. Review of reported cases and active case finding was conducted. A case was defined as acute onset of fever (102°F) and severe arthralgia or arthritis not explained by other medical conditions, in a resident of District Tharparkar from 13 July to 27 August 2017." A structured questionnaire was used to collect the information. Blood samples were sent to National Institute of Health for confirmation RT-PCR. An entomological survey was conducted in the affected area. Frequencies were calculated, attack rates computed, and multivariate analysis undertaken at 95% confidence interval and a 5% margin of error.

Results A total of 204 cases were identified, 155 through active case search. Mean age was 25.5 years (range 01 month – 80 years) with a female preponderance (n=112; attack rate (AR) 54%). The overall AR was 26.7/1000, with 20-29 years being the most severely affected age group (AR 33/1000). Presence of uncovered water containers was significantly associated with disease (OR=10.4, 95% CI= 6.54-16.6). Use of bed nets had a protective effect. Out of 48 samples sent, 34 were positive on RT-PCR. Entomological Survey revealed house index 79.3%, container index 40.6% and breteau index 182%.

Conclusions Presence of uncovered water containers in the house were potential breeding sites for the vector and was the most probable cause of the outbreak. Indoor residual spray followed by fogging and community awareness were conducted.

Vector Borne Diseases

Abstract Code: 2017-EOH-391

Presenter Name: Dr. Asim Minallah
Country: Pakistan
Presenter Email: asim3307@hotmail.com
Author: A. Baig, N. Azam

Title Investigation of Dengue Fever Outbreak- District Peshawar, Pakistan, September 2017

Background About 50 to 100 million people get infected with Dengue every year. Since 2003, Pakistan has seen multiple outbreaks with biggest one in 2011 in Lahore involving 22,000 cases. From August 6 to September 16, 2017, 112 Dengue fever cases were reported to a hospital in town X of District Peshawar.

Purpose A case-control study was conducted to assess the magnitude of the disease, evaluate risk factors and recommend control measures.

Methodology A desk review of available records and active case finding was conducted. A case was defined as "fever of >38 0C for 2 to 10 days with minimum two of the following; headache, rash, retro-orbital pain, myalgia and bleeding in a resident of town X from July 24 to September 21, 2017 and a positive NS-1 test". Age and sex-matched controls were identified from the same locality. A structured questionnaire was used to collect information about cases and controls. Frequencies were calculated, attack rates computed, and odd ratios determined at 95% confidence interval with p value <0.05.

Results A total of 140 cases were identified (28 cases through active case finding) with mean age of 28.9 years (range 8-55 years). Male to female ratio was 8:1 with an overall attack rate of 9.3%. The most affected age group was 21-30 years (AR=15.1%). Out of 140 cases, 88 had open water containers in the house (OR=3.9, 95%CI=2.5-6.0) and 84 had larvae present in their households (OR=2.5, 95%CI=1.6-3.8). Regular use of repellents and screened doors and windows showed to have a protective effect.

Conclusions Presence of open water containers inside the house served as breeding grounds for the vector and were the most probable cause of the outbreak. Regular use of repellents was shown to be protective. Local breeding sites were destroyed, and residents were educated about the risk factors and protective measures.

Vector Borne Diseases

Abstract Code: 2017-EOH-61

Presenter Name: Dr. Fawad Khurshid

Country: Pakistan

Presenter Email: fawad_khurshid@yahoo.com

Title Epidemiological Determinants Associated with the Spread of Dengue Fever in Lahore, Punjab -2013

Background Dengue is the most rapidly spreading mosquito-borne viral disease in the world. It is one of the important public health emergencies of international concern as per International Health Regulations (IHR). In Punjab the disease suddenly saw an upsurge towards August 2011 especially in Lahore and adjoining areas of Punjab. By 2011, total of 20864 cases of Dengue had been reported in the province, including 17256 in Lahore alone. A large number of these cases i.e. 21292 in Punjab including 17232 in Lahore only were cured while a total of 352 deaths including 279 in Lahore were reported. Apart from Lahore, maximum number of cases have been reported from Faisalabad (783), followed by Rawalpindi (410), Pakpattan (233) and Sheikhupura (225).

Purpose To identify epidemiological determinants responsible for causation of Dengue for preventing future outbreaks in the study area particularly and in Punjab in general.

Methodology A case control study was conducted in December 2013 to identify the epidemiological determinants for spread of Dengue Fever. Cases were those confirmed with IgM/IgG positive (n=147) living in Data Ganjbaksh town Lahore and controls (n=300) were selected from the same area who were suspected cases with laboratory negative results. A standardized questionnaire was developed to collect data. A line list of cases was developed, and data was analyzed using Epi Info® version 7.0

Results Variables found significant in the bivariate analysis were included in a logistic regression analysis. The presence of indoor stagnant water (OR = 3.7), indoor larvae (OR = 3.1), not using repellent (OR = 2.7), and older age (OR = 1.2) were independent determinants of dengue infection ($P < 0.01$ for all).

Conclusions Health education campaigns for improved water storage practices. Indoor residual sprays in urban and peri-urban high-risk areas 1 month before the transmission period. Community based environmental management was recommended.

Vector Borne Diseases

Abstract Code: 2017-EOH-441

Presenter Name: Dr. Jamshed Khan

Country: Pakistan

Presenter Email: dr.jamshedkhanzada@hotmail.com

Title Outbreak Investigation of Dengue Fever in District Malir, Karachi, Sindh, Pakistan, 2015

Background Dengue fever is an acute arboviral disease transmitted to humans by bite of mosquito genus Aedes. On September 13, 2015, private hospital reported cluster of 19 suspected dengue cases from district Malir. In response to this, we initiated an epidemiological and entomological investigation.

Purpose To know magnitude, risk factors, Contain and suggest recommendations.

Methodology Descriptive followed by case-control study was carried-out Age- and sex-matched controls were taken with a ratio of 1:2. World Health Organization standard case definition was used to identify suspected and confirmed cases. Active case finding done in health facilities and in community from September 14 to November 10, 2015.Data were analyzed using EpiInfo® version 7.0. Bivariate analysis done on 95% Confidence interval with 5% margin of error. Blood was collected for confirmation. Entomological surveillance was carried out.

Results One hundred five cases identified, and two deaths reported. CFR=1.9%). Eighty-three (79%) were males with attack rate 1.5/1000.Mean age for cases was 26 years (range: 7-84 years). Most affected age group was 21-30 years n=42; 40% with attack rate (2.4/1000). Over all attack rate was 0.9/1000. Uncovered fresh water reservoir around and the house (OR 6.9; 95% CI: 2.9-11.32; $p<0.05$) non-usage of repellents (OR 3.0; 95%CI: 1.36-3.7; $p <0.05$), absence of window screens (OR 9.5; 95%CI 6.4-24.3; $p <0.05$) were statistically significant associations. All cases were confirmed on ELISA and NS1 Antigen. Adult misquotes inside the homes, while Larva and pupa detected in storage fresh water.

Conclusions Stagnant of fresh water in and around domestic premises and not using of repellents in homes and larvicides were most probable causes of this outbreak. Failure to implement mosquito preventive control measures may have contributed to this outbreak. Establishment of isolation wards, Mass awareness, distribution of bed nets, spraying mosquito insecticide and fogging contributed to control outbreak.

Vector Borne Diseases

Abstract Code: 2017-EOH-362
Presenter Name: Dr. Sandeep Mehraj
Country: Pakistan
Presenter Email: drsandeepguriro@gmail.com
Author: N. Masood

Title Outbreak Investigation of Dengue Fever in Water Scarce District Tharparkar of Pakistan, 2016.

Background On 8th December 2016, 43 cases of dengue fever were reported from Tharparkar to Director General Health Office. The very next day FELTP fellows were assigned to investigate the outbreak.

Purpose Objectives were to assess the magnitude, evaluate the risk factors and recommend control measures.

Methodology Review of hospital records and active case finding was done. A descriptive followed by a case control study was conducted in December 2016. A case was defined as acute fever more than 102°F lasting >3 days, plus a positive NS-1 test in a resident of Tharparkar during September to December 2016. During entomological survey objects containing water were sampled and investigated for presence of larvae or pupa. The collected vectors were examined for species identification.

Results A total of 254 cases were identified (211 by active case finding) with 73% males. Overall attack rate (AR) was 0.02 with 10-14 years being the most affected age group (AR=0.03). Out of 254 cases, 79%(n=201) had indoor water receptacles (OR 32, CI 19.6-54 with p < 0.00), 61% (n=155) had potted plants inside the house (OR 8, CI 5-13, p value < 0.00), and 46% (n=118) had outdoor water receptacles (OR 3, CI 2-4 with p value < 0.00) whereas intact window nets 52% of cases (n=132) (OR 0.44, CI 0.02-0.08, P value < 0.00) were found protective against getting the dengue infection. Total 2616 Aedes larvae-(58.3 per dip) and 423 pupae-(8 per dip) were collected by 320 dips. Among 152 houses 182 breeding sites were identified. Adult Aedes were found in 12 of 230 rooms.

Conclusions The outbreak was likely caused by presence of vector breeding sites inside and outside the house. On the recommendation of the study, health authorities initiated health awareness sessions and promoted mechanical control of breeding sites as well as use of windows net.

Vector Borne Diseases

Abstract Code: 2017-EOH-35
Presenter Name: Dr. Inayatur Rehman
Country: Pakistan
Presenter Email: drinayat.na43@gmail.com
Author: M. Saleem

Title Outbreak Investigation of Cutaneous Leishmaniasis (CL) in North Waziristan Agency, Federally Administered Tribal Area (FATA) – April 2016

Background On 4th April 2016, community representative from village Spinwam, North Waziristan reported 39 suspected cases of Cutaneous Leishmaniasis. FELTP Fellow and N-STOP Officer North Waziristan attached to Political Agent Office North Waziristan was tasked to visit the affected area and carry out necessary investigations.

Purpose The objectives were to identify any additional cases, associated risk factors and recommendation for control.

Methodology A case was defined as a resident of Tehsil Spinwam with one or more papular, nodular or ulcerative lesions on the skin after history of sand fly bite from February 2016 to April 2016.Hospital records were reviewed, patients and health staff were interviewed, and suspected sites visited. A case control study was conducted with one control for each case.

Results Sixty-seven cases were identified (58 clinically and 9 by microscope). Male to female ratio was 1:2. Median age: 10 years (1 – 60 years). The Epi curves shows intermittent source of infection. Risk factor analysis showed that private gardens (O.R=1.95, 95%CI=1.11-3.26;p-value<0.05), stagnant water-body (O.R=1.84, 95%CI=1.08-3.13;p-value<0.05) and not using mosquito-protective measures (OR=36.11, 95%CI=16.8-77.5; p-value<0.05) were significantly associated with the illness. Use of bed nets (O.R=0.05, 95%CI=0.02-0.12; p-value<0.05) and mosquitoes repellents (O.R=0.05, 95%CI=0.02-.012; p-value<0.05) were negatively associated with the illness.

Conclusions The area is bordering with Afghanistan and index case had a travel history from Afghanistan four months before appearance of his lesion. He left it untreated allowing the parasite to be transmitted to the indigenous sandflies and subsequently resulted in an outbreak. Based on results existing surveillance system for Leishmaniasis was strengthened, indoor residual spray and fumigation for vector control carried out, bed nets and insect repellants provision were ensured. Injection Glucontine was arranged. Regular awareness sessions were recommended. During the 04-month follow up period 32 cases were reported who had sand fly-bite histories of longer than 05 months.

Vector Borne Diseases

Abstract Code: 2017-EOH-411
Presenter Name: Dr. Waheed Ahmed
Country: Pakistan
Presenter Email: drlashari@yahoo.com

Title Outbreak Investigation of Malaria in Usta Mohammad, District Jaffarabad

Background On 7 August 2017, District Health office received information about 2 deaths due to high grade fever at Usta Mohammad. 7 similar cases were also reported from same village.

Purpose Descriptive followed by Case Control study was conducted to investigate outbreak and to assess risk factors

Methodology Descriptive followed by Case Control study was conducted to investigate outbreak and to assess risk factors. Case was defined "any person resident of Usta Mohammad, regardless of age and sex, having fever with chills with August 7th August to 26th September 2017. Review of hospital records and active case finding was done through house to house survey. Matched for Age and sex controls were taken from locality with a ratio of 1:2 and interviewed. Blood samples were taken for microscopy and rapid diagnostic tests. Data were analyzed using EpiInfo® version 7.0.

Results 180 cases identified with mean age of 22.5 years (range: 01-60). Males were 59%. Age group 1-20 years was most affected (n = 59). All cases were confirmed on microscopy and 90% (n = 162) were positive on rapid test. Plasmodium falciparum was positive in 35% (n = 64) while rest were positive for Plasmodium vivax. Attack rate was 31.4%. Case fatality was 12%. Spot map shows clustering around a stagnant Pond water. Epi curve shows sudden outburst of cases on 23th of September 2017. statistical associations was found between disease occurrence and following risk factors; Stagnant water pond (OR 48.23; 95%CI: 4.43-31.27; p <0.05), non-usage of repellents (OR 5.3; 95%CI:2.67-13.52; p <0.05), absence of window screens (OR 11.2; 95%CI 5.44-41.12; p <0.05), absence of insecticide spray (OR 4.22; 95% CI:6.76-183.72; p<0.05) and waste dump near houses (OR 7.2; 95%CI 4.91-33.41; p<0.05).

Conclusions Stagnant Pond water was major mosquito breeding site and probable cause of outbreak. Mass awareness, Residual sprays, pond treatment by larvicidal, provision of bed nets and prophylaxis with chloroquine were able to control the outbreak.

Vector Borne Diseases

Abstract Code: 2017-EOH-324
Presenter Name: Dr. Manar Keshk
Country: Egypt
Presenter Email: manar.keshk@gmail.com
Author: S. Elshourbagy, M. Genedy, A. Kandeel

Title Investigation of Malaria Outbreak in Aswan, Egypt, 2014

Background On May 26th 2014, new malaria cases were reported from Aswan in upper Egypt to the ministry of health and population (MOHP) through the national surveillance system(NEDSS), with no history of travel to a malaria-endemic country. MOHP interfered with a rapid response team(RRT) to investigate and contain any suspected outbreak. Efforts were concentrated on active-case finding, laboratory testing, entomological surveillance, proper treatment of patients and health education

Purpose To investigate and contain any suspected outbreak.

Methodology Field investigation in the affected village and its surrounding 15 villages. started on May 28th,2014 after confirmation of the first case. The RRT used a standardized case definition and distributed it to all hospitals and health unites in Aswan. Cases were diagnosed clinically, and laboratory confirmed. Active case finding was done. Investigation continued till June 19th till no additional cases were reported.

Results A total of 319 suspected cases at Edfu Fever Hospital; about 14,696 samples examined during active case-finding; where 4 samples proved positive and were transferred to Edfu Fever Hospital to receive proper treatment. A total of 22 cases were confirmed for malaria, all were caused by P. vivax. The median age of patients was 19 (range 6-90 years), with male: female ratio 1:1. No complications or deaths were reported. All cases were discharged after full recovery and after performing 4 blood films all of which are negative for plasmodia. The first onset of symptoms occurred on May 20th 2014 and the final case revealed symptoms on the 15th of June 2014.

Conclusions The Egyptian MoHP succeeded in early detection and rapid containment of a new focus of re-emerged malaria cases at a village in Upper Egypt through intensive malaria control procedures. Malaria Surveillance should be continued and reinforced for early detection of future outbreaks

Vector Borne Diseases

Abstract Code: 2017-OTH-86
Presenter Name: Dr. Labiba Anam
Country: Yemen
Presenter Email: labibaanam25@gmail.com

Title Descriptive Analysis of Malaria Surveillance System Data, Yemen, 2011-2015

Background Malaria remains one of the most serious health problems in Yemen where 68% of population is living in malaria risk areas. An Integrated Malaria Surveillance System (IMSS) was introduced in 2009 to improve reporting.

Purpose To describe the epidemiology of malaria and identify groups at risk.

Methodology Data for 2011-2015 was obtained from the National Malaria Control Program (NMCP). According to the NMCP Guidelines, confirmed malaria case is defined as a case that is positive by microscopy or rapid test. We calculated incidence rate (IR) by age group, sex, type of plasmodium, seasonality and population at risk using projections from the 2014 Central Statistical Organization data.

Results Although the overall malaria IR dropped from 11/1000 in 2011 to 5 in 2015, the IR among < 5 children increased from 8 to 15/1000 and the percentage of confirmed cases increased from 0.64% to 0.83%. Among pregnant women, the IR increased from 4/1000 in 2011 to 6 in 2014 but decreased to 2 in 2015. Two thirds of malaria cases were reported among males and from the coastal governorates. Plasmodium Falciparum accounted for 99% of cases.

Conclusions Despite IR dropped from 2011 to 2015, such drop might not reflect improvement in control and prevention measures, but could reflects underreporting due to political instability, war situation and poor access to health facilities. Proper targeting especially of coastal areas by insecticide treated bed nets and indoor residual spraying is necessary. Strengthening of surveillance system for high-risk groups i.e. <5 children and pregnant women is recommended. A qualitative research should investigate reasons behind the predominance of malaria among males. Further IMSS evaluation is recommended.

Zoonotic Diseases

Abstract Code: 2017-EOH-451
Presenter Name: Dr. Farida Khudaidad
Country: Pakistan
Presenter Email: drfaridakhan9@gmail.com
Author: A. Saeed

Title Descriptive Analysis of Suspected Crimean-Congo Haemorrhagic Fever (CCHF) Cases in Isolation Ward of Public Sector Hospital, Quetta from March-August 2017

Background CCHF cases from Balochistan and Afghanistan are referred to isolation ward in Quetta. CCHF is endemic to Balochistan but still there is no established surveillance system in province and no tick bite reporting system. The main objective was to determine the means of transmission and the epidemiologic characteristics of disease.

Purpose Describe the Epidemiology of CCHF and analyze the situation of health facility.

Methodology A descriptive study was carried out in the CCHF isolation ward in Quetta from March-August 2017. Using standardized case definition, all patients admitted in Isolation ward with clinical evidence of CCHF were included in the study. After taking informed consent, data was collected on demographic factors, history of animal contact, tick bite history, co morbidity, laboratory results and treatment outcome. Means and percentages were calculated.

Results During the study period, 51 suspected CCHF patients were admitted in Isolation ward, 38 (74.5%) males were affected. Mean age of the cases was 30 years (range 02-75years). Most affected 16 (31%) age group was 21-30 years.48 (94%) cases had history of animal contact and 44 (86%) with tick bite. Majority of cases 42 (82%) were reported from May -August. 30 patients in study were tested by PCR, 16 (53.3%) were positive, out of which 5 (31%) expired. It is only isolation ward in whole province with 02 doctors, 02 nurses & 01 paramedics. Proper Personal protective equipment was not available. No Laboratory was available for immediate investigations.

Conclusions Given the overall results important risk factors for CCHF are history of tick bite, high-risk occupations and having contact with livestock. Public health measures should focus on preventing tick bites, increasing awareness of CCHF signs and symptoms, adopting hospital infection control practices, timely investigation & treatment to reduce mortality. Government should set up isolation units in all Major hospitals with proper surveillance system in Province.

Zoonotic Diseases

Abstract Code: 2017-OTH-463
Presenter Name: Dr. Muhammad Farooq Tahir
Country: Pakistan
Presenter Email: drmftahir@gmail.com
Author: F. Afzal, M. Athar

Title Prevalence and Antimicrobial Susceptibility Patterns of Salmonella Enteritidis and Salmonella Typhimurium Isolates from Commercial Poultry in Punjab, Pakistan

Background Non-typhoid salmonella infections are one of the leading food borne infections worldwide. Similarly, ever increasing antimicrobial resistance has become a major problem to animal as well as human health worldwide. Poultry being the single largest animal protein source in Pakistan is one of the major suspects for both these public health concerns.

Purpose This study was aimed to determine the prevalence of Salmonella Enteritidis and Salmonella Typhimurium in commercial poultry flocks of Punjab and to evaluate their antimicrobial susceptibility patterns.

Methodology Specimens were collected from morbid or dead birds suspected for salmonella infection on the basis of clinical signs or post-mortem lesions brought to five poultry diagnostic laboratories in Punjab between 2014 and 2017. The samples were then processed for bacterial isolation and molecular confirmation through PCR. The isolates were then subjected to antibiotic sensitivity test using disc diffusion method. The susceptibility was determined using CLSI guidelines for antimicrobial susceptibility testing. The frequencies and percentages were calculated using Epi info.

Results A total of 28150 samples were processed, among them 1.04% (294/28150) were positive. 89.5% (263/294) of the isolates were Salmonella Enteritidis while 10.5% (31/294) were Salmonella Typhimurium. The isolates were most sensitive to Ciprofloxacin and least to doxycycline with 96% (273/294) and 56% (166/294) response rates respectively. 7.8% (23/294) of the isolates were found to be resistant to three or more antibiotics. For other drugs the sensitivity percentages were Gentamicin 86% (254/294), Enrofloxacin 82% (241/294), Amoxicillin 77% (227/294), Norfloxacin 74% (219/294), Colistin 71% (208/294) and Neomycin 67% (197/294).

Conclusions Salmonella isolates were found more sensitive to Ciprofloxacin, followed by Gentamicin, Enrofloxacin and Amoxicillin while the isolates were least sensitive to Doxycycline.

Zoonotic Diseases

Abstract Code: 2017-OTH-464
Presenter Name: Dr. Muhammad Farooq Tahir
Country: Pakistan
Presenter Email: drmftahir@gmail.com

Title Surveillance and Molecular Epidemiology of Avian Influenza H9N2 Viruses Circulating in Pakistan

Background Avian influenza H9N2 is highly endemic in commercial and backyard poultry in Pakistan. Its widespread circulation and high mutation rates provide a possibility of novel reassorted viruses hence posing a serious public health threat.

Purpose This study was aimed to isolate and evaluate the AI H9N2 viruses circulating in poultry as well as aquatic birds in Pakistan between 2014 and 2017.

Methodology Specimens were collected from morbid or dead birds suspected for AI H9N2 on the basis of clinical signs or post-mortem lesions brought to five poultry diagnostic laboratories in Punjab. The samples were subjected for virus isolation. The isolates then were confirmed for H and N type using PCR. Six isolates were subjected to phylogenetic analysis of Haemagglutinin gene. The results were compared with isolate reported previously from Pakistan and other regional countries for homology.

Results 129,622 samples from 7481 poultry flocks were processed, 5.3% (399/7481) were positive for AIV H9N2. Sequence analysis showed that it had homology of 84-93% with different regional strains. Changes were seen at 24 different sites and at cleavage site at K148R and I151R in comparison to previous Pakistani isolates. Six possible glycosylation sites were observed. Neighbor joining phylogenetic tree confirmed its 93.4% homology with the isolate of Iran. The isolates were the same clade as other regional isolates and have common ancestors.

Conclusions The prevailing H9N2 viruses in Pakistan have certain markers and elements in the HA gene that may improve its avian to human transmission. Continuous surveillance of influenza A viruses is necessary to monitor their antigenic determinants. Protocols for the AI surveillance have officially been notified by Department of Livestock & Dairy Development Department, Punjab as a result of these findings.

Zoonotic Diseases

Abstract Code: 2017-HIV/STI-156

Presenter Name: Dr. Om Parkash Suthar
Country: Pakistan
Presenter Email: om_rajani2005@yahoo.com
Author: M. Athar, M. Aamir, T. Ghafoor

Title Prevalence of Brucellosis in Sindh Pakistan

Background Brucellosis is a bacterial disease caused by genus brucella. Bovine brucellosis affects cattle, sheep & goats, camels, equines, dogs and may also infects other animals. The disease in animals is characterized by abortion in last trimester and reproductive failure. All ages of cattle are susceptible, and infection can last for many years. Infected males develop epididymitis, orchitis and testicular abscesses. In humans, brucellosis can be caused by B. abortus, B. melitensis, B.suis (biovars 1-4) and rarely B. canis. The major losses caused by brucellosis are; decreased milk production, weight loss, calf mortality, infertility and lameness.

Purpose To identify the animals infected with brucellosis in selected districts and to develop disease control and mitigation measures of brucellosis.

Methodology The present study was aimed to determine the prevalence of brucellosis in cattle with reproductive disorders, randomly selected cattle, cross breed cattle and small ruminants (sheep and goat) in eight districts of Sindh by Milk Ring Test (MRT), Rose Bengal Plate Test (RBPT) and Indirect ELISA.

Results A total of 4559 animals with reproductive disorders (4225 female and 334 male) were screened. The prevalence was found 25.16% in female cattle while 27.84% in male cattle. A total of 6390 randomly selected animals (6248 female and 142 male) were screened. The prevalence was found to be 10.57% in female cattle while 21.12% in male cattle. The 1002 samples were screened from cross breed cattle, the prevalence was 25.54%. A total of 1639 samples were screened from sheep and goat. The prevalence was found to be 14.46%.

Conclusions It was concluded from the present study that brucellosis is endemic in Sindh, therefore the prevention of brucellosis with vaccination is best policy for the control of the disease.

Zoonotic Diseases

Abstract Code: 2017-EOH-455

Presenter Name: Dr. Saima Dil
Country: Pakistan
Presenter Email: saimatarrar@gmail.com
Author: Z. Hayat, A. Abbass

Title Outbreak of Brucellosis Among Workers of Cattle Dairy Farm at Renala Khurd- District Okara, Pakistan, January 2017

Background Brucellosis is endemic in Pakistan and poses a great challenge owing to nonspecific clinical manifestations. On 7th January 2017 three workers of cattle dairy farm at Renala Khurd reported to have intermittent fever.

Purpose A team was sent on 8th January 2017 to estimate magnitude of outbreak, evaluate risk factors and recommend control measures.

Methodology A case was defined as "prolonged intermittent fever, profuse night sweats and headache in a worker of cattle dairy farm at Renala Khurd from 7th to 21st January 2017." Cases and controls were matched by age and locality (1:4). Epidemiological information was recorded on a questionnaire. Serological testing was conducted using Rose Bengal plate test & iELISA. Frequencies were calculated, odd ratios determined at 95% confidence interval with p value less than 0.05.

Results A total of 9 cases were identified and mean age was 30 year (range 24-42 years). Overall attack rate was 29% and attack rate in cattle attendant was 47%. Among cases 78% (7/9) were involved in milking, feeding, cleaning, 44% (4/9) were habitual consumers of raw milk while 22% (2/9) were drivers and watchmen. Persons consuming contaminated raw milk (OR: 10; 95%CI: 1.4-70.2; p=0.024) and workers having direct contact with animals (OR: 8.3; 95%CL: 1.4-49; p=0.01) were more likely to have brucellosis. Apart from intermittent fever, night sweats (88%), headache (88%), fatigue (44%) and backache (11%) were the most frequent symptoms. All 9 cases were positive for brucella antibodies.

Conclusions The most probable cause of this outbreak was consumption of unprocessed contaminated milk and contact with secretions/excretions of infected animals. Monthly screening of workers, for a period of six months was recommended. Human cases were referred for medical treatment and Department of Health was notified.

Zoonotic Diseases

Abstract Code: 2017-EOH-454
Presenter Name: Dr. Saima Dil
Country: Pakistan
Presenter Email: saimatarrar@gmail.com
Author: A. Abbass

Title An Outbreak of Brucellosis in Cattle Dairy farm, District Okara, Pakistan – January 2017

Background Brucellosis is prevalent in livestock causing huge economic losses due to loss of production. On January 4, 2017, six abortions at third trimester of gestation were reported from a cattle dairy farm at Renalakhurd, District Okara.

Purpose An Outbreak investigation was initiated to assess the magnitude, identify risk factors and recommend control measures.

Methodology A case-control study was conducted. A case was defined as "adult cow in the affected dairy farm aborting at third trimester, without fever, from 18th December 2016 to 7th January 2017". Age-matched controls were selected from the same farm (1:4). Semen doses used for insemination, were tested by molecular and culturing technique. Serological testing was done through RBPT and i-ELISA. Frequencies were calculated, odd ratios determined calculated at 95% confidence interval with p value less than 0.05.

Results A total of 49 pregnant cows were identified and 16 of them had aborted at 3rd trimester. The age ranged from 5-7 year (median=5 year). For the 16 cases attack rate was 33% for aborting cows, 24% for close contact cows. The aborting cows were more likely to have brucellosis (OR: 35; 95%CI: 7-175; p=0.00) as compared to non-aborting cows and close contact cows were more likely to have brucellosis (OR: 5; 95%CI: 1.4-16; p=0.017) as compared to cows from other sheds. A total of 18.4% (23/125) farm cattle were found infected with brucellosis on serological testing. Brucellae were not detected in semen doses. Index case was a newly added (2 month before) exotic cow that was not screened by RBPT neither quarantined.

Conclusions Infected exotic cow was the most probable cause of the outbreak. Healthy animals got infection by licking aborted fetus or vaginal secretions of aborting cow. Vaccination of eligible calves using strain19, culling of confirmed cases, isolation of pregnant cows, screening & quarantine of newly purchased animals was recommended.

Zoonotic Diseases

Abstract Code: 2017-EOH-466
Presenter Name: Dr. Faisol Talukdar
Country: Bangladesh
Presenter Email: faisolvet@yahoo.com
Author: M. Sabrina, R. Sultana, M. Sazzad

Title Outbreak of Cutaneous Anthrax in Kalukhali Upazilla, Rajbari District, Bangladesh 2017

Background Anthrax is endemic and human infection has been identified in 15 of 64 districts of Bangladesh. Outbreak are associated with exposure to infected animals during droughts, floods or soil disturbances. On September 6, 2017 the civil surgeon of Rajbari District, reported six people with anthrax like lesions with exposure to one sick cow.

Purpose We investigated to determine cause of outbreak, describe the epidemiological and clinical features of cases, and to control the outbreak.

Methodology We selected cutaneous anthrax cases among the people presented with painless skin lesion including papular, vesicular and depressed black eschar living in Kalukhali, Rajbarj from 27th August to 09th September 2017. Human cases exposure with sick cow identified by contract tracing. We interviewed the suspected cases for clinical symptoms and types of exposure. We collected skin swabs from 5 humans and meat from the slaughtered sick cow and tested these samples by RT-PCR.

Results We found 11 cases from community by contract tracing and 6 cases from medical record. Most (71%) cases were male; median age of cases was 30 years. Among the cases, five females cleaned meat, nine males butchered the animal, and three males carried the meat. Most (59%) cutaneous lesions were found on hands. The cattle became sick after eating of "Kolmi shak" (water spinach) from a nearby flooded area. Cutaneous symptoms developed in 1 to 9 days (median 5 days). Bacillus anthacis was positive by PCR in one cattle meat sample and one human swab sample.

Conclusions This was the first reported anthrax outbreak in the Rajbari district. To control this outbreak, all the cattle in the village were vaccinated and an awareness program to avoid slaughtering of sick cattle was conducted. To prevent future outbreaks, anthrax vaccination of ruminants should be conducted yearly, and priority should be given in the flood affected district.

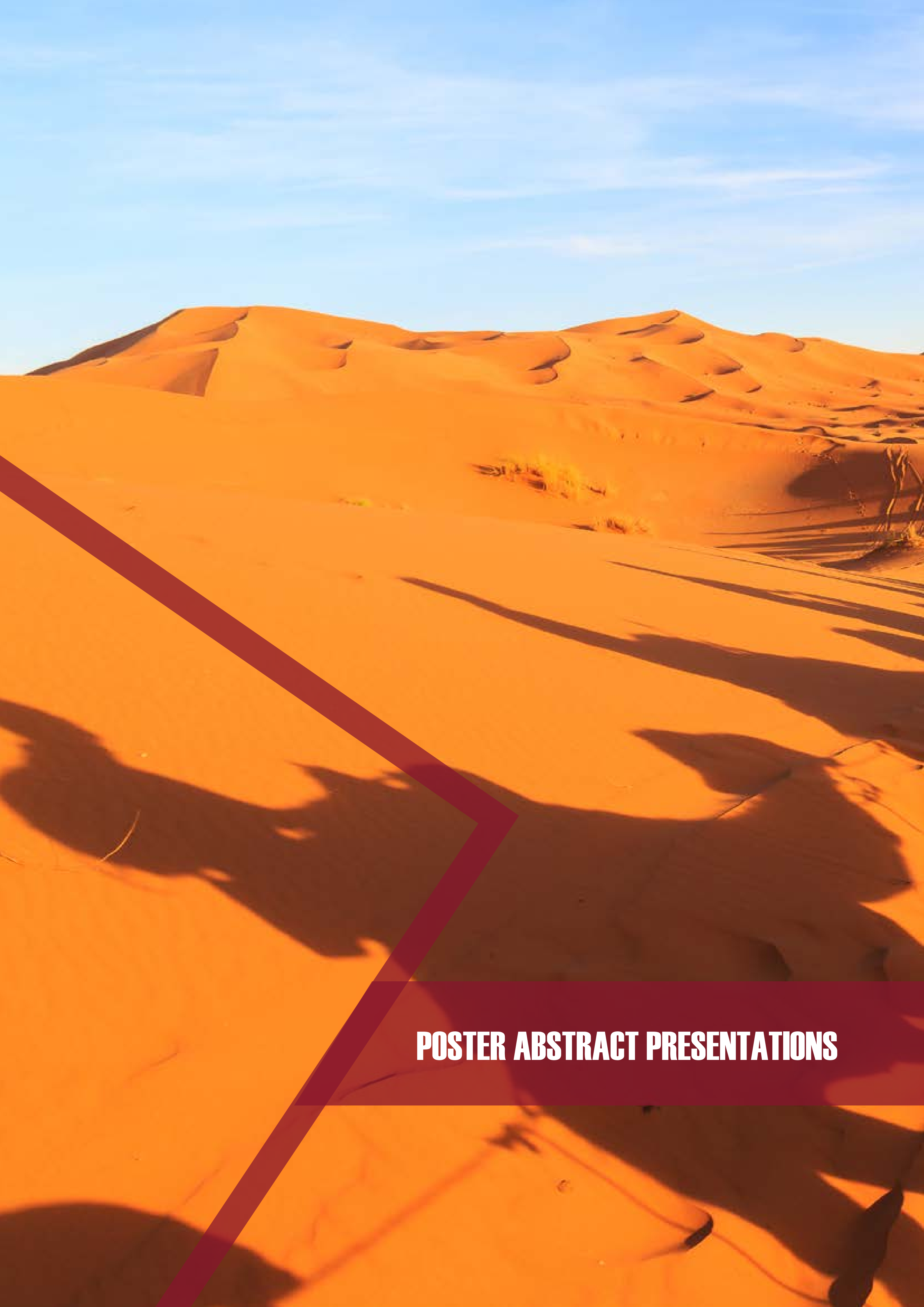

POSTER ABSTRACT PRESENTATIONS

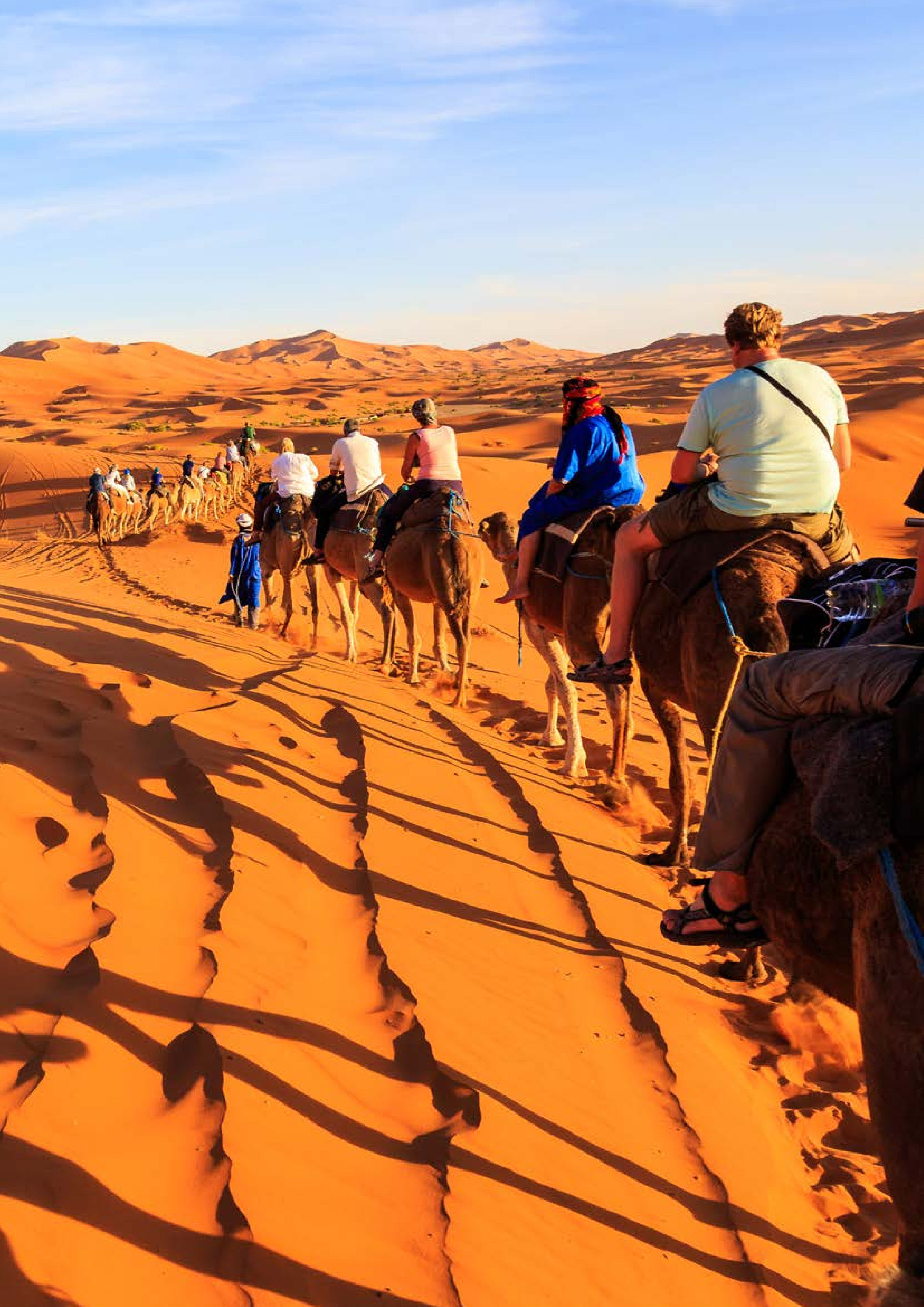

Anti-Microbial Resistance

Abstract Code: 2017-OTH-538

Presenter Name: Dr. Souad Redwane
Country: Morocco
Presenter Email: asmaekhattabi@yahoo.fr

Title Epidemiology and Determinants for Multidrug-Resistant Tuberculosis in Casablanca-Settat Region, Morocco 2012-2016

Background Multidrug-resistant tuberculosis (MDR-TB) refers to tuberculosis (TB) disease caused by strains of Mycobacterium tuberculosis resistant to isoniazid and rifampicin. In Morocco, the prevalence of MDR-TB is 0.1 % in new cases and around 8.7 % in among the restatement cases.

Purpose The aim of this study was to identify the epidemiology and determinants for MDR-TB, in Casablanca-Settat region, Morocco.

Methodology This was a case control (1 cases: 2 controls) study design. It included patients notified between January 1st, 2012 and the December 31st, 2016 at the Center for the diagnosis of tuberculosis in the region. The cases were patients with MDR-TB and the controls were patients with drug-sensitive tuberculosis. Socio-demographics, clinical characteristics, drug susceptibility testing (DST) and MDR-TB patient contact were analyzed. Data from MDR-TB cases and controls were analyzed using logistic regression.

Results One hundred sixty-eight cases and 336 controls were collected. A male predominance was noted in the two groups. The mean age was 37 years for cases and 35 years for controls. Fifty-one percent of the MDR-TB cases were considered primary resistance and the resistance rates for anti-tuberculosis agents were Isoniazid, 10.1%; Rifampin, 4.2%; Ethambutol, 1.6%; Streptomycin, 5%; Pyrazinamide, 6.6%. Multivariate analysis revealed that cases compared to controls were significantly more likely to have had a previous history of retreatment (OR = 60.9) or relapse after antituberculosis treatment (OR = 20.6) or a patient - contact with MDR-TB (OR = 10.5) or a hookah smoker (OR = 3.6) or have a low monthly income.

Conclusions Our results emphasize that previous history of retreatment with first-line anti-tuberculosis drugs is the main risk factor. The application of the directly observed treatment, Short-Course (DOTS) strategy is recommended.

Anti-Microbial Resistance

Abstract Code: 2017-EOH-225

Presenter Name: Dr. Kenza Bennani
Country: Morocco
Presenter Email: kenzabennani20@gmail.com
Authors: A. Khattabi, M. Akrim, A. Maaroufi

Title Epidemiological Profile of Multi-Drug-Resistant Tuberculosis in Morocco, 2007-2016

Background Multidrug-resistant tuberculosis (MDR-TB) is a public health problem worldwide. In Morocco, in 2014, the prevalence of MDR-TB was low with 1.0% in new TB patients and 8.7% in patients who have previously received anti-TB treatment. The drug-resistant TB surveillance system based on routine drug susceptibility testing (DST) was implemented in Morocco, to monitor and to control epidemiology of MDR-TB.

Purpose This study aimed to describe the epidemiological characteristics of MDR-TB in Morocco from 2007 to 2016.

Methodology This is a retrospective descriptive study, using data reported from 2007 to 2016. MDR-TB cases were defined as confirmed based on rapid diagnostic test "Xpert MTB/RIF" and Conventional DST results. The description focused on the analysis of temporo-spatial distribution, the individual characteristics and classification of the MDR-TB cases. Analysis was done using Epi-Info 7.

Results From 2007 to 2016, among 1063 DR-TB patients reported, 979 (92%) were MDR-TB confirmed cases. The number of MDR-TB cases increased by 65% between 2007 and 2016. About 54% of cases were reported from 2014 to 2016. A total of 735 (75%) cases were male, 705 (72%) aged 15-44 years, and 766 (78%) resided in the urban areas with 44% of cases in Casablanca-Settat region. A total of 873 (93%) cases have previously received anti-TB treatment. Of those, 578 (66%) were treatment failures. From 2007 to 2011, MDR-TB were not identified among new TB cases. The proportion of MDR-TB in new TB cases was 2% in 2011 and had gradually increased to 20% in 2016.

Conclusions Trend towards increased of MDR-TB cases was observed. MDR-TB affected young men, aged 15-45 years, resident in urban area and who have previously received anti-TB treatment. It is recommended to improve treatment outcomes for sensible tuberculosis patients, specially to fight against TB treatment failure; to maintain the efforts for MDR-TB detection among patients previously received anti-TB treatment and to strength MDR-TB detection among new TB patients.

Anti-Microbial Resistance

Abstract Code: 2017-OTH-359
Presenter Name: Dr. Mumtaz Ahmad Khan
Country: Pakistan
Presenter Email: mumtazahmad8166@gmail.com
Author: S. Nadeem-ur-Rehman

Title Surveillance of Methicillin-resistant Staphylococcus Aureus (MRSA) in Abbas Institute of Medical Sciences Muzaffarabad, Azad Kashmir, Pakistan-2017

Background Methicillin-resistant Staphylococcus aureus (MRSA) is an important nosocomial and community pathogen. It has a high impact not only on patient morbidity and mortality but also on hospitalization costs.

Purpose This study was carried out to determine the frequency of Methicillin-resistant Staphylococcus aureus (MRSA) in a tertiary care hospital.

Methodology This descriptive study was carried out in the Department of Microbiology, Abbas Institute of Medical Sciences Muzaffarabad from December 2015 to November 2017. Various clinical specimens of both indoor and outdoor patients were received in the laboratory of the hospital. All specimens were inoculated on appropriate media. Staph aureus was identified on the basis of colony morphology, Gram staining, catalase test, coagulase test and DNase test. All confirmed S. aureus isolates were screened for methicillin resistance using cefoxitin (Fox) disc. To check methicillin resistance, bacterial lawn was prepared on Mueller–Hinton agar and Fox disk was placed on it, followed by incubation at 37 °C for 18 to 24 hours. Antibiotic sensitivity was interpreted as per the CLSI guidelines (CLSI 2015).

Results Out of total 261 S. aureus isolates, 84(32.18%) were MRSA, while MRSA exhibited 100 % susceptibility to vancomycin and linezolid. Among MRSA, the frequencies of community acquired MRSA and hospital acquired MRSA were 26.2 % (n = 22) and 73.8 % (n = 62) respectively. Antibiotic sensitivity patterns were analyzed using WHONET software 5.6 versions.

Conclusions High frequency of MRSA was observed in the hospital. The MRSA is a potential threat to the public health as this bug can disseminate both in community and indoor patients causing severe clinical conditions. There is urgent need to improve infection control practices and establish a laboratory surveillance programs in healthcare settings to limit the spread of such resistant pathogens.

Anti-Microbial Resistance

Abstract Code: 2017-HIV/STI-230
Presenter Name: Dr. Farhana Naz
Country: Pakistan
Presenter Email: farhananazdr@gmail.com

Title Evaluation of Multi Drug Resistant Tuberculosis (MDR-TB) Surveillance System in Balochistan Province, 2015

Background Pakistan ranks 5th amongst 22 High burden countries (HBCs) with TB and 4th among 27 MDR-TB high burden countries. Pakistan contributes about 65% of T.B burden in Eastern Mediterranean Region. In Balochistan the patients are filtered from chest and medicine OPDs of tertiary care hospitals and from periphery the sputum of eligible MDR-TB patient is sent to the lab. Diagnosis of MDR-TB requires X-pert test. Fatima Jinnah Hospital Quetta has one site with two working machines. Recently, two more sites become functional in Balochistan at Turbat and Loralai.

Purpose To find out strengths and weaknesses of system and make recommendations for improvements.

Methodology Evaluation was performed during January 2016 for year 2015. Qualitative and quantitative assessment of system attributes on the basis of CDC updated guidelines for evaluating public health surveillance systems 2001 was done. Identification and engagement of stakeholders and interviews were held using semi structured questionnaire.

Results Surveillance system is very simple, easy to operate. System is less flexible but able to integrate with other systems. Data quality is good and complete, and reporting is based on clinical Signs and symptoms and confirmation with X-Pert, DST and culture. Acceptability is good, has good coordination with other health systems. Timeliness is good regarding reporting (100%) in time. System is stable, secure and available when required. Representativeness is good owing to involvement of public and private sectors. Sensitivity and PVP calculated was 93% and 100% respectively for year 2015.

Conclusions System is working effectively at PMDT sites, but Response of private sector is poor. There is integration with other health systems but there is need to increase public private collaboration.

Anti-Microbial Resistance
Abstract Code: 2017-EOH-416
Presenter Name: Mr. Arslan Salam
Country: Pakistan
Presenter Email: arslan_ahm691@hotmail.com
Author: Z. Akram, A. Ikram

Title Factors Responsible for Relapse Among the Tuberculous Patients Who Had Completed Their Anti-Tubercular Treatment and Frequency of Drug Resistance Among Them

Background For the successful Anti- tubercular treatment the relapse rate is an important indicator for the success of any treatment regimen, but it has not been normally measured. So a study was planned to examine the predictors of relapse among a cohort of sputum smear-positive pulmonary tuberculosis (PTB) patients who had successfully completed treatment of tuberculosis and the frequency of drug resistance among them.

Purpose To determine the frequency of drug resistance among the patients and determine factors associated with relapse among anti-tubercular treatment completed pulmonary TB patients under DOTS program.

Methodology After taking the informed written consent 200 relapsed patients were enrolled and are suspected to have relapse of pulmonary tuberculosis. From each patient two sputum specimens were collected for Ziehel Neelsen (ZN) smear microscopy and L-J culture. Those found positive on culture were subjected for drug susceptibility testing by standard drug proportion method on L-J medium .A semi-structured questionnaire was used to collect the information regarding the factors for relapse. Data was analyzed by using the SPSS.

Results A total of 200 patients were enrolled in the study. The major factors for the relapse were found to be previous addiction history (50%) and non-adherence to the previous treatment (52.5%). Around 167(83.5%) cases had not submitted the sputum at the end of previous treatment for which they were considered to be cured of TB. Among the Co-morbidities the diabetes is found to be very prominent as most the diabetic patients 45(22.5%) had relapse of TB. Regarding drug resistance the isoniazid and rifampicin resistance were 36% and 28% respectively.

Conclusions Important factors associated with the relapse of TB are addiction and non-adherence to the previous treatment so steps needs to be taken to stop the development of Drug resistance.

Maternal and Child Health
Abstract Code: 2017-EOH-65
Presenter Name: Dr. Rakan Aburoman
Country: Jordan
Presenter Email: RAKAN224@YAHOO.COM
Authors: A. Amatoq, A. Abuslaih, Y. Goued, S. Khresat

Title Epidemiology of HIV/AIDS Among Jordanians, 1986-2016

Background HIV continues to be a major global public health issue, having claimed more than 35 million lives so far. In 2016, 1.0 million people died from HIV-related causes globally. There were approximately 36.7 million people living with HIV at the end of 2016 with 1.8 million people becoming newly infected in 2016 globally. In 2015, an estimated 230,000 people were living with HIV in the MENA region. In the same year, there were roughly 21,000 new HIV infections and 12,000 AIDS-related deaths. MENA is increasingly becoming a region of concern regarding HIV and AIDS. Since 2001, new HIV infections have risen by 35%.

Purpose This study aimed to determine the prevalence and epidemiology of HIV/AIDS among Jordanian people.

Methodology Data were retrieved from the national surveillance system. All cases that had been reported among Jordanians during 1986-2016 were analyzed.

Results A total 325 Jordanian patients (261 males and 57 females and 7 unknown) were reported during the period 1986-2016. Overall, HIV prevalence is 5.4 per 100,000 pop. Their age ranged between 1year to 90 years, with a mean 32.8 (SD) of 12.5 year. The vast majority of patients aged between 20 and 40 years at the time of diagnosis. The number of cases increased sharply between 2000 and 2016. The mode of transmission was heterosexual in 54.1% of cases and blood in 18.7%. Man to man sex was reported in 11.3% of cases. The place of infection was outside Jordan in 70% of cases. Two thirds of patients (63.0%) were living in the Capital of Jordan.

Conclusions The number of HIV/AIDS is increasing among Jordanians by time being the highest in the age group of 20-40 years. HIV prevention campaigns, condom promotion and distribution, programs addressing gender-based violence, HIV testing and counseling.

Maternal and Child Health

Abstract Code: 2017-FWBD-46

Presenter Name: Dr. Sa'ed Assaf

Country: Jordan

Presenter Email: doctorassaf81@gmail.com

Title Stillbirth in Jordan

Background WHO estimates that, worldwide, almost four million stillbirths occur each year (7,8). Many of the conditions linked to stillbirth could be prevented or treated. No available data in Jordan about rates and common causes of stillbirth.

Purpose This study was conducted to determine stillbirth rate in Jordan and determine its main causes.

Methodology The study is based on secondary data analysis from the national study of perinatal mortality which was conducted between 2011 and 2012 in Jordan. Extensive data were collected on each woman included in the study and her newborn through interview and by abstraction relevant data from medical records. The stillbirth rate was calculated as the number of stillbirths per 1,000 live births plus fetal deaths (stillbirths). The causes of stillbirths were ascertained using the NICE classification system.

Results A total of 21,928 women gave birth to 22,591 births (22,330 live births and 261 Stillbirths) at 20 weeks of gestation or later in the 18 selected hospitals during the period 1st March 2011 and 30th April 2012. The rates of stillbirths were 11.6/1,000 total births for a gestational period of =20 weeks. According to the WHO and UNICEF's international reporting criteria, the corrected stillbirth rate was 8.2 per 1,000 total births. According to NICE classification, the main causes of stillbirths were maternal diseases (19.5%), unexplained immaturity (18.8%), congenital anomalies (17.6%), unexplained antepartum stillbirths (17.6%), obstetric complications (8.4%), placental abruption (5.7%) and multiple births (5%). It was judged that 34.5% of all fetal deaths were preventable and 30.3% were possibly preventable with optimal care.

Conclusions The rate of stillbirth in Jordan is comparable to that in other neighboring countries. However, many of the conditions that were shown to be linked to stillbirth in this study could be prevented or treated. Evidence-based and cost-effective interventions needed to be implemented to decrease the burden of stillbirth.

Maternal and Child Health

Abstract Code: 2017-EOH-22

Presenter Name: Dr. Noureddine Sakhri

Country: Morocco

Presenter Email: sakhriph@gmail.com

Title Opportunistic Infections and CD4 Cell Correlates Among HIV-infected Cases at Antiretroviral Health Care Centers in Morocco, 2015 – A Cross-sectional Study

Background Morbidity and mortality in HIV disease due to immunosuppression is leading to life-threatening opportunistic infections (OIs) during the natural course of the disease. In 2015, the HIV prevalence is low in general population and concentrated among key populations.

Purpose This study aimed to assess the prevalence and CD4 correlates of OIs among adult HIV-infected patients attending antiretroviral health care in Morocco during 2015.

Methodology We conducted a cross-sectional study among all adult HIV-infected patients admitted in the health care centers during 2015, who had acquired infection disease. Patients' opportunistic infection status was determined through clinical diagnosis and laboratory investigations. CD4 count was determined using flow cytometry technique. The clinical stage of HIV was identified by the classification of Centers for Disease Control and Prevention (CDC). We collected Socio-demographic and clinical data from patients' medical records. We performed statistical analysis by using Epi-Info 7.2.0.1 software. The appropriate test was applied, bivariate analysis was made, and the differences were significant when $p<0.05$.

Results 299 HIV-infected cases were included; 53% were males. The most represented age group was 25-34 years (36.1%). The mean age of the cases was 38.7 ± 16.8. The prevalence of OIs was 47.8%. Tuberculosis (65/299, 21.7%), Pneumocystis jiroveci pneumonia (40/299, 13.4%) and oral candidiasis (22/299, 7.4%) were the most frequently observed OIs. OIs were more prevalent among cases when the CD4 count was less than 200/mm3 (OR=7.140, $p<0.0001$) and among cases who had a CDC clinical stage C (OR=88.393, $p<0.0001$).

Conclusions Tuberculosis, Pneumocystis and oral candidiasis were the leading OIs, encountered by HIV-infected cases. CD4 count less than 200/mm3 and advanced CDC clinical stages of the disease were found to be predictors of OIs. Preventive measures and early treatment of HIV associated to OIs are crucial.

Maternal and Child Health

Abstract Code: 2017-HIV/STI-457
Presenter Name: Dr. Shams Ur Rehman
Country: Pakistan
Presenter Email: drshams142@gmail.com

Title Evaluation of Maternal Mortality Surveillance system in FATA, Pakistan, 2017

Background An estimated 529,000 maternal deaths occur each year who mostly belong to developing world. Maternal Mortality Ratio (MMR) in Pakistan is 276 maternal deaths per 100,000 live births. Poor socio economic and health infrastructure in FATA (Federally Administrated Tribal Areas) makes its population more vulnerable to maternal morbidity and mortality. The aim of this study was to evaluate the Maternal Mortality Surveillance System of FATA to identify its strengths & gaps and to suggest recommendations.

Purpose To assess the strengths and weaknesses of existing surveillance system.

Methodology A descriptive study was conducted during March-April 2017 at FATA Regional level using Updated CDC Guidelines for Evaluating Public Health Surveillance Systems, 2001. Data was collected by review of reports and filled forms. Semi-structured questionnaire was used and in-depth interviews of stakeholders were conducted from three systems, MNCH (Maternal & Neonatal Child Health), LHWs (Lady Health Workers) and DHIS (District Health Information System) Programs involved in Maternal Mortality surveillance to assess their system's performance attributes.

Results Systems were simple with uniform case definition. DHIS & LHWs Program were not flexible and had limited capacity to accommodate any additional information for new health event. Completeness of data was good in DHIS and MNCH but poor in LHWs Program. Acceptability was good. Representativeness was poor for MNCH & LHWs Program. Timeliness of LHWs Program was poor. Systems were not integrated. The sensitivity of the systems was very poor.

Conclusions All the systems are weak to detect Maternal Mortality. Coordination among all the three systems and with Khyber Pakhtunkhwa Health Department is important and to extend coverage to private health care facilities for timely and uniformly detection of Maternal Mortality cases. Improving damaged health infrastructure, maximizing representativeness of MNCH & LHWs Program, sustainability of funds, accountability & data quality are few challenges that need to be addressed.

Maternal and Child Health

Abstract Code: 2017-OTH-332
Presenter Name: Dr. Ahmed Ehsan
Country: Pakistan
Presenter Email: ehsanlarik@gmail.com

Title Risk Factors Associated with Mortality from Neonatal Tetanus in District Naseerabad Pakistan

Background Neonatal Tetanus (NNT) is a vaccine preventable disease.

Purpose To investigate Neonatal Tetanus cases to evaluate and determine risk factors for mortality from Neonatal Tetanus and to make recommendation for future strategies.

Methodology Case-control study was conducted in District Naseerabad. Matched for Age and sex, controls were taken from community with a ratio of 1:2 and interviewed. Case was defined "any neonate resident of Naseerabad with a normal ability to suck and cry during the first 2 days of life; and who between 3-28 days of age cannot suck normally and becomes stiff or has convulsions during 2016-2017." Hospital record and NNT surveillance data, case investigation form reviewed. Verbal autopsy and inspection of delivery scenes were conducted. Questionnaire was developed for data collection. Epi info version 7 was used for data compilation and analysis.

Results A total of 82 cases were identified. Mean age of cases was 8.6 days with range from 4-22 days. Male female ratio was 1.5:1. Overall Mortality was 18 (22%). 79 (96%) deliveries were conducted at homes by untrained traditional birth attendant with zero TT shots. Survived had significantly greater mean bodyweight, later onset of disease, hospitalized early and received TIG while generalized rigidity, fever, fits, cyanosis, undercurrent infections and respiratory arrest were significantly more common in fatal group. Delivery scenes were unclean on inspection. Significant statistical associations were found between umbilical cord cutting with unsterilized scissor (OR 12.9; CI 1.4 – 13; p value <0.05) and NNT. Umbilical cord tied with unsterilized thread (P = 0.002), ash used to cover umbilical cord (P: 0.004), no hand washing during delivery by TBAs (P: 0.006), illiteracy (P: 0.0002), low socioeconomic status (P: 0.003), and nonuse of antenatal care services played aggravating role.

Conclusions Poor TT immunization and umbilical cord cutting with unsterilized scissor were probable cause. Lack of awareness regarding antenatal care and poor hygienic deliveries were main risk factors. Enhancing antenatal care, improvement in TT vaccination coverage and awareness in community regarding TT vaccination were recommended.

Non-communicable Diseases

Abstract Code: 2017-NCD-11
Presenter Name: Dr. Mohammad Ismail Zubair
Country: Afghanistan
Presenter Email: drismailzubair@gmail.com
Author: T. Saeed Ali, N. Asad, N. Rizvi, A. Riaz

Title Exploring the Factors Affecting Quality of Mental Health Services at Primary Healthcare Level in Kabul-Afghanistan, 2016

Background According to World Health Organization (WHO) around two million Afghans (almost 7% of the total population) suffer from mental disorders. Mental health (MH) became a part of Basic Package of Health Services (BPHS) in 2005 and integrated as community-based MH services to Primary healthcare (PHC) level. Due to this initiative, the availability and accessibility of MH services have significantly increased, however the quality of services is still not up the mark.

Purpose Explore the factors affecting quality of MH services at PHC level in Kabul, Afghanistan.

Methodology This qualitative exploratory study was conducted from July to September 2016 at five PHC clinics in Kabul. A purposive sampling technique was used. In-depth interviews (IDIs) were conducted with patients' attendants and clinics' staff. Key informants' interviews (KIIs) were conducted with MH senior management staff of MoPH and implementing NGOs. Desk review was also done. The data were triangulated and manually analyzed by using thematic approach.

Results The findings of study draw a connection between the main influences on the quality of MH in Kabul—specifically financial constraints, security problems, weak stewardship, corruption, poor management and limited public health capacity of the MH department. Moreover, shortage and low quality of supplies, turnover of staff, high workload, lack of work plan, low salaries and no benefits for clinic staff were perceived to be the major barriers. Above all, fragile monitoring and evaluation system, weak coordination, low public awareness, and lack of community support were found to be the major factors undermining the quality of MH service at PHC clinics a in the country.

Conclusions Study findings highlighted various factors affecting quality of MH in PHC clinics which need to be considered by healthcare providers, health system managers, and policy makers for improving quality of MH services at PHC clinics in Afghanistan.

Non-communicable Diseases

Abstract Code: 2017-FWBD-72
Presenter Name: Dr. Abdul Wahhab Jewad
Country: Iraq
Presenter Email: alwehab1964@yahoo.com

Title Prevalence and Risk Factors of Diabetes Mellitus Type II Among Internal Displacement Persons in Al Najaf Al Ashraf Province, Iraq 2017

Background Iraq witnessed subsequent waves of mass displacement and exodus of millions of people to other provinces as a result of war with the takfiri groups. Many people were displaced to Al Najaf Al Ashraf province from the North and the central provinces.

Purpose To determine the prevalence and risk factors of D.M type II among Internal Displacement Persons (IDPs) in Al Najaf Al Ashraf province, 2017.

Methodology A cross-sectional study was conducted between Nov. - Jan. 2017 among sample of IDPs aged > 40 years selected randomly (multistage, systematic cluster sampling method). Thirty clusters were selected in the first stage and ten households from each cluster were selected randomly in the second stage. The selected IDPs were interviewed; a questionnaire including socio-demographic data and potential risk factors of D.M was filled. Associations between D.M and different risk factors were evaluated using the chi-square test. To assess the strength of association the odds ratio and 95% confidence interval of odds ratio was calculated.

Results The total number of interviewed participants was 300. Of those, 68 were confirmed as cases of D.M (prevalence 24%). Of confirmed cases, 24 (33%) were newly detected. Male: female ratio was 1:1.2. The significant risk factors were overweight (BMI >25) [odds ratio 2.74, CI 1.33- 5.67], high cholesterol level [odds ratio 2.90, C.I 1.63-5.16], low physical activity [odds ratio 1.80, CI 1.05-3.10], age (> 60 years) [odds ratio 1.94, C.I 1.06-3.56] and history of hypertension [odds ratio 2.24, CI 1.29-3.88).Conclusions One quarter of adult IDPs had D.M; one third are newly detected. Obesity was the most important modifiable risk factor. Screening of IDPs for D.M and Health education of IDPs on prevention and control of DM are recommended.

Non-communicable Diseases
Abstract Code: 2017-FWBD-496
Presenter Name: Dr. Monaf Faik
Country: Iraq
Presenter Email: dr_munaf.faik@yahoo.com
Title Factors Associated with Poor Adherence to Medication Among Diabetic and Hypertensive Patients, Iraq, 2016

Background The prevalence of Non-Communicable Diseases is increasing throughout the world. Drugs and lifestyle changes to control Hypertension and diabetes and associated conditions can only be effective through adherence to the overall prescribed regimen. Reduced adherence not only results in poor health outcomes, but it also has a significant impact on healthcare costs.

Purpose To measure prevalence and identify the demographic, social and personal factors affecting adherence in a sample of diabetic and hypertensive patients, Baghdad, Iraq, 2016.

Methodology A cross-sectional study was conducted on a sample of 584 male and female adults with HTN and/or DM. Two questionnaires were filled by direct interview. The first questionnaire for socio-demographic information and the second was the Morisky Medication Adherence Scale (MMAS-8). A score < 6 was considered poor, 6 - < 8 was considered moderate and 8 was considered good adherence.

Results The mean age was 48.9±11.8 years; 57.2% were females; 69.5% were currently married; and 83% were from urban areas. Around 73% had HTN, 53% had DM (92% type2 DM), and 27.1% had both HTN and DM. The prevalence of poor adherence was 44.3%, moderate was 33.2% and good was 22.4%. Multinominal logistic regression analysis revealed the following significant risk factor for poor adherence: Aging (OR=8.464; 95% C.I (2.04-35.07)), divorced/widowed (OR=2.452; 95% C.I (1.01-4.92)), and HPT duration < 5 years (OR=2.124; 95% C.I (1.22-4.86)).

Conclusions Poor adherence to medication is a significant problem among HTN and DM patients. Current study findings may be used to identify the population at risk of poor adherence who should be targeted for interventions to achieve better blood pressure and glucose level and hence prevent complications. There is a need to increase the awareness of patients towards the importance of medications. Future larger scale studies are needed to confirm the exact burden and the determinants of poor adherence in Iraq.

Non-communicable Diseases
Abstract Code: 2017-FWBD-449
Presenter Name: Dr. Samir Mounach
Country: Morocco
Presenter Email: samirmounach@yahoo.fr
Authors: H. Elberri, M. Obtel, R. Abouqal
Title Research on Non-Communicable Diseases in Morocco: A scoping Review 2000-2013

Background Non-communicable diseases (NCDs) are one of the major health and development challenges of the 21st century. Research is one of e major ways to understand their dynamic and to give evidences to adapt national strategies.

Purpose This study aims to describe the state of research on NCDs In Morocco from 2000 to 2013.

Methodology The study is a scoping review analysis of NCD publications in Morocco following Arksey's and O'Malley's five-stage methodological framework: identification of the research purpose, identification of relevant studies, study selection, data charting, and results summary. The literature search was conducted electronically using PubMed, searching articles from January 1, 2000, until December 31, 2013. The search strategy included NCD outcomes focused on the four prominent conditions: CVDs, cancers, chronic obstructive pulmonary disease and diabetes. Other NCD-related terms were used for the search strategy.

Results From 2000 to 2014, a total of 1111 publications on NDCs in Morocco were reviewed. 112 nationals and internationals researches institutes, faculties, International organizations had conducted studies on NCDs in Morocco. International researches constitute 6.5% of researches. NCDs Moroccan publications appeared in 357 revues, internationals in majority. The median of Impact factor of revues where Moroccan publications appeared is 0.8 [0.36,1.85], the mode is 0.29. The number of publications has increased from 2009 to 2014 (70 to 200). Cancer is the most included variable in NCD researches with 67%, diabetes and NCDs social determinants reached more than 10% of apparition in researches.

Conclusions Publication of researches on NCDs in Morocco should be enhanced quantitatively and qualitatively. Some measures can promote researches on NCDs as creating a national database on NCDs researches, Integration of the researches on national surveillance systems of NCDs.

Non-communicable Diseases

Abstract Code: 2017-FWBD-479

Presenter Name: Dr. Sanae Sendaoui
Country: Morocco
Presenter Email: sanaesendaoui@gmail.com
Author: M. Adnane, S. El Majjaoui, M. Aghoutane, A. Er-Raki, N. Benjaafar, A. Khattabi

Title Delays Determinants of Consultation, Diagnosis, and Access to Treatment of Women With Cervical Cancer in the National Oncology Institute in Rabat, Morocco

Background In Morocco, cervical cancer is the second women's cancers with an annual incidence of 12.3 per 100 000 Inhabitants and 82.2 % are diagnosed at advanced stages (IIB, III and IV of FIGO classification). It's related to the delays at every care steps.

Purpose The aim of this study was to identify factors associated with different delays in the management of cervical cancer: "Patient delay," "Diagnostic delay," "Access to treatment delay," and "Total delay."

Methodology It is a cross-sectional study, descriptive with analytic purpose including all cervical cancer patients admitted, from September 2016 to February 2017, at the National Institute of Oncology in Rabat, Morocco. Data were collected using questionnaire among eligible and consenting women. Dates of first symptoms, first consultation, diagnosis and of access to treatment were used to define different delays.

Results Among the 132 women, 81% were diagnosed at advanced stage FIGO (IIB, III and IV). The median of "Total delay" and "patient delay" were respectively 35 and 15.5 weeks. High risk for a long "Total delay" more than 6 months was observed for women with children (ORa= 6.8, CI 1.0- 46.0), having vaginal discharge (ORa=4.1, CI 1.02- 16.1) and for who the diagnostic of cancer was serious (ORa=4.3, CI 1.3 - 14.0). However, women had lower risk to have a "Total delay" more than 6 months when they had a family history of cancer (ORa=0.2, CI 0.05-0.60) and when they had used transport to consultation place (ORa=0.3, CI 0.08-0.82).

Conclusions This study allowed a better understanding of determinants of different delays in the management of cervical cancer, since the first symptoms until start treatment. Efforts must be directed to the population awareness to the target of the early detection program and to the recognition of cervical cancer symptoms in order to reduce the burden of morbidity and mortality of this disease.

Non-communicable Diseases

Abstract Code: 2017-FWBD-377

Presenter Name: Dr. Aslam Pervaiz
Country: Pakistan
Presenter Email: aslam_pervaiz92@yahoo.com
Author: M. Ali

Title Risk Factors for Non-Communicable Diseases (NCDs) Among Prison Population in Punjab, Pakistan

Background The burden of NCDs is disproportionately carried by prisoners. Chief Justice of Pakistan ordered for the improvement of health status of prisoners in Punjab prisons. Subsequently this study was planned, to highlight areas for action.

Purpose To assess the prevalence of potential risk factors of NCDs among prison population in Punjab, Pakistan.

Methodology A descriptive cross-sectional study was undertaken. A total of 301 male adult prisoners were enrolled through proportionate random sampling from 29 prisons of Punjab. Anthropometric measurements taken and information on age, education, type of prisoner, recreational/work activities, and smoking gathered by trained medical staff. Prison diet menu reviewed. Fractions of basic dietary ingredients and per day caloric value of diet worked out.

Results Mean age of participants was 40 years. Overall prevalence of obesity/overweight was 51% and Central obesity/overweight was 44%. Recreational facilities were not available in any prison. Convicted prisoners (CT) have been deputed for moderate physical labor. Under trial (UT) and condemned prisoners (CP) remained sedentary almost all the time. Prevalence of overweight/obesity among CP, UT and CT was 59%, 54% and 43%, respectively. Overweight/obesity depicted increasing trend with age. Being educated was significantly associated with obesity (OR 2.86; 95% CI 1.58-5.17, $P < .05$) and physical inactivity with central obesity (OR 1.6; 95% CI 1.03 - 2.69, $P < .05$). Active and passive smoking among prisoners was 44% and 78% respectively. Daily caloric intake was 2955 (recommended; 2150 for sedentary and 2350 for moderate physical work). Daily added salt, fiber and fat intake was 7gms, 5 - 6gms and 60gms respectively (recommended: added salt; < 5gms, fiber; 30gms and fat; <30gms).

Conclusions Prison population has shown high prevalence of multiple preventable risk factors of NCDs. We recommended revision of prison diet menu, arrangements for recreational activities and initiation of smoking cessation programs.

Non-communicable Diseases

Abstract Code: 2017-FWBD-57
Presenter Name: Dr. Mohammed Dahnan
Country: Yemen
Presenter Email: abofares4000@hotmail.com
Author: A. Almahdi, A. Assabri

Title Risk factors of End-Stage Renal Failure Among Hemodialysis Patients in Algomhory Hospital - Sa'adah Governorate Yemen, 2016

Background End-stage renal failure (ESRF) become a major public health problem and it is highly prevalent globally. More than 1.4 million patients receiving renal replacement therapy (RRT) globally, with an annual incident rate reaching 8%. In Yemen 7000 new patients every year, the estimated annual incidence of ESRF in Sana'a region is 385 per million population.

Purpose Identify the risk factors of ESRF in Sa'adah governorate of Yemen and provide recommendations to prevent and control ESRF.

Methodology A hospital-based case control study of ESRF patients attending Aljomhory Hemodialysis center in Sa'adah. We interviewed 349 persons, 86 of them were cases attending Aljomhory Hemodialysis center for regular hemodialysis and 263 were control who are healthy persons, the case definition of ESRF is GFR (< 15 mL/min/1.73 m^2) or very high albuminuria (>300 mg/24 h). Analysis was performed using Epi Info, OR was calculated.

Results The mean age for the cases and control was 43.3 ± 17.7 and 32.3 ± 12.98 years respectively, with slight male predominance among cases 55%. Illiteracy rate was 49% and 38% among cases and controls respectively OR 2.13 (95% CI, 1.28-3.55). Hypertension was 49%, 10% among cases and controls respectively OR 8.3 (95% CI, 4.5-14.9). Diabetes mellitus was 13%, 5% among cases and controls respectively OR 3.05 (95% CI, 1.2-7.32). Urolithiasis was 41%, 3% among cases and controls respectively OR 21.87 (95% CI, 9.6-49.9). Recurrent infection of kidney or urinary tract was 79%, 28% among cases and controls respectively OR 9.64 (95% CI, 5.34-17.31), Family History of ESRF 29%, 5%, among cases and controls respectively OR 7.88 (95% CI, 3.81-16.29).

Conclusions Hypertension, recurrent urinary tract infection, urolithiasis, and family history of ESRF were risk factors for ESRF in Sa'adah, north of Yemen. Follow up for those factors well help to prevent and control ESRF.

Outbreak Investigation/FWBD

Abstract Code: 2017-EOH-155
Presenter Name: Dr. Slavica Maris
Country of Nationality Serbia
Country of Work Other
Presenter Email: slavicamaris@me.com
Author: A. Uzelac Škoric, D. Durlevic, V. Risimovic, S. Randelovic, Z. Vidakovic

Title An Outbreak of Staphylococcal Food Poisoning Among School Children, Belgrade, Serbia, October 2017

Background Staphylococcal food poisoning is one of the most common food-borne disease worldwide. The incidence of food poisoning is underestimated, in Belgrade several individual cases are registered each month. On October 3, 2017, the Institute of Public Health of Belgrade received the information of potential outbreak-20 cases of gastroenteritis in one school in Belgrade.

Purpose To investigate the potential outbreak and to identify the etiologic agent and the source of the infection with intention to recommend the control measures.

Methodology A retrospective cohort study was used. Study population was 2,276 children from 21 schools in Belgrade. The standardized epidemiological questionnaire for data collection was used. A case was defined as a consumer of macaroni with Bolognese sauce prepared in one catering company and served at lunch time (11:30-14:00h) in 21 schools at 2nd October 2017 vomiting AND/OR diarrhea AND/OR stomach cramps developed within 8 hours.

Results In total 176 cases from 21 schools in Belgrade were identified with a median age of 7.8 years (range:6-12). The onset of illness for all cases was the 2nd October 2017. Macaroni with Bolognese sauce was associated with illness (RR=114.5, 95% CI:15.5 to 785.1). First and second graders in schools were at greatest risk of food poisoning comparing with older students (RR=76.2, 95% CI:10.7 to 543.3; RR=33.2, 95% CI:4.6 to 242.2). Two samples of food (macaroni with Bolognese sauce on 2nd October and cabbage salad on 3rd October) were collected in two schools. Staphylococcal enterotoxins were detected. In the throat/nose swabs of five food handlers, staphylococcus aureus was detected.

Conclusions As transmission patch in the outbreak were macaroni with Bolognese sauce contaminated with staphylococcal enterotoxins. The potential source of infection could be food handlers in whose throat/nose swabs Staphylococcus aureus were detected. A similar staphylococcal food poisoning among students from 20 schools was registered in October 2013 (263 cases). It is necessary to improve knowledge and sanitary-epidemiological habits for food handlers.

Outbreak Investigation/VPD

Abstract Code: 2017-EOH-483
Presenter Name: Dr. Abdul Hadi
Country: Pakistan
Presenter Email: drhadikakar@gmail.com
Author: A. Saeed

Title Measles Outbreak Investigation in Village Kakshai, District Zhob, Pakistan- 2016

Background On December 27, 2016, District Health officer Zhob reported five suspected measles cases with three expiries from village Kakshai (with population of 2508) District Zhob to provincial disease surveillance & response unit and requested for investigation.

Purpose A FELTP fellow was assigned were to confirm, investigate outbreak identify and risk factor and suggest control measures on 28th December 2016.

Methodology A case was defined as any person resident of village Kakshai District Zhob with high grade fever, maculopapular rash and having any of following; conjunctivitis, coryza or cough, from December 13, 2016 to January 11, 2017. Descriptive study was conducted through active case finding, deaths were verified by verbal autopsy and vaccination status was assessed. Blood samples collected and sent to National Institute of Health.

Results Total 35 cases (30 through active case finding) including nine deaths were identified (Overall AR: 3.2% and CFR: 25.7%). Males were most affected (n=19, 54%). Mean age was 5 years (range: 1-22 year). Most affected age group was 0-5 years (n=22, 63%). Coryza (n=25,71%), conjunctivitis (n=11, 31%), and diarrhea (n=10, 29%) were most frequent symptoms. All 210 children (12-23months age) and 9 deceased with pneumonia were unimmunized. Vaccination efficacy could not be calculated as entire village was unvaccinated. Reason for non-vaccination were given as vaccinator did not visit (n=21, 60%) and unawareness about routine vaccination (n=8, 22%), and misconceptions/misbeliefs about vaccination (n=6,17%). Socioeconomic, nutritional and hygiene status of village were very poor.

Conclusions Most probable cause of outbreak was no routine immunization and poor nutritional status of children of village. Misconceptions and unawareness found regarding RI in affected Village. We recommended Immediate Mass vaccination of whole village and a total 250 children were vaccinated. Awareness sessions conducted among community regarding importance of RI, Health/Personal Hygiene. Surveillance for measles and other VPD diseases needs improvement to prevent future outbreaks.

Outbreak Investigation/VPD

Abstract Code: 2017-EOH-426
Presenter Name: Dr. Aftab Khaskheli
Country: Pakistan
Presenter Email: draftabali@gmail.com
Author: N. Masood

Title Measles Outbreak Investigation at Village Jogi Sabbri, District Umerkot-2017

Background On 25th September 2017, District Health Office Umerkot reported 14 cases of fever with rash at Village Jogi Sabbri, to Provincial Disease Surveillance and Response Unit Hyderabad. On 26th September 2017, a team of FELTP was deputed.

Purpose To estimate the magnitude, evaluate risk factors and formulate control measures.

Methodology The investigation was carried out from 26th September to 10th October 2017. Review of reported cases and active case finding was carried out and verbal autopsies were conducted for deceased children. A case was defined as sudden onset of fever with maculopapular rash and one of the following clinical features; cough, coryza and conjunctivitis in a resident of Village Jogi Sabbri from 22nd August to 10th October 2017. Vaccination coverage survey was done using 30 x 7 cluster-sampling strategy. Blood samples were sent to NIH Islamabad for Lab confirmation.

Results A total of 67 cases were identified; 53 through active case search. The cases included 7 deaths (CFR 10%). Median age was 36 months (range 7-144months) and females were more affected (53.7%). Overall attack rate (AR) was 7.6% with 36-59 months being the most affected (n=23; AR 9.9%). Apart from fever and maculopapular rash, coryza (82%) and conjunctivitis (67%) were the most common symptoms. A total of 210 eligible children were assessed in the vaccination coverage survey. The AR in unvaccinated was 17.9% and AR in vaccinated was 3.8 % with vaccine efficacy of 78.9%. The most common reason for non-vaccination was misbelief on vaccination (93%). Out of the blood samples sent 16 were positive for measles IgM.

Conclusions Low immunization status was the most probable cause of the outbreak. Refusal to vaccination due to misbelief was the major contributor. Community was sensitized for vaccination through religious leaders and health education imparted. Follow up vaccination activity was done in which 512 children were vaccinated.

Outbreak Investigation/VPD

Abstract Code: 2017-EOH-506
Presenter Name: Dr. Ahsan Khan
Country: Pakistan
Presenter Email: nayyarjamal@hotmail.com
Author: S. Wasif

Title Investigating the Measles Outbreak in District Dir Upper, Khyber Pakhtunkhwa (KPK), Pakistan, October 2016

Background On 3rd October 2016, a local newspaper reported one death and five suspected cases of Measles from union council (UC) Chapara, District Dir Upper. Disease Surveillance & Response Unit (DSRU) launched an outbreak investigation.

Purpose To identify the possible risk factors of the outbreak and make recommendations for future prevention and control.

Methodology A Descriptive study was conducted between October 4-6, 2016 at UC Chapara. Medical and Vaccination records of suspected cases were reviewed along with active case search. A suspected case was defined as "any person of age = 60 months with sudden onset of fever (> 38.0C) and rash with or without red eyes, cough and coryza, resident of union council Chapara from 30th August to 29 October 2016". Data is collected on pretested questionnaire. Five blood samples were sent for laboratory confirmation. Vaccine coverage survey was conducted. Descriptive statistics and attack rates were calculated.

Results A total of 21 cases with one death were identified. Overall attack rate was 0.05% and case fatality rate was 4.7%. 34% (n=7) were vaccinated while 66% (n=14) were unvaccinated. Among cases 44% (n=9) were male and 56% (n=12) were female. Most affected Villages were Shahkanai with 52% (n=11) of total cases followed by Bandokhoar 38% (n=8) and Kamarsar 14% (n=3). Mean age of cases was 23months (range 12 - 60). Age specific Attack rate (3.8%) was highest among 0 to 48 months of age group. Attack rate among unvaccinated & vaccinated children was 14.1% and 1.5% respectively. Total 210 children surveyed in which 52% (n=110) were found vaccinated. Three samples were found positive.

Conclusions The outbreak identified three high risk villages with potential unvaccinated children due to hard areas, limited outreach activities and difficult access to healthcare facility. Measles mopping up activity with Vitamin A administration was conducted. Health education sessions were conducted in the community.

Outbreak Investigation/VPD

Abstract Code: 2017-EOH-491
Presenter Name: Dr. Bisma Memon
Country: Pakistan
Presenter Email: drbisma@gmail.com
Author: N. Masood

Title Measles Outbreak Investigation in Village Ahmed khan Babur, District Badin, Pakistan-2017

Background On 16th May 2017, Civil Hospital Hyderabad notified three cases of fever with rash from Village Ahmed Khan Babur, District Badin (population <15 years of age=2600) to DG Health Office Sindh. In response to this a team of FELTP fellows was assigned the same day to investigate the suspected outbreak.

Purpose The objectives were to assess the magnitude, evaluate the risk factors and recommend control measures.

Methodology Health facility records were reviewed and active case finding was done. A case was defined as sudden onset of fever and rash with or without cough, coryza and conjunctivitis in a resident of any age of Village Ahmed Khan Babur from May 5 to June 10, 2017. Data was collected using a pre-tested questionnaire. A vaccine coverage survey in eligible children (12-23 months) was done using 30 x 7 cluster sampling strategy. Blood samples were sent for confirmation of measles-specific IgM antibody to National Institute of Health. Frequencies were calculated, attack rates computed, and vaccine efficacy determined.

Results A total of 35 cases (32 through active case search) were identified. Mean age was 42 months (ranged 8 months to 9 years) with male preponderance (n=25; 71%). Overall attack rate (AR) was 1.34% with 12-48 months being the most affected age group (n=23; AR 8%). Apart from fever and rash, cough (n=25; 71.4%) and diarrhea (n=16; 45.7%) were the most prominent symptoms. Out of 18 samples 14 tested positive for measles specific IgM. On vaccination coverage survey in 210 children, AR in unvaccinated children was 11.9% and in vaccinated children was 2.54% with vaccine efficacy of 78.6 %.

Conclusions The most probable cause of the outbreak was low immunization status. Door to door vaccination campaign was initiated in the village and Vitamin A was also administered.

Outbreak Investigation/VPD

Abstract Code: 2017-EOH-83
Presenter Name: Dr. Muhammad Hakim
Country: Pakistan
Presenter Email: dr.muhammad.hakim@gmail.com
Author: A. Khan

Title Investigation of Measles Outbreak in Union Council Anjani and Union Council Kadda, Orakzai Agency-Federally Administered Tribal Areas May-2017, Pakistan

Background Measles is readily spread to susceptible individuals, a leading cause of morbidity and mortality in the developing countries including Pakistan. During week, 20 of May-2017 total 17 measles patients reported, eight cases from UC Anjani and seven cases from UC Kadda Orakzai Agency.

Purpose The objective of a field investigation to identify gaps and provide recommendations for measles outbreak response and immunization program improvement.

Methodology The study conducted from 25th May to 8th June 2017.A case of measles was defined as "A person of any age resident of UC Anjani and UC Kadda, with rash, fever and at least one of the following cough, coryza and conjunctivitis presenting between 1st May to 8th June-2107. Epidemiological, clinical and risk factors were recorded and analyzed by using Epi Info.

Results There were 51 cases and Zero deaths identified of measles during outbreak investigation. The symptoms were fever, rash, coryza and conjunctivitis in 51(100%) and pneumonia in 41(80%) of the 51 cases. About 22 (45%) cases were males and 28(55 %) were females. The mean age of cases was 56 months, while most of the cases 26 (51%) of 51cases were in the age group of 6-10 years. All of the 51(100%) respondent parents were illiterate. The vaccination status of all 51 (100%) cases were zero doses. The index case was on May 1, 2017 and cases increased from 5th May 2017.The EPI survey in UC Anjani were 40% RI coverage. The EPI survey in UC Kadda were 80% RI coverage. Majority 41(80.39 %) of 51respondents were unaware about RI, whereas 29 (56.36%) of 51 respondents stated that the vaccinator did not visit their areas. The Vaccine Efficacy were 28%.

Conclusions This outbreak was a result of vaccination coverage gap. Functionalization of EPI centre, vaccinations of all children and mass education was strongly recommended. Outreach activity must be carried out regularly to reach scattered population. Surveillance for vaccine preventable diseases should be strengthened to prevent outbreaks.

Outbreak Investigation/VPD

Abstract Code: 2017-EOH-258
Presenter Name: Dr. Mawahib Rashid
Country: Sudan
Presenter Email: talentsforall@yahoo.com
Author: A. Bahier

Title The Carriage Rate of Neisseria Meningitides, Serotypes and Risk Factors in Children 1- 15 years, Khartoum State, Sudan, 2016

Background Epidemic meningitis in African meningitis belt remains an important public health problem. Sudan as one of 26 countries of the belt remain suffering of the diseases. Studying meningococcal carriage and human-to-human transmission is a key because Neisseria meningitides (Nm) hosted only by humans. Transmission of the meningococcal occurs usually through close contact or by air-borne large respiratory droplets from asymptomatic human carriers. Identifying the sero-types and the risk factors are essential to implement suitable control measures such as new vaccine introduction.

Purpose To study the frequency of colonization of Neisseria meningitides as well as the distribution of the different N. meningitides serotypes isolated from in healthy children from 1-15 years in Khartoum State 2016.

Methodology This is a cross-sectional community-based study in Khartoum State, Sudan. A total 824 of pharyngeal swabs were taken from children 1-15 years old using multi stage cluster sampling technique and processed using conventional PCR in order to identify Nm type and subtypes.

Results The overall prevalence of Nm was 18.1%(149 out of 824). In under 5 years children, the prevalence was 18.2% (25/137). The prevalence was 23.5% in children 5-15 years (124 out of 528). The dominant identified sero-groups was type C. Age group 5-15 years, living in crowdedness environment and passive smoking were in great risk to be a carrier positive.

Conclusions The dominant sero-type was C with no detection of Nm type A which was predominant in Sudan, which is most probably due to the effect of conjugate A meningitis vaccine introduction. Risk factors associated with high risk acquisition rate of Nm carriage included age group 5-15, crowdedness and passive smoking. Therefore, it is recommended to introduce a new meningitis conjugate vaccine including sero-type C.

Respiratory Diseases

Abstract Code: 2017-EOH-337
Presenter Name: Dr. Noureddine Sakhri
Country: Morocco
Presenter Email: sakhriph@gmail.com

Title Virological Monitoring in the System of Epidemiological Surveillance for Influenza and Acute Respiratory Diseases in Epidemic Seasons of 2015/2016 in Morocco

Background Influenza has a huge impact on morbidity and mortality worldwide. The threat of new and highly pathogenic respiratory pathogens emerging around the world requires continuous global influenza surveillance. Sentinel surveillance of influenza-like illness (ILI) and severe acute respiratory infection (SARI) have been established since 2009. A routine outpatient surveillance is also in place.

Purpose The aim of this study was to provide an overview of 2015-2016 influenza season in Morocco.

Methodology Both epidemiological and virological data were collected and analyzed on a national level and weekly basis, during the period of surveillance, October 2015 to May 2016. For laboratory confirmation of influenza, from combined nasal and throat swabs Real time polymerase chain reaction (RT-PCR) was used.

Results A total of 961 samples from patients with ILI and SARI were collected. The number of positive samples was 174 (18.1%). The highest proportion of laboratory-confirmed influenza cases was 64.4% in week 6/2016. Based on laboratory-confirmed SARI cases (sentinel sources) influenza activity peaked in week 8/2016 with 89.7% of confirmed cases. Medium-intensity influenza activity was reached in week 8/2016. All three viruses were confirmed: A(H1N1)pdm09, A(H3N2) and B. A(H1N1)pdm09 virus was responsible for 70.7%, A(H3N2) for 17.8% and virus type B for 11.5% of all confirmed cases. Influenza A(H1N1)pdm09 viruses predominated first and then type B with A(H1)pdm09 co-dominated from week 12/2016.

Conclusions In Morocco, influenza activity started during first week of 2016. Existing high match between primary care data, the percentage of influenza positive samples and SARI hospitalizations indicates the adequacy of universal and sentinel surveillance of ILI and sentinel SARI surveillance.

Surveillance System

Abstract Code: 2017-HIV/STI-262
Presenter Name: Dr. Rania Attia
Country: Egypt
Presenter Email: rmaghawrya@yahoo.com
Author: A. Kandeel, H. Abu Elsood, S. Refaey, S. Alshorbagy

Title Evaluation of National Surveillance of Hepatitis C, Cairo governorate, Egypt, 2016

Background Hepatitis C viral infection is endemic in Egypt. Egyptian demographic health survey conducted in 2015 concluded that 4.4% of the populations have been infected. In 2001, the Ministry of Health and Population (MOHP) began developing the National Electronic Disease Surveillance System (NEDSS), an Egyptian communicable disease reporting system. In 2006, the web-based system was established and started in all Governorates in 2012. Acute viral hepatitis infection is one from the reportable diseases on the system.

Purpose Detect gaps of the system and give recommendations.

Methodology Reported cases of acute viral hepatitis were extracted from the NEDSS for year 2016. Hepatitis C cases were classified according to the criteria of surveillance case definition of the national surveillance guidelines into suspected, probable and confirmed. CDC guidelines were used to evaluate the system attributes. A structured questionnaire was designed to interview 22 participants randomly selected to evaluate the system on four levels: Epidemiology and surveillance general administration at the Ministry of Health (central level), Health directorate level, Health district level and Fever hospital level (peripheral levels).

Results The total number of reported hepatitis C cases in Cairo in 2016 was 676 (527(78%) confirmed cases, 1 probable case and 148(22%) suspected cases). The system was simple (85.4%), flexible (85%), stable (77%), and acceptable (67%). Twelve variables were used for evaluating data completeness with mean 74.6%. Timeliness was not well expressed. The system had low representativeness and fairly sensitive but with 78% Predictive Value Positive.

Conclusions Surveillance system was useful, simple, flexible, stable and acceptable. It needs to increase the capacity involvement to ensure data completeness, expanding number of reporting sites of hepatitis C surveillance to include liver centers, private hospitals and clinics. Further modification for involvement of disease risk factors is recommended. Enhanced surveillance will improve the completeness and timeliness of the data.

Surveillance System

Abstract Code: 2017-HIV/STI-414
Presenter Name: Dr. Ayad Kareem Hwayyiz
Country: Iraq
Presenter Email: ayadalmukhtar70@yahoo.com

Title Trend of Treatment Outcome of Tuberculosis in Baghdad-Kerkh, Iraq, 2009-2015

Background Iraq ranked 8th among 22 EMRO countries and 108th globally (from 213 countries) in the TB burden according to estimates of TB incidence. The directly Observed Treatment Short course (DOTS) was first introduced into Iraq National Tuberculosis Program (NTP) in 1998 and expanded to all governorates in 2008. The case detection rate in Iraq is still 60% (recommended 70%) but the cure rate is 89% (recommended 85%). Baghdad-Kerkh is the western side of Baghdad with 4 million inhabitants.

Purpose The objective of this study was to assess the treatment outcome of tuberculosis patients in Baghdad-Kerkh-Iraq, 2009-2015.

Methodology Records' based cross-sectional study was conducted in the Chest Diseases Clinic in Baghdad-Karkht. We reviewed and analyzed treatment outcome data of all registered TB patients for the period 2009-2015. WHO DOTS standard definitions of the TB case, cure rate, treatment success rate, defaulter rate, relapse rate and failure rates were followed.

Results A total of 6,210 TB patients were reported. Male to female ratio was 1.1:1. The case detection rate ranged from 55% in 2009 and 72% in 2015. The treatment success rate (TSR) increased from 70% in 2009 to 94% in 2015. The cure rate varied from 20% (2011) to 37% (2015). Defaulter rate decreased from 24% (2009) to 2% (2015). The death rate was 3-5% and failure rate was 1-2%. The most affected age group in treatment failure was 15-24 years while in treatment default was 25-34 years, and in relapse was 35-45years. The highest average TSR was in I'elam district (94%) and the lowest was in Dora district (66%).

Conclusions All DOTS program indicators had improved over the seven years period in Baghdad-Kerkh. Sustained close supervision and monitoring can enhance the momentum and help in reducing TB burden.

Surveillance System

Abstract Code: 2017-EOH-88
Presenter Name: Dr. Hind Ezzine
Country: Morocco
Presenter Email: ezzinehind@gmail.com
Author: M. Benhafid, A. Rguig, A. Maaroufi, I. Jroundi

Title Trends in Admissions and Ambulatory Visits for Rotavirus Acute Gastroenteritis in Children Younger Than 5 Years of Age in Morocco Before and After Rotavirus Vaccine Introduction, June 2006-April 2014

Background In Morocco, acute gastroenteritis (AGE) is a public health problem. Several strategies to face it have been developed, including the introduction of the monovalent anti-rotavirus vaccine (RV) into the National Immunization Program in 2010.

Purpose To evaluate the impact of this vaccine among out and inpatients under 5 years old with AGE four year after the rotavirus vaccine implementation.

Methodology Pre- and post-vaccination epidemiological surveillance data from the national sentinel surveillance system and from the national registry of diarrhea disease among children under five years old were analyzed to describe the clinical and epidemiological characteristics, the trends of the admissions and visits for AGE from 2006 to 2014 and to estimate the proportion of RV positive and the Vaccine Effectiveness.

Results The decrease of numbers of AGE was about 5.2% and mainly among children aged 0-11 months. The proportion of AGE RV positives decreased from 37 to 31.1% after the vaccine introduction; it was statistically significant among the 0-11 months (from 38.8 to 28.1%, p=0.009). The proportion of AGE-RV positives among inpatients with AGE decreased from 97% to 91.7% (p=0.022). Diarrheal cases without dehydration increased from 7.8% à 11.1% (p< 0.000). AGE-RV positive illness was 2.3 times more frequent among unvaccinated children. Vaccine effectiveness was 57%. Infections due to Genotype G1P [8] decreased from 56 to 40% after introduction of the vaccine (p< 0.000) while G2P [4] became more frequent (from 13% to 21%; p=0.015).

Conclusions This study showed a significant reduction of the proportion of RV AGE and its severe form among the vaccine target population. The strength of RV sentinel surveillance should be insured to able the conduct of other epidemiological and economical studies to evaluate the impact of the vaccination among Moroccan children.

Surveillance System

Abstract Code: 2017-OTH-444

Presenter Name: Ms. Mariama Bahalou
Country of Nationality: Morocco
Presenter Email: douaae2005@gmail.com
Author: M. Khouchouaa, A. Khattabi

Title Epidemiological Profile of Tuberculosis Disease in Meknes, Morocco: Descriptive Analysis, 2011-2015

Background Globally, the estimation of new reported tuberculosis (TB) cases was 9.6 million in 2014 making this disease a major global public health issue. In Morocco, despite the progress made, the disease remains of high incidence and requires more effort to reach its control.

Purpose The objective of the study was to describe the epidemiological profile of tuberculosis in Meknes Prefecture in terms of its morbidity and mortality.

Methodology It is a descriptive analysis of TB cases reported between 2011 and 2015 at regional diagnostic center of tuberculosis and respiratory diseases. We performed analysis by using Microsoft Excel and Epi Info 7.

Results Based on records, the number of reported tuberculosis cases was 3797 over the five years. Its average incidence was higher in Meknes than the national rate with respectively 99 cases compared to 85cases/100 000 populations.88.5% of cases live in urban areas in poor and overcrowding conditions. The median age is 32 year showing male predominance with a sex ratio male/female at 1.4. Primary pulmonary TB was predominant in 51% of cases. The TB detection methods were in passive screening in 54% of cases and in contact tracing in 1% of cases. Confirmed tuberculosis cases represent 41% of reported cases. The treatment completion rate is 84.2%. The estimated case fatality rate is 3.6%. Treatment failure is 0.7% and the rate of lost to follow up is 7.1%. Tuberculosis VIH co-infection varied from 0.1% to 0.4%.

Conclusions The incidence of tuberculosis in Meknes remains high compared to the national level. Thus, itis imperative to reinforce the earlier detection; improve the contact tracing, detection methods of cases for their confirmation and treatment and to reduce the proportion of the lost to follow up as well.

Surveillance System

Abstract Code: 2017-HIV/STI-493

Presenter Name: Dr. Rabia Subuktageen
Country: Pakistan
Presenter Email: drrabia_babna@hotmail.com
Author: N. Azam, A. Baig

Title Evaluation of Disease Early Warning System for Typhoid Fever in Multan Cantonment, Pakistan-2016

Background Typhoid fever is the sixth most common cause of death in Pakistan and its prevalence is estimated to be 412 cases per 100,000 population per year. The Disease Early Warning System (DEWS) in Multan District was evaluated.

Purpose This study aimed at evaluation of the strengths and weaknesses of the DEWS for detection of Typhoid fever cases and to recommend improvements in the system.

Methodology DEWS surveillance system in Multan Cantonment for Typhoid fever was evaluated. Literature was reviewed while departmental documents reports were evaluated for year 2016. Qualitative and quantitative system attributes were assessed using Updated CDC Guidelines for Evaluating Public Health Surveillance Systems, 2001. Stakeholders were identified and interviewed using a pre-tested semi-structured questionnaire.

Results Reporting mechanism of system was simple with use of standardized case definition. The system was flexible to accommodate other related health events and was acceptable to all stakeholders. The reports were 100% complete and 83% reports were received within stipulated time limits, but the system was lacking feedback mechanism to the grass root level. The system was stable as it was dependent on public funds. The representativeness was not good because it had no involvement of private sector. Sensitivity of the system was 0.07% while predictive value positive (PVP) was12.69%.

Conclusions Sensitivity and representativeness of the system needs to be improved by increasing coverage of the system and involvement of private sector. A mechanism is required to be established for sharing feedback to all levels of the systems.

Surveillance System

Abstract Code: 2017-HIV/STI-485
Presenter Name: Dr. Sanam Hussain
Country: Pakistan
Presenter Email: sanam.rehan66@gmail.com
Author: N. Masood, B. Memon

Title Evaluation of Tuberculosis Surveillance System in District Hyderabad, Province Sindh, Pakistan-2017

Background Tuberculosis (TB) is an immense public-health threat, accounting for 10.4 million new cases with 1.4 million tuberculosis related deaths worldwide in 2016. Eastern Mediterranean Region (EMRO), reported 38% morbidity and 39% mortality of the global TB burden, 2013. Pakistan shares 61% tuberculosis cases in EMRO region. In Pakistan, estimated Tuberculosis is 270/100,000 population/year.

Purpose To identify strengths and weaknesses of TB surveillance system in order to recommend improvements in performance and efficiency.

Methodology Descriptive evaluative study was conducted during March and April 2017. Qualitative and quantitative system attributes were assessed, using "Updated CDC Guidelines for Evaluating Public Health Surveillance Systems, 2001". Stakeholders were identified and interviewed. Semi-structured questionnaire was used for data collection. Desk review of documents and reports of 2016 was done.

Results The system was useful in estimating magnitude and trends effectively. System was simple in data flow, using standard case-definitions. System was flexible to adopt new changes. Having trained staff, and 90% forms completeness led to good data quality. System was poorly representative, covering only 30-40% of the district, because of less public-private collaboration and poor response from private-sector. Program's acceptance was good among all stakeholders. Manual data management at sentinel sites and quarterly reporting delays in epidemic detection and decision making resulted in poor timeliness. Sensitivity and positive predictive values were 56% and 40.5% respectively. Program depends mainly on international donors for funding, making it averagely stable. System is not integrated with other systems resulting in data duplication.

Conclusions Tuberculosis control program is performing good in most of the attributes. Establishing new TB sentinel sites with advanced rapid diagnostic services is recommended. Private practitioners need to be sensitized to enhance public-private collaboration. Reporting should be computerized and monthly for timely decision-making. Public funding needs to be increased. Integration of the system is recommended.

Surveillance System

Abstract Code: 2017-EOH-517
Presenter Name: Dr. Muhammad Saleem
Country: Pakistan
Presenter Email: saarcuk@yahoo.co.uk
Author: N. Jamal, A. Ameen

Title Review of the Influenza Sentinel Site Surveillance Data of Seasonal Influenza (H1N1) Cases Reported in Peshawar, Pakistan from December 2015 to April 2016

Background H1N1 2009 Influenza infection is a notifiable disease under International Health Regulations (IHR 2005). As the world is passing through post-pandemic phase and it is expected that the virus will continue to circulate in the years to come. Lack of information about seasonal influenza, identification of high risk persons and necessary preventive measures to be taken by the general public warranted the need for strengthening of the surveillance systems.

Purpose To detect unusual cases, clusters or outbreaks for action.

Methodology Using WHO standardized case definition, patients from the sentinel site were enlisted for further investigations. Hospital records of all the admitted patients were reviewed. Demographic information and medical illnesses record of the patients were collected for identification of risk factors if any. Throat swab samples for viral isolation were taken and sent to National Institute of Health (NIH) Islamabad. Descriptive analysis of the data was carried out.

Results 113 suspected Seasonal Influenza patients were reported from 31st December 2015 to 8th April 2016. Laboratory reports of all the patients were received from NIH wherein 11 (10%) patients were confirmed with Inf-A(H1N1)pdm09. A total of 41 suspected cases were reported to the sentinel site from Lady Reading Hospital, 15 cases from Khyber Teaching Hospital & 57 cases from Hayatabad Medical Complex. Among the suspected cases; 60 (53%) were males. Age range of the suspected cases was 2 months to 88 years with a mean age of 31 years. All these suspected cases belonged to different areas and there was no clustering of the cases.

Conclusions Guidelines including case definition, management and required preventive measures were sent to all district health offices and hospitals. Awareness sessions were arranged for healthcare staff with emphasis on case identification, personal protective measures and hand washing techniques.

Surveillance System

Abstract Code: 2017-HIV/STI-82

Presenter Name: Dr. Yasser Ghaleb
Country: Yemen
Presenter Email: y.ghaleb@ymail.com
Author: A. Al Serouri, M. Alamad, A. Alsoumainy

Title Evaluation of Blood Transfusion Services in Public and Private Blood Bank Centers, Sana'a Capital, Yemen, 2016

Background Evaluating Blood Transfusion Services (BTS) is the corner stone to ensure its proper delivery and avoiding related problems such as transfusion-transmitted infections (TTIs).

Purpose To evaluate BTS at the main blood banks of the Sana'a Capital.

Methodology The four main blood banks (BBs) in Sana'a Capital at the National Blood Transfusion and Research Centre (NBTRC), National Central Public Health Laboratories (NCPHL), University of Science and Technology Hospital (USTH) and Kuwait University Hospital (KUH) were evaluated according to World Health Organization safe BTS standards. Qualitative and quantitative data were collected through in-depth interviews with BBs officers using semi-structured questionnaires covering seven components: activities, quality assurance system (QAS), training, donation, grouping and compatibility testing, components, and screening for TTIs. An overall BTS score was calculated where < 60% was unsatisfactory, 60-79.9% satisfactory, = 80% highly satisfactory.

Results Although the four BBs are performing collection, screening, grouping and cross matching, none is performing therapeutic transfusion. While 75% of the BBs have received training in biosafety and half in Standard Operating Procedures (SOPs), no QAS in place at any. The four BBs depended on 71% of their transfusions on replacement family donors. Although all BBs reported using SOPs for compatibility testing, half do not perform Reverse Grouping and half do not keep patient/donor samples for the required minimum five days. All BB reported screening blood for HIV, Hepatitis B, and Hepatitis C using ELISA. The overall BTS score shows that only NBTRC achieved highly satisfactory and USTH achieved satisfactory scores.

Conclusions Findings highlight the increasing challenges facing BTS in Sana'a Capital especially lack of therapeutic transfusion, poor QAS, and predominant dependence on the family donors. Therefore, there is a need to develop and train on QAS and to increase awareness among public on importance of voluntary donation. A wider scale evaluation of BTS in whole Yemen is recommended.

Vector Borne Diseases

Abstract Code: 2017-OTH-421

Presenter Name: Dr. Farooq Gull Mohammad
Country: Pakistan
Presenter Email: farooqgm82@gmail.com

Title Dengue Outbreak Investigation in Gwadar City, Pakistan, November 2016

Background On 10th November 2016, District Health Officer reported Provincial Disease Surveillance & Response Unit Quetta about 25 suspected dengue cases from Gwadar City (total population =16365) and requested for investigation.

Purpose A FELTP fellow was deputed to Gwadar to confirm, investigate outbreak and suggest control measures on 12th November 2016.

Methodology Case was defined "as onset of fever, and two or more of following signs/symptoms; headache, retro-orbital pain, joint/bone pain, myalgia and petechial rash, in a resident of Gwadar city, from 1st November 2016 to 21st February 2017". Descriptive study was carried out to identify cases, affected areas and risk factors through active case finding. Hospital records were also reviewed. Blood samples were sent to NIH for laboratory confirmation.

Results Total 203 cases (overall AR=1.24%) were identified during active case finding and from records of only health facility in Gwadar city. Males were more affected (n= 126, 62%). Mean age was 28 years (range:6 to 60 years) and most affected age group was 25-29 years (n=41, 20%, AR=3.1%). Nausea/vomiting (95.1%) and myalgia (90.6%) were most frequent symptoms. All the cases had no travel history during last one month. Among 185 samples, 151 (81.6%) were dengue positive. First case reported on 1st November and peak was achieved on 12th November, but after on 21st February 2017 no case was reported. Four stagnant water pools with Larvae were identified.

Conclusions Presence of stagnant water pools in and around houses being breeding places for Aedes aegypti mosquitos was most probable cause of outbreak. Elimination of mosquito breeding sites, larvicidal activities and advocacy on use of protective measures against mosquito bites were recommended. Thermal fogging in densely populated localities and Indoor Residual Spraying (IRS) were carried out in 250 households. Sensitization and awareness on dengue control were conducted among community, schools, projects and key players(n=1225) in outbreak area.

Vector Borne Diseases

Abstract Code: 2017-EOH-341

Presenter Name: Dr. Farman Ali

Country: Pakistan

Presenter Email: drfarmanali.pk@gmail.com

Author: A. Khan, T. Ghafoor, J. Asghar

Title Cutaneous Anthrax Outbreak Investigation in Bajaur Agency, Federally Administered Tribal Area, Pakistan, August 2016

Background Anthrax is caused by Bacillus Anthracis, classified as A-priority Pathogen for biodefense with a high case fatality rate. On August 25, 2016, unusual deaths of a female and cattle in village Umarai, Bajaur were reported to health department.

Purpose An outbreak investigation team was constituted to assess the magnitude, identify risk factors and to implement appropriate control measures.

Methodology A case was defined as "Any person of village Umarai suffering from acute illness revealing a painless skin lesion developing over 2 to 6 days into eschar with surrounding edema and any of the symptoms: fever, malaise, myalgia, edema, lymphadenopathy or epidemiological evidence relating it to anthrax from 12th to 29th August 2016". Data was collected on a pre-tested structured questionnaire. Descriptive statistics were computed; attack rates and odds ratios determined at 95% confidence interval and p value < 0.05. Age and sex matched controls were selected from the same area with 1:3.

Results Total 12 cases were identified with male to female ratio of 11:1. Case Fatality Rate was 8.3 %(n=1). Mean age was 38.5 Years (Range: 16-65 Years). Fifty eight percent (n=8) cases were farmers. All the cases were involved in slaughtering of animals. All cases had Myalgia, Malaise and lymphadenopathy while 92% (n=11) cases had eschar and headache. Significant statistical association was found with having contact with open wounds/cuts (OR 23.8, 95% CI 3.82- 148.45, p value <0.05) low socioeconomic status (OR 8.8,95% CI 1.68- 46.70, p value <0.05). Laboratory results were found negative for Bacillus Anthracis probably due to prior antibiotics use.

Conclusions The outbreak investigation revealed strong association of Anthrax with low socioeconomic status and contact with open wounds and cuts. Community awareness regarding proper handling and disposal of infected animals and development of a coordinated strategy to establish surveillance system for zoonotic diseases by Health and Livestock departments were suggested.

Zoonotic Diseases

Abstract Code: 2017-EOH-306

Presenter Name: Dr. Shimaa Okasha

Country: Egypt

Presenter Email: drshosho79@yahoo.com

Author: S. Elshorbagy

Title Descriptive Analysis of Reported Cases of Avian Flu (H5n1) - Kafr Elsheikh, Egypt, 2015-2017.

Background Avian flu A (H5N1) is an influenza A virus subtype that infects mainly birds and it is highly contagious. It became endemic among birds in Egypt. Kafr Elsheikh is considered at risk governorate because 90% of its population breeds domestic birds in houses. The peak of avian flu in Kafr Elsheikh showed an outbreak in 2015.

Purpose This study aimed to describe reported cases of H5N1 in Kafr Elsheikh governorate, 2015-2017.

Methodology Analysis of surveillance data of H5N1 reported cases of Kafr Elsheikh were extracted from the National Electronic Disease Surveillance System (NEDSS).

Results Out of the 384 reported cases of avian flu from Kafr Elsheikh between 2015 and 2017; 120, 107, 157 cases were suspected in 2015, 2016 and 2017, respectively. In 2016 and 2017 there were no confirmed cases while in 2015 there were nine confirmed H5N1 cases, representing 2.3% (9/384) from the total suspected cases. Death occurred in one case with case fatality rate 11% (1/9). All confirmed cases were housewives with one infant. The most affected age group was between 35 and 65 years. All confirmed cases had bird exposure; 3 cases had exposed to diseased bird, 1 exposed to dead bird, 5 exposed to birds during slaughtering.

Conclusions After the 2014-2015 H5N1 outbreak, there was an apparent 2 years absence of confirmed cases in Kafr Elsheikh. Enhancement of the surveillance capacity was reflected by the increasing number of suspected cases. Increasing awareness of women to follow the safe breeding practices should be maintained. There is a need to enhance the capacity of community health workers to promote and sustain wider community behavioral change and raise awareness about the disease and the correct breeding methods.

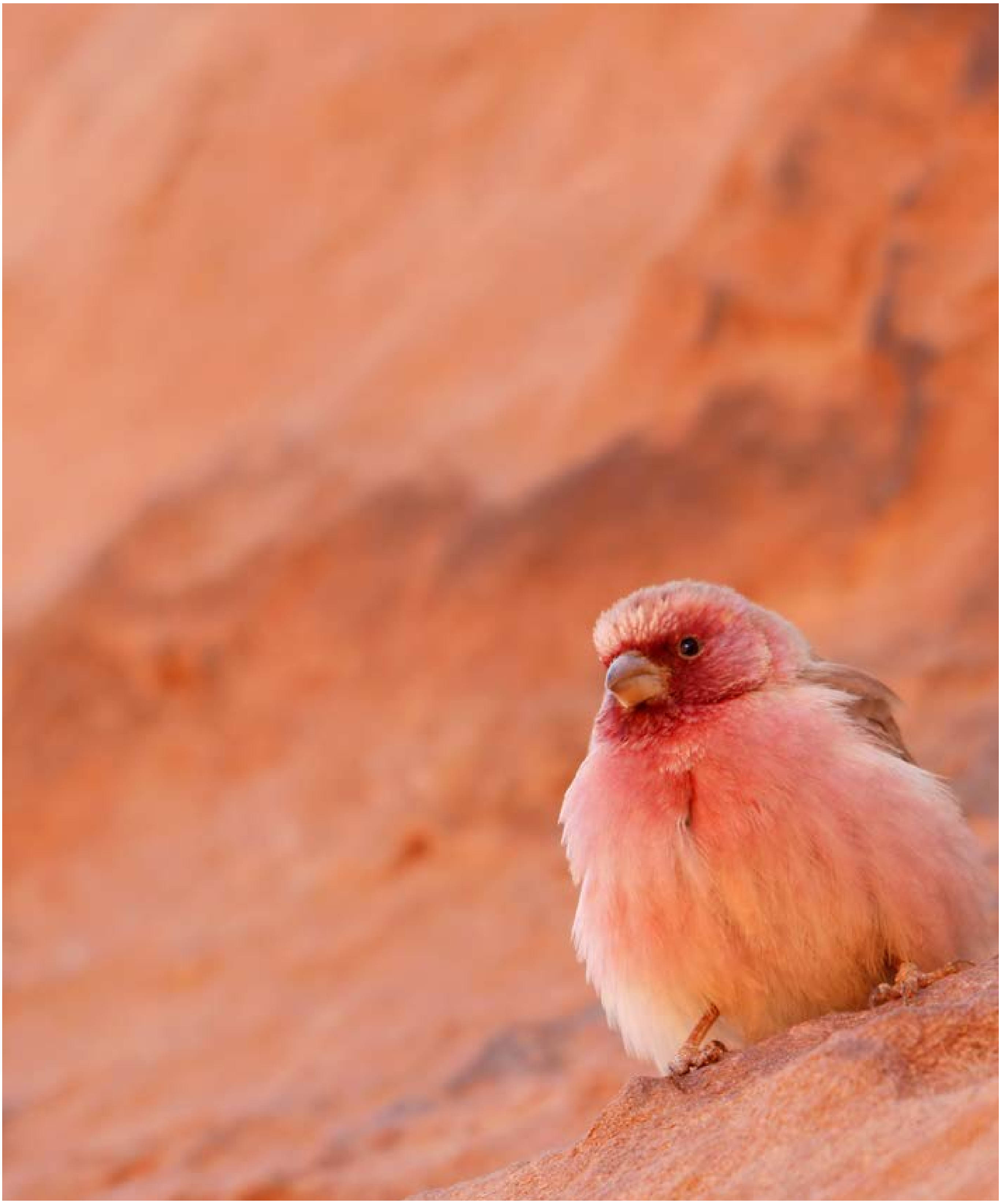

> The way we work in public health is, we make the best recommendations and decisions based on the best available data

Tom Frieden

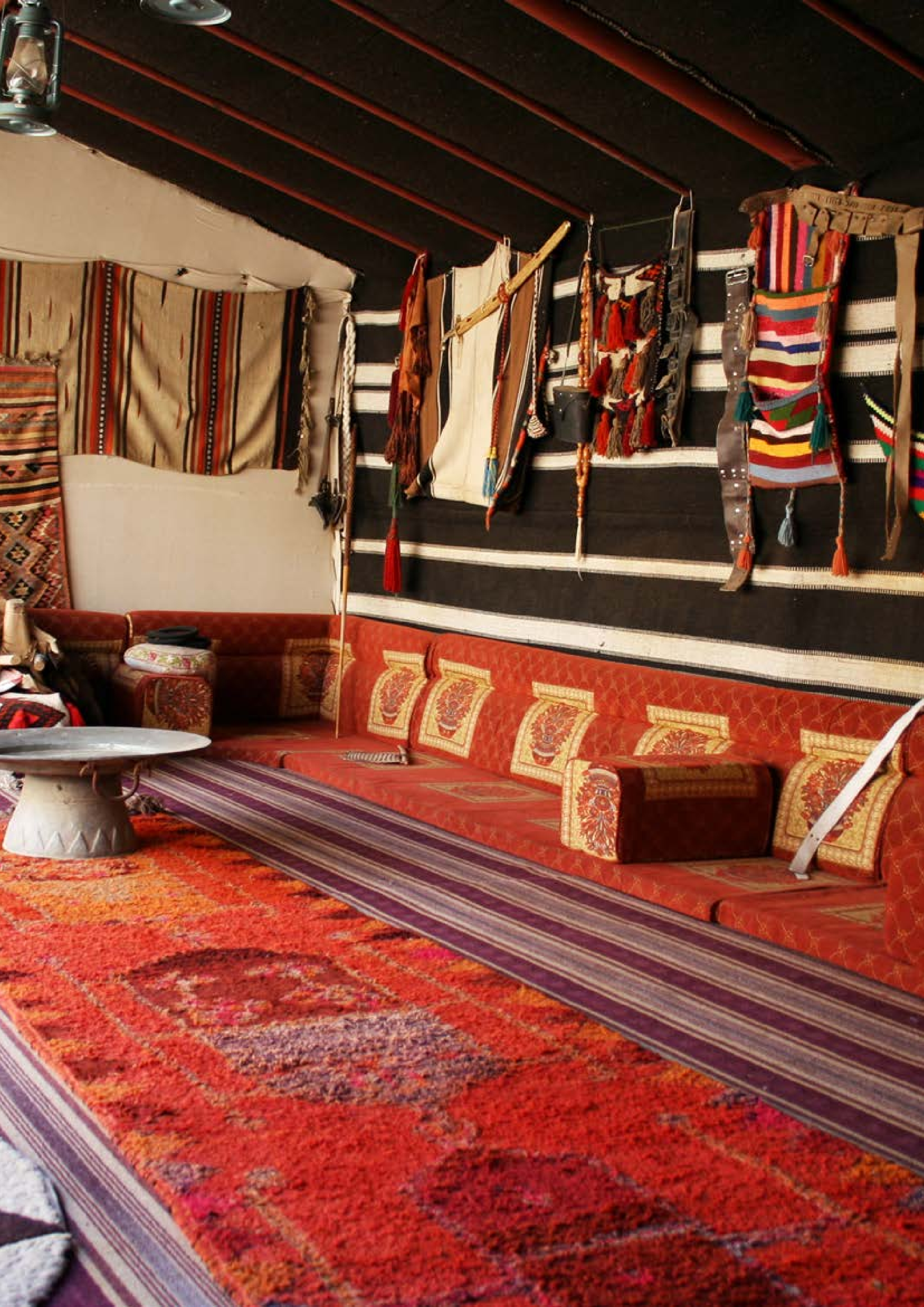

Amman, Jordan
Shmeisani, Abdallah Ben Abbas Street,
Building No 42,
Tel: +962-6-5519962
Fax: +962-6-5519963
Email: info@emphnet.net
Web: www.emphnet.net

Global Health Development

EMPHNET
The Eastern Mediterranean
Public Health Network

GHD and EMPHNET: working together for better health
